# Synergistic C–H bond activation across molybdenum–iridium multiply bonded complexes: a cascade of transformations†

**DOI:** 10.1039/d5sc03465e

**Published:** 2025-07-07

**Authors:** Zachary Dubrawski, Iker Del Rosal, Erwann Jeanneau, Laurent Maron, Chloé Thieuleux, Clément Camp

**Affiliations:** a Laboratory of Catalysis, Polymerization, Processes and Materials (CP2M UMR 5128), CNRS, Universite Claude Bernard Lyon 1, CPE-Lyon, Institut de Chimie de Lyon 43 Bd du 11 Novembre 1918 F-69616 Villeurbanne France clement.camp@univ-lyon1.fr; b LPCNO, Université de Toulouse, INSA Toulouse 135 Avenue de Rangueil Toulouse 31077 France; c Centre de Diffractométrie Henri Longchambon, Universite Claude Bernard Lyon 1 5 Rue de la Doua 69100 Villeurbanne France

## Abstract

Heterobimetallic compounds offer unique opportunities for activating substrates through cooperative interactions between two distinct metal centers, potentially leading to catalytic reactivity beyond the reach of monometallic systems. The pairing of molybdenum with iridium has recently shown remarkable performance in heterogeneous catalytic and electrocatalytic processes, yet remains surprisingly unexplored in the context of homogeneous catalysis and well-defined molecular complexes. In this work, we address this gap by reporting the synthesis and characterization of complexes featuring rare molybdenum–iridium multiple bonds: Cp*Ir(H)Mo(NMe_2_)_3_, 1 and (Cp*IrH_2_)_2_Mo(NMe_2_)_2_, 2. Both complexes undergo electrophilic insertions of heteroallenes (CO_2_, *t*BuNCO) into the ancillary amido ligands. In the case of compound 1, the insertion of *tert*-butyl isocyanate initiates a unique cascade of bond breaking/forming events – including triple C–H activations and C_sp^3^_–C_sp^3^_ coupling – ultimately yielding a Mo(vi) metallacyclopropane complex, 5. Addition of multiple equivalents of isocyanate instead interrupts this mechanism, leading to C–N and C–O bonds cleavage and the formation of aminocarbyne and imido ligands bridging the Ir and Mo centers, along with a molybdenum(vi)-oxo moiety. This unprecedented reactivity, mediated by a key reaction intermediate featuring an unsupported Mo–Ir quadruple bond, illustrates the interest of heterobimetallic compounds for complex bond activation and reorganization.

## Abstract 2

Heterobimetallic compounds offer unique opportunities for activating substrates through cooperative interactions between two distinct metal centers, illustrated here on molybdenym–iridium species promoting a cascade of bond breaking/forming events.

## Graphical abstract

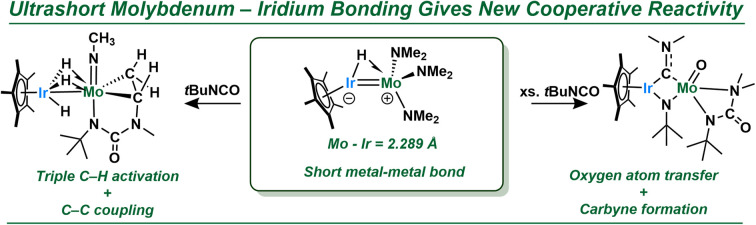

## Introduction

The first discovery of dimolybdenum paddlewheel complexes by Mason in 1965 launched a near-constant excitement for interesting multiple bonding in metal complexes.^1–5^ Since then, many supported multiply bonded bimetallic complexes have been reported (*i.e*. with a ligand bridging both transition metals in the complex), however unsupported systems present a particular challenge.^6–8^ Ligands must be small enough to allow for efficient orbital overlap between transition metal centers and avoid bridging across the metals, yet large enough to stabilize the often reactive transition metal complexes.^9,10^ Perhaps due to added complexity, species featuring unsupported multiple bonding between two different transition metals have rarely been investigated, with only a few reports in the literature.^11–13^

This is surprising, given the growing interest in heterobimetallic transition metal complexes for promoting challenging bond activations.^14–16^ Indeed, by utilizing the electrons localized within metal–metal bonds and the inherent polarity of these architectures, such systems can access unique reactivity which is often beyond that of monometallic or homobimetallic congeners.^17^ A prototypical example is the phosphinoamide supported Zr–Co complexes from Thomas and coworkers, which can oxidatively add electrophiles such as CO_2_ or methyl iodide across the two metal centers.^18^ Building on this strategy, this group^19^ and others have since employed with success heterobimetallic complexes in a variety of catalytic reactions.^20–23^ However, many heterobimetallic systems featuring multiple metal–metal bonds rely on polydentate ligands to drive their assembly. While effective for structural organization, these bulky ligands can significantly hinder reactivity, particularly when concerted substrate activation across both metal centers is required, as ligand steric encumbrance can block access to the reactive site.

Unsupported metal–metal bonded systems, or those bridged by small hydride ligands, offer a promising strategy to overcome this limitation and access more reactive species. For example, in our prior work, we demonstrated that Ta–Ir species are highly active hydrogen isotope exchange catalysts as a result of a cooperative C–H bond activation across the two metals.^28^ In pursuit of a more covalent metal–metal bonding arrangement, we identified molybdenum as a promising candidate for the development of heterobimetallic complexes. While the Mo–Ir pairing has recently shown exceptional reactivity in heterogeneous catalysis and electrocatalysis,^29–33^ it remains surprisingly underexplored in the context of homogeneous catalysis and molecular complexes, with very few well-defined Mo–Ir compounds reported to date.^34,35^ To address this gap in literature, we report here the synthesis and characterization of rare molybdenum–iridium complexes featuring short, multiple metal–metal bonds. We further explore their reactivity towards electrophilic heteroallenes (CO_2_, *tert*-butyl isocyanate), which triggers unexpected cascade transformations involving multiple C–H, C–C, C–N, and C–O bond-breaking or -forming events. The underlying reaction mechanisms were rationalized through a combination of experimental studies and computational analysis.

## Results and discussion

Addition of one equivalent of tetrakis(dimethylamido)molybdenum(iv), Mo(NMe_2_)_4_, to tetrahydrido(pentamethylcyclopentadienyl)iridium(v), Cp*IrH_4_, generates complex Cp*Ir(H)Mo(NMe_2_)_3_, 1, in 75% yield (Scheme 2). Complex 1 is diamagnetic with NMR data consistent with the structural assignment. The hydride signal in the ^1^H NMR spectrum (−6.5 ppm in C_6_D_6_) integrates for only one proton, suggesting the loss of three hydrides during the reaction (Fig. S2†). One hydride is lost in the protonolysis of one dimethylamido ligand, releasing one equiv. of free dimethylamine, as can be observed by monitoring the reaction by ^1^H NMR (Fig. S6†). This reaction is typical of the Brønsted acidic Cp*IrH_4_ starting material and has been used in our lab with great success to prepare Ir-based heterobimetallic compounds from metal-alkyl reagents.^11,22,36,37^ Here, we show that this approach can be extended to amido metallic species, expanding the scope of this synthetic strategy. Performing the reaction in a J-Young NMR tube fully filled with C_6_D_6_ allows for the detection and quantitation of H_2_ generated, and confirming the release of a stoichiometric amount of dihydrogen (see Fig. S6†).

**Scheme 1:**
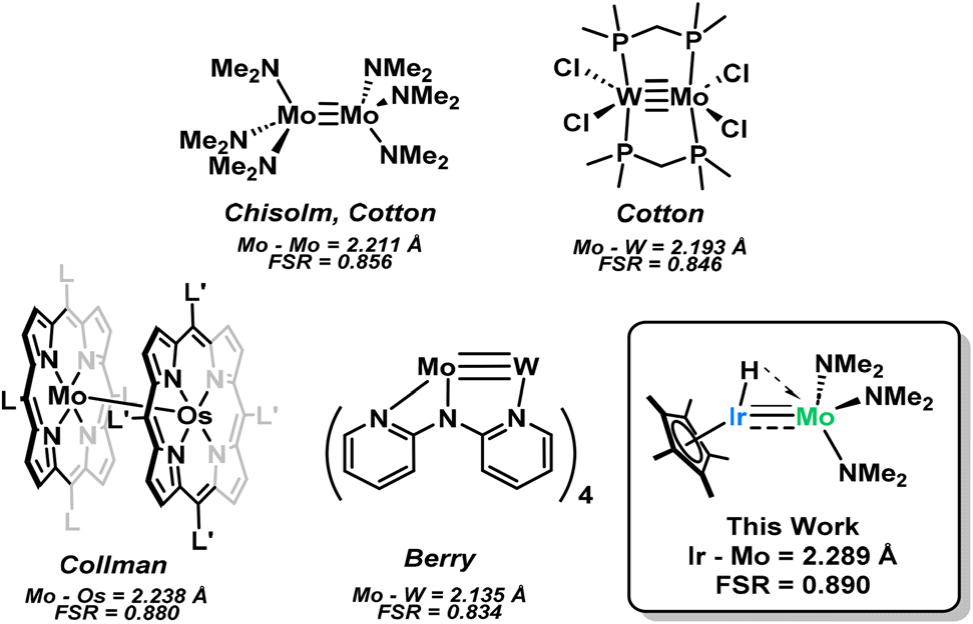
Representative examples of molybdenum-based homo- and heterobimetallic complexes reported in the literature. Bond lengths and structural representations are taken from the respective article as described in the original text. A table with further examples is located in the ESI (Table S1).†^24–27^

Two resonance structures, both obeying the 18-electron rule for each metal center, can be proposed for 1, as shown on Scheme 1. According to Green's formalism, where the hydride is treated as X-type, the amidos as XL-type and the Cp* ligand as an XL_2_-type ligands, the total oxidation state across both metals in compound 1 is +5 (total d electron count of 10). Starting from monometallic precursors with a combined formal oxidation state of +9, this decrease is consistent with the net double reductive elimination of H_2_ and HNMe_2_. How this total oxidation state is partitioned between the two metals, however, remains open to interpretation. One approach is to consider the metal–metal bonds as nonpolar, leading to formal oxidation states of +2 for Ir (d^7^) and +3 for Mo (d^3^) in the neutral resonance form 1a, or +1 for Ir (d^8^) and +4 for Mo (d^2^) considering the zwitterion form 1b. Alternatively, given the higher electronegativity of Ir compared to Mo and the polar nature of the metal–metal bonding, an uneven electron distribution across the metal–metal bond can be proposed. In the extreme case, this would correspond to Mo in the +6 state (d^0^) and Ir in the −1 state (d^10^), a description that is chemically questionable but could offer a rationale for the diamagnetism observed in compound 1. While oxidation state formalism plays a valuable role in our understanding of chemical bonding and reactivity, it remains an inherently arbitrary method for partitioning electrons between atomic centers. For this reason, we refrain assigning specific oxidation states to the metals in such compounds throughout the remainder of this article.

**Scheme 2:**
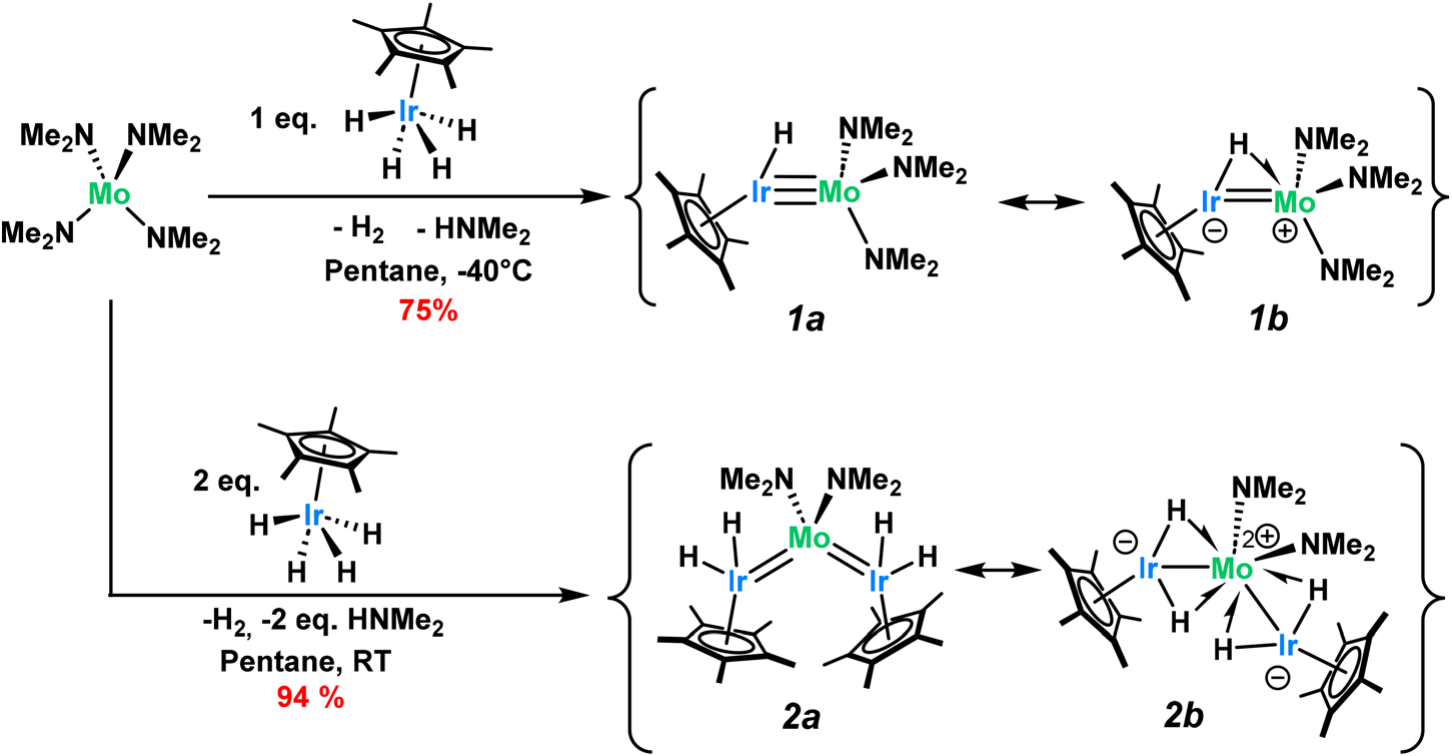
Synthesis of the multiply bonded iridium–molybdenum complexes.

Another point of discussion is whether the hydride is terminal (resonance form 1a in Scheme 2), or engages in a three-center two-electron (3c–2e) interaction, in which the iridium hydride bond donates electron density to the molybdenym center, in a Ir–H^−^ → Mo^+^ agostic-type of bonding. The latter scenario is illustrated in form 1b in Scheme 2, using a half-arrow notation originally introduced by Parkin and Green,^38^ and subsequently applied to related systems.^39–41^ Depending on the limiting bonding scenario considered, the metal–metal bond order is adjusted (triple or double) to satisfy the 18-electron rule. Further characterization of complex 1 was thus carried out to gain a deeper understanding of its structure.

**Fig. 1:**
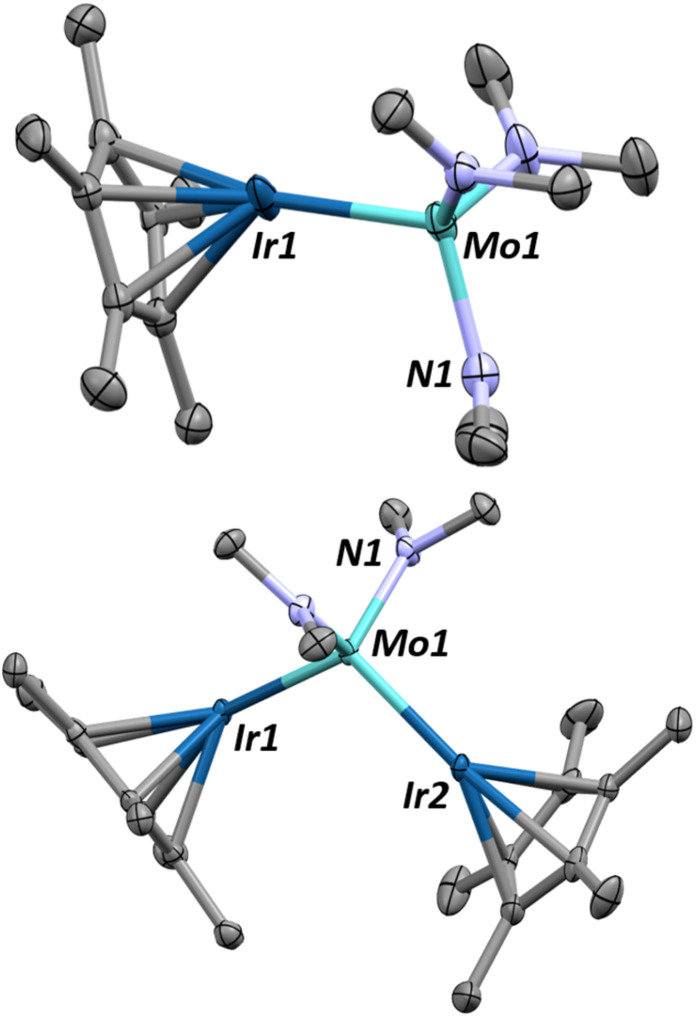
X-ray diffraction crystal structures of complexes 1 (top) and 2 (bottom) with thermal ellipsoids drawn at 50% probability and hydrogens removed for clarity. Relevant structural parameters for complex 1: Mo–Ir = 2.2889(7) Å, FSR_MoIr_ = 0.89, Mo–N = 1.935(5)–1.955(5) Å. ∠Cp*_centroid_–Ir–Mo = 156.0(1)°, *τ*_4_ = 0.93. Relevant structural parameters for complex 2: Mo–Ir = 2.3946(5)–2.4075(5) Å, FSR_MoIr_ = 0.93–0.94, Mo–N = 1.945(4)–1.948(5) Å. ∠Cp*_centroid_–Ir1–Mo = 154.0(1)°, ∠Cp*_centroid_–Ir2–Mo = 151.5(1)°, *τ*_4_ = 0.93.

Single crystals of 1 can be obtained by cooling a saturated pentane solution of complex 1 to −40 °C (Fig. 1). The most pertinent feature of the solid-state structure is the very short metal–metal bond distance of 2.2889(7) Å. Formal Shortness Ratio (FSR, = MM′ distance/sum of MM′ covalent radii) introduced by Cotton *et al.* can be a good indicator for the qualitative identification of multiply bonded species from structural data.^42^ Structural analysis of 1 gives an FSR of only 0.89, suggestive of a Mo–Ir bond of 2 or above. While other Mo–Ir heterobimetallic complexes have been reported in the literature, to our knowledge, none of the other crystallographically characterized examples available in the CCDC database exhibit an FSR below 1.^34,35,43–47^ The sum of the Mo–N–C and C–N–C angles at each amido ligand is close to 360° (359.6(1)°, 358.1(1)° and 359.7(1)°), consistent with sp^2^ hybridization of the nitrogen atoms, which enables them to act as 3e^−^ donors (LX-type ligands) to maintain a 18-valence electron configuration.

Although assigning hydrides adjacent to the heavy nuclei in X-ray crystallography was not possible, the significant tilting of the Cp* ligand with respect of the Mo–Ir multiple bond (Cp*_centroid_–Ir–Mo angle of 156.08°) is indicative of the presence of a hydride on the Ir center.^48^ The presence of the hydride is further supported by the Diffuse Reflectance Infrared Fourier Transform (DRIFT) spectrum for 1, which exhibits one signal typical of a iridium hydride stretch (1990 cm^−1^, Fig. S33†).^49,50^

The geometry of complex 1 was optimized at the DFT level (B3PW91 functional, Fig. S44†). The optimized structure compares well with the experimental one and among others the Mo–Ir distance is perfectly reproduced (2.30 Å *vs.* 2.29 Å experimentally). Bonding analysis confirms the presence of metal–metal bonding interactions at the Natural Bonding Orbital (NBO level). Indeed, two almost purely covalent Mo–Ir bonds (50–50 and 55–45) are found at the NBO level. One Mo–Ir bond involves overlap between sd hybrid orbitals while the second one implies pure d atomic orbitals (see Fig. S44†). The associated Wiberg Bond Indexes (WBI) are 1.67 indicating the presence of a highly covalent Mo–Ir double bond. A substantial donation from an iridium d lone pair into an empty d orbital at Mo is observed at the second order. The latter may explain the experimental observation of a bond order above two, but a backdonation from an occupied lone pair on Ir to an antibonding Mo–Ir bond is also observed so that complex 1 is better described with a Mo–Ir double bond.

The computed Mo–Ir–H angle in 1 is estimated to be 65°, which is compatible with the presence of a 3c–2e interaction. The Mo–H distance in 1 (2.19 Å) is significantly longer than typical values observed in classical μ-hydride molybdenum complexes (1.83–1.87 Å)^51,52^ while the computed Ir–H bond is notably shorter at 1.65 Å, despite Ir having a larger atomic radius than Mo. This suggests an asymmetric hydride interaction, with stronger bonding to Ir. The latter is corroborated by an analysis of the Wiberg bond indexes (WBIs, Fig. S44†). The Ir–H WBI is 0.57, in line with a mainly covalent interaction, whereas the Mo–H WBI is 0.30, in line with some electron delocalization from the Ir–H bond onto an acceptor orbital on Mo. These data support the description of 1 as stabilized by partial Ir–H bond donation to the [Mo(NMe_2_)_3_]^+^ fragment. Assuming an η^2^ interaction between the Ir–H bond and Mo, and that each terminal amido group donates three electrons, this necessitates a Mo=Ir double bond to satisfy the 18-electron rule – an assignment consistent with natural bond orbital (NBO) analysis. Additionally, the Ir–H^−^ → Mo^+^ donation shortens the Ir–Mo distance further, in agreement with the XRD data. Naturally, the bonding lies along a continuum between the two limiting forms 1a and 1b, as illustrated in Scheme 2.

The trinuclear complex (Cp*IrH_2_)_2_Mo(NMe_2_)_2_, 2, is similarly synthesized in 94% yield through the addition of two equivalents of Cp*IrH_4_ to Mo(NMe_2_)_4_ and the release of one equivalent of dihydrogen and two equivalents of dimethylamine (Scheme 2). The X-ray crystal structure of complex 2, shown on Fig. 1-bottom, reveals a trinuclear MoIr_2_ core with a tetrahedral molybdenum center (*τ*_4_ = 0.93). The Mo–Ir bond distances range from 2.3946(5) to 2.4075(5) Å (FSR = 0.93–0.94) indicating some multiple bonding character but less than that of 1. Again, the hydrides could not be located from the Fourier difference map of the X-ray crystallography experiment, however the hydride signals in the ^1^H NMR spectrum for 2 (−10.23 ppm, 4H, Fig. S7†) and the clear hydride stretching frequencies in the DRIFT spectrum (2022 and 1929 cm^−1^, Fig. S34†) confirm their presence. Similarly, the Cp*_centroid_–Ir–Mo angles (151.5(1)°–154.0(1)°) give a qualitative understanding of the hydride positions in the solid state structure.

Complex 2 was also investigated computationally (Fig. S45†). The optimized structure compares well with the experimental one. As for complex 1, the Mo–Ir distances are correctly reproduced (2.44 Å *vs.* 2.40–2.41 Å experimentally). The bonding analysis at the NBO level indicates the presence of two single covalent Mo–Ir bonds (55–45) with an associated WBI of 1.0, as a result of overlap between d orbitals on Mo and Ir. At the second-order level, there is evidence of donation from an occupied Mo orbital to vacant orbitals on Ir, which could suggest a bond order greater than one. However, simultaneous backdonation into the Mo–Ir bonding orbitals is also observed. As a result, the bonding is more accurately described as comprising two polarized Mo–Ir single bonds. The bonding situation for the hydrides in 2 is similar to that in 1 (see Fig. S45†).

**Scheme 3:**
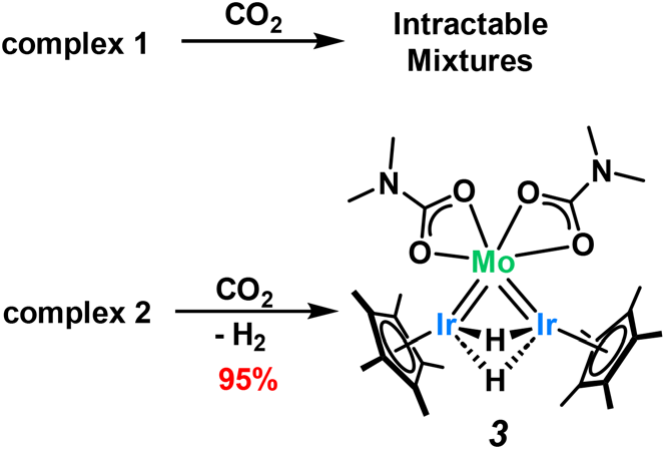
Reaction of complexes 1 and 2 with CO_2_.

Carbon dioxide rapidly reacts with complex 1 to give intractable mixtures in which the major component is paramagnetic (Fig. S10†). This complex mixture of products persists across different reaction conditions, including changes in solvents, in the absence of light, varying the concentration or stoichiometric amounts of CO_2_ and reduction of the temperature. In contrast, complex 2 cleanly reacts with CO_2_*via* insertion of CO_2_ into the dimethylamido ligands, affording the carbamato complex, 3, in 95% yield (Scheme 3). ^1^H NMR spectroscopic analysis shows a shift of the dimethylamido signals from 3.49 ppm to 2.37 ppm, with the appearance of a resonance in the ^13^C NMR spectrum at +169 ppm, corresponding to the carbamato carbonyl (Fig. S12†). The DRIFT spectrum for 3 exhibits a characteristic *υ*_CO_ signal at 1570 cm^−1^, further confirming the formation of carbamato moieties (Fig. S35†). The hydride ^1^H NMR signal shifts from −10.23 ppm in 2 to −6.54 ppm in 3, however the integral of this signal falls to two protons (Fig. S11†) indicating that the CO_2_ insertion is coupled with an irreversible loss of one equivalent of dihydrogen (as confirmed by ^1^H NMR reaction monitoring, see Fig. S15†). Interestingly, we see no indication of CO_2_ insertion into the M–H bonds of 2 despite the literature precedent for Mo and Ir hydride insertion.^53,54^

**Fig. 2:**
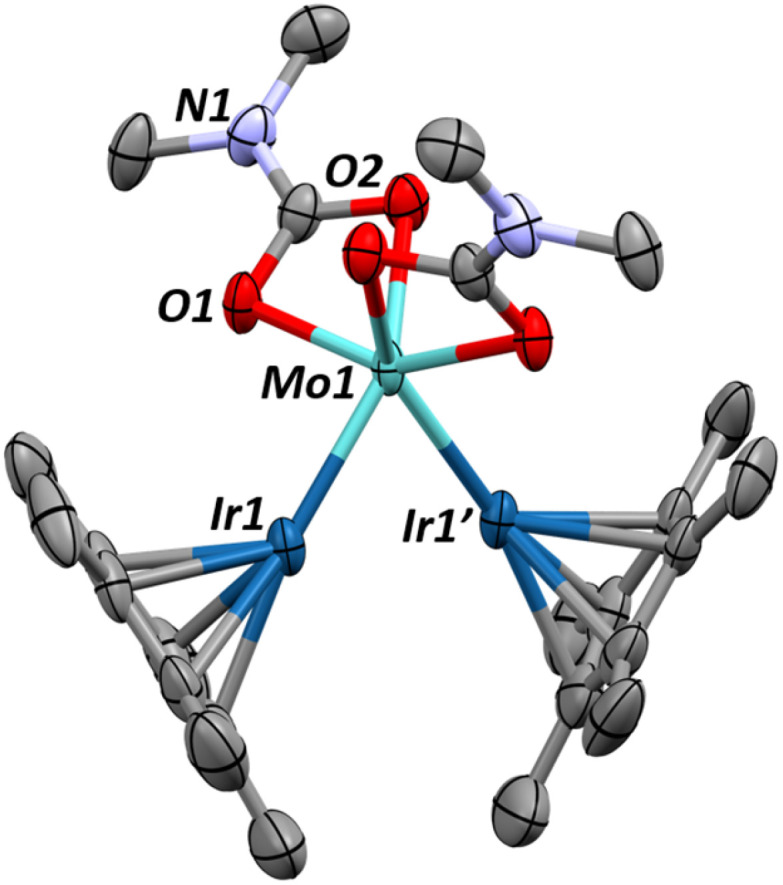
X-ray crystal structure of complex 3, grown from a saturated pentane solution, reveals a major structural change upon CO_2_ insertion into the dimethylamido ligands. Thermal ellipsoids shown at 50% and hydrogens removed or clarity. Relevant structural parameters are as follows: Mo–Ir = 2.37659(6) Å, FSR_MoIr_ = 0.93, Ir–Ir = 2.7722(3) Å, FSR = 1.10, Mo–O = 2.199–2.149 Å, ∠Ir–Mo–Ir = 71.3(1)°, ∠Cp*_centroid_–Ir–Mo = 155.7(1)°, ∠Cp*_centroid_–Ir1–Ir1′ = 139.5(1)°.

This CO_2_ insertion also leads to a surprising structural rearrangement as revealed by the single crystal X-ray structure of complex 3 (Fig. 2). Here, the iridium centers bend down toward one another, reducing the Ir–Mo–Ir′ angle from 108.8(1)° in 2 to 71.4(1)° in 3. The long interatomic distance between the Ir centers (2.7722(3) Å, FSR = 1.10) suggests an absence of formal Ir–Ir′ bonding interaction, a conclusion further supported by computational analysis (see below). We instead propose that the close proximity of the Ir centers is maintained by two bridging hydrides. The Mo–Ir bond distance (2.3759(6) Å, FSR = 0.93) still gives a FSR well below 1, suggesting that the multiple bonding character of the Mo–Ir bonding is maintained through this structural change. Note that the carbamato oxygens are solely bound to molybdenum.

As already carried out for complexes 1 and 2, DFT optimizations were performed for complex 3. The resulting optimized geometry compares satisfactorily with the experimental one (Fig. S46†). Among other, the Mo–Ir distances are nicely reproduced (2.32 and 2.36 Å *vs.* 2.37 Å experimentally). The Ir–Ir distance is a bit longer than the experimental one (2.85 Å *vs.* 2.78 Å experimentally), but still in an acceptable range. NBO-level analysis of bonding indicates the presence of two Mo–Ir double bonds, which are polarized toward the Ir center (60%) and primarily imply d–d orbital overlap. These polarized double bonds explain the relatively low Mo–Ir WBI of 1.3–1.5. In contrast, the Ir–Ir interaction is only detected at the second order donor–acceptor level, which is in line with the low WBI of 0.35.

**Fig. 3:**
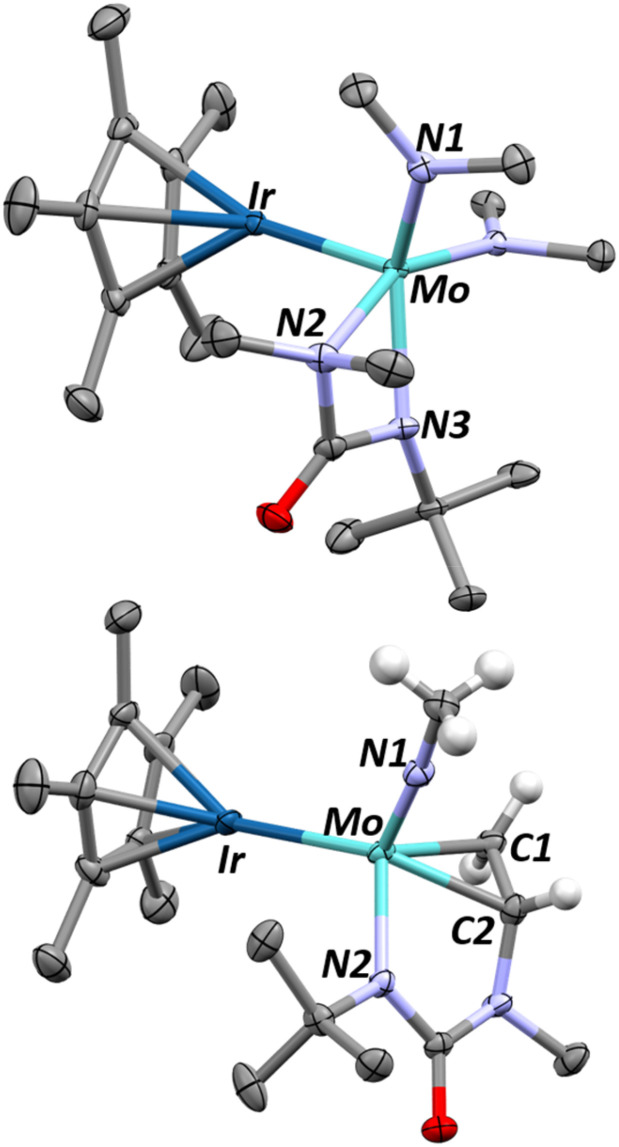
X-ray diffraction crystal structures of complexes 4 (top) and 5 (bottom). Thermal ellipsoids are shown at 50% probability and most hydrogens are removed for clarity. Relevant structural parameters for complex 4: Mo–Ir = 2.2646(7) Å, FSR_MoIr_ = 0.89, Mo–N1 = 1.957(4) Å, Mo–N2 = 2.286(4) Å, Mo–N3 = 2.139(4) Å, ∠Cp*_centroid_–Ir–Mo = 167.1(2)°. Relevant structural parameters for complex 5: Mo–Ir = 2.6229(4) Å, FSR_MoIr_ = 1.04, Mo–N1 = 1.724(3) Å, Mo–N2 = 2.080(4) Å, Mo–C1 = 2.175(5) Å, Mo–C2 = 2.169(4) Å, C1–C2 1.419(7) Å, ∠Cp*_centroid_–Ir–Mo = 175.3(1)°.

We hypothesize that 1 undergoes a similar CO_2_ insertion as 2 but does not stop at the carbamato ligated product. Accordingly, we sought alternatives to further explore this chemistry. Isocyanates are isoelectronic with CO_2_ and often used to investigate mechanisms in CO_2_ activation.^55–57^ Addition of one equivalent of *tert*-butylisocyanate (*t*BuNCO) to 1 quickly generates a new product, 4, in 95% yield as quantified by ^1^H NMR spectroscopy. Complex 4 was isolated (65% recovered yield after recrystallization) by quickly (within 10 min) removing the volatiles from a room temperature reaction mixture of 1 and one equivalent of *t*BuNCO. X-ray quality single crystals of complex 4, obtained from a saturated pentane solution of this reaction mixture cooled to −40 °C, confirms the insertion of *t*BuNCO into a dimethylamido ligand generating the κ^2^-ureate complex 4 (Fig. 3 – top), reminiscent of isocyanate insertions into alkoxide ligands of structurally similar molybdenum complexes.^58,59^ Computational analysis of this insertion confirms the low enthalpic barrier (19.9 kcal mol^−1^, Scheme 5) through a classical C–N double insertion transition state (TS), previously explored in the context of polymerization catalysis.^60^ The very short Mo–Ir bond length (2.2646(7) Å, FSR = 0.89) has not significantly changed from 1, indicating the electronic nature of the complex has not been significantly altered. The Cp* ligand tilting (Cp*–Ir–Mo angle of 167.1(2)°) indicates that the terminal hydride is still present. The presence of the hydride is further confirmed by DRIFT spectroscopy, revealing an intense signal at 2153 cm^−1^, assigned to *ν*(Ir–H) (Fig. S36†).^49^

A clean ^1^H NMR spectrum could be obtained before significant conversion to complex 5 (see below). This ^1^H NMR spectrum reveals a hydride resonance shift from −6.40 ppm to −9.85 ppm and significant broadening of the *N*-methyl signal of both the dimethylamido and ureate ligands (3.49 ppm and 2.89 ppm respectively, Fig. S16†). The formation of 4 is computed to be only lightly exothermic (−5.6 kcal mol^−1^, Scheme 5) and given the low barrier we anticipate a chemical equilibrium between 1 and 4. Exchange spectroscopy (EXSY) 2D NMR experiments show a broad cross peak between the two signals and those of remaining 1 in solution (Fig. S19†), hinting at chemical exchange on the NMR timescale. Unfortunately, due to the low barrier to further transformation, Van't Hoff analysis on the equilibrium could not be performed (see below). VT-NMR spectra collected in toluene-d8 allow some deconvolution of these signals into four broad singlets, each integrating for 3H, but assignment remains unclear (Fig. S17†). A ^13^C NMR spectrum could also be obtained at −25 °C and features a characteristic ureato signal at +164 ppm (Fig. S18†), allowing for confirmation of the isocyanate insertion.^61^

**Scheme 4:**
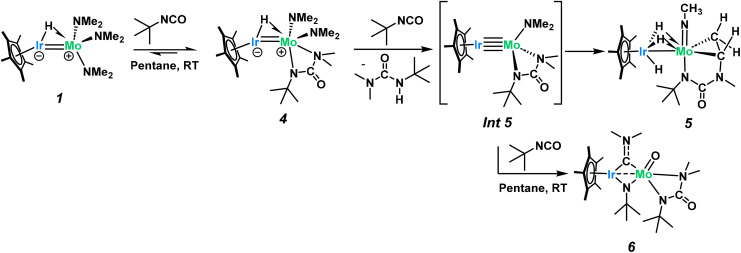
Complex 1 reacts with one equivalent of *t*BuNCO in a clean dimethylamine insertion to yield complex 4, which then undergoes a triple C–H activation process to yield the new product 5. Addition of 5 equivalents of *t*BuNCO to 1 instead leads to multiple C=N and C=O bond activations to yield complex 6.

Complex 4 is metastable and slowly converts at room temperature into complex 5 (Scheme 4). The ^1^H NMR spectrum of 5 shows a shift of the hydride signal from −9.85 to −12.55 ppm with an increase in the integral from one to three protons. Three characteristic signals, corresponding to the diastereotopic protons of a metallacyclopropyl fragment, are observed in the alkyl region at +4.20 ppm (doublet of doublets), +2.69 ppm (pseudotriplet) and +2.17 ppm (doublet of doublets). Each of these metallacyclopropyl signals integrates for one H showing typical ^2^*J* and ^3^*J* coupling constants (6.5 and 9.7 Hz, respectively, see Fig. S20†).

A single crystal of 5, suitable for X-ray diffraction, was grown from a saturated pentane solution cooled to −40 °C. The resulting structure (Fig. 3-bottom) unequivocally confirms the product, formed in an apparent triple C–H activation process combined with C_sp^3^_–C_sp^3^_ bond formation to give a *tert*-butyl-imido metallacyclopropane complex. The Ir–Mo distance in 5 is substantially lengthened from 2.2889(7) Å in 1 to 2.6224(4) Å (FSR = 1.04), in 5, precluding multiple bonding between Mo and Ir. The Cp* ligand is nearly perpendicular to the Mo–Ir axis, with a Cp*_centroid_–Ir–Mo angle of 175.3°, as a result of three hydrides bridging the two metals, consistent with architectures previously reported in our group.^37,50,62,63^

The presence of three hydrides is confirmed by ^1^H NMR (Fig. S20†) and the DRIFT spectrum of 5, displaying several metal-hydride stretching signals between 1971 and 1780 cm^−1^ (Fig. S37†). The newly formed carbon–carbon bond between C1 and C2 (Fig. 3) is 1.419(7) Å long, which lies between typical C–C single and double bond lengths.^64^ The considerable C–C bond elongation compared to free ethylene (1.34 Å) suggests that the structure is better described as a metallacyclopropane complex rather than olefin-type bonding arrangement.^65,66^ The Mo–C bond lengths (Mo–C = 2.175(5) Å, 2.169(4) Å) agree with this assignment as well with typical metallacyclopropane Mo–C lengths of 2.209 Å.^67^ Previous examples of α-*N*-metallacyclopropanes are extremely limited, however a previously reported tantalum complex does show diasterotopic ^1^H NMR resonances for the M–CH_2_ at about 4 ppm and a C1–C2, bond distance of 1.468(8) Å, agreeing well with our results.^68^ The molybdenum imido bond length (Mo–N1 = 1.724(3) Å) is consistent with other examples of Mo(vi) alkylimido complexes.^69,70^

DFT calculations were carried out on complex 5 (Fig. S48†). The optimized geometry compares well with the experimental one (Mo–Ir distance of 2.66 Å *vs.* 2.62 Å experimentally and two Mo–C bonds of 2.16 Å *vs.* 2.17 Å experimentally). The NBO analysis indicates the presence of one polarized Mo–Ir single bond and two Mo–C single bonds (WBIs of 0.6–0.7). This further support the metallacyclopropane description of this complex proposed experimentally. Two hydrides appear to engage in 3c–2e bridging interactions (Ir–H distances of 1.65–1.66 Å and Mo–H distances of 2.02–2.03 Å) while the third hydride is best described as terminal (longer Mo–H distance of 2.24 Å). This description is in agreement with an 18 electron count for Ir (assuming a XL_2_-type Cp* ligand, 3 X-type hydrides and the metal–metal bond) and an 18 electron count for Mo (assuming 2 X-type alkyls, a X-type amido, a X_2_L-type imido, 2 agostic Ir–H interations and the Mo–Ir bond).

**Scheme 5:**
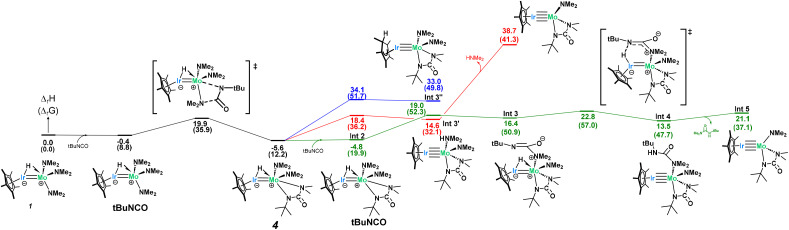
Computed mechanism for the formation of complex 4 and the key intermediate, Int 5.

Computational analysis to examine the mechanism of 4 going to 5 was found to be quite complex and necessitated a deeper investigation into the initial stability of complex 4 (Scheme 5). Given the loss of both a dimethylamido and a hydrido ligand in 5, the initial loss of dimethylamine from complex 4 was hypothesized but found to be exergonically inaccessible at room temperature (Δ*G*_r_ = 41 kcal mol^−1^, Scheme 5-red). However, since 1 and 4 are in equilibrium, there exists a concentration of free *t*BuNCO available in solution for a second NMe_2_ insertion. The associated barrier is similar to the one found for the formation of complex 4 (23.8 kcal mol^−1^) and yields a rather unstable intermediate (Int 3, 16.4 kcal mol^−1^) which further reacts through hydride abstraction from the Ir center with a kinetically accessible barrier (6.4 kcal mol^−1^ from Int 3, 28.4 kcal mol^−1^ from complex 4). This gives a Mo urea adduct which is not very stable (Int 4, 13.5 kcal mol^−1^ with respect to the entrance channel, see Scheme 5), with loss of urea from the coordination sphere inducing an extra energetic destabilization of 7.6 kcal mol^−1^. This results in the formation of the key reaction intermediate, Int 5, whose NBO and AIM analysis reveal an unsupported metal–metal quadruple bond (Table S3†), in line with the 18-electron rule. Note that the computed Mo–Ir bond length in Int 5 (2.210 Å, FSR = 0.86) is considerably shorter than that in 1 (2.2889(7) Å).

**Scheme 6:**
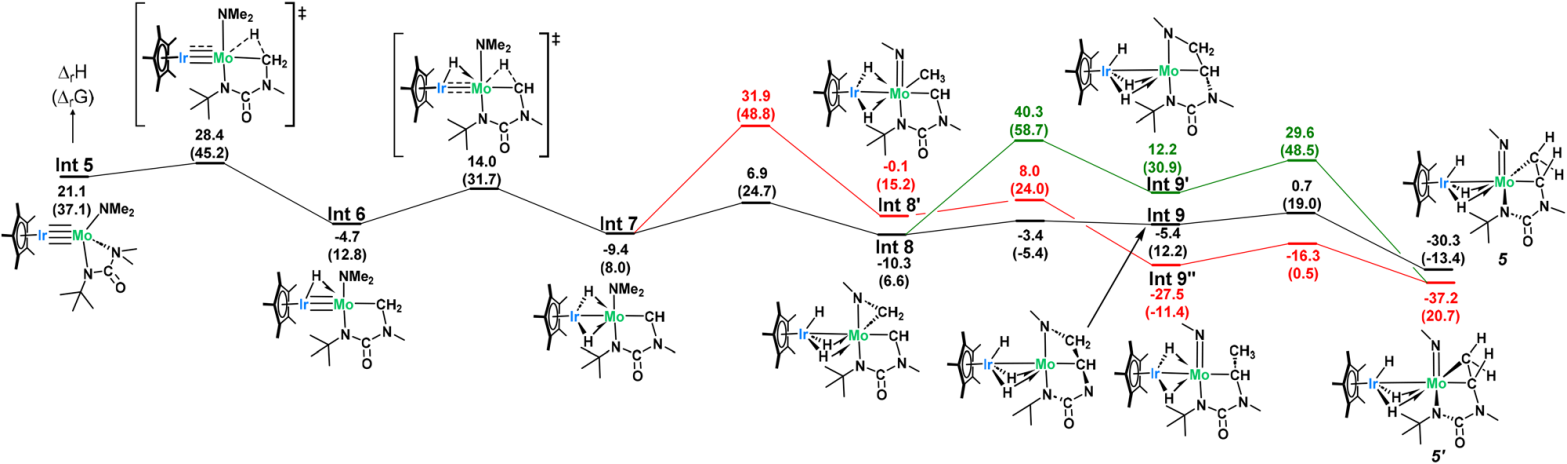
Calculated mechanism for the formation of 5.

Int 5 can then undergo two subsequent intramolecular hydrogen abstractions from a methyl group of the bound ureate ligand with relatively low barriers (7.3 and 18.7 kcal mol^−1^, Scheme 6) to yield a bridging dihydride iridium–molybdenum methylidene complex, which is the first intermediate more stable than complex 4 (Int 7, −9.4 kcal mol^−1^, Scheme 6). This finding accounts for the slow conversion of complex 4 to complex 5 found experimentally. Although stable, this amido–methylidene complex (Int 7) undergoes another hydrogen abstraction from the methyl group of the amido ligand this time with a low barrier (16.3 kcal mol^−1^) to yield a surprisingly stable trishydride–alkyl–methylidene complex (Int 8, −10.3 kcal mol^−1^). The changes in the metal–metal bond orders during the transformation from Int 5 to Int 8, in which the bridging hydrides are consecutively formed, is shown by the gradual decrease in the Ir–Mo WBI (2.41 in Int 5, 1.63 in Int 6, 1.14 in Int 7, 0.62 in Int 8; see Table S4† for more details). This trend highlights the pivotal role of the Ir–Mo bond in mediating this reactivity. The heterobimetallic core functions as a dynamic platform for electron storage and release *via* the Mo–Ir multiple bond, enabling multiple C–H activations and hydride transfers.

A C–C coupling reaction can then be done between the alkyl and the methylidene ligand with almost no barrier (4.9 kcal mol^−1^) followed by a C–N bond breaking (barrier of 6.1 kcal mol^−1^) to yield the very stable complex 5 (−30.3 kcal mol^−1^). Gratifyingly, NMR reaction monitoring shows, over time, the increase in concentration of 1 (as the pre-equilibrium shifts to the left), the formation of one equivalent of *N*,*N*-dimethyl-N′-*tert*-butyl-urea (as complex 4 converts to Int 5) and the formation of 5 (see Fig. S26†).

Alternative mechanisms were also explored, including the β-methyl migration (Scheme 6, red) to first form a Mo imide (Int 7′) before C–C coupling, but these were found to have prohibitively high activation barriers and so were excluded. Despite literature precedent showing isocyanate insertion into molybdenum imides, complex 5 does not undergo further reaction with *t*BuNCO, even under heating nor extended reaction times.^71^

**Fig. 4:**
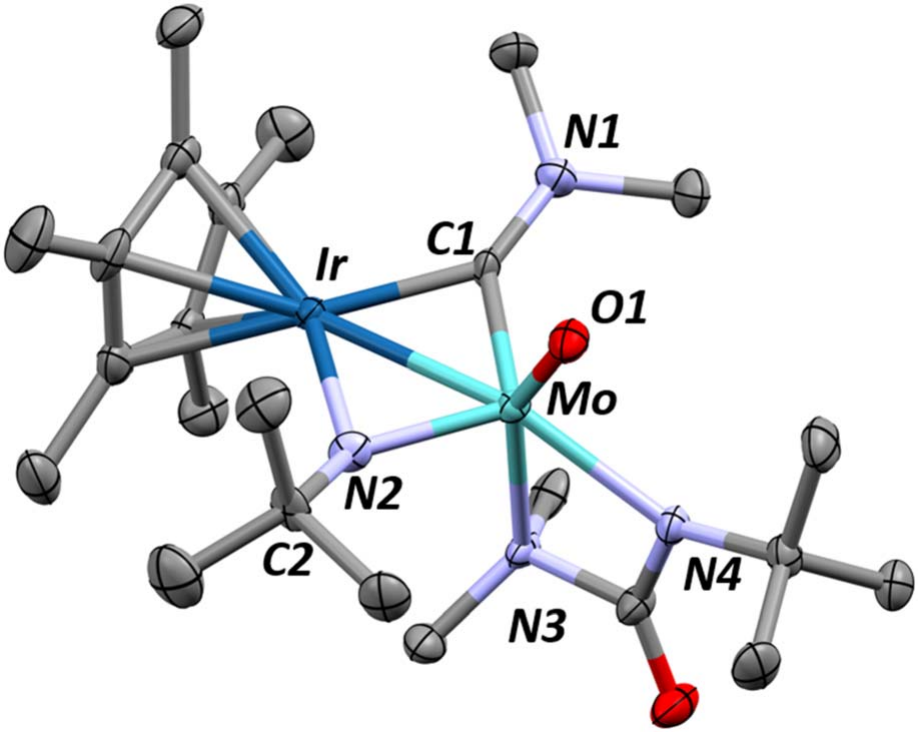
X-ray diffraction crystal structure of complex 6 with thermal ellipsoids represented at 50% probability and hydrogens removed for clarity. Relevant structural parameters are as follows: Mo–Ir = 2.6339(5) Å, FSR_MoIr_ = 1.03, Ir–C1 = 1.962(6), Mo–C1 = 1.965(6) Å, Ir–N2 = 2.040(6) Å, Mo–N2 = 1.860(6) Å, C1–N1 = 1.342(8) Å, N2–C2 = 1.464(9) Å, Mo–O1 = 1.738(5) Å, ∠Cp*_centroid_–Ir–Mo = 173.3(1)°.

Interestingly, the room temperature addition of five equivalents of *t*BuNCO to complex 1 generates a different complex, 6, in 82% isolated yield (Scheme 4). X-ray quality crystals of this product, grown from an approximately 9 : 1 toluene : pentane saturated solution, allow for an unambiguous assignment of ligand identity following this reaction (Fig. 4). The iridium–molybdenum distance is 2.6339(5) Å, corresponding to an FSR of 1.03. The elongated metal–metal distance is most likely due to the presence of two bridging ligands. Indeed, the two metals are bridged by an *N*,*N*-dimethylaminocarbyne ligand and an *N-t*Bu imido ligand, as a result of the insertion of the reactive *t*BuNCO into the last remaining dimethylamido ligand in Int 5 (see below). The C1–N1 bond length (1.342(8) Å) is typical of bridging aminocarbyne ligands (1.284–1.442 Å) with C1–M distances (1.962(6)–1.965(6) Å) also in line with previous reports on dinuclear systems (2.012 Å for a Mo_2_ μ-aminocarbyne).^72–75^

The methyl groups of the aminocarbyne moiety of complex 6 are equivalent in the ^1^H NMR spectrum, recorded in C_6_D_6_ solution at room temperature, which is suggestive of free rotation around the C1–N1 bond (Fig. S28†). The presence of the aminocarbyne moiety in 6 is confirmed by the characteristic ^13^C resonance found at +295 ppm (Fig. S29†). Note that whereas bridging aminocarbynes on homobimetallic molybdenum complexes typically appear further downfield (+305–390 ppm), the shift is highly metal dependant and homobimetallic iridium complexes show this resonance at +255 ppm.^74–77^ The carbyne ^13^C NMR chemical shift in 6 is thus an intermediate case between the Mo and Ir extremes. Neither the ^1^H NMR nor the DRIFT spectra (Fig. S28 and S38†) show signals typical of hydrides for this complex, in agreement with the proposed structure.

Complex 6 has a vibrant blue colour due to an intense absorption at 585 nm (*ε* = 2760 M^−1^ cm^−1^, Fig. S42†), consistent with previous reports of aminocarbyne LMCT bands.^78^ Altogether, the spectroscopic and the structural data point more to a μ-carbyne description (in contrast to a μ-iminium), where the bonding is more akin to a Schrock-type carbyne.^75,79^

Computational analysis shows that the HOMO is localized on the π bonding between the Mo–Ir core and empty orbitals from the aminocarbyne (Fig. S49†). The Mo–O bond length in the XRD structure, of 1.738(5) Å, is typical of Mo(vi) oxo complexes and precludes the possibility of a hydroxo ligand which tend to have much longer Mo–O bond distances (1.9–2.2 Å).^80–82^ Similarly, the bridging imido ligand does not have a proton on N2 as the geometry around nitrogen is more trigonal planar than tetrahedral (C2–N2–MoIr_plane_ = 153° *vs.* 109° for a tetrahedral geometry), agreeing with literature on bridging imido *vs.* amido ligands in binuclear systems.^83–86^ The Ir–N2 bond length of 2.040(6) Å also agrees well with the assignment of a bridging imido, with typical values of 1.88–2.10 Å, compared to bridging amido ligands which show longer bond distances (2.15–2.30 Å).^85,87–90^

As discussed above, the formation of Int 5 is accompanied by the release of one equivalent of *N*,*N*-dimethyl-*N*′-*tert*-butyl-urea, isolated from the reaction mixture by trituration with pentane (92% isolated yield from moles of 1). This co-product was unequivocally identified through both ^1^H NMR and X-ray crystallography (Fig. S30 and S43,† respectively), which shows a proton attached to the urea nitrogen, confirming the computed mechanism for the formation of Int 5 in Scheme 5.

**Scheme 7:**
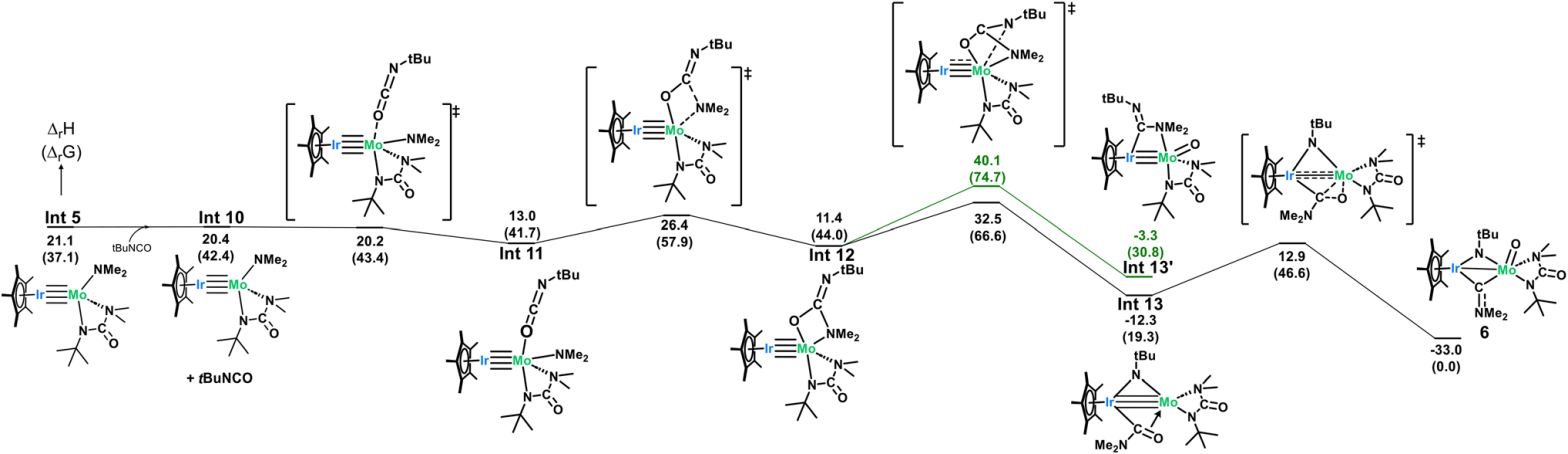
Computed mechanism for the formation of 6.

Due to the surprising ligand rearrangement occurring in this reaction, we turned to computational analysis to understand the mechanism at play (Scheme 7). Addition of another equivalent of *t*BuNCO to Int 5 results in a third C=N insertion into the Mo–N_amido_ bond. This insertion intermediate (Int 12) undergoes a C–N bond breaking reaction (barrier of 21.1 kcal mol^−1^) to yield a very stable bridging imido-carbamoyl complex (Int 13, −12.3 kcal mol^−1^). In this intermediate, the carbamoyl ligand is bound to the Ir center with an extra stabilization by the interaction between the η^2^ C=O double bond and the Mo center. This π-type interaction allows a C–O bond breaking reaction to occur at the Mo center with an accessible barrier of 25.2 kcal mol^−1^, yielding complex 6 which is thermodynamically stable (−33.0 kcal mol^−1^). This side-on carbamoyl ligand has been previously characterized in the context of decarbonylation chemistry but never seen to instead undergo oxygen atom transfer to make a Mo-oxo.^91–93^ This C–O bond cleavage is reminiscent of Chisholm's work, in which the C–O triple bond of carbon monoxide is cleaved across a ditungsten complex, leading to the formation of a tungsten-oxo moiety and insertion of the carbon atom into an alkylidyne ligand to generate a bridging alkynyl species.^94^ While aminocarbyne ligands have been well explored, this is the first reported synthesis from the oxygen atom transfer of a carbamoyl ligand. The calculated mechanism only requires one equivalent of *t*BuNCO from Int 5 (3 equiv. in total from moles of 1). However, a slightly larger excess (5 equiv. with respect to 1) was found to be required experimentally to avoid the competitive formation of complex 5.

## Conclusions

We report the synthesis and characterization of two novel iridium–molybdenum heterobimetallic complexes, Cp*Ir(H)Mo(NMe_2_)_3_ (1) and (Cp*IrH_2_)_2_Mo(NMe_2_)_2_ (2), prepared *via* protonolysis reactions between Cp*IrH_4_ and Mo(iv) amido complexes. To the best of our knowledge, these compounds represent the first crystallographically characterized examples of Mo–Ir multiple bonds. Both compounds react with CO_2_; however, only the trinuclear complex 2 yields a well-defined product. This transformation proceeds *via* a combination of H_2_ reductive elimination and CO_2_ insertion into the dimethylamido ligands, resulting in the formation of the carbamato complex 3. Complex 1 was found to react cleanly with *tert*-butylisocyanate (*t*BuNCO). In this transformation, insertion of the isocyanate into the amido ligand generates the intermediate 4, from which a urea ligand can be decoordinated, yielding a highly reactive, low-coordinate Mo–Ir quadruply bonded species, intermediate Int 5. This key species initiates a cascade of intriguing reactivity. In the absence of additional equivalents of isocyanate, this activation pathway leads to an unexpected triple C–H bond activation, through subsequent α-hydride abstractions promoted by the metal–metal bond, and a C–C coupling event. This ultimately yields a Mo(vi) metallacyclopropane complex, 5. However, this mechanism is interrupted if the key intermediate, Int 5, reacts with another equivalent of isocyanate. This results instead in C–N and C–O bonds cleavage and the formation of aminocarbyne and imido ligands bridging the Ir and Mo centers, along with a molybdenum(vi)-oxo moiety, yielding complex 6. The mechanisms of these complex transformations have been elucidated in details through DFT studies, which agree with the experimental observations and reveal the role of the bimetallic assembly in mediating hydride transfer and serving as a reservoir for electron storage and release *via* the Mo–Ir multiple bond. This unprecedented reactivity illustrates the promise of heterobimetallic compounds for enabling complex bond activations and reorganizations.

This work naturally leads to a discussion of alternative ligands as a strategy to tune the reactivity of the Mo–Ir multiple bond. Previous studies have demonstrated that modifying the ligand environment around Mo centers in multiply bonded dimolybdenum systems can profoundly influence their reactivity. Investigations are currently ongoing in our group to further research this topic.

## Author contributions

The manuscript was written through contributions of all authors. ZD performed the experimental work. LM and IDR directed the computational work. EJ contributed the X-ray structure determinations. CC and ZD conceptualized the research. CC found the funds and administrated the project. CC and CT supervised the work. ZD and CC wrote the initial version of the manuscript. All authors have given approval to the final version of the manuscript.

## Conflicts of interest

There are no conflicts to declare.

## Supplementary Material

SC-OLF-D5SC03465E-s001

SC-OLF-D5SC03465E-s002

## Data Availability

Crystallographic data for complexes 1–6 and *N*,*N*-dimethyl-*N*′-*tert*-butyl-urea has been deposited at the Cambridge Crystallographic Date Centre under accession codes 2410538–2410544 and can be obtained at https://www.ccdc.cam.ac.uk/data_request/cif.
