# Supplementary material for: Synergistic C–H bond activation across molybdenum–iridium multiply bonded complexes: a cascade of transformations
Source: Chem Sci. 2025 Jul 7;16(32):14564–77. doi: 10.1039/d5sc03465e (PMC12277905; doi:10.1039/d5sc03465e)
Supplement: SC-016-D5SC03465E-s001 [file SC-016-D5SC03465E-s001.pdf]

Supplementary Information (SI) for Chemical Science.
This journal is © The Royal Society of Chemistry 2025

Supporting Information

Synergistic C-H Bond Activation Across Molybdenum-Iridium Multiply Bonded Complexes: a Cascade of Transformations

Dr. Zachary Dubrawski,[a] Dr. Iker del Rosal,[b] Dr. Erwann Jeanneau,[c] Dr. Laurent Maron,[b] Dr. Chloé Thieuleux,[a] Dr. Clément Camp[a,*]

[a] Laboratory of Catalysis, Polymerization, Processes and Materials (CP2M UMR 5128), CNRS, Universite Claude Bernard Lyon 1, CPE-Lyon, Institut de Chimie de Lyon, 43 Bd du 11 Novembre 1918, F-69616 Villeurbanne, France
*clement.camp@univ-lyon1.fr

[b] LPCNO, Université de Toulouse, INSA Toulouse, 135 Avenue de Rangueil, Toulouse 31077, France

[c] Centre de Diffractométrie Henri Longchambon, Universite Claude Bernard Lyon 1, 5 Rue de la Doua, 69100 Villeurbanne, France

Table of Contents

General Considerations 2
Syntheses 3
Synthesis of complex $Mo(NMe_2)_4$ 3
Synthesis of complex $Cp^*Ir(H)Mo(NMe_2)_3$, **1** 3
Synthesis of complex $(NMe_2)_2Mo(IrH_2Cp^*)_2$, **2** 4
Reaction of **2** with CO_2 to generate **3** 4
Reaction of **1** with 1 equivalent *t*BuNCO and stopping at intermediate product **4** 5
Reaction of **1** with 1 equivalent *t*BuNCO and continuing to **5** 5
Reaction of **1** with excess *t*BuNCO to give **6** 6
NMR Spectra 7
Diffuse Reflectance Infrared Fourier Transform (DRIFT) Spectra 23
UV-Vis Spectra 26
X-ray Crystallography Data 28
Computational Data 33
References 124

General Considerations

Unless otherwise noted, all reactions were performed either using standard Schlenk line techniques or in an MBRAUN glovebox under an atmosphere of purified argon (<1 ppm of O_2/H_2O). Glassware and cannulas were stored in an oven at ~100 °C for at least 16 h prior to use. THF and *n*-pentane were purified by passage through a column of activated alumina, dried over Na/benzophenone, vacuum-transferred to a storage flask, and freeze–pump–thaw degassed prior to use. Deuterated solvents (toluene-d_8, THF-d_8, and C_6D_6) were dried over Na/benzophenone, vacuum-transferred to a storage flask, and freeze–pump–thaw degassed prior to use. The syntheses of Cp^*IrH_4 and $MoCl_4(OEt_2)_2$ were carried out following literature procedures.[1,2] The synthesis of $Mo(NMe_2)_4$ follows a modified literature procedure and is discussed below.[3] All other reagents were acquired from commercial sources and used as received.

IR Spectroscopy
The samples were prepared in a glovebox (either pure crystalline material, or diluted in dry KBr powder), sealed under argon in a Diffuse Reflectance Infrared Fourier Transform (DRIFT) cell fitted with KBr windows, and then analyzed using a Nicolet 670 FT-IR spectrometer.

Elemental Analyses
Elemental analyses were performed under an inert atmosphere at Mikroanalytisches Labor Pascher, Germany.

X-Ray Diffraction - Structural Determinations
Suitable crystals were coated in parabar oil, selected manually under a binocular microscope and mounted on a Rigaku-OD Synergy-S single-crystal diffractometer equipped with an Hypix-100 detector. Intensities were collected at 100K with molybdenum radiation (λ=0.71073 Å) for compounds **1** to **6** and with copper radiation (λ=1.54184 Å) for N,N-dimethyl-N'-*tert*butyl urea by means of the CrysalisPro software.[4] Reflection indexing, unit-cell parameters refinement, Lorentz-polarization correction, peak integration and background determination were carried out with the CrysalisPro software.[4] An analytical absorption correction was applied using the modeled faces of the crystal.[5] The resulting set of *hkl* was used for structure solution and refinement. The structures were solved with the ShelXT structure solution program using the intrinsic phasing solution method and by using Olex2 as the graphical interface.[6,7] The model was refined with version 2018/3 of ShelXL using least-squares minimization.[6]

CCDC 2410538-2410544 contain the supplementary crystallographic data for this paper. These data can be obtained free of charge from The Cambridge Crystallographic Date Centre via www.ccdc.cam.ac.uk/data_request/cif.

NMR Spectroscopy
Solution NMR spectra were recorded on Bruker AV-300 and AV-500 spectrometers. ^1H and ^{13}C chemical shifts were measured relative to residual solvent peaks, which were assigned relative to an external TMS standard set at 0.00 ppm. ^1H and ^{13}C NMR assignments were confirmed by ^1H–^1H COSY, ^1H–^{13}C HSQC, and HMBC experiments. NMR data recorded as follows: chemical shift (δ) [multiplicity, coupling constant(s) *J* (Hz), relative integral], where multiplicity is defined: s = singlet, d = doublet, t = triplet, q = quartet, m = multiplet or combinations thereof, and prefixed br = broad.

UV-Visible Spectroscopy
Samples were dissolved in n-pentane or THF and transferred to a quartz cuvette with a J. Young valve inside an argon filled glove box. Solvent backgrounds were subtracted manually from an appropriate solvent blank experiment. Absorption spectra were recorded on a Perkin-Elmer Lambda 1050 UV/Vis/NIR spectrophotometer.

Syntheses

Synthesis of complex $Mo(NMe_2)_4$

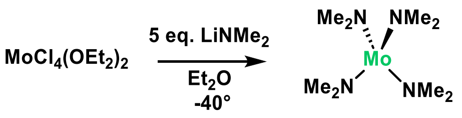

In an argon-filled glovebox, a 500 mL Schlenk flask was charged with 3.005 g $MoCl_4(OEt_2)_2$ (7.78 mmol, 1 eq), 250 mL diethylether and a very large, Teflon-coated stir bar. The flask was fitted with a solid addition funnel filled with 1.986 g $LiNMe_2$ ($3.89e^{-2}$ mol, 5 eq). The bright orange suspension of $MoCl_4(OEt_2)_2$ was cooled to -40 °C using an EtOH/liquid nitrogen bath and the $LiNMe_2$ powder was added in one portion with vigourous stirring. The suspension was slowly warmed to room temperature and allowed to stir at room temperature for 4h after which the solvent was removed under reduced pressure. The flask was then fitted with a distillation arm and heated to 60 °C with the rapid evolution of a bright purple material collected in the receiving flask. Yield = 0.712 g, 33%. NMR spectroscopic analysis confirms the formation of $Mo(NMe_2)_4$ and no further characterization was performed.

^{1}H NMR (300 MHz, C_6D_6, 298K) δ 3.27 (s, 24H), matches literature expectation.[3]

Synthesis of complex $Cp^*Ir(H)Mo(NMe_2)_3$, **1**

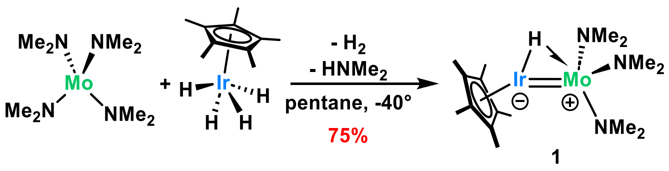

In an argon-filled glovebox, a 50 mL Schlenk flask was charged with 0.150 g of complex $Mo(NMe_2)_4$ (5.56 mmol, 1 eq) and 50 mL pentane was added to yield a deep purple solution. This was brought out to a Schlenk line and cooled to approx. -50°C. A colorless solution of 0.184 g Cp^*IrH_4 (5.56 mmol, 1 eq) in 25 mL pentane was added to the rapidly stirring $Mo(NMe_2)_4$ solution *via* cannula over 2 mins. The solution was slowly warmed to room temperature and left to react for 6 hours. The volatiles were removed *in vacuo* to yield a sticky brown solid, which was extracted and recrystallized from pentane at -35°C to yield brown needles along the edge of the liquid phase in the scintillation vial. The dilute conditions and cold temperature were found to be necessary to avoid over substitution and formation of an excessive amount of complex **2**. Despite these conditions, some complex **2** is always present and complex **1** was only obtained pure through multiple, successive recrystallizations from pentane and manual separation of the crystals, reflected in the lower yield. Isolated mass = 0.231 g, Yield = 75%. Caution: the released dimethylamine is potently odorous.

Characterization data:

^{1}H NMR (500 MHz, C_6D_6, 298 K) δ 3.36 (s, 18H, N**Me₂**), 2.28 (s, 15H, Cp*), -6.41, (s, 1H, Ir-H)

^{13}C NMR (125 MHz, C_6D_6, 298K) δ 88.17 (Cp*-**C**), 49.80 (N-**Me**), 11.39 (Cp*-**Me**)

UV-Vis (**Figure S 39**): 495 nm (5500 $M^{-1}cm^{-1}$)

DRIFTS (**Figure S 33**): 2990-2772 cm^{-1} (C-H, s), 1990 (M-H, m)

EA ($C_{16}H_{34}N_3MoIr$) Expected: C 34.52, H 6.16, N 7.55. Found: C 34.97, H 6.35, N 7.43

*Despite several attempts, the carbon analysis for compound **1** is 0.45% higher than the calculated value, slightly exceeding the generally accepted margin of 0.4%.*

Synthesis of complex $(NMe_2)_2Mo(IrH_2Cp^*)_2$, **2**

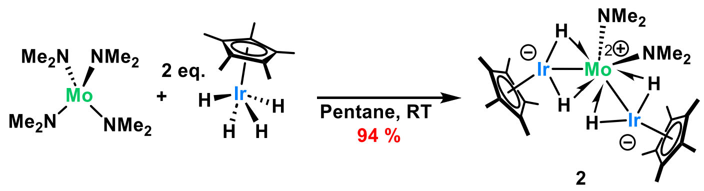

In an argon-filled glovebox, a 250 mL Schlenk flask was charged with 0.200 g of $Mo(NMe_2)_4$ ($7.35e^{-4}$ mol, 1 eq) and 50 mL pentane was added to it, yielding a deep purple solution. A solution of 0.487 g of complex Cp^*IrH_4 ($1.47e^{-3}$ mol, 2 eq) in 25 mL pentane was added at room temperature to the rapidly stirring Mo solution *via* cannula over 2 minutes, with instant reaction to yield a wine-red solution. The reaction was left to react at room temperature over 18 hours before the volatiles were removed under vacuum to yield a deep red/black solid. This solid was recrystallized from a minimum volume of pentane at -35°C to give X-ray quality, black block shaped crystals of complex **2**. Isolated mass = 0.588 g, Yield = 94%. Caution: the released dimethylamine is potently odorous.

Characterization data:

^{1}H NMR (500 MHz, C_6D_6, 298 K) δ 3.49 (s, 12H, N**Me₂**), 2.34 (s, 30H, Cp*), -10.23 (s, 4H, Ir-H).

^{13}C NMR (125 MHz, C_6D_6, 298K) δ 90.52 (Cp*-**C**), 55.56 (N-**Me**), 11.80 (Cp*-**Me**)

UV-Vis (**Figure S 40**): 280 nm (11063 $M^{-1}cm^{-1}$), 472 nm (2258 $M^{-1}cm^{-1}$)

DRIFTS (**Figure S 34**): 3002 -2729 cm^{-1} (C-H, s), 2028 cm^{-1} (M-H, m), 1928 cm^{-1} (M-H, m)

EA ($C_{24}H_{46}N_2MoIr_2$) Expected: C 34.19, H 5.50, N 3.32. Found: C 34.12, H 5.54, N 3.25

Reaction of **2** with CO_2 to generate **3**

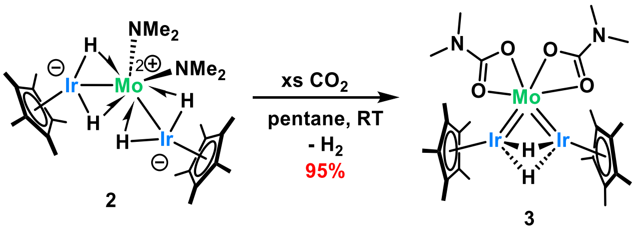

In an argon-filled glovebox, a 100 mL Teflon screw-capped Schlenk flask was charged with a solution of 0.051 g ($6.05e^{-5}$ mol, 1 eq) of complex **2** dissolved in 20 mL of pentane. The wine-red solution was degassed twice *via* a freeze-pump-thaw procedure and the atmosphere of the flask was replaced with 0.984 bar high-purity CO_2 (N48, $3.2e^{-3}$ mol, 52 eq). The solution was left to stir at room temperature over 18h with a slow colour change to dark green. The solvent was removed under reduced pressure and the solid was recrystallized from pentane at -40°C. Isolated mass = 0.053 g, yield = 95%

Characterization data:

^{1}H NMR (500 MHz, C_6D_6, 298K) δ 2.37 (s, 12H, N**Me₂**), 2.32 (s, 30H, Cp*), -6.54 (s; 2H, Ir-**H**).

^{13}C NMR (125 MHz, C_6D_6, 298K) δ 168.78 (Me_2N**C**OO), 88.03 (Cp*-**C**), 33.82 (**Me₂**NCOO), 10.99 (Cp*-**Me**).

UV-Vis (**Figure S 41**): 290 nm (9420 $M^{-1}cm^{-1}$), 384 nm (2917 $M^{-1}cm^{-1}$), 595 nm (441 $M^{-1}cm^{-1}$)

DRIFTS (**Figure S 35**): 3084 – 2758 cm^{-1} (C-H, s), 2131 (M-H, m), 2019-1849 (M-H, s) 1571 cm^{-1} (C=O, s)

EA ($C_{26}H_{44}N_2MoIr_2$) Expected: C 33.61, H 4.77, N 3.02. Found: C 34.01, H 4.92, N 3.08

Reaction of **1** with 1 equivalent *t*BuNCO and stopping at intermediate product **4**

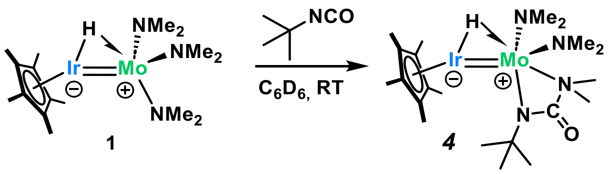

In an argon-filled glovebox, a 20 mL scintillation vial was charged with 20 mg of complex **1** (3.59e-5 mol, 1 eq) and 2 mL of pentane. 43 µL of a 0.82 mol/L *t*BuNCO ($3.59e^{-5}$ mol, 1 eq) stock solution in pentane was added with a slight darkening of the solution colour. The solution was left to stir for 10 minutes at ambient temperature before the volatiles were removed under reduced pressure to yield a reddish brown powder. This powder was recrystallized from a saturated pentane solution (2.5 mL) at -40 °C to yield 15.3 mg of X-ray quality, dark orange, block shaped crystals in (65% isolated yield, 95% from NMR quantification).

Characterization data:

^{1}H NMR (300 MHz, C_6D_6, 298K) δ 3.49 (br s, 10H, Mo-N**Me$_2$**), 2.86(br s, 6H, (**H$_3$**C)$_2$N$_{urea}$)), 2.02 (s, 15H, Cp*), 1.62 (s, 9 H, (**H$_3$**C)$_3$C-N$_{urea}$) -9.85 (s, 1H, Ir-**H**).

^{1}H NMR (500 MHz, toluene-*d*8, 248K) δ 4.80 (br s, 3H, N**Me**), 3.61 (br s, 3H, N**Me**), 3.35 (br s, 3H, N**Me**), 3.22(br s, 3H, N**Me**), 2.00 (s, 15H, Cp*), 1.60 (s, 9 H, (**H$_3$**C)$_3$C-N$_{urea}$), -9.69 (s, 1H, Ir-**H**).

^{13}C NMR (125 MHz, toluene-*d*8, 248K) δ 164.29 (N$_2$**C**=O), 88.93 (Cp*-**C**), 54.90((H$_3$C)$_3$**C**-N$_{urea}$), 52.65 (N$_{urea}$-**C**H$_3$), 50.91 (N$_{amido}$(**C**H$_3$)$_2$), 4, 30.24 (H$_3$**C**)$_3$C-N$_{urea}$, 10.85 (Cp*-**Me**).

DRIFTS: 3015-2809 cm^{-1} (C-H), 2155 cm^{-1} (M-H)

Owing to the instability of this reaction intermediate, satisfactory elemental analysis results could not be obtained.

Reaction of **1** with 1 equivalent *t*BuNCO and continuing to **5**

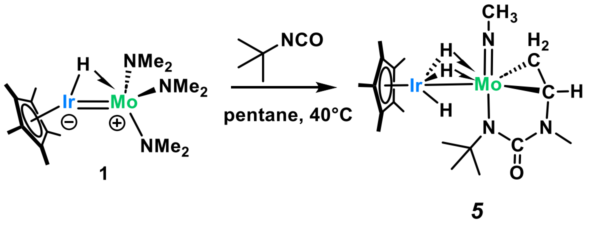

In an argon-filled glovebox, a 20 mL Schlenk flask was charged with 25 mg of complex **1** ($4.49e^{-5}$ mol, 1 eq) and 5 mL of pentane. To this solution was added 52.2 µL of a 0.86 mol/L *t*BuNCO solution ($4.49e^{-5}$ mol, 1 eq) in pentane. The solution was stirred at room temperature for 1 hour before removing from the glovebox and heating at 40°C for 1h. The solvent was removed under reduced pressure and the brownish solid was recrystallized from pentane cooled to -40 °C to give orange rhombohedral crystals of X-ray quality. Isolated product mass = 17.8 mg (yield = 59%). Complex **1,** which is generated along **5** in this reaction, can be removed as dark brown needles along the edge of the liquid phase.

Characterization data:

^{1}H NMR (500 MHz, C_6D_6, 298 K) δ 4.20 (dd, *J* = 9.8, 6.7 Hz, 1H, H$_2$C=C**H**-N), 3.03 (s, 3 H, **H$_3$**C-N=Mo), 2.98 (s, 3 H, **H$_3$**C-N$_{urea}$), 2.69 (t, *J* = 6.2 Hz, 1H **H$_2$**C=CH-N), 2.17 (dd, *J* = 9.7, 5.6 Hz, 1H, **H$_2$**C=CH-N), 1.98 (s, 9 H, (**H$_3$**C)$_3$C-N$_{urea}$), 1.82 (s, 15 H, Cp*), -12.60 (s, 3 H, Ir-**H**).

^{13}C NMR (125 MHz, C_6D_6, 298K) δ 169.77 (N$_2$**C**=O), 92.14 (Cp*-**C**), 73.67 (H$_2$C=**C**H-N), 57.10 ((H$_3$C)$_3$C-N$_{urea}$), 53.19 (N$_{urea}$-**C**H$_3$), 47.22 (H$_2$C=**C**H-N), 37.82 (Mo=N-**C**H$_3$), 31.40 (H$_3$**C**)$_3$C-N$_{urea}$), 10.83 (Cp*-**Me**).

DRIFTS (**Figure S 37**): 3025-2802 cm^{-1} (s, C-H), 1974-1782 cm^{-1} (M-H), 1634 cm^{-1} (C=O)

Reaction of **1** with excess *t*BuNCO to give **6**

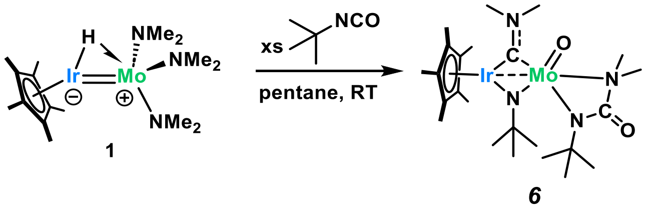

In an argon-filled glovebox, a 20 mL scintillation vial was charged with 25 mg of complex **2** (4.49e^{-5} mol, 1 eq) in 4 mL of pentane. To this solution was added 26µL of neat *t*BuNCO (2.24e-4 mol, 5 eq) in one portion. This solution was stirred at room temperature overnight to yield a dark blue/black suspension. The solvent and unreacted *t*BuNCO were removed under reduced pressure and the dark solid was triturated with 2 mL pentane to remove unreacted **1** and N,N-dimethyl-N'-*tert*butylurea. The resulting deep blue powder was recrystallized from 9:1 toluene:pentane to yield 26 mg of X-ray quality crystals of **6** in 82% yield.

Characterization data:

^{1}H NMR (500 MHz, C_6D_6, 298K) δ 3.44 (s, 6H, (**H**$_3$C)$_2$N=C-M), 2.15 (s, 9H, (**H**$_3$C)$_3$C-N$_{imide}$), 1.82 (s, 9H, (**H**$_3$C)$_3$C-N$_{urea}$), 1.68 (s, 21H, Cp* and (**H**$_3$C)$_2$N$_{urea}$).

^{13}C NMR (125 MHz, C_6D_6, 298K) δ 295.43 (**C**=NMe$_2$), 166.07 (N$_2$**C**=O), 93.45(Cp*-**C**), 71.46 (N$_{imide}$-**C**(CH$_3$)$_3$), 55.16 (N$_{urea}$-**C**(CH$_3$)$_3$), 48.33 (C=N**Me$_2$**), 45.46(**Me$_2$**N-C=O), 32.70 (N$_{imide}$-C(**C**H$_3$)$_3$), 30.66(N$_{urea}$-C(**C**H$_3$)$_3$), 11.32 (Cp*-**Me**).

UV-Vis (**Figure S 42**): 302 nm (7285 M^{-1}cm^{-1}), 387 nm (3582 M^{-1}cm^{-1}), 585 nm (2760 M^{-1}cm^{-1})

DRIFTS (**Figure S 38**): 3020-2808 cm^{-1} (s, C-H), 1669 cm^{-1} (C=O)

EA ($C_{24}H_{45}N_4O_2MoIr$) Expected: C 40.61, H 6.39, N 7.89. Found: C 40.74, H 6.45, N 7.82.

NMR Spectra

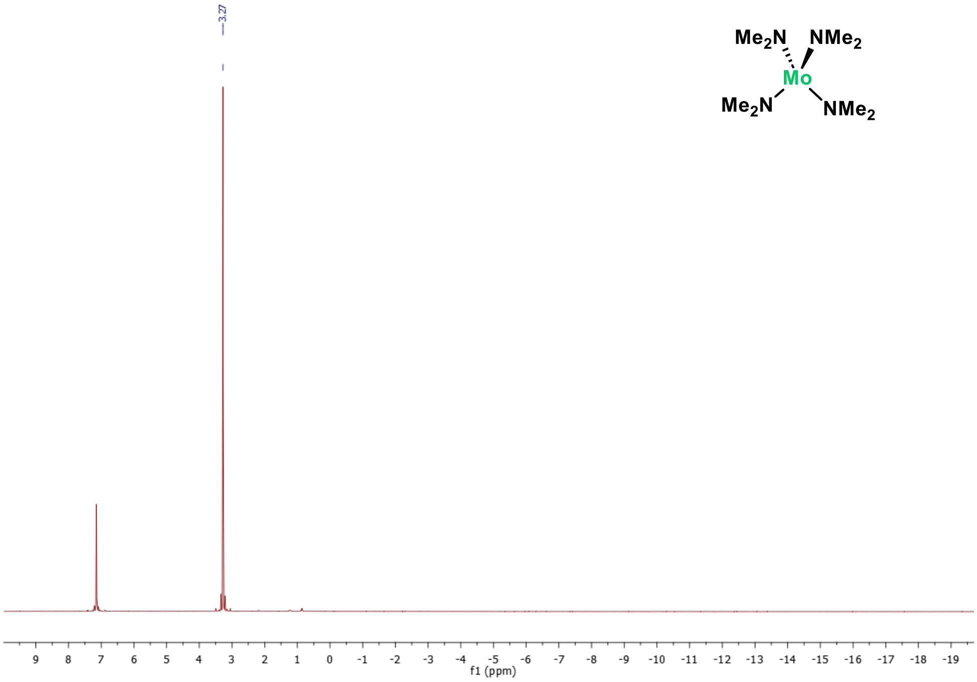

Figure S 1 : ^{1}H NMR spectrum of $Mo(NMe_2)_4$ in C_6D_6 solution, recorded at 300 MHz, 298K. No paramagnetic impurities were observed when expanding the collection window to ±150 ppm.

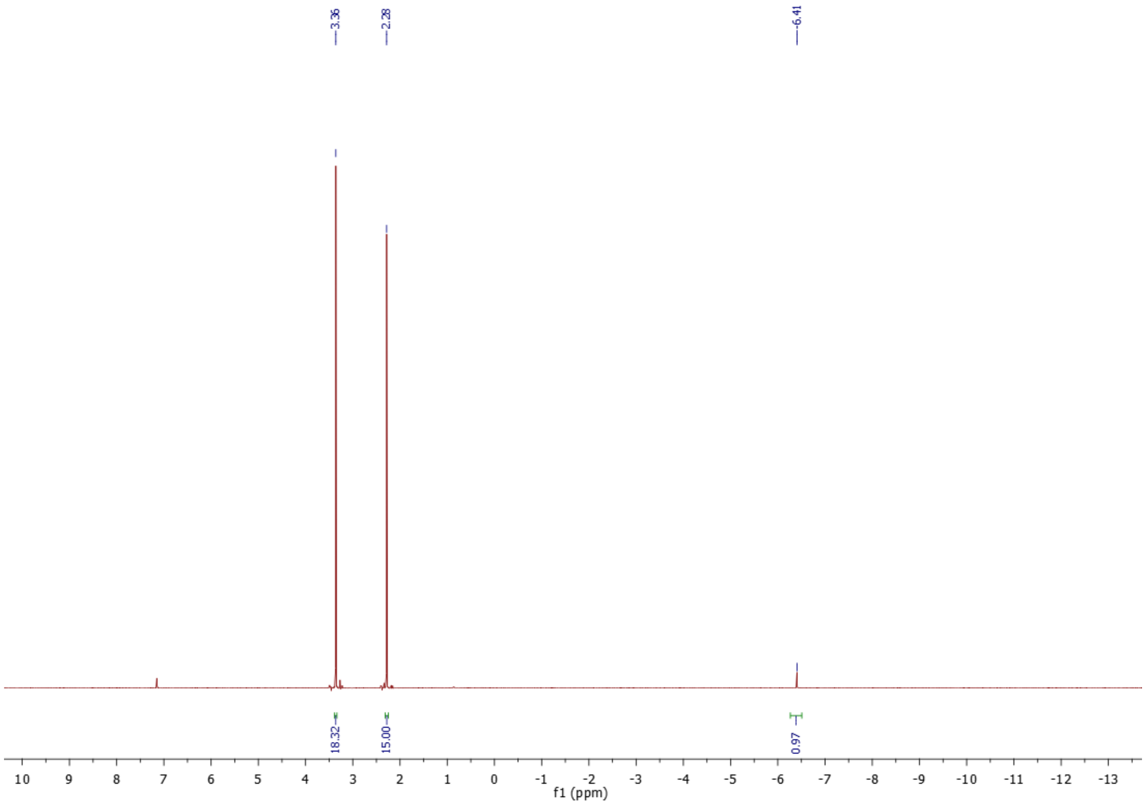

Figure S 2: ^{1}H NMR spectrum of compound 1 in C_6D_6 solution, recorded at 500 MHz, 298K.

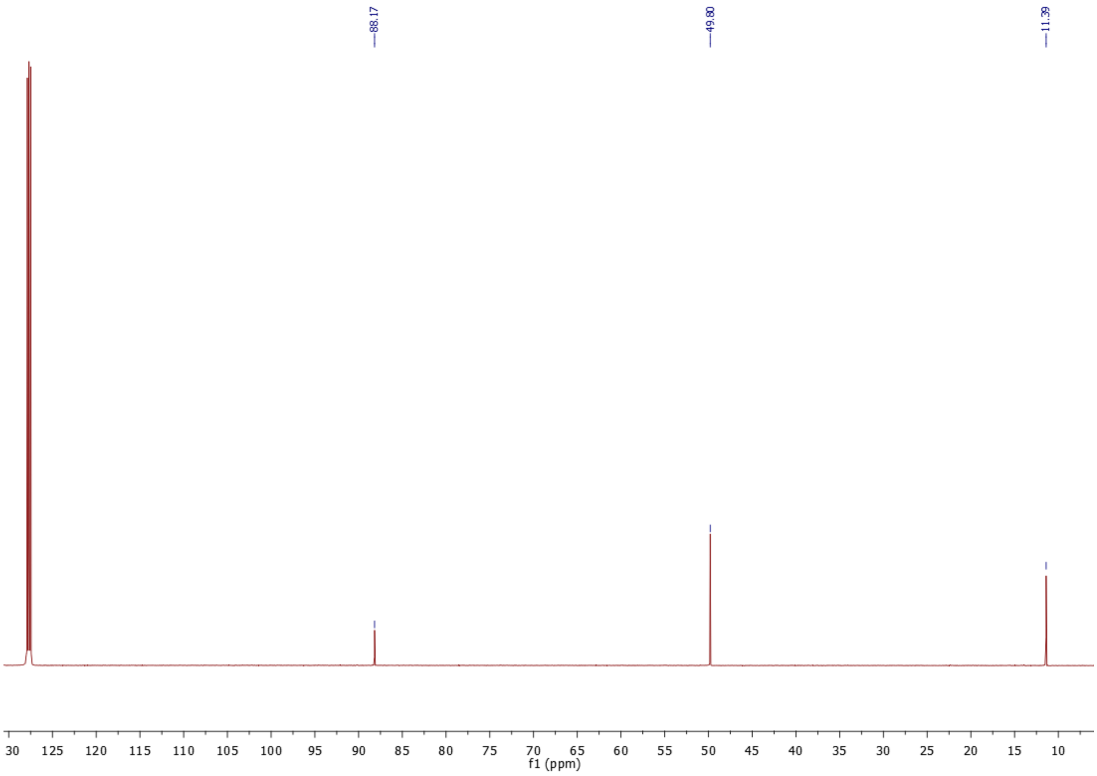

Figure S 3 : ^{13}C NMR spectrum of compound 1 in C_6D_6 solution, recorded at 125 MHz, 298K.

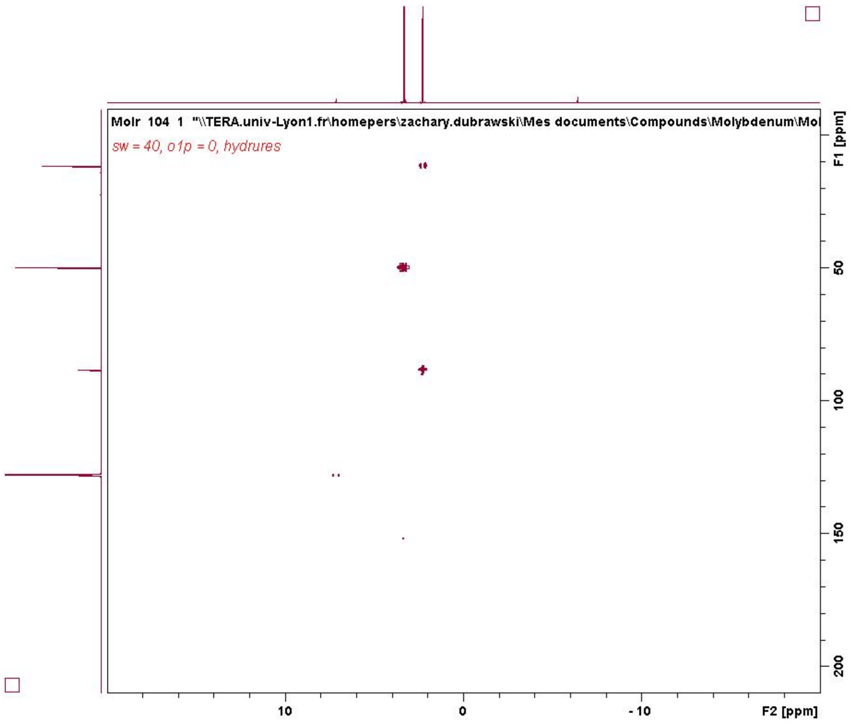

Figure S 4 : ^{1}H-^{13}C HMBC 2D NMR spectrum of compound 1 in C_6D_6 solution, recorded at 500 MHz, 298K. Spectra presented along axes are external projections (for clarity) of ^{1}H and ^{13}C spectra recorded on the same sample.

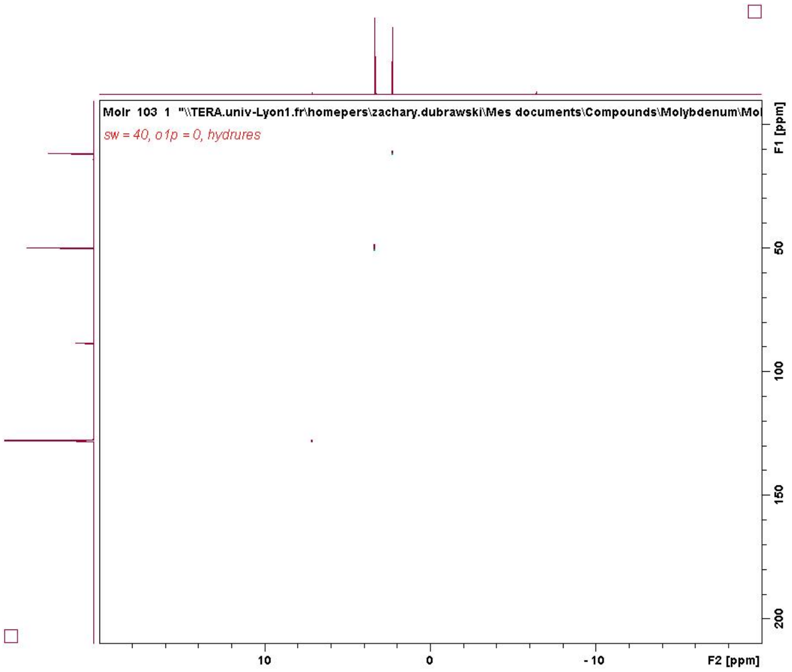

Figure S 5 : ^{1}H-^{13}C HSQC 2D NMR spectrum of compound 1 in C_6D_6 solution, recorded at 500 MHz, 298K. Spectra presented along axes are external projections (for clarity) of ^{1}H and ^{13}C spectra recorded on the same sample.

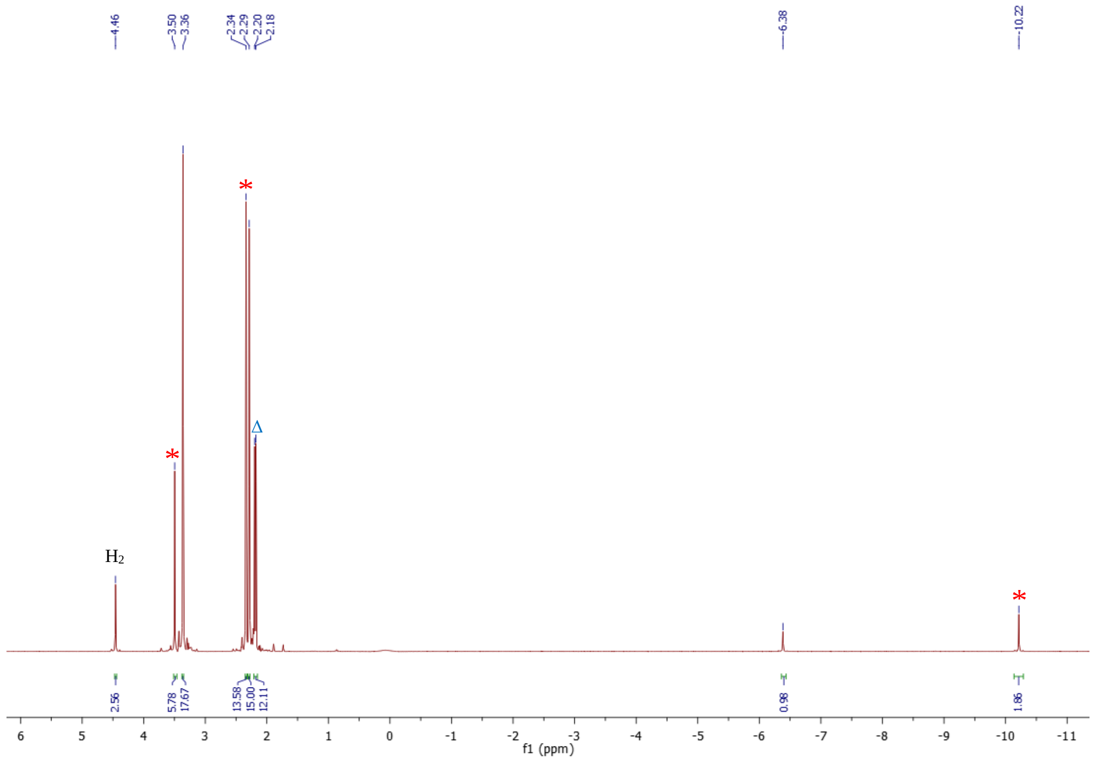

Figure S 6 : ^{1}H NMR spectrum of a synthesis of 1 in a J-Young NMR tube fully filled with C_6D_6 (no headspace). This allows for the detection and titration of dihydrogen produced in this reaction. Due to the speed of the reaction and a lack of good mixing, a full J-Young is challenging to load and mix in a timely manner, significant over substitution of 1 can be observed and a large concentration of 2 (marked with a red asterisk,*) can be observed in this reaction in about a 1:0.5 ratio. 2 equivalents of dimethylamine can also be observed (d, 2.19 ppm, marked with a blue triangle). Dihydrogen (4.46 ppm, chemical shift consistent with literature [8]) can be observed in about 2 equivalent ratio with the product, complex 1.

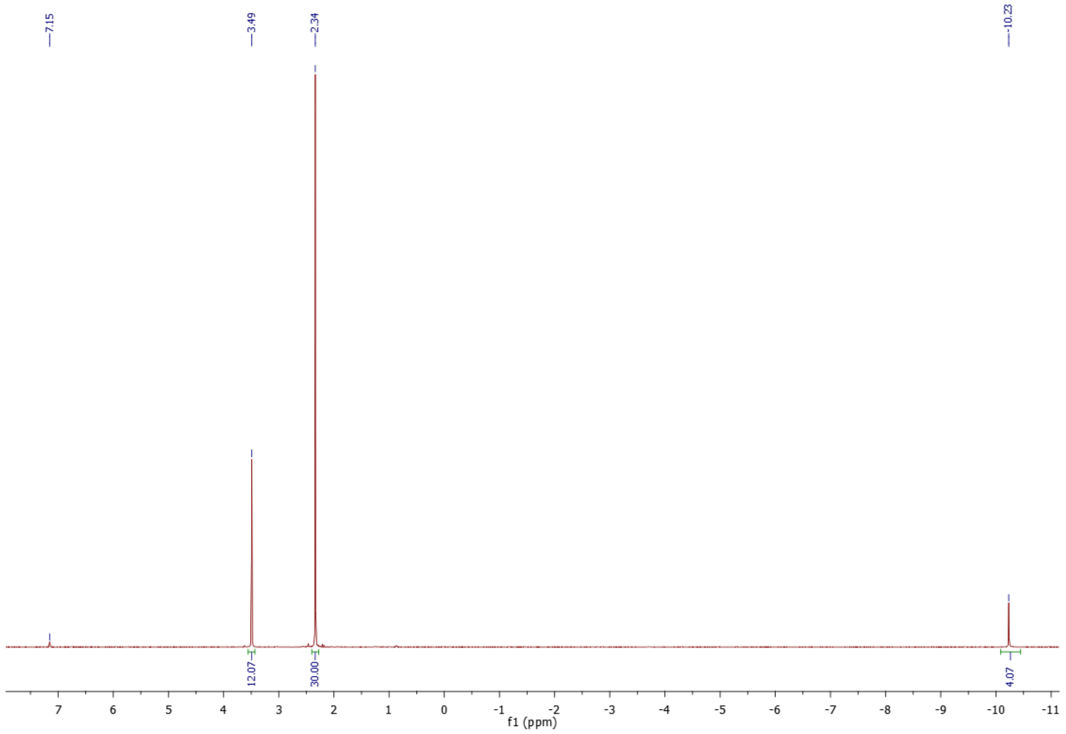

Figure S 7 : ^{1}H NMR spectrum of compound 2 in C_6D_6 solution, recorded at 500 MHz, 298K.

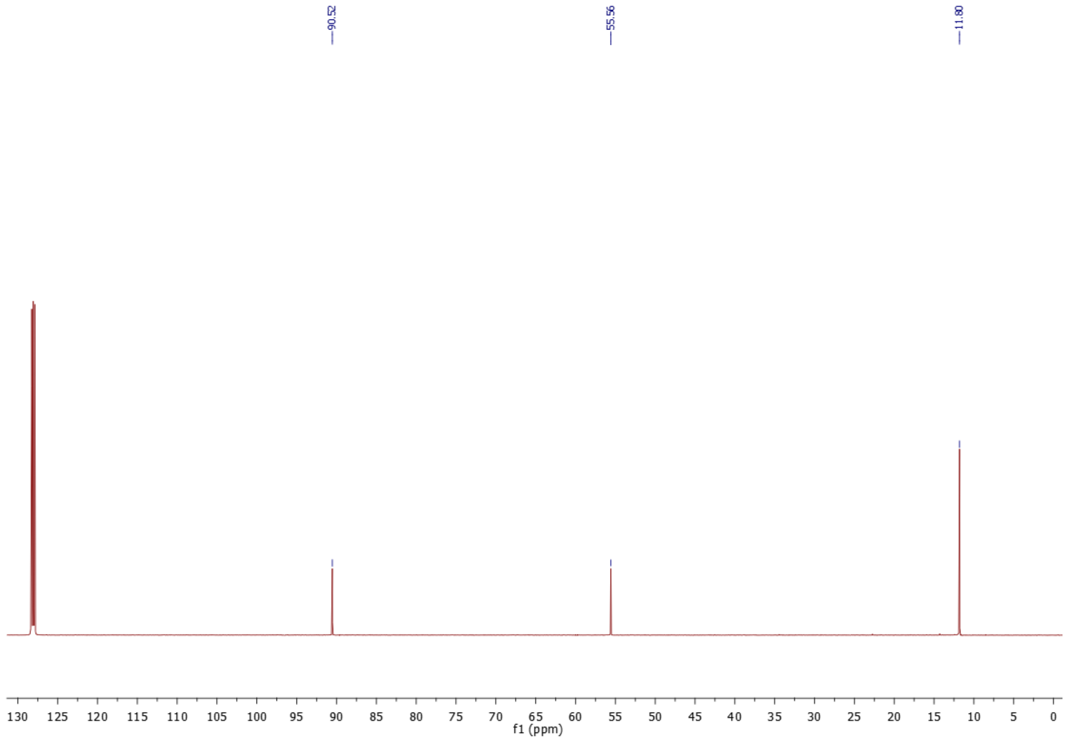

Figure S 8 : ^{13}C NMR spectrum of compound 2 in C_6D_6 solution, recorded at 125 MHz, 298K.

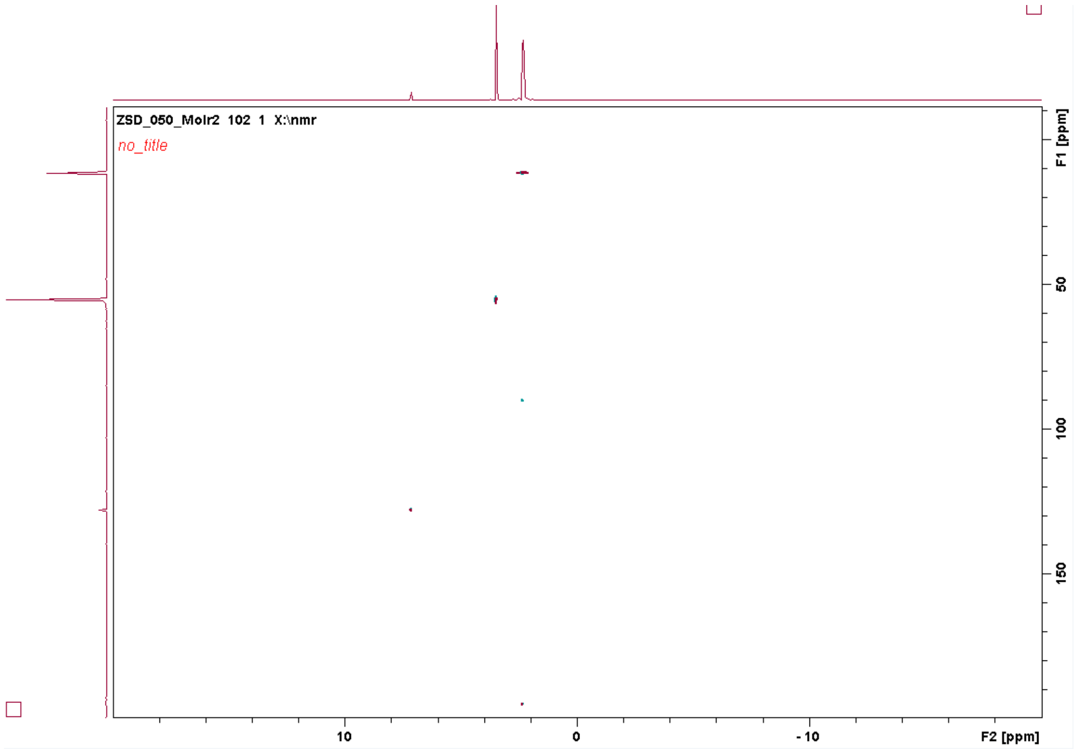

Figure S 9 : ^1H-^{13}C HSQC 2D NMR spectrum of compound 2 in C_6D_6 solution, recorded at 500 MHz, 298K. Spectra presented along axes are external projections (for clarity) of ^1H and ^{13}C spectra recorded on the same sample.

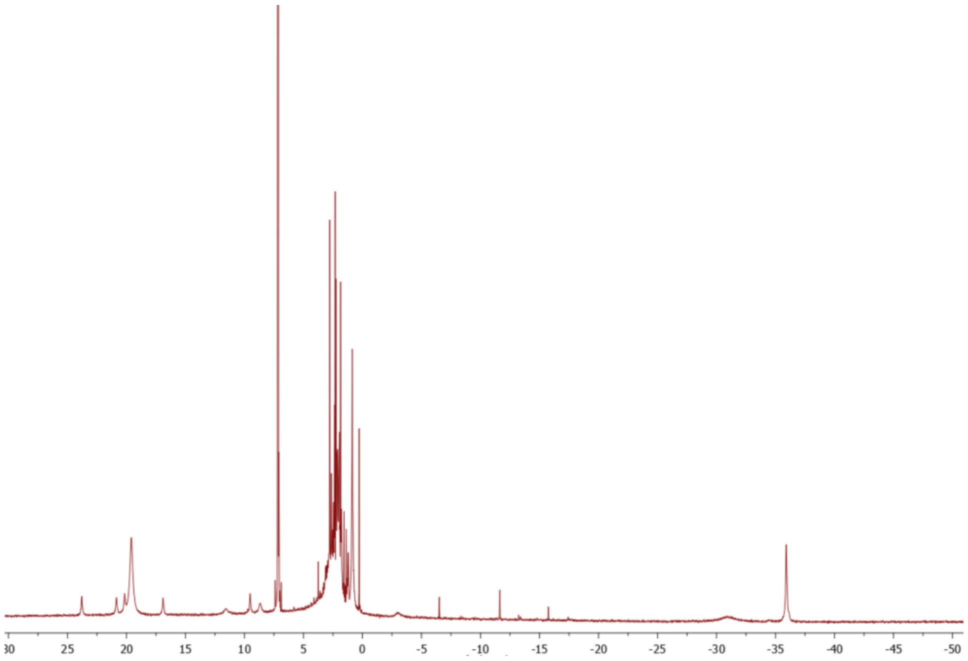

Figure S 10 : The reaction of 1 with CO_2 yields many products, as shown by ^1H NMR analysis of the crude reaction mixture. This particular reaction was exposed to an excess of CO_2 in C_6D_6 (300 MHz, 298K) at room temperature but similar results were obtained from reactions performed with stoichiometric amounts of CO_2, reduced temperature or with the exclusion of light.

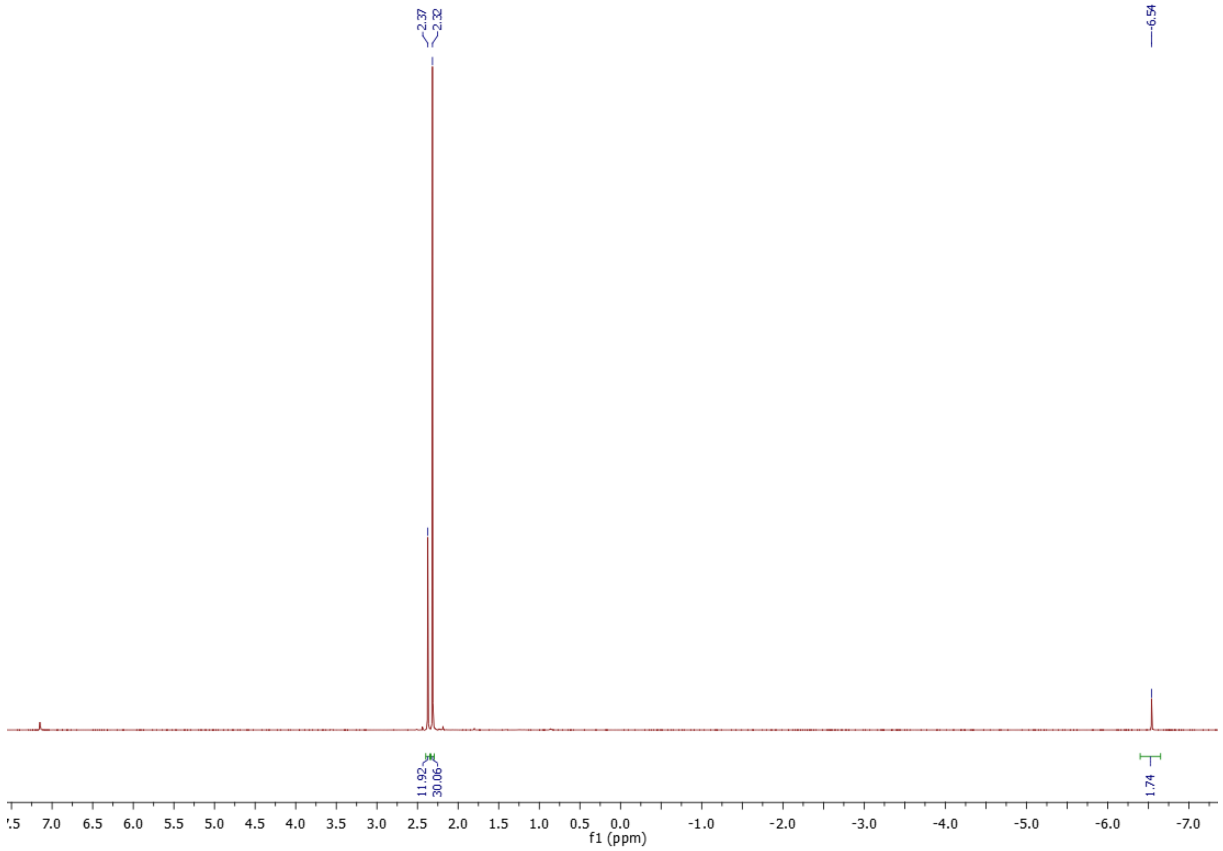

Figure S 11 : ^{1}H NMR spectrum of compound 3 in C_6D_6 solution, recorded at 500 MHz, 298K.

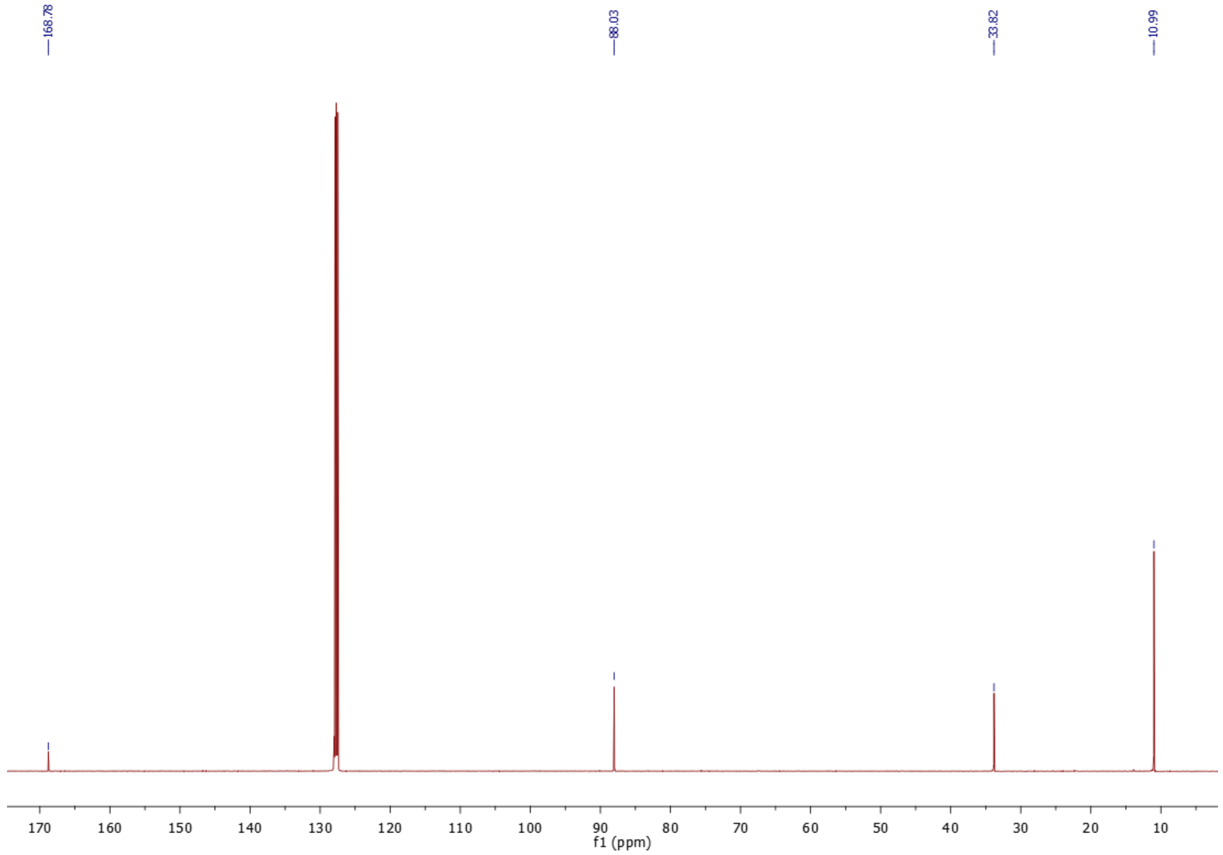

Figure S 12: ^{13}C NMR spectrum of compound 3 in C_6D_6 solution, recorded at 125 MHz, 298K.

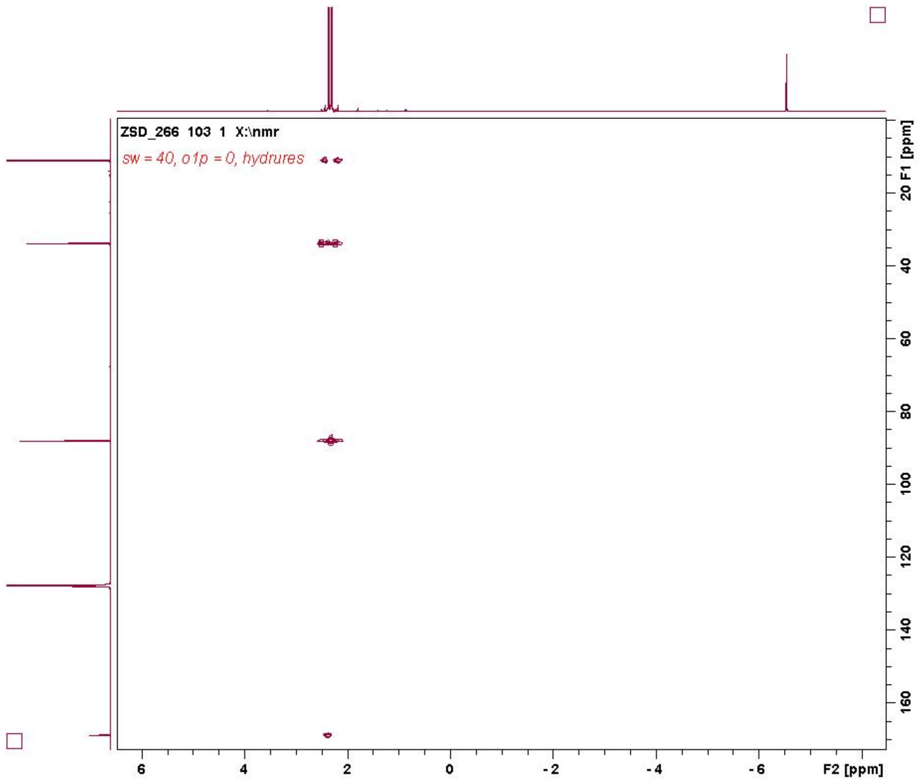

Figure S 13 ^{1}H-^{13}C HMBC 2D NMR spectrum of compound 3 in C_6D_6 solution, recorded at 500 MHz, 298K. Spectra presented along axes are external projections (for clarity) of ^{1}H and ^{13}C spectra recorded on the same sample.

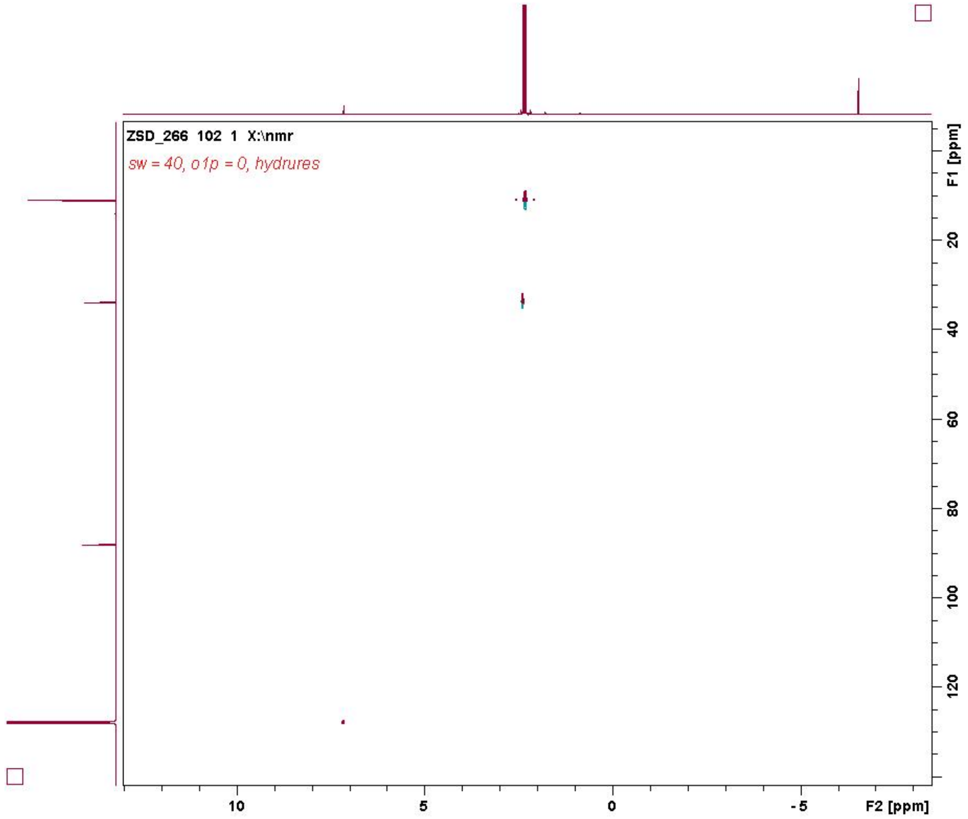

Figure S 14 ^{1}H-^{13}C HSQC 2D NMR spectrum of compound 3 in C_6D_6 solution, recorded at 500 MHz, 298K. Spectra presented along axes are external projections (for clarity) of ^{1}H and ^{13}C spectra recorded on the same sample.

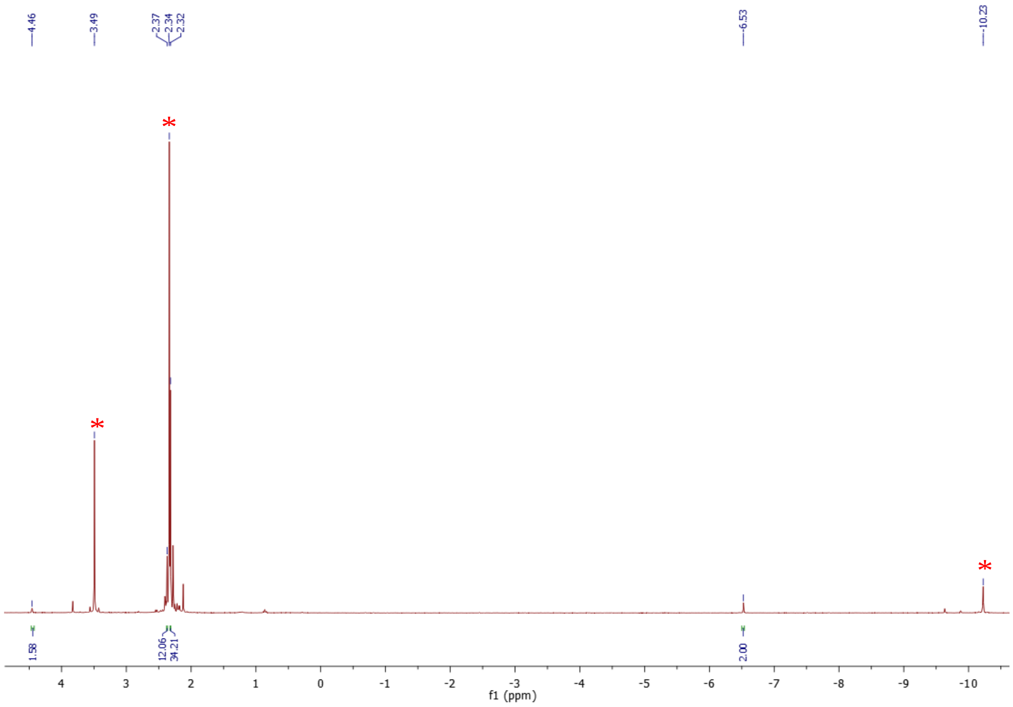

Figure S 15 : ^{1}H NMR spectrum of the crude reaction mixture from the synthesis of 3 in a J-Young NMR tube filled with C_6D_6, recorded at 300 MHz, 298K. This allows for the detection and titration of dihydrogen produced in this reaction. The starting material, complex 2, is marked with red asterisks (*). The necessarily small headspace in the J-Young tube only permits a substoichiometric volume of CO_2 and therefore the reaction cannot go to completion. Dihydrogen (4.46 ppm, chemical shift consistent with literature [8]) can be observed in about 1 equivalent ratio with the product, complex 3.

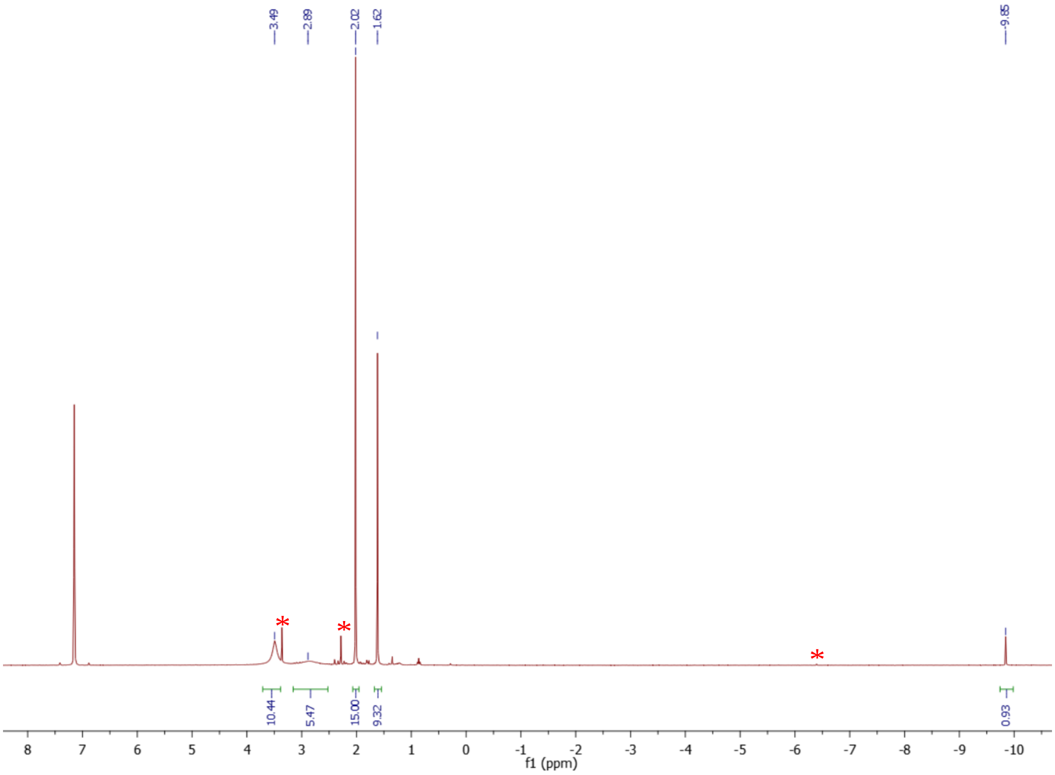

Figure S 16 : ^{1}H NMR spectrum of complex 4 in C_6D_6 solution, recorded at 298K, 300 MHz. The broad signals at 3.49 and 2.89 ppm correspond to the Mo-NMe_2 and the urea NMe_2 moieties, respectively. Some unreacted 1 can be observed and is marked with a red asterisk.

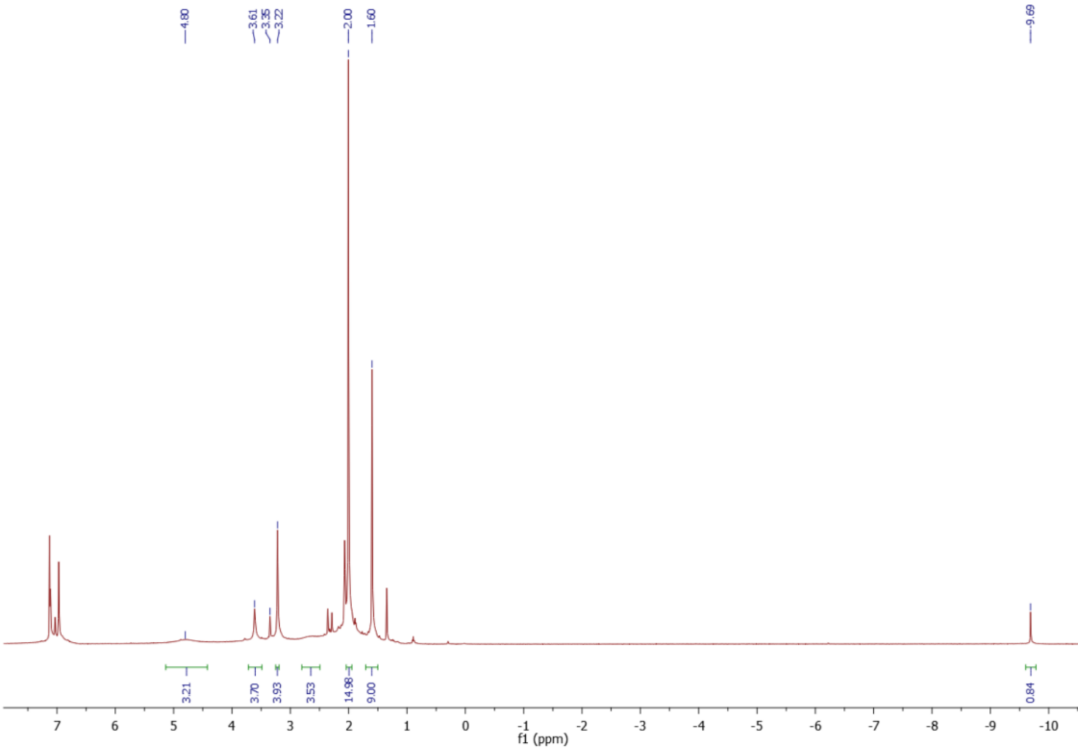

Figure S 17 : ^{1}H NMR spectrum of complex 4 in toluene-*d*8 solution, recorded at 248K, 500 MHz. One can observe some deconvolution of the signals for the NMe_2 signals at this reduced temperature however, the assignment is unclear.

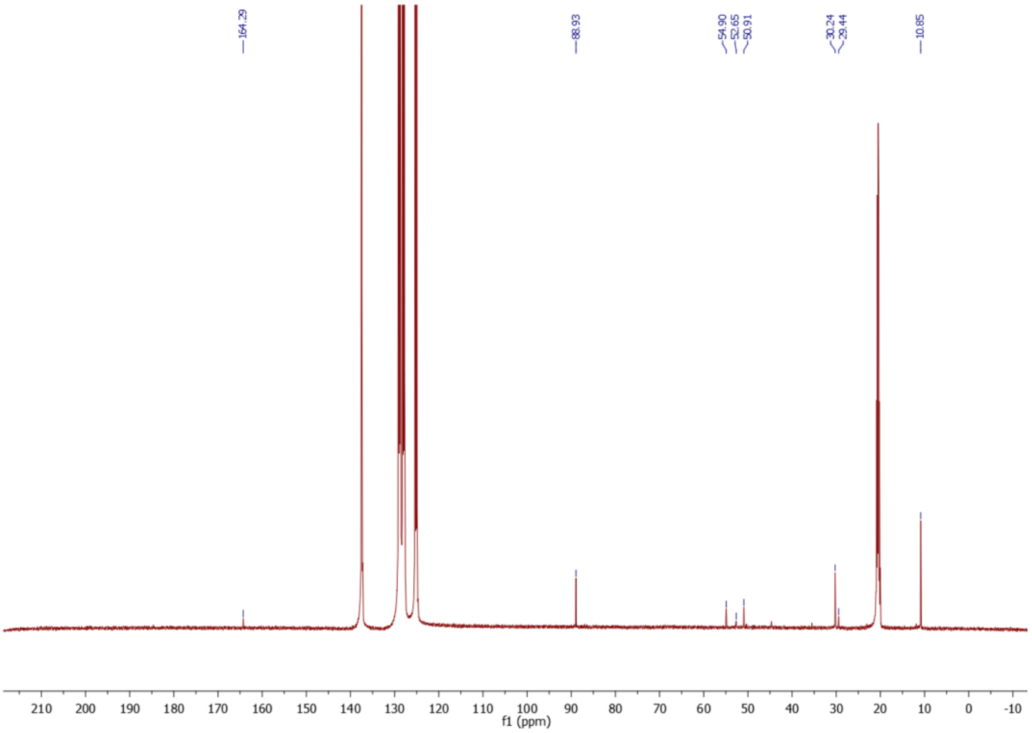

Figure S 18 : ^{13}C NMR spectrum of complex 4 in toluene-*d*8 solution, recorded at 248K to avoid the conversion of the complex to 5 at room temperature.

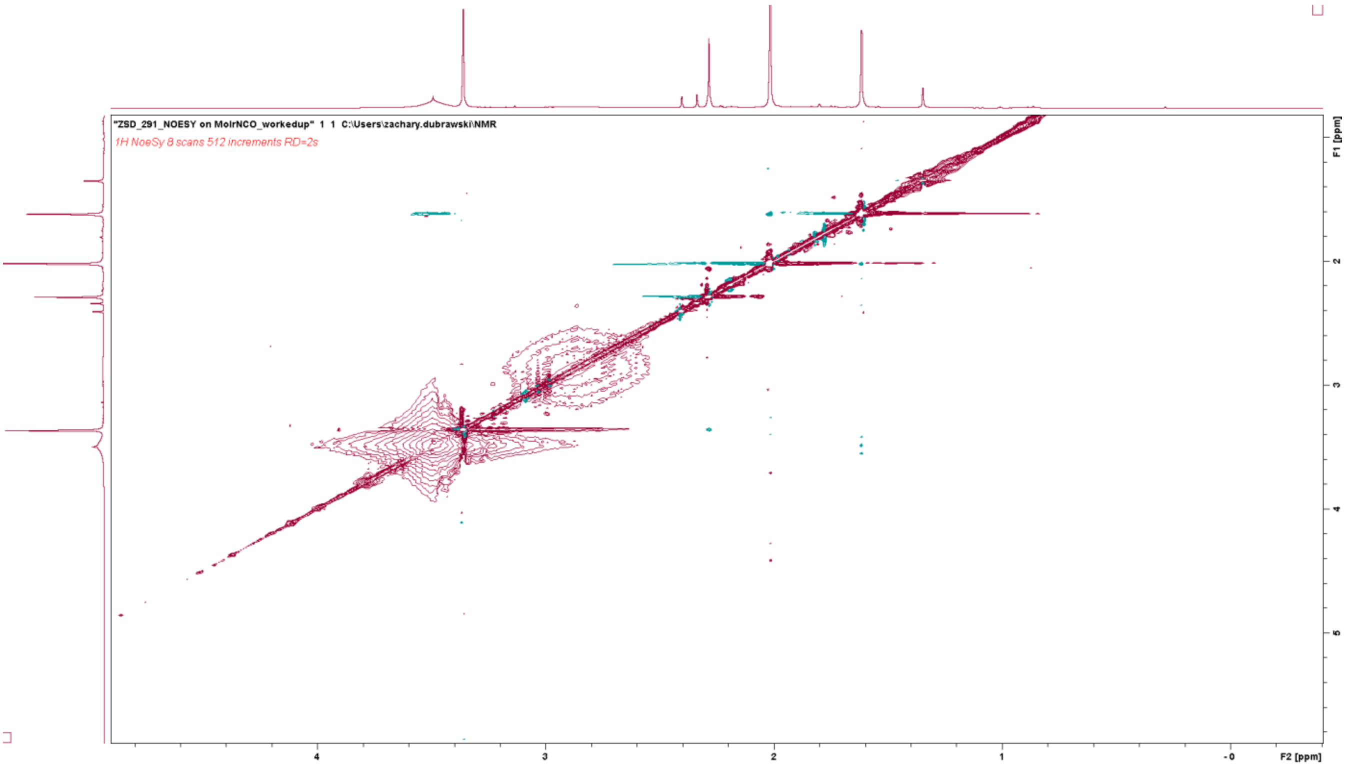

Figure S 19 : The NOESY spectrum (EXSY) of compound 4 in C_6D_6 with a very large broad signal between the NMe_2 groups of compound 4 (3.48ppm) and those of compound 1 (3.36 ppm). The cross peak(s) are in-phase with the diagonal, consistent with chemical exchange. They are however, extremely broad and could be a chemical exchange process with the other NMe_2 groups on complex 4 at (2.86 ppm).

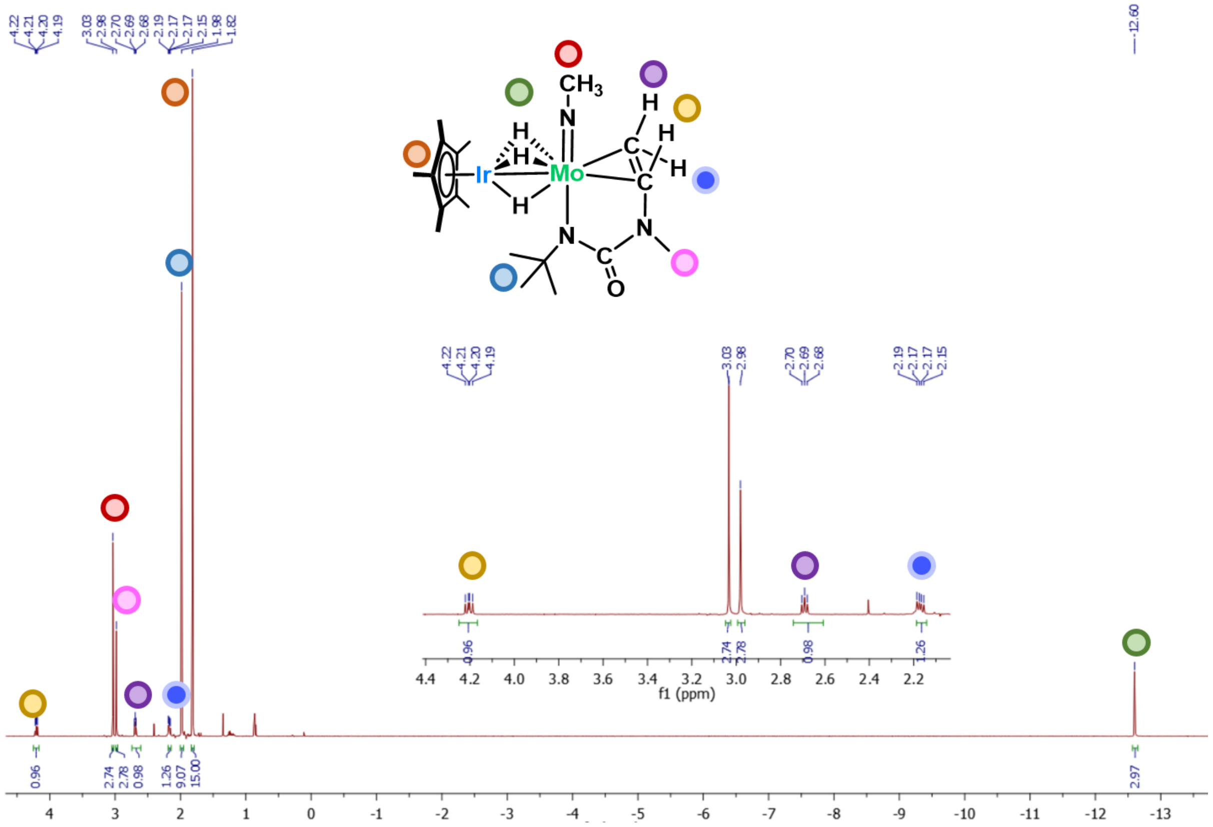

Figure S 20 : ^1H NMR spectrum of compound 5 in C_6D_6 solution, recorded at 500 MHz, 298K. The inset is expanding the region between 4.5 – 2.0 ppm, highlighting the diasterotopic protons on the metallacyclopropane moiety.

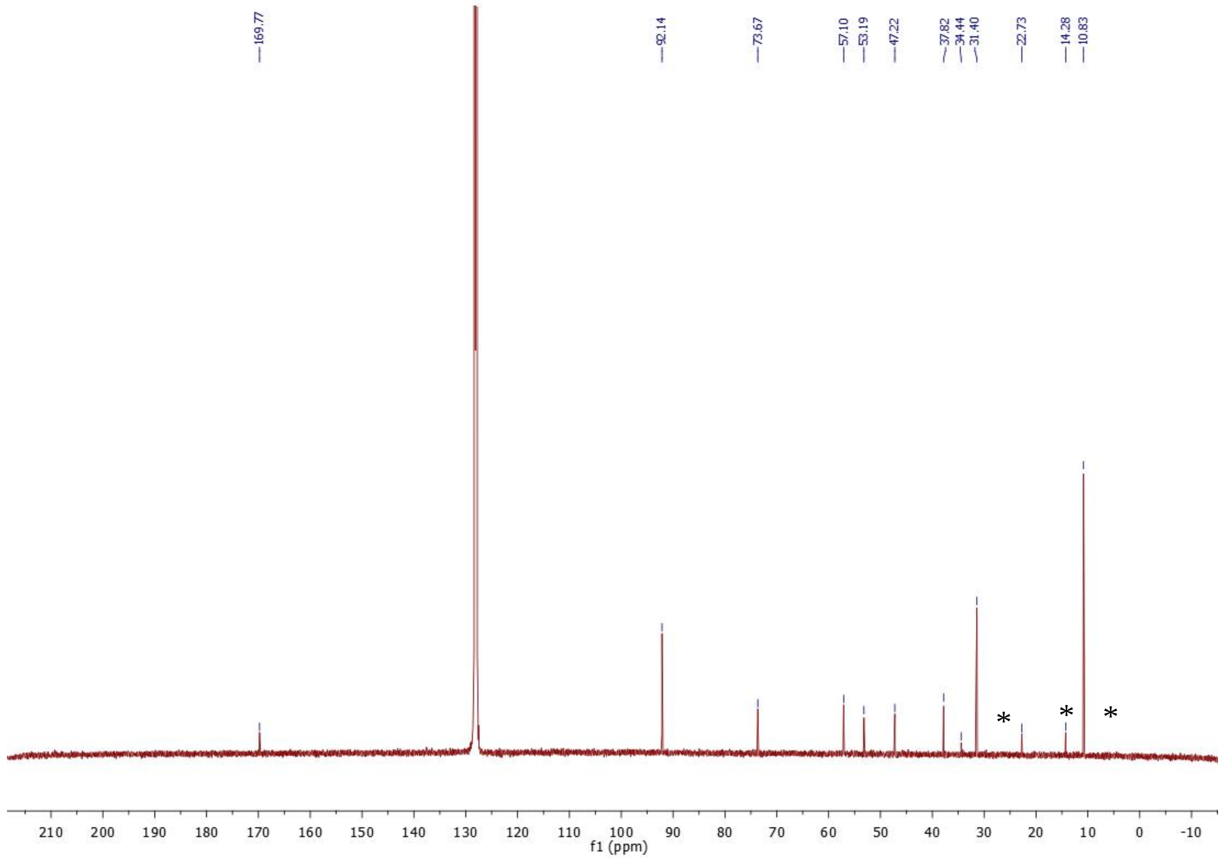

Figure S 21 : ^{13}C NMR spectrum of compound 5 in C_6D_6 solution, recorded at 125 MHz, 298K. Pentane impurity peaks marked with an asterisk (*).

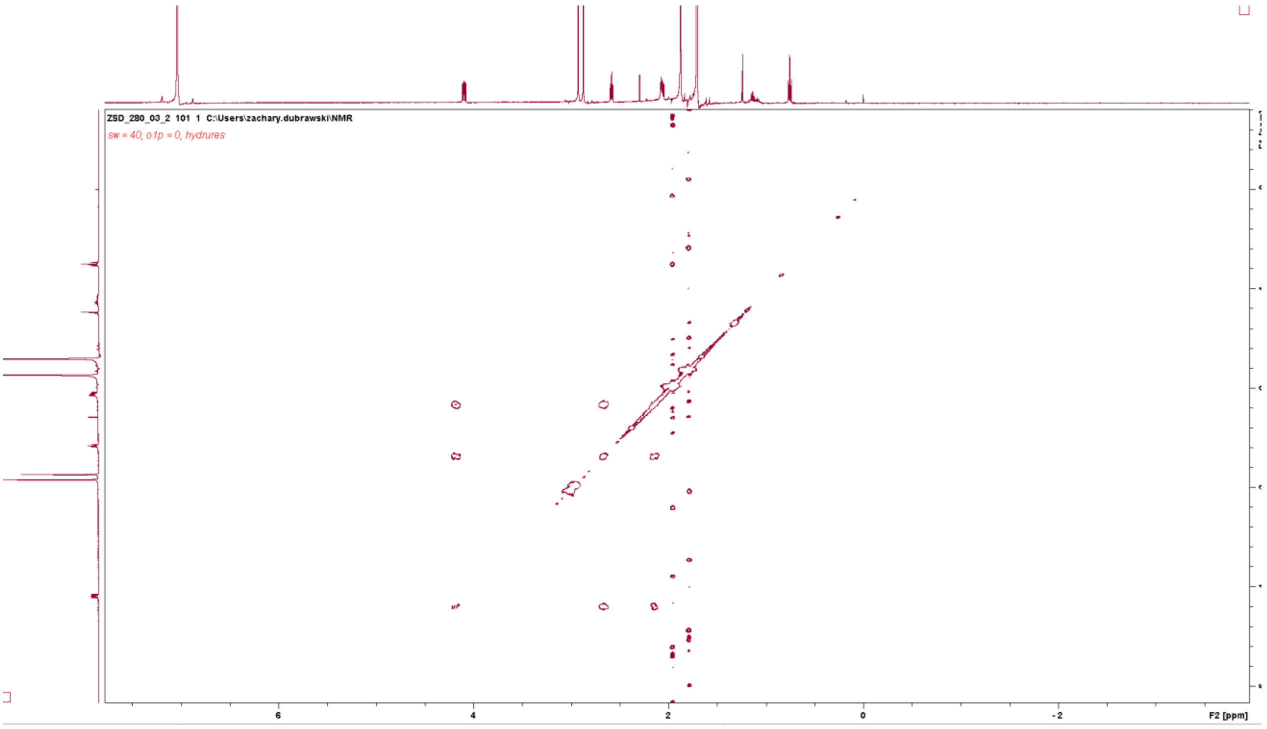

Figure S 22 : ^{1}H-^{1}H COSY 2D NMR spectrum of compound 5 in C_6D_6 solution, recorded at 500 MHz, 298K. Spectra presented along axes are external projections (for clarity) of ^{1}H spectra recorded on the same sample.

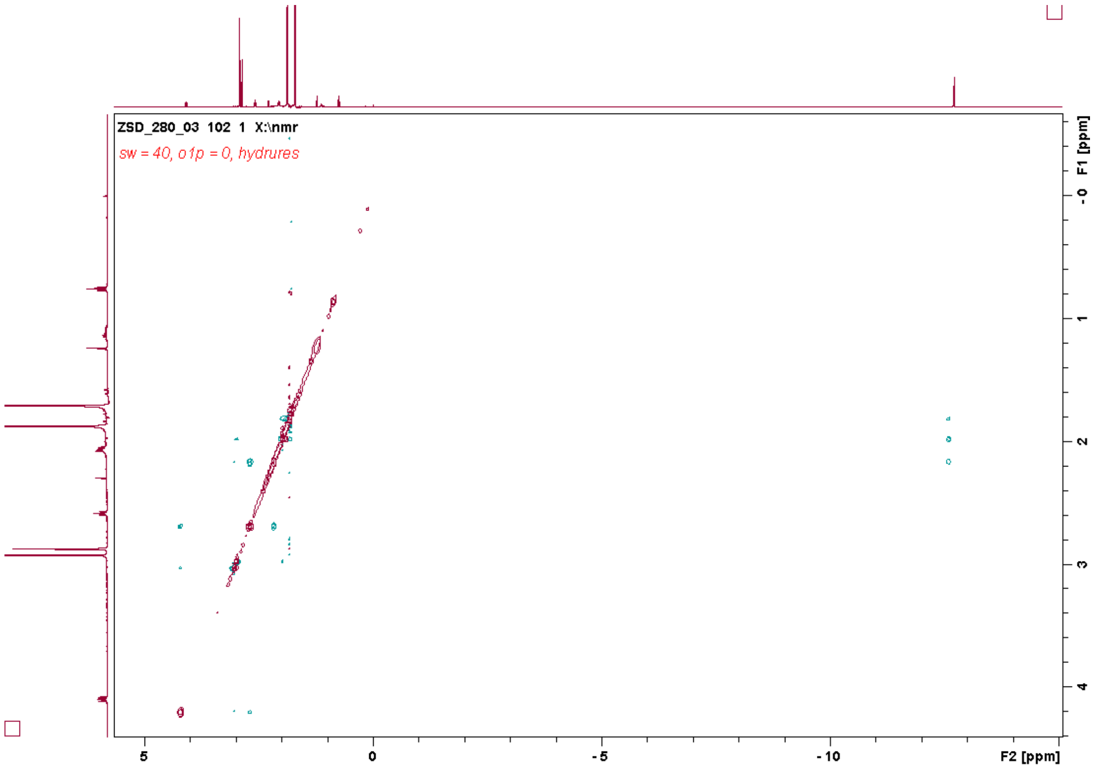

Figure S 23 : 2D ¹H-¹H NOESY NMR spectrum of compound 5 in C_6D_6 solution, recorded at 500 MHz, 298K. Spectra presented along axes are external projections (for clarity) of ¹H spectra recorded on the same sample.

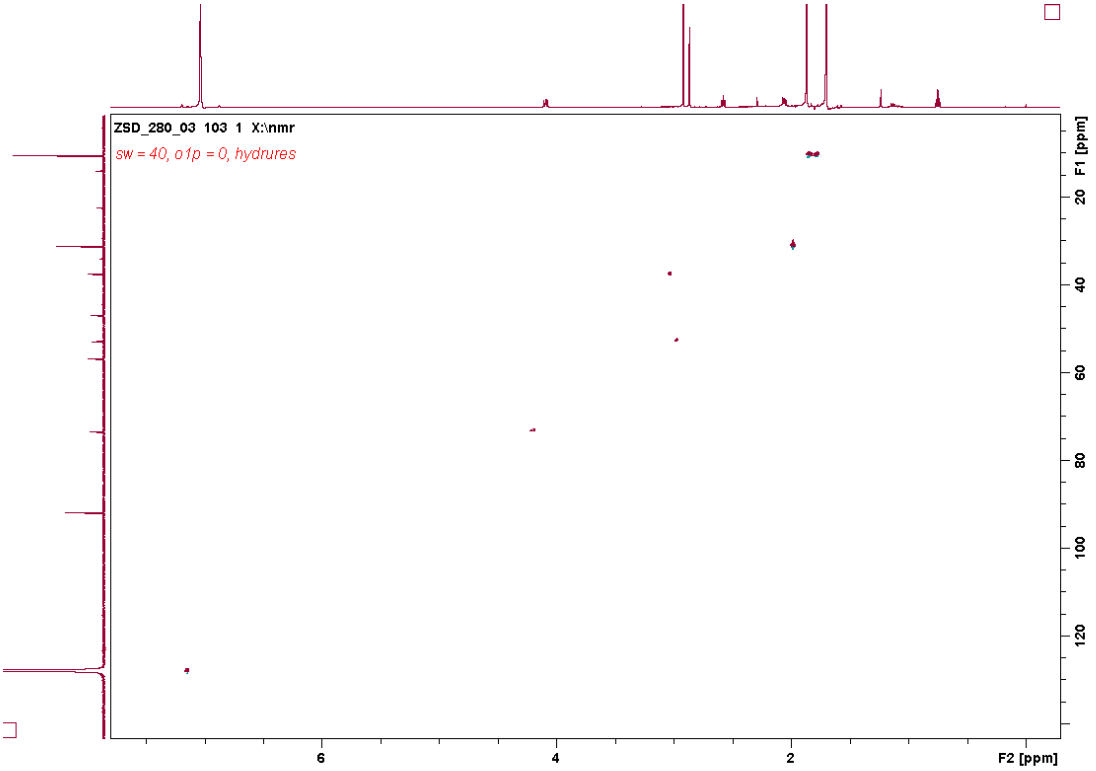

Figure S 24 : ¹H-¹³C HSQC 2D NMR spectrum of compound 5 in C_6D_6 solution, recorded at 500 MHz, 298K. Spectra presented along axes are external projections (for clarity) of ¹H and ¹³C spectra recorded on the same sample.

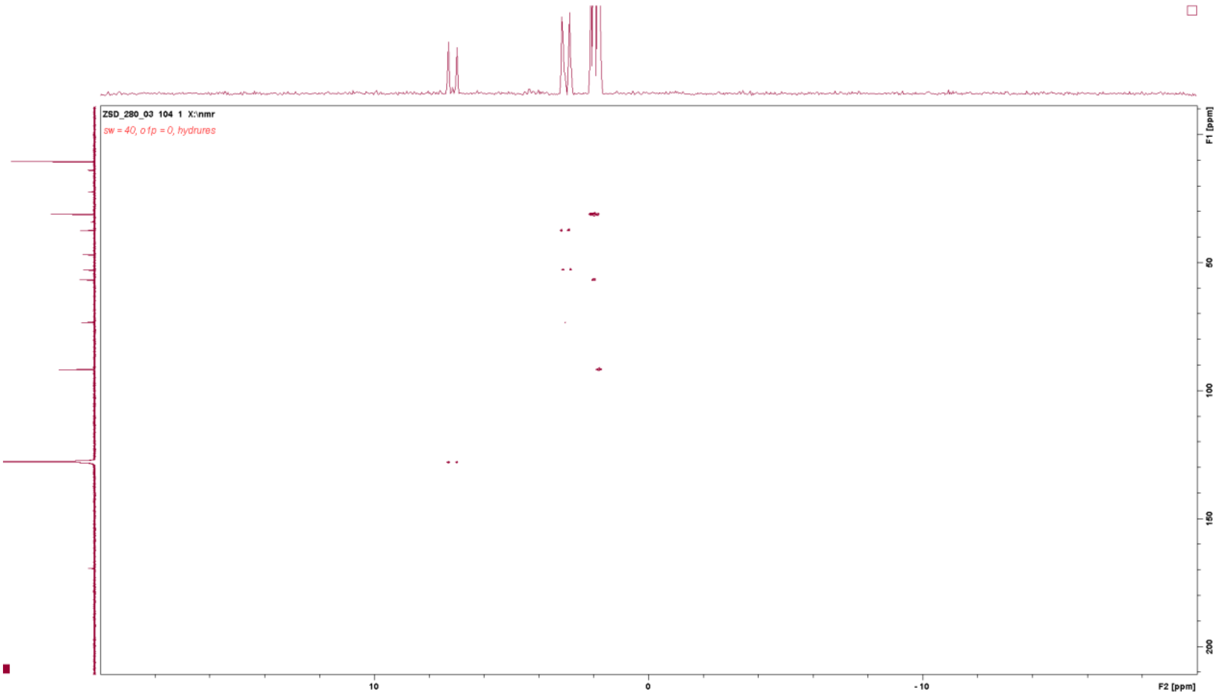

Figure S 25 : ^{1}H-^{13}C HMBC 2D NMR spectrum of compound 5 in C_6D_6 solution, recorded at 500 MHz, 298K. Spectra presented along axes are external projections (for clarity) of ^{1}H and ^{13}C spectra recorded on the same sample.

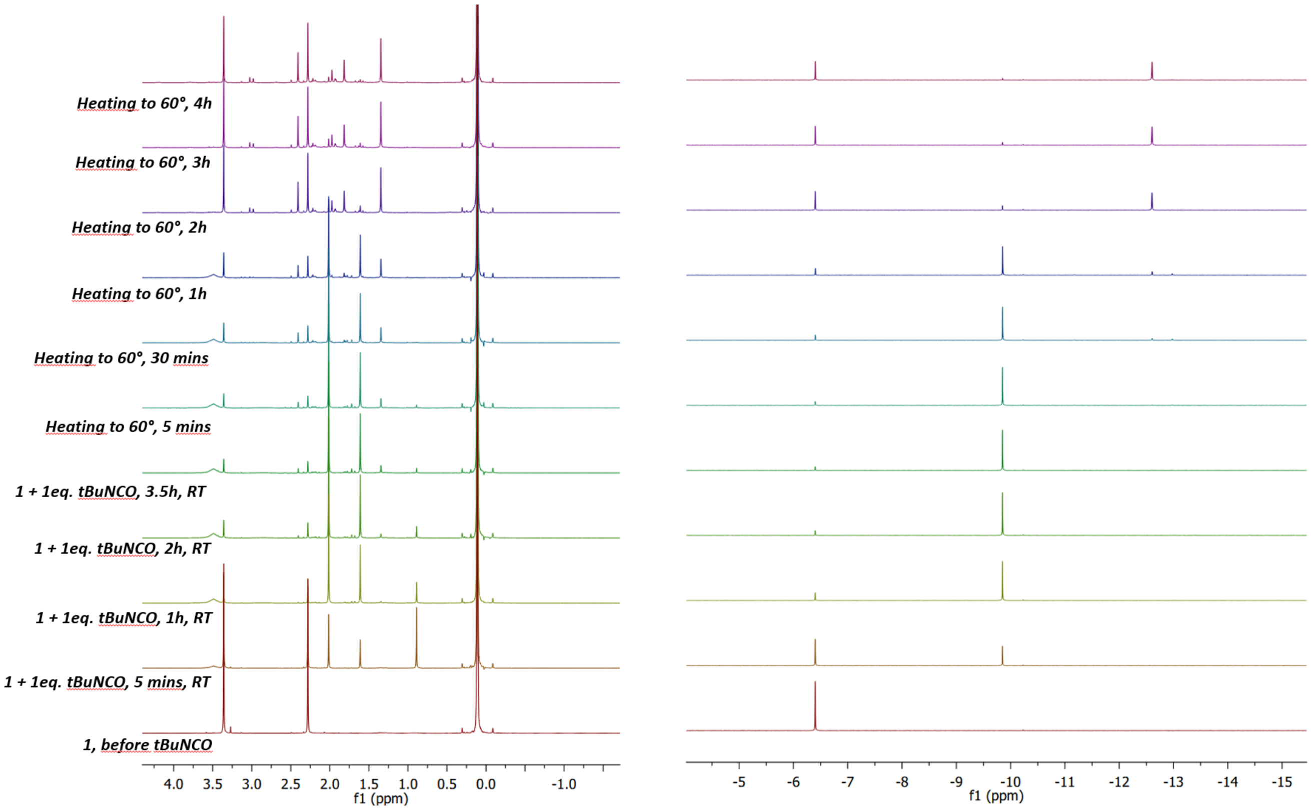

Figure S 26 : ^{1}H NMR spectra recorded (300 MHz, 298K, d1 = 20s) in C_6D_6 (with 0.184 M HMDSO as internal standard, 0.01 ppm) during the reaction of 1 with one equivalent of tBuNCO, passing through complex 4 to yield complex 5. The consumption and subsequent regeneration of 1 can be most easily observed from the hydride signal at -6.4 ppm. Complex 4 converts to 5 at room temperature (as observed in the signal at 1.8 ppm, attributed to the Cp*- Me moiety) however, heating was used to ensure conversion and a timely experiment duration.

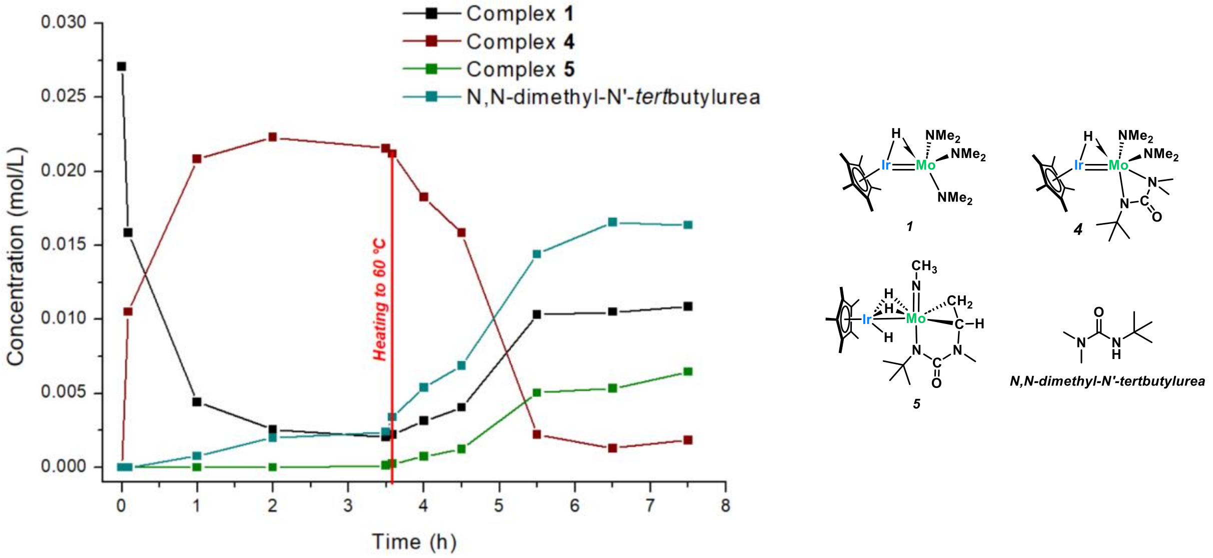

Figure S 27: Concentrations of species from Figure S 26 as determined from NMR integration against the HMDSO internal standard compared to the Cp* groups of 1, 4 and 5 and the *t*Bu group of the N,N-dimethyl-N'-tert-butyl-urea. The concentration of the urea equals 95% of the sum of the final concentrations of 1 and 4 Note that the yield of 5 from this reaction (24%) is quite low

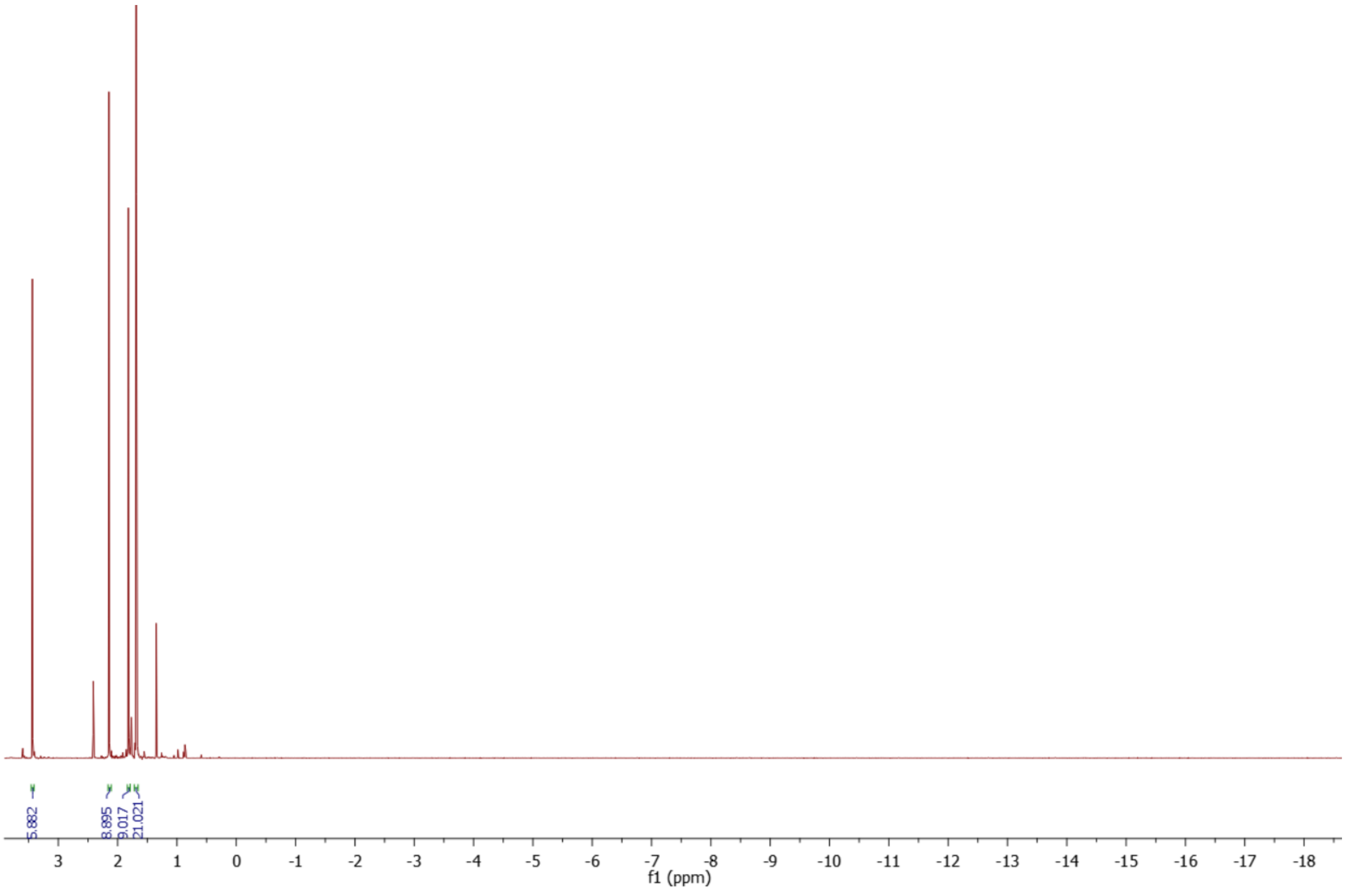

Figure S 28 : ^{1}H NMR spectrum of compound 6 in C_6D_6 solution, recorded at 500 MHz, 298K.

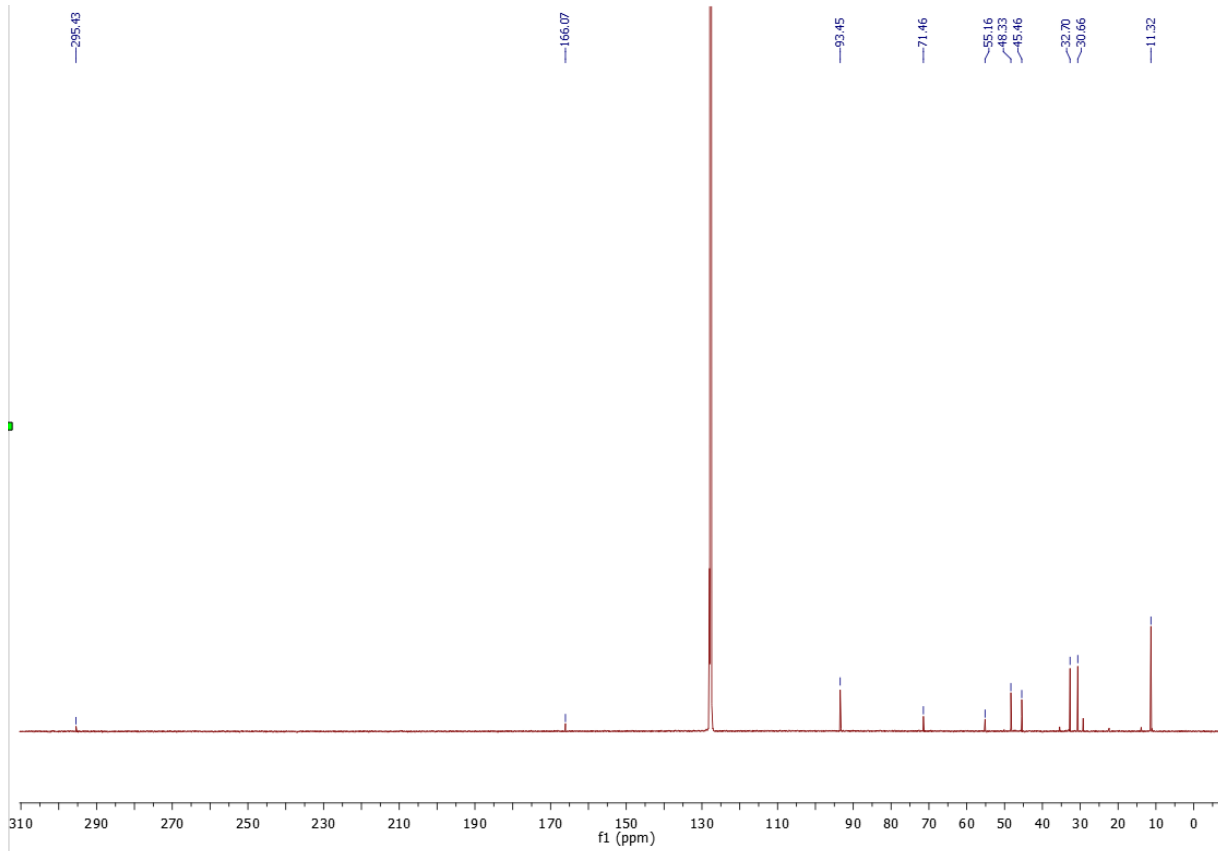

Figure S 29 : ^{13}C NMR spectrum of compound 6 in C_6D_6 solution, recorded at 125 MHz, 298K.

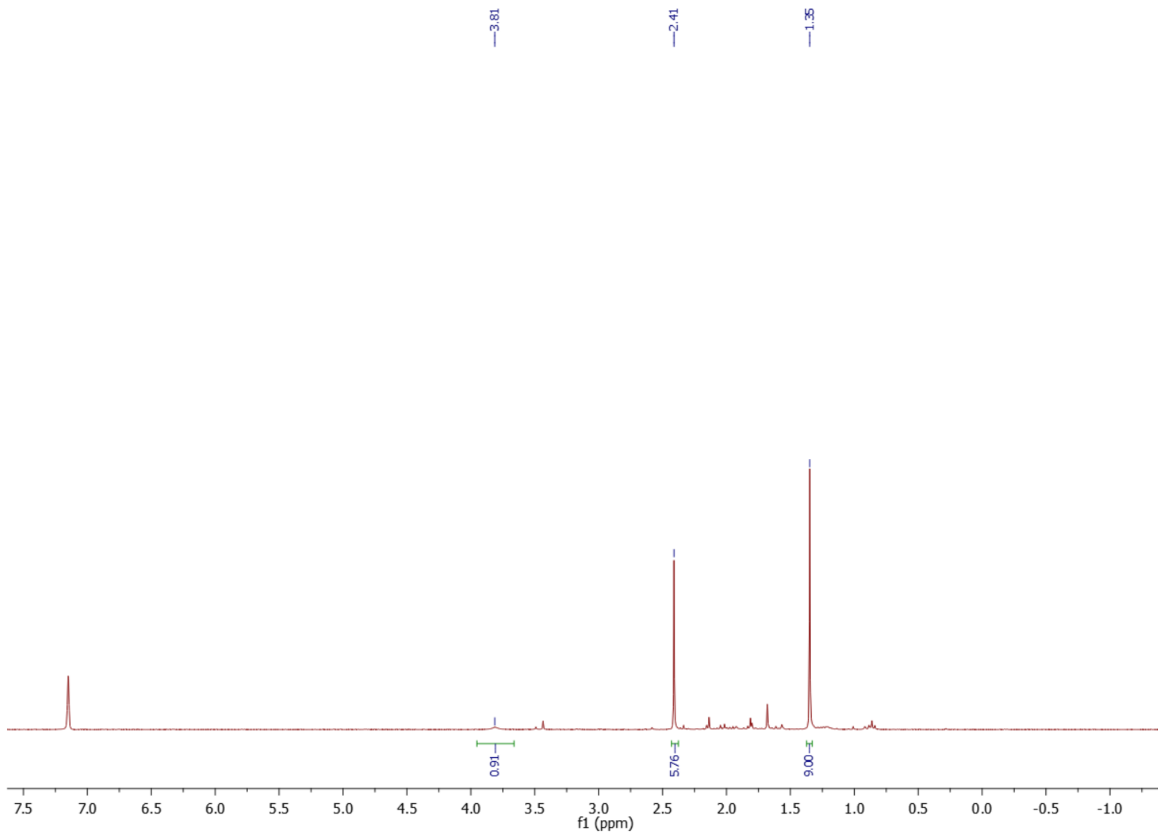

Figure S 30 : ^{1}H NMR spectrum of N,N-dimethyl-N'-*tert*butylurea isolated from the reaction mixture, recorded in C_6D_6 solution, at 300 MHz, 298K.

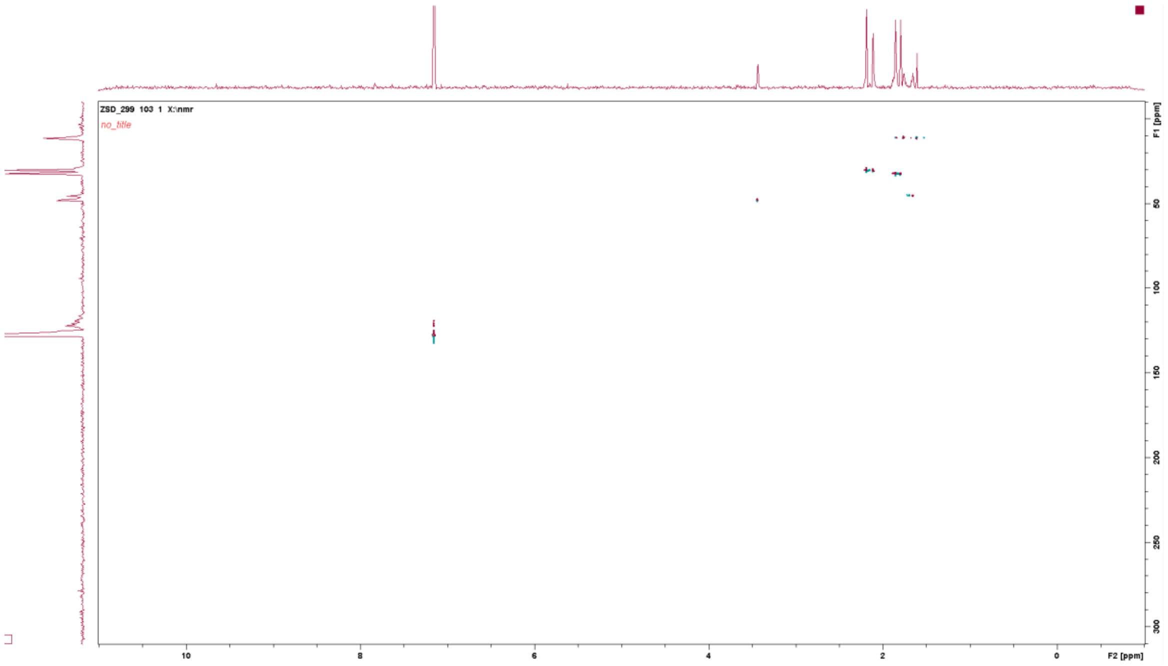

Figure S 31 : ^{1}H-^{13}C HSQC 2D NMR spectrum of compound 6 in C_6D_6 solution, recorded at 500 MHz, 298K.

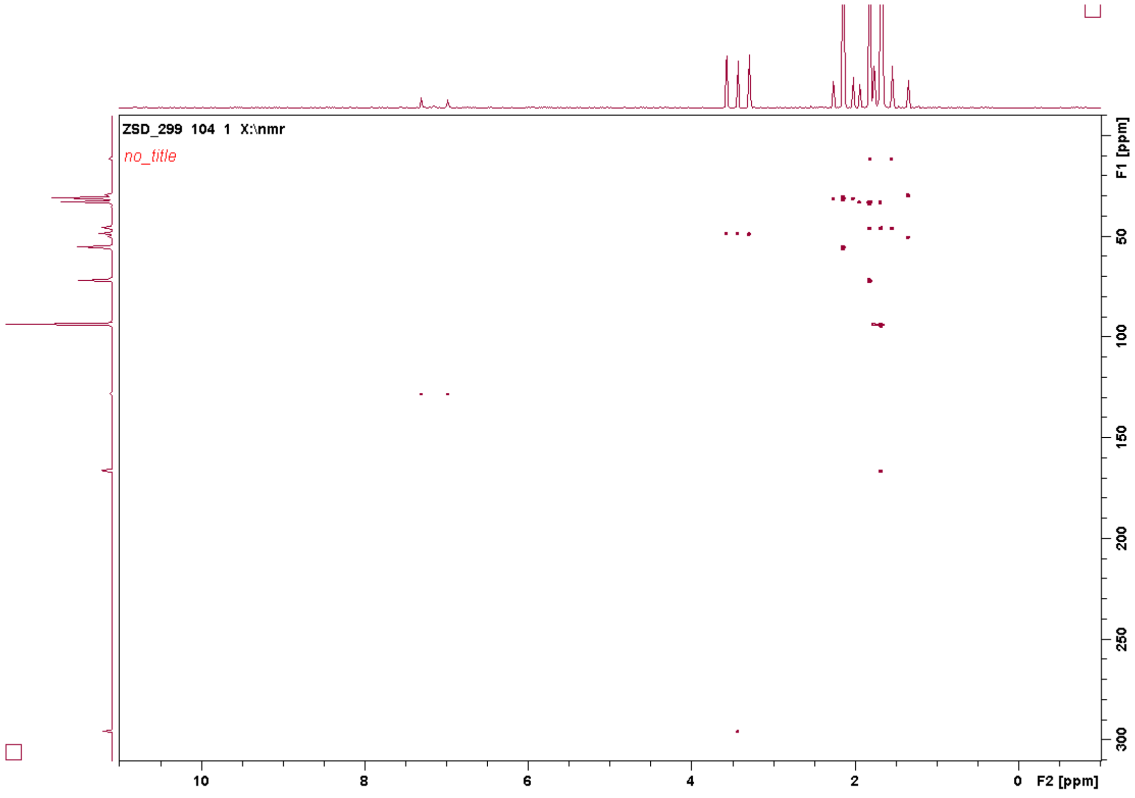

Figure S 32: ^{1}H-^{13}C HMBC 2D NMR spectrum of compound 6 in C_6D_6 solution, recorded at 500 MHz, 298K.

Diffuse Reflectance Infrared Fourier Transform (DRIFT) Spectra

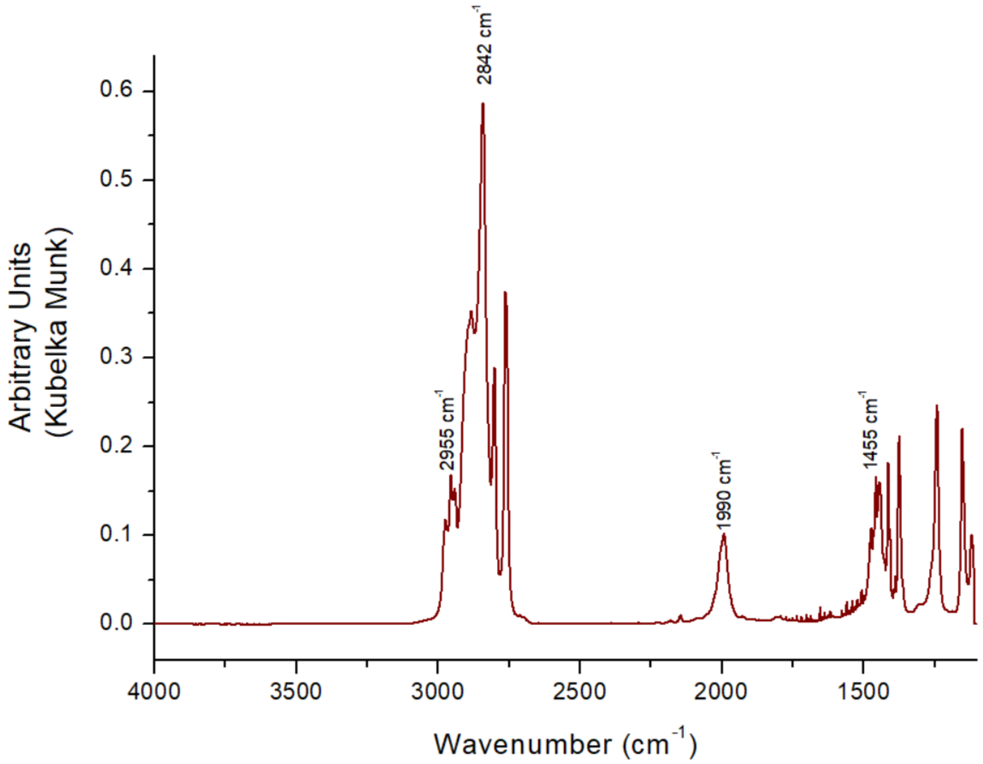

Figure S 33 : DRIFT spectrum of compound 1, diluted in KBr.

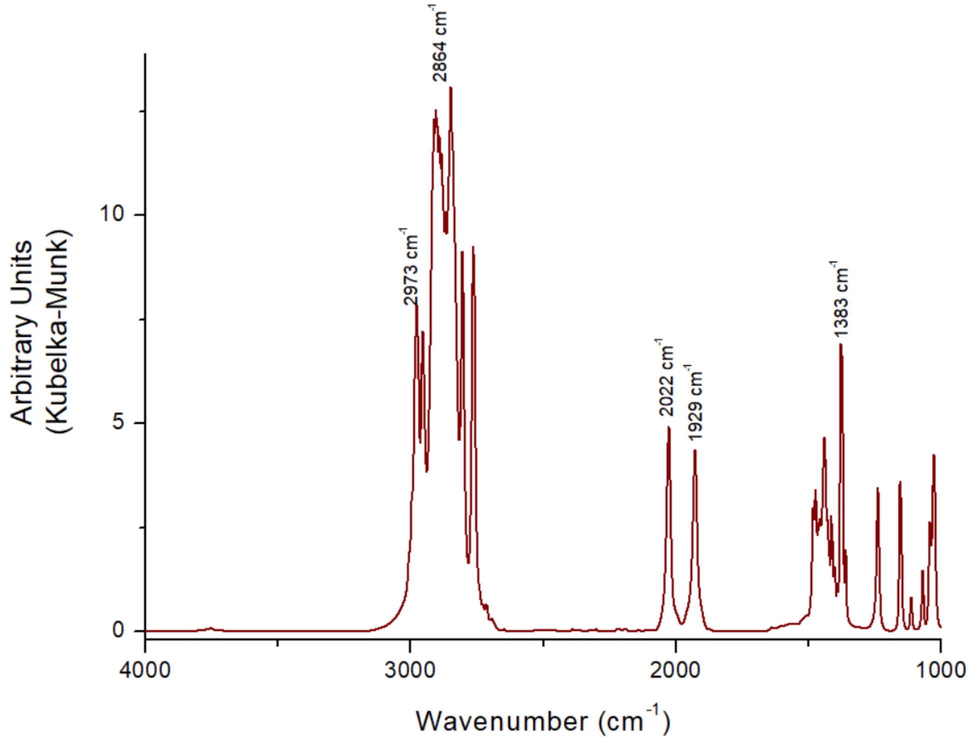

Figure S 34 : DRIFT spectrum of compound 2, pure crystalline material.

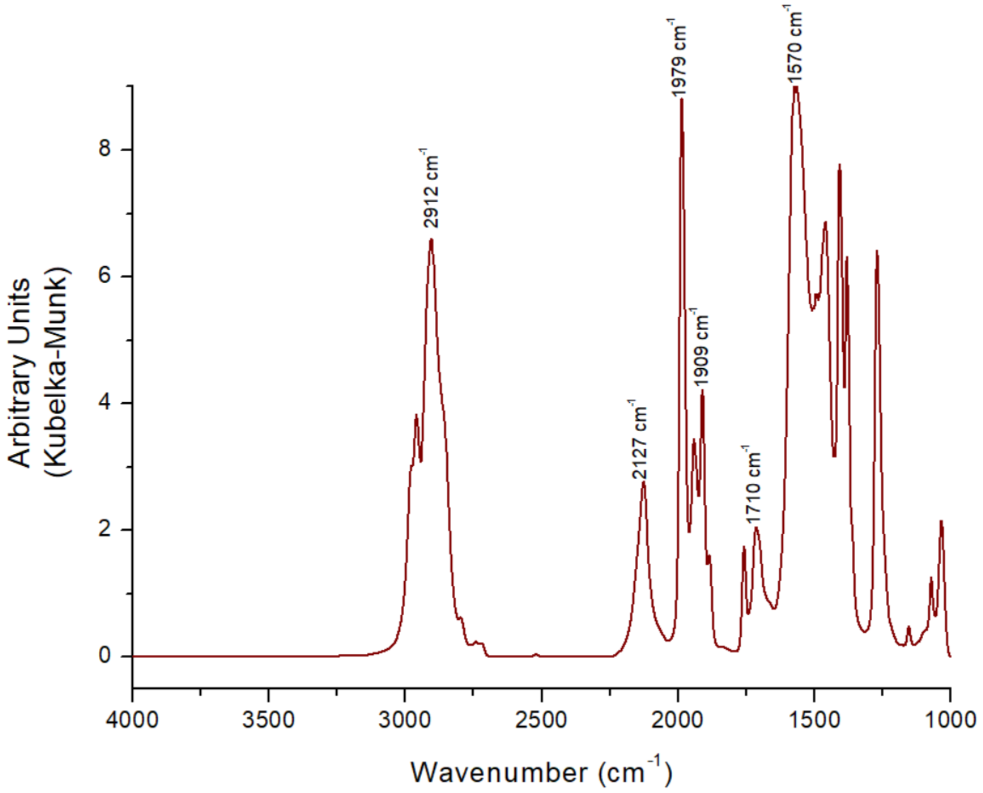

Figure S 35 : DRIFT spectrum of compound 3, pure crystalline material.

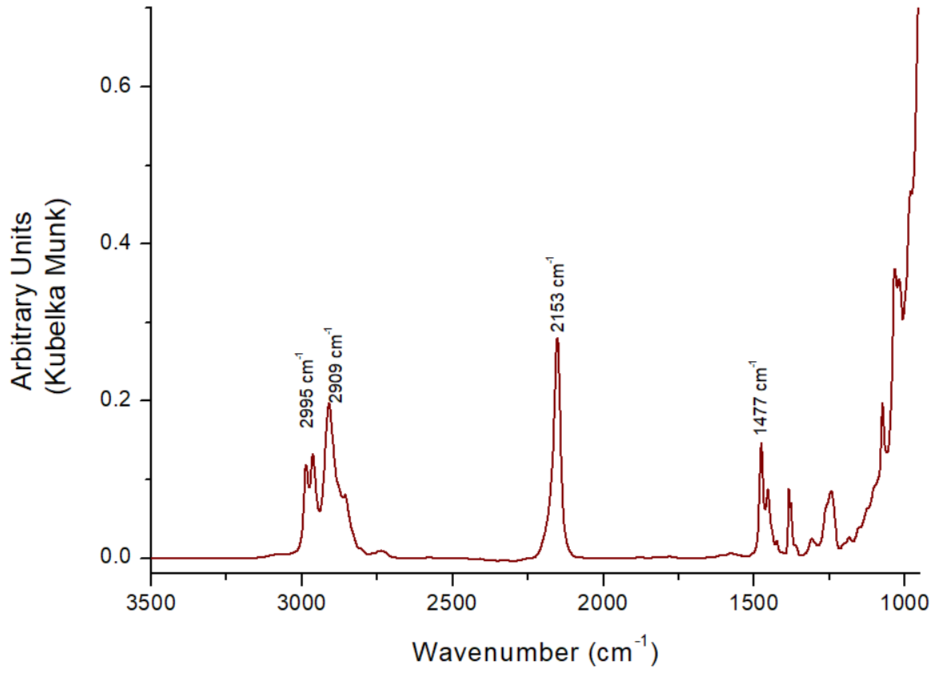

Figure S 36 : DRIFT spectrum of compound 4, diluted in KBr.

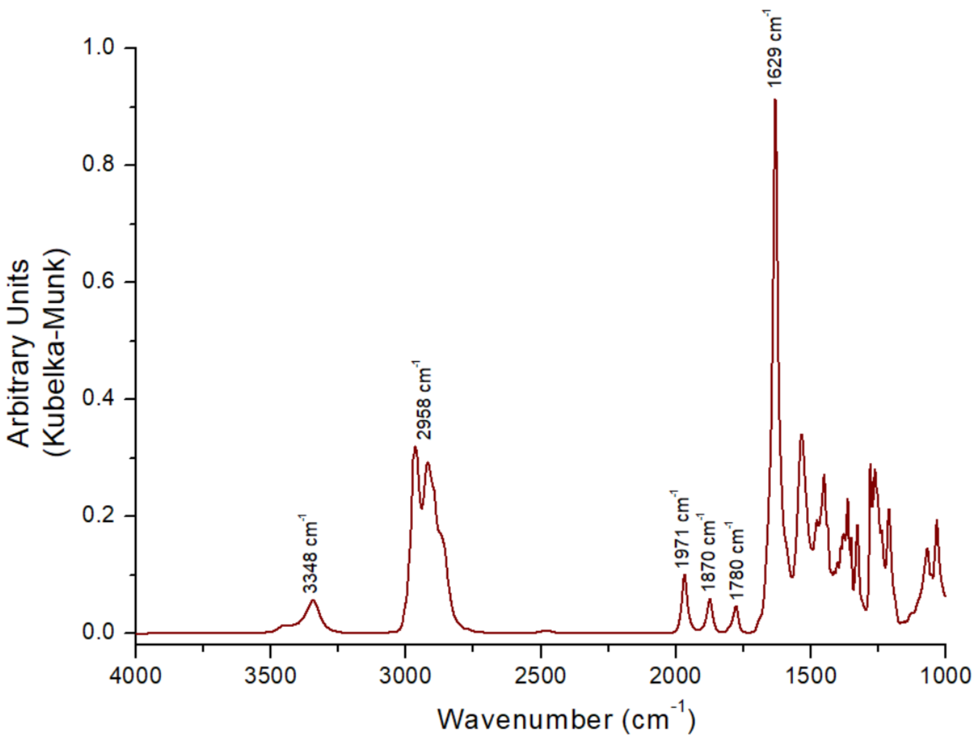

Figure S 37 : DRIFT spectrum of compound 5, diluted in KBr.

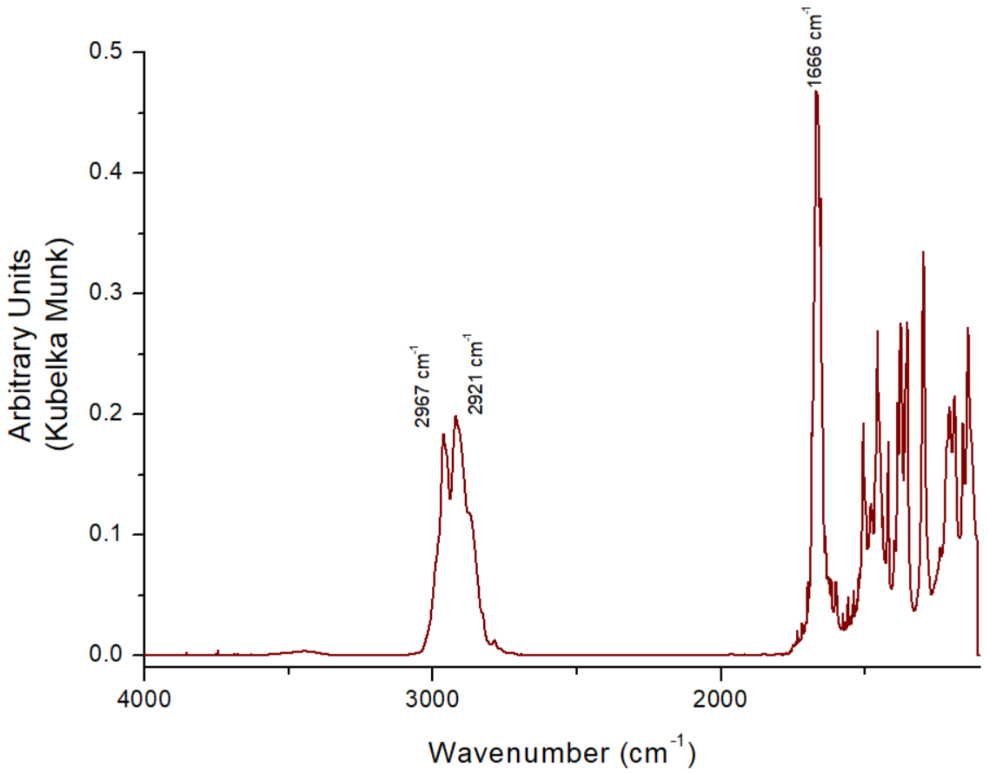

Figure S 38 : DRIFT spectrum of compound 6, diluted in KBr.

UV-Vis Spectra

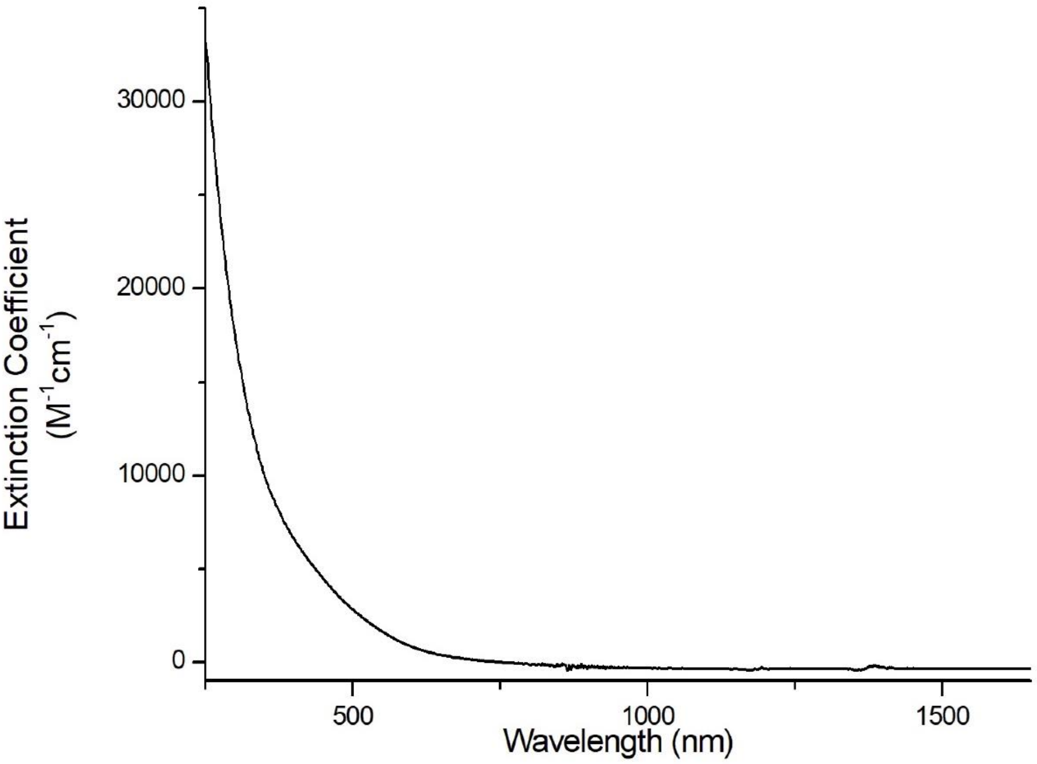

Figure S 39 : UV-Vis spectrum of 1, recorded in THF solution (0.061 mM). Signal noise at 850, 1200 and 1350 nm are artifacts from the instrument.

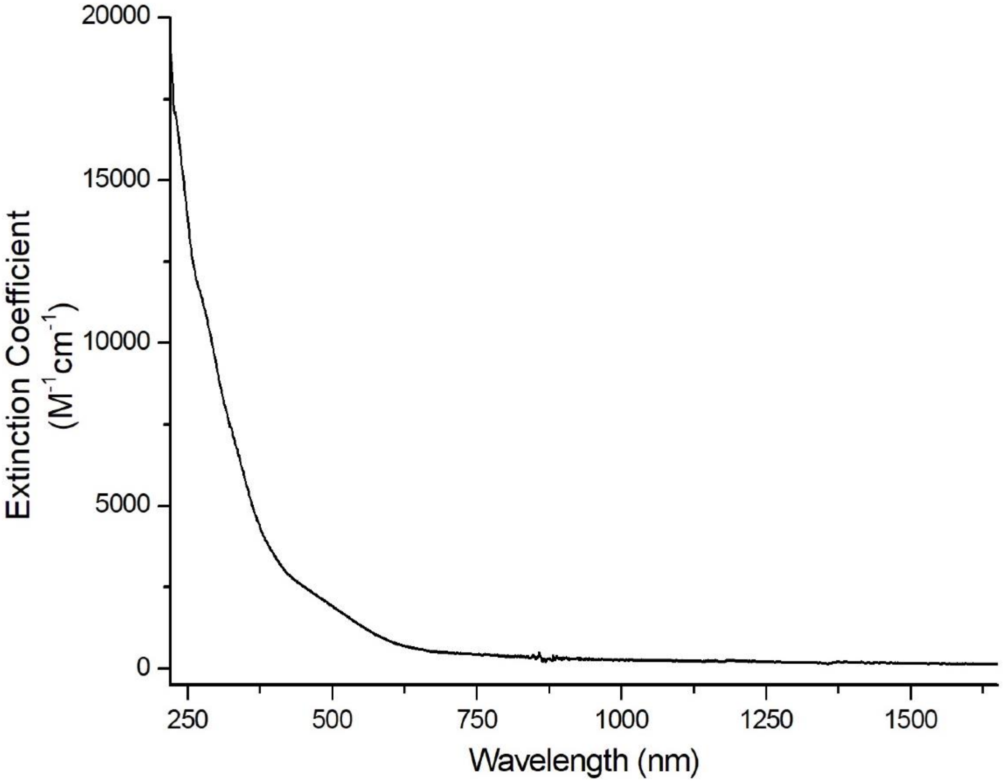

Figure S 40: UV-Vis spectrum of complex 2, recorded in THF solution (0.091 mM). Signal noise at 850, 1200 and 1350 nm are artifacts from the instrument

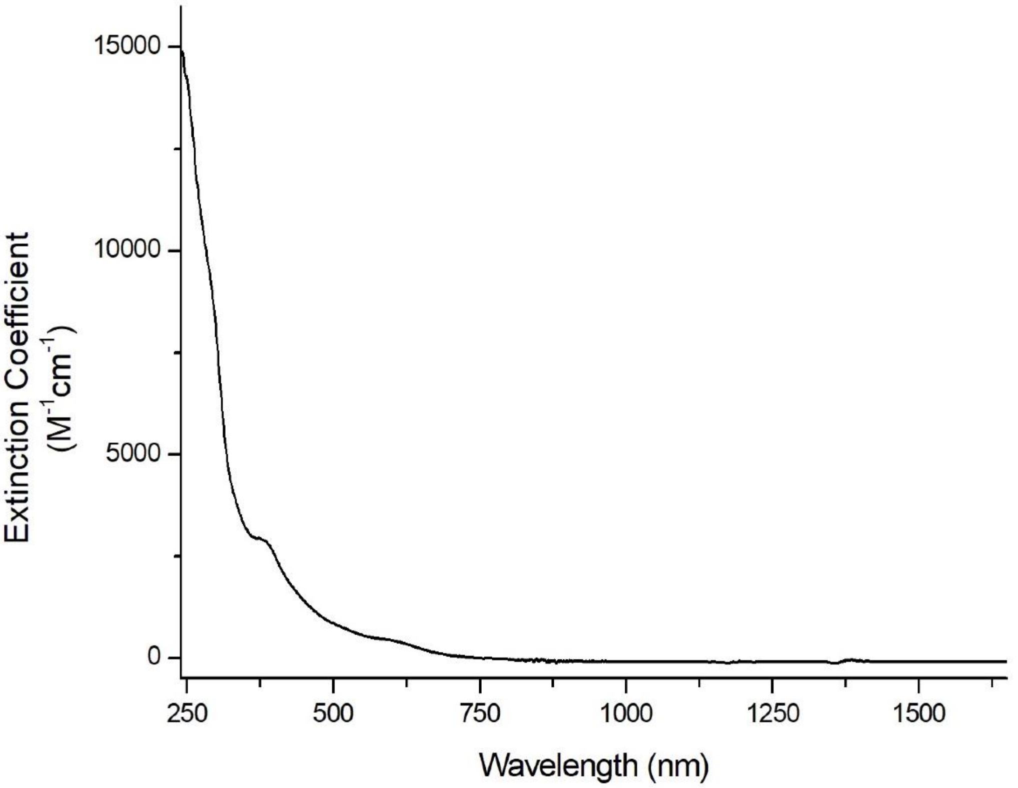

Figure S 41: UV Vis spectrum of complex 3, recorded in THF solution (0.16 mM). Signal noise at 850, 1200 and 1350 nm are artifacts from the instrument.

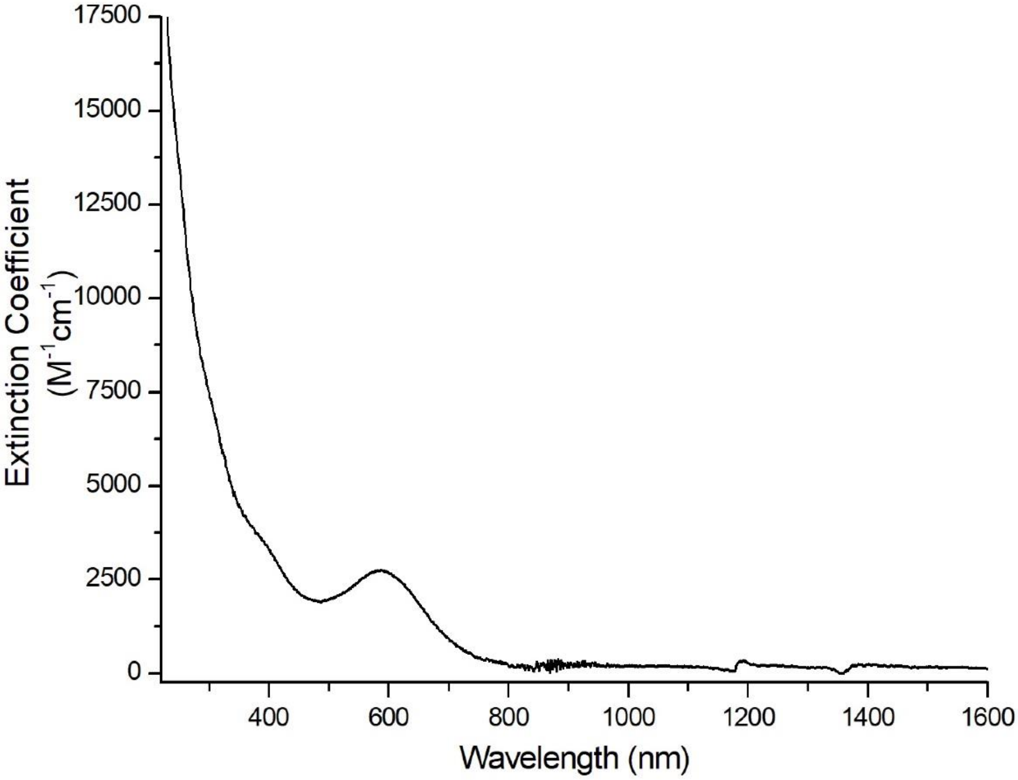

Figure S 42 : UV Vis spectrum of compounds 6, recorded in THF solution (0.043 mM). Signal noise at 850, 1200 and 1350 nm are artifacts from the instrument

X-ray Crystallography Data

Table S1: Literature bond lengths and FSR analysis of homo- and heterobimetallic complexes involving molybdenum and iridium, found in the CCDC database. The structures are drawn as they appear in their respective articles.

	Structure	M-M' Bond Length	Formal Shortness ratio	Reference
1	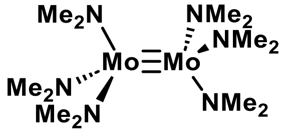	2.211(2)	0.856	[9]
2	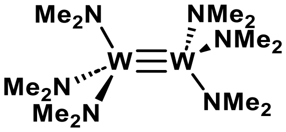	2.294(1)	0.883	[10]
3	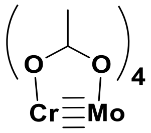	2.050(1)	0.832	[11]
4	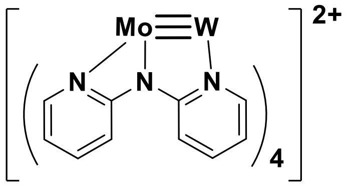	2.736(4)	1.056	[12]
5	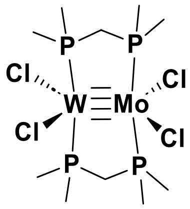	2.193(2)	0.846	[13]
6	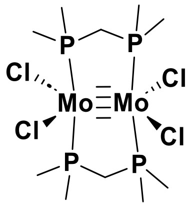	2.1271(8)	0.823	[13]
7	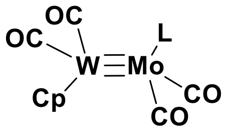	2.562(1)	0.988	[14]
8	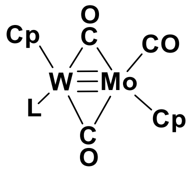	2.526(1)	0.975	[15]
9	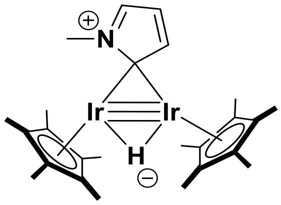	2.462(2)	0.977	[16]

10	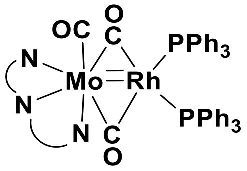	2.6066(5)	1.027	17
11	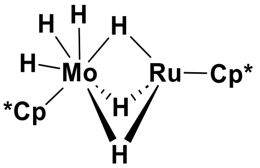	2.5255(7)	0.997	18
12	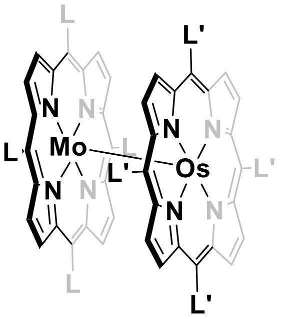	2.238(3)	0.880	19
13	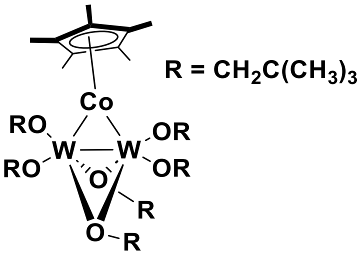	Co – W = 2.279(5) – 2.340(5)<br>W – W = 2.504(3)	Co – W = 0.928<br>W – W =0.96	20
14	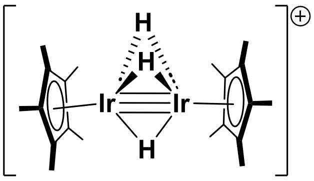	2.465(3)	0.978	21
15	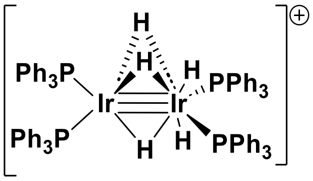	2.518	0.999	22
16	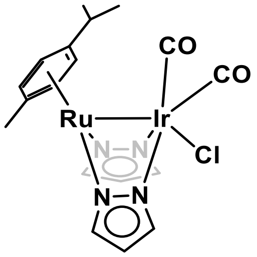	2.6962	1.078	23

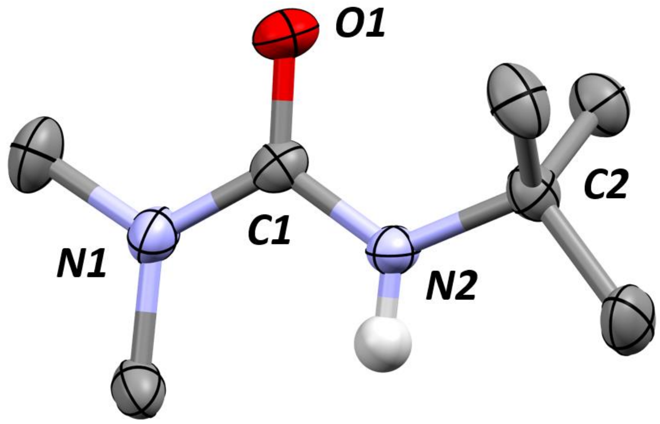

Figure S 43: X-ray crystal structure of N,N-dimethyl-N'-*tert*butyl urea. Displacement ellipsoids represented at 50% with most hydrogens removed for clarity. Relevant bond distances (Å): N1 – C1 = 1.369(2), C1 – O1 = 1.239(2), C1 – N2 = 1.358(2), N2 – C2 = 1.478(2)

Table S2: Crystallographic data collections and refinements details

**Compound**	**1**	**2**	**3**	**4**
Formula	$C_{16}H_{33}IrMoN_3$	$C_{24}H_{42}N_2MoIr_2$	$C_{26}H_{42}N_2O_4MoIr_2$	$C_{21}H_{42}IrMoN_4O$
$D_{calc.}$/ g cm^{-3}	1.827	2.079	2.100	1.713
μ/mm^{-1}	7.201	10.378	9.507	5.749
Formula Weight	555.59	838.93	926.95	654.72
Colour	black	black	black	dark red
Shape	plate-shaped	block-shaped	plate-shaped	block-shaped
Size/mm^3	0.35×0.28×0.05	0.33×0.26×0.15	0.27×0.20×0.07	0.44×0.30×0.22
T/K	100.0(2)	100.1(4)	150.01(10)	100.0(3)
Crystal System	monoclinic	triclinic	monoclinic	triclinic
Space Group	$P2_1/c$	P-1	$P2/n$	P-1
a/Å	18.3443(4)	10.3728(2)	10.9967(2)	10.5680(2)
b/Å	8.22852(16)	11.1282(2)	10.9728(2)	14.3581(2)
c/Å	13.3820(3)	11.9391(2)	12.1515(2)	17.6451(2)
α/°	90	86.4790(10)	90	100.1480(10)
β/°	90.251(2)	80.4780(10)	90.354(2)	101.6170(10)
γ/°	90	80.6460(10)	90	98.0710(10)
V/Å^3	2019.94(7)	1340.23(4)	1466.23(4)	2538.22(7)
Z	4	2	2	4
Z'	1	1	0.5	2
Wavelength/Å	0.71073	0.71073	0.71073	0.71073
Radiation type	Mo K_α	Mo K_α	Mo K_α	Mo K_α
Θ_{min}/°	2.713	2.440	2.490	2.411
Θ_{max}/°	30.334	30.615	30.307	30.731
Measured Refl's.	46772	36487	33776	112854
Indep't Refl's	5431	6919	3901	13733
Refl's I≥2 σ(I)	4428	6208	3310	12780
R_{int}	0.0822	0.0840	0.0461	0.0459
Parameters	298	276	263	800
Restraints	919	0	623	2607
Largest Peak	2.741	3.306	2.020	1.769
Deepest Hole	-4.443	-3.973	-2.134	-1.801
GooF	1.060	1.029	1.028	1.029
wR_2 (all data)	0.1181	0.1113	0.0668	0.0635
wR_2	0.1114	0.1095	0.0638	0.0626
R_1 (all data)	0.0646	0.0445	0.0448	0.0293
R_1	0.0495	0.0409	0.0352	0.0265

**Compound**	**5**	**6**	**N,N-dimethyl-N‘-tertbutylurea**
Formula	$C_{19}H_{33}IrMoN_3O$	$C_{24}H_{45}IrMoN_4O_2$	$C_7H_{16}N_2O$
$D_{calc.}$/ g cm^{-3}	1.766	1.712	1.086
μ/mm^{-1}	6.376	5.309	0.585
Formula Weight	607.62	709.78	144.22
Colour	black	black	black
Shape	block-shaped	block-shaped	block-shaped
Size/mm^3	0.51×0.23×0.13	0.21×0.15×0.09	0.20×0.10×0.10
T/K	100.02(16)	100.0(4)	100.0(3)
Crystal System	monoclinic	monoclinic	orthorhombic
Space Group	$P2_1/n$	$P2_1/c$	*Pbca*
a/Å	10.4733(2)	10.3416(2)	9.9756(3)
b/Å	14.1270(3)	18.4312(4)	11.6881(3)
c/Å	15.9700(3)	14.9177(3)	15.1321(4)
α/°	90	90	90
β/°	104.700(2)	104.443(2)	90
γ/°	90	90	90
V/Å^3	2285.52(8)	2753.56(11)	1764.34(8)
Z	4	4	8
Z'	1	1	1
Wavelength/Å	0.71073	0.71073	1.54184
Radiation type	Mo K_α	Mo K_α	Cu K_α
Θ_{min}/°	2.474	2.314	5.848
Θ_{max}/°	30.508	29.940	74.130
Measured Refl's.	49581	119373	8064
Indep't Refl's	6080	7504	1722
Refl's I≥2 σ(I)	5164	6502	1454
R_{int}	0.0610	0.0767	0.0308
Parameters	236	304	96
Restraints	0	0	0
Largest Peak	3.224	1.447	0.292
Deepest Hole	-1.926	-2.891	-0.310
GooF	1.038	1.253	1.081
wR_2 (all data)	0.0880	0.1010	0.1086
wR_2	0.0828	0.0988	0.1030
R_1 (all data)	0.0442	0.0617	0.0522
R_1	0.0337	0.0520	0.0418

Computational Data

All DFT calculations were carried out with the Gaussian 09 suite of programs.[24] Geometries were fully optimized in gas phase without symmetry constraints, employing the B3PW91 functional.[25,26] The nature of the extrema was verified by analytical frequency calculations. The calculation of electronic energies and enthalpies of the extrema of the potential energy surface (minima and transition states) were performed at the same level of theory as the geometry optimizations. IRC calculations were performed to confirm the connections of the optimized transition states. Iridium and Molybdenum atoms were treated with a small-core effective core potential (60 MWB), associated with its adapted basis set[27–29] augmented with a polarization function (ζ_f = 0.938 and 1.0434 respectively for Ir and Mo).[30] For the other elements (H, C, N and O), Pople's triple-ζ basis set 6-311G(d,p) was used.[31–33] The electronic charges (at the DFT level) were computed using the natural population analysis (NPA) technique.[34]

Figure S 44: Computational data for complex 1.

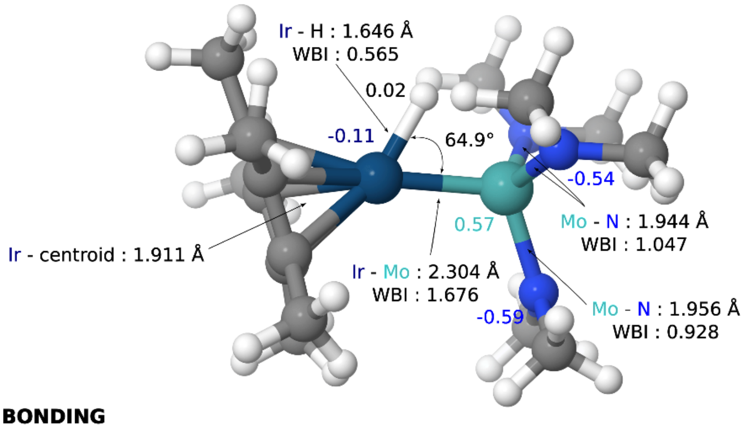

BONDING

(1.75512) BD (1)Ir 6 -Mo 7

(49.13%) 0.7009*Ir 6 s(38.03%) p 0.16(6.06%) d 1.47(55.89%) f 0.00(0.03%

(50.87%) 0.7132*Mo 7 s(32.26%) p 0.54(17.53%) d 1.55(50.13%) f 0.00(0.07

(1.87579) BD (2)Ir 6 -Mo 7

(55.25%) 0.7433*Ir 6 s(0.01%) p 1.00(1.58%) d 62.40(98.40%) f 0.01(0.01

(44.75%) 0.6689*Mo 7 s(0.00%) p 1.00(18.08%) d 4.53(81.85%) f 0.00(0.07

(1.65990) BD (1)Ir 6 - H 55

(47.66%) 0.6904*Ir 6 s(42.97%)p 0.26(11.31%)d 1.06(45.68%) f 0.00(0.03%)

(52.34%) 0.7234* H 55 s(99.88%)p 0.00(0.12%)

Second Order Perturbation Theory Analysis

LP Ir 6 -> LP* Mo 7 180.49

LP Ir 6 -> BD*(1)Ir 6 -Mo 7 153.08

BD Ir 6 - H 55 -> LP*(1)Mo 7 1848.15

BD Ir 6 - H 55 -> BD*(1)Ir 6 -Mo 7 1721.62

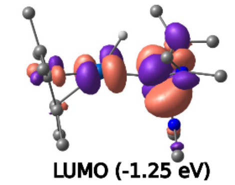

LUMO (-1.25 eV)

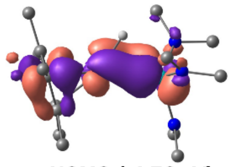

HOMO (-4.76 eV)

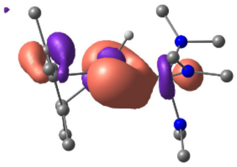

HOMO-1 (-4.97 eV)

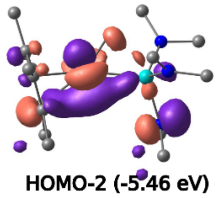

HOMO-2 (-5.46 eV)

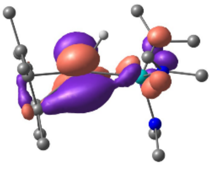

HOMO-4 (-5.78 eV)

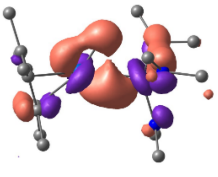

HOMO-7 (-7.15 eV)

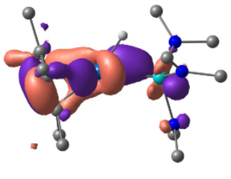

HOMO-8 (-7.49 eV)

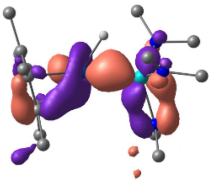

HOMO-9 (-7.50 eV)

Figure S 45: Computational data for complex 2.

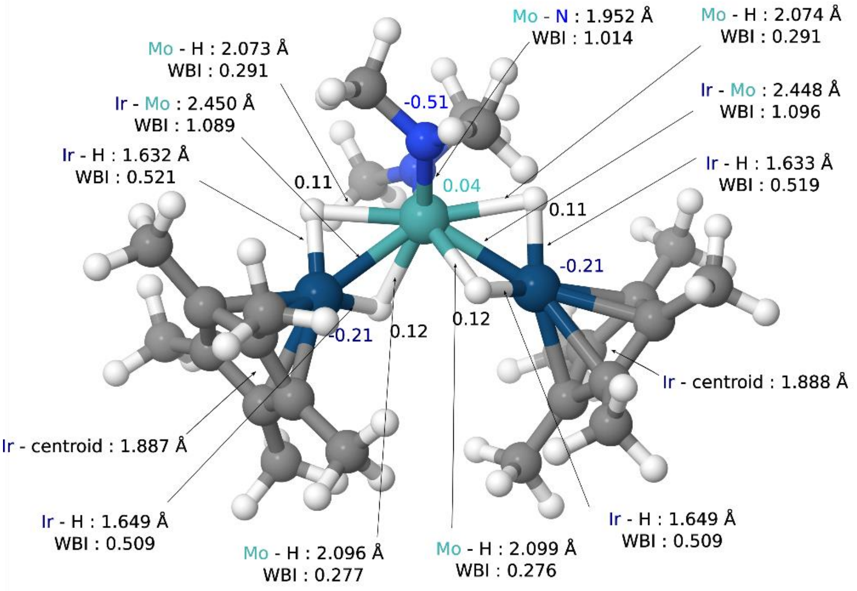

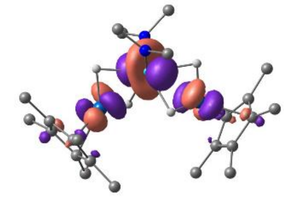

LUMO (-1.33 eV)

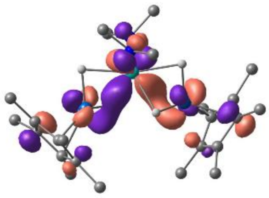

HOMO (-4.70 eV)

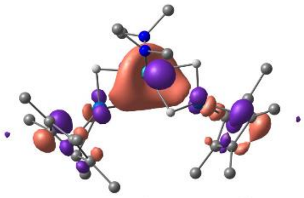

HOMO-2 (-5.05 eV)

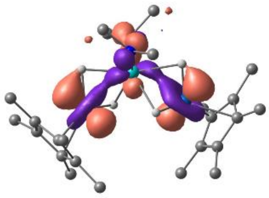

HOMO-9 (-6.80 eV)

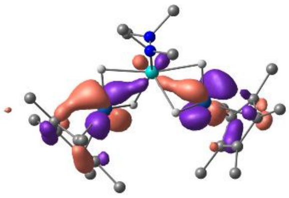

HOMO-10 (-7.01 eV)

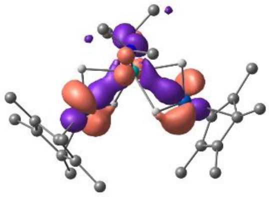

HOMO-11 (-7.12 eV)

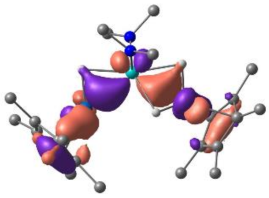

HOMO-12 (-7.57 eV)

BONDING

(1.92397) BD (1)Ir 6 -Mo 7

(44.95%) 0.6704*Ir 6 s(15.18%)p 0.24(3.68%)d 5.34(81.13%)f 0.00(0.01%)

(55.05%) 0.7420*Mo 7 s(1.46%)p 2.87(4.20%)d64.53(94.32%) f 0.01(0.02%)

(1.61124) BD (1)Ir 6 - H 73

(53.77%) 0.7333*Ir 6 s(31.02%)p 0.37(11.55%)d 1.85(57.40%)f 0.00(0.03%)

(46.23%) 0.6800* H 73 s(99.85%)p 0.00(0.15%)

(1.62558) BD (1)Ir 6 - H 75

(53.54%) 0.7317*Ir 6 s(30.96%)p 0.37(11.60%)d 1.85(57.41%) f 0.00(0.03%)

(46.46%) 0.6816* H 75 s(99.85%)p 0.00(0.15%)

(1.50586) BD (1)Ir 15 - H 72

(45.95%) 0.6779*Ir 15 s(37.63%)p 0.73(27.40%)d 0.93(34.85%) f 0.00(0.12%)

(54.05%) 0.7352* H 72 s(99.85%)p 0.00(0.15%)

(1.53706) BD (1)Ir 15 - H 74

(46.74%) 0.6837*Ir 15 s(28.44%)p 1.18(33.58%)d 1.33(37.85%) f 0.00(0.13%)

(53.26%) 0.7298* H 74 s(99.86%)p 0.00(0.14%)

Second Order Perturbation Theory Analysis

LP Mo 7	-> LP* Ir 6	109.6
LP Ir 6	-> LP* Mo 7	47.7
LP (1)Ir 6	-> BD* Ir 6 -Mo 7	15.6
BD Ir 6 -Mo 7	-> LP* Ir 6	226.9
BD Ir 6 -Mo 7	-> LP* Mo 7	648.4
BD Ir 6 -Mo 7	-> BD* Ir 6 -Mo 7	214.2
BD Ir 6 -Mo 7	-> BD* Ir 6 - H 73	283.7
BD Ir 6 -Mo 7	-> BD* Ir 6 - H 75	281.5
BD Ir 6 - H 73	-> LP* Mo 7	1980.8
BD Ir 6 - H 73	-> BD* Ir 6 -Mo 7	4620.9
BD Ir 6 - H 75	-> LP* Mo 7	2054.7
BD Ir 6 - H 75	-> BD* Ir 6 -Mo 7	4673.4

LP Mo 7	-> LP* Ir 15	590.8
LP Mo 7	-> BD* Ir 15 - H 72	386.6
LP Mo 7	-> BD* Ir 15 - H 74	392.7
LP Ir 15	-> LP* Mo 7	13.5
BD Ir 15 - H 72	-> LP* Mo 7	3812.9
BD Ir 15 - H 72	-> BD* Ir 6 -Mo 7	1.02
BD Ir 15 - H 74	-> LP* Mo 7	34627.7
BD Ir 15 - H 74	-> BD* Ir 6 -Mo 7	50.1

Figure S 46: Computational data for complex 3.

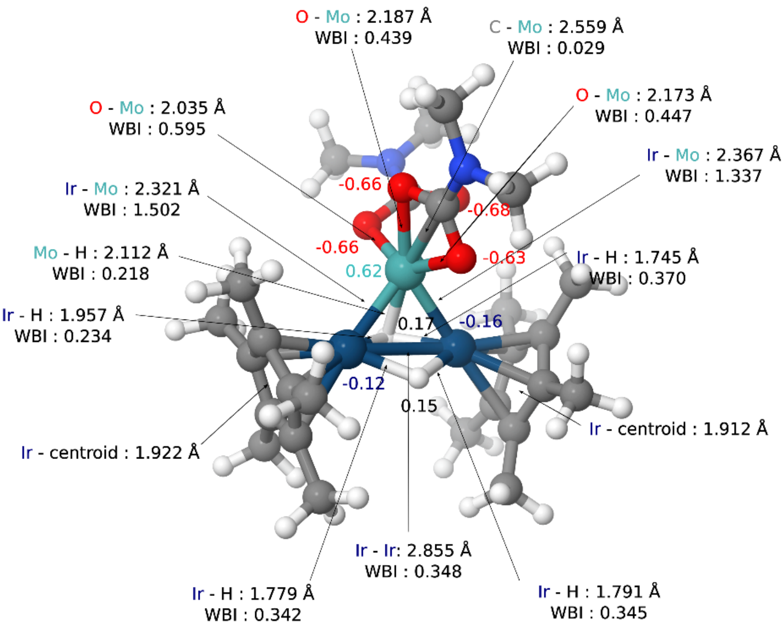

BONDING

(1.94225) BD (1)Ir 6 -Mo 7

(62.51%) 0.7907*Ir 6 s(5.29%)p 0.34(1.81%)d17.57(92.88%) f 0.00(0.01%)

(37.49%) 0.6123*Mo 7 s(9.77%)p 0.45(4.42%)d 8.78(85.75%) f 0.01(0.05%)

(1.82095) BD (2)Ir 6 -Mo 7

(61.81%) 0.7862*Ir 6 s(0.05%)p29.55(1.55%)d99.99(98.39%) f 0.20(0.01%)

(38.19%) 0.6180*Mo 7 s(0.36%)p 4.68(1.70%)d99.99(97.89%) f 0.13(0.05%)

(1.94394) BD (1)Mo 7 -Ir 16

(39.74%) 0.6304*Mo 7 s(6.97%)p 0.59(4.11%)d12.75(88.87%) f 0.01(0.05%)

(60.26%) 0.7763*Ir 16 s(4.05%)p 0.34(1.39%)d23.36(94.55%) f 0.00(0.01%)

(1.74197) BD (2)Mo 7 -Ir 16

(34.24%) 0.5852*Mo 7 s(0.76%)p 2.69(2.05%)d99.99(97.15%) f 0.06(0.05%)

(65.76%) 0.8109*Ir 16 s(0.17%)p10.44(1.76%)d99.99(98.07%) f 0.03(0.01%)

Second Order Perturbation Theory Analysis

LP Ir 6	-> LP* Mo 7	242.3
LP Ir 6	-> BD* Ir 6 -Mo 7	94.5
LP Ir 6	-> BD* Mo 7 -Ir 16	18.9
LP Ir 16	-> BD* Ir 6 -Mo 7	10.2
LP Ir 16	-> LP* Mo 7	115.9
LP Ir 16	-> BD* Mo 7 -Ir 16	62.0
LP Ir 6	-> LP* Ir 16	114.1
LP Ir 16	-> LP* Ir 6	17.7
BD Ir 6 -Mo 7	-> LP* Ir 16	197.6
BD Ir 6 -Mo 7	-> BD* Mo 7 -Ir 16	112.5
BD Mo 7 -Ir 16	-> LP* Ir 6	171.9

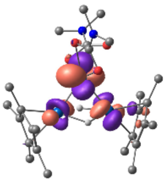

LUMO (-1.22 eV)

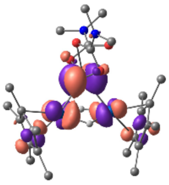

HOMO (-4.07 eV)

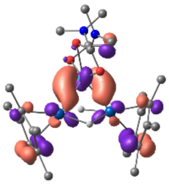

HOMO-1 (-5.02 eV)

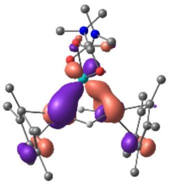

HOMO-2 (-5.29 eV)

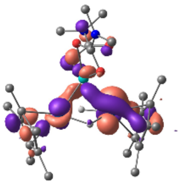

HOMO-3 (-5.68 eV)

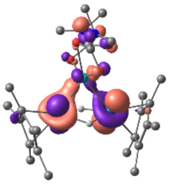

HOMO-5 (-6.06 eV)

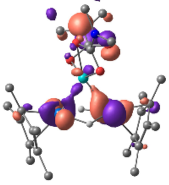

HOMO-6 (-6.28 eV)

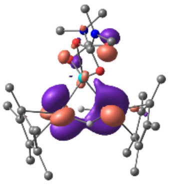

HOMO-8 (-6.55 eV)

Figure S 47: Computational data for complex 4.

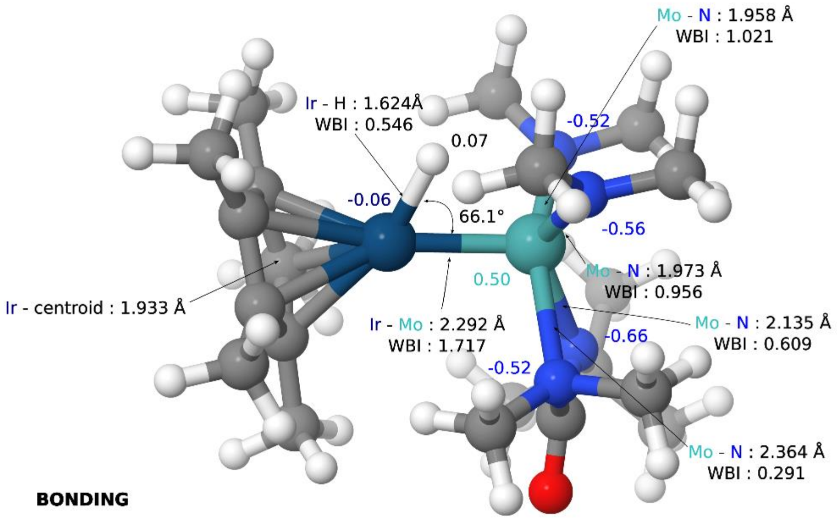

BONDING

(1.80264) BD (1)Ir 1 -Mo 2

(48.23%) 0.6945*Ir 1 s(26.02%)p 0.18(4.68%)d 2.66(69.27%) f 0.00(0.03%)

(51.77%) 0.7195*Mo 2 s(32.87%)p 0.23(7.42%)d 1.81(59.58%) f 0.00(0.13%)

(1.89080) BD (2)Ir 1 -Mo 2

(55.55%) 0.7453*Ir 1 s(1.34%)p 0.79(1.06%)d72.96(97.59%) f 0.01(0.02%)

(44.45%) 0.6667*Mo 2 s(1.09%)p12.38(13.48%)d78.28(85.21%) f 0.21(0.23%)

(1.67948) BD (3)Ir 1 -Mo 2

(81.61%) 0.9034*Ir 1 s(9.82%)p 0.33(3.22%)d 8.85(86.95%) f 0.00(0.02%)

(18.39%) 0.4288*Mo 2 s(1.63%)p20.73(33.73%)d39.56(64.37%) f 0.17(0.27%)

(1.73869) BD (1)Ir 1 - H 71

(57.81%) 0.7603*Ir 1 s(43.75%)p 0.21(9.31%)d 1.07(46.91%) f 0.00(0.03%)

(42.19%) 0.6496* H 71 s(99.82%)p 0.00(0.18%)

Second Order Perturbation Theory Analysis

LP Ir 1	-> LP* Mo 2	179.2
LP Ir 1	-> BD* Ir 1 -Mo 2	283.2
BD Ir 1 - H 71	-> LP* Mo 2	2734.67
BD Ir 1 - H 71	-> BD* Ir 1 -Mo 2	2964.53

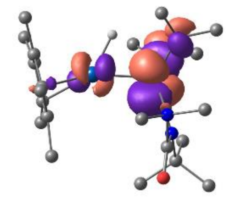

LUMO (-1.29 eV)

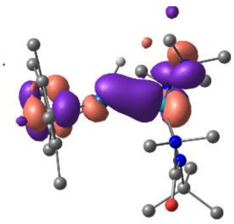

HOMO (-4.97 eV)

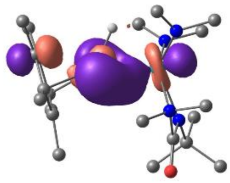

HOMO-1 (-5.16 eV)

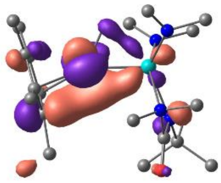

HOMO-3 (-5.72 eV)

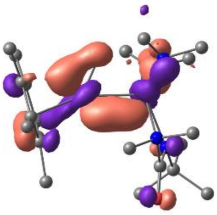

HOMO-8 (-7.36 eV)

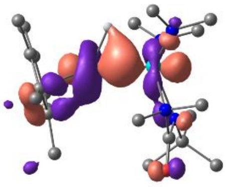

HOMO-9 (-7.51 eV)

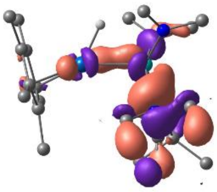

HOMO-11 (-7.76 eV)

Figure S 48: Computational data for complex 5.

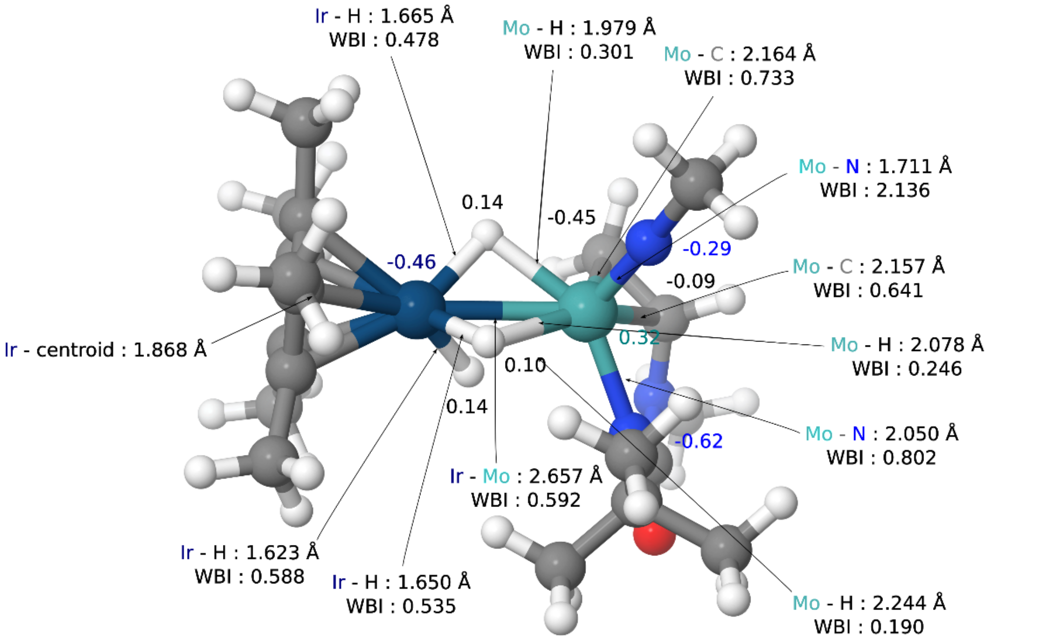

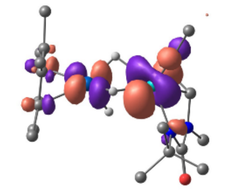

LUMO (-1.54 eV)

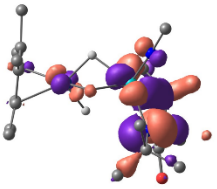

HOMO (-5.27 eV)

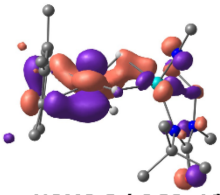

HOMO-3 (-6.32 eV)

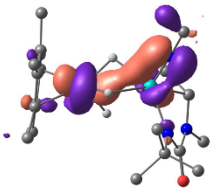

HOMO-6 (-7.32 eV)

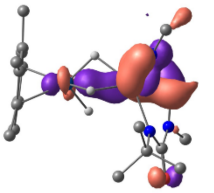

HOMO-11 (-8.10 eV)

BONDING

(1.75512) BD (1)Ir 6 -Mo 7

(49.13%) 0.7009*Ir 6 s(38.03%) p 0.16(6.06%) d 1.47(55.89%) f 0.00(0.03%)

(50.87%) 0.7132*Mo 7 s(32.26%) p 0.54(17.53%) d 1.55(50.13%) f 0.00(0.07%)

(1.87579) BD (2)Ir 6 -Mo 7

(55.25%) 0.7433*Ir 6 s(0.01%) p 1.00(1.58%) d 62.40(98.40%) f 0.01(0.01%)

(44.75%) 0.6689*Mo 7 s(0.00%) p 1.00(18.08%) d 4.53(81.85%) f 0.00(0.07%)

(1.65990) BD (1)Ir 6 - H 55

(47.66%) 0.6904*Ir 6 s(42.97%)p 0.26(11.31%)d 1.06(45.68%) f 0.00(0.03%)

(52.34%) 0.7234* H 55 s(99.88%)p 0.00(0.12%)

Second Order Perturbation Theory Analysis

LP Ir 1 -> LP* Mo 2 8.11

LP H 59 -> LP* Ir 1 145.9

BD Ir 1 - H 60 -> LP*(1)Mo 2 1909.8

BD Ir 1 - H 61 -> LP*(1)Mo 2 2674.6

BD Ir 1 - H 61 -> BD* Ir 1 - H 60 822.11

BD Ir 1 - H 60 -> BD* Ir 1 - H 61 744.01

Figure S 49: Computational data for complex 6.

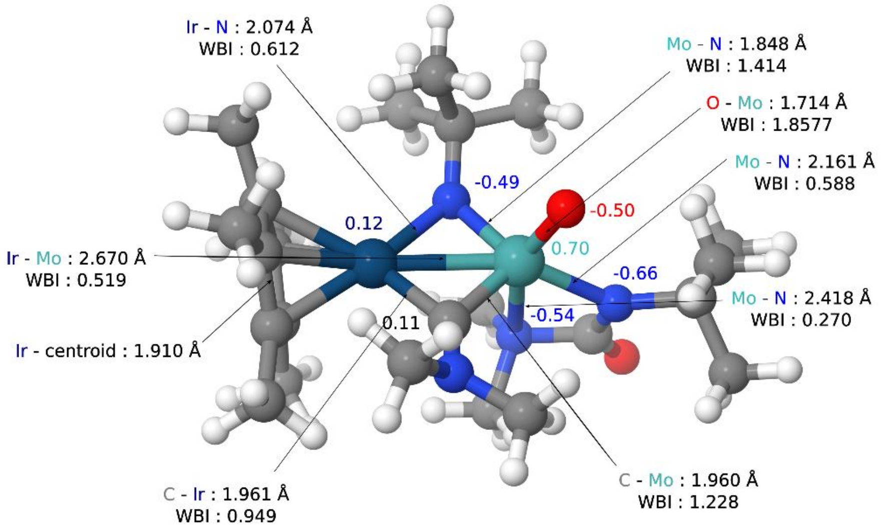

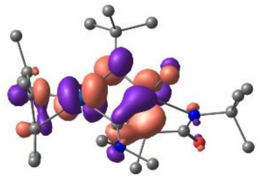

LUMO (-1.77 eV)

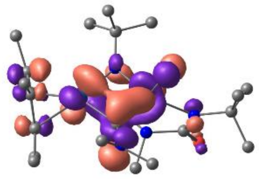

HOMO (-4.55 eV)

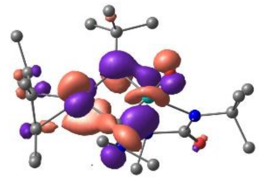

HOMO-1 (-5.55 eV)

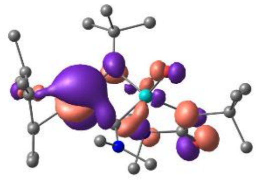

HOMO-2 (-6.10 eV)

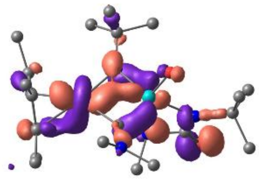

HOMO-7 (-7.30 eV)

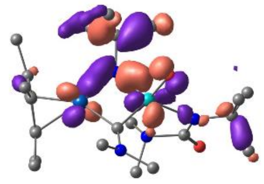

HOMO-12 (-8.32 eV)

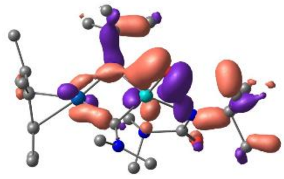

HOMO-13 (-8.55 eV)

Second Order Perturbation Theory Analysis

BD Ir 1 - N 7	-> LP* Mo 2	828.3
BD Ir 1 - C 44	-> LP*)Mo 2	1080.8
BD Mo 2 - O 3	-> LP*(5)Ir 1	45.0
BD Mo 2 - N 6	-> LP*(5)Ir 1	12.9
BD Mo 2 - N 7	-> LP*(5)Ir 1	68.3
BD Mo 2 - C 44	-> LP*(5)Ir 1	133.7
BD Mo 2 - C 44	-> LP*(6)Ir 1	4.7
LP Ir 1	-> LP* Mo 2	793.8

	Int 1	Compd 4	Int 2	Int 3	Int 3’	Int 3’’	Int 4	Int 5
Distances in Å and Angles in °								
d(Ir - Mo)	2,299	2,285	2,292	2,285	2,244	2,313	2,242	2,210
d(Ir - H)	1,644	1,621	1,624	1,613	-	-	-	-
d(Mo - H)	2,214	2,212	2,200	2,285	-	-	-	-
d(Ir - Cp)	1,917	1,936	1,933	1,928	1,931	1,956	1,954	1,951
Cp-Ir-Mo	161,3	166,1	166,1	168,1	179,4	166,3	173,9	169,4
Wiberg Bond Indices								
Ir - Mo	1,688	1,735	1,719	1,678	2,112	1,721	2,107	2,413
Ir - H	0,571	0,548	0,543	0,542	-	-	-	-
Mo - H	0,296	0,319	0,321	0,310	-	-	-	-
Natural Charges								
Ir	-0,12	-0,05	-0,06	-0,01	-0,02	-0,01	-0,03	-0,02
Mo	0,58	0,51	0,50	0,59	0,22	0,44	0,26	0,49
H	0,02	0,08	0,07	0,09	-	-	-	-
QTAIM – Ir – Mo Bond								
Density of all electrons	0.13817868	0.14178894	0.13983436	0.14149116	0.15117225	0.13505756	0.15170664	0.16198112
Laplacian of electron density	0.22839847	0.25005742	0.24508059	0.26087553	0.34241592	0.23979208	0.34196980	0.32173476
Lagrangian kinetic energy G(r)	0.12375363	0.13179036	0.12895682	0.13410578	0.16223080	0.12354756	0.16248950	0.16685695
Potential energy density V(r)	-0.1915681	-0.2023856	-0.19790791	-0.20434828	-0.24065786	-0.18842817	-0.24132236	-0.25522421
Energy density E(r) or H(r)	-0.06781438	-0.07059529	-0.06895109	-0.07024250	-0.07842706	-0.06488061	-0.07883286	-0.08836726
Ellipticity of electron density	0.216291	0.217052	0.226151	0.218064	0.017855	0.275401	0.015123	0.051861
Type	(3,-1)	(3,-1)	(3,-1)	(3,-1)	(3,-1)	(3,-1)	(3,-1)	(3,-1)

Table S3 – Bond distances, angles, Wiberg Bond Indices, Natural Charges and QTAIM results for the Ir – Mo bond of intermediates and complexes of Scheme 5.

	Int 5	Int 6	Int 7	Int 8	Int 8'	Int 9	Int 9'	Int 9''	Compd 5	Compd 5'
Distances in Å and Angles in °										
d(Ir - Mo)	2,210	2,315	2,434	2,628	2,502	2,592	2,590	2,456	2,639	2,657
d(Ir - H)	-	1,670	1,650 / 1,636	1,689 / 1,633 / 1,626	1,640 / 1,653	1,689 / 1,676 / 1,628	1,670 / 1,754 / 1,605	1,635 / 1,679	1,650 / 1,657 / 1,625	1,650 / 1,665 / 1,623
d(Mo - H)	-	2,051	2,061 / 2,238	1,897 / 2,135 / 2,232	2,139 / 2,013	1,917 / 1,955 / 2,263	2,060 / 1,790 / 2,393	2,230 / 1,988	2,029 / 2,016 / 2,239	2,078 / 1,979 / 2,244
d(Ir - Cp)	1,951	1,897	1,891	1,874	1,870	1,872	1,875	1,880	1,870	1,868
Cp-Ir-Mo	169,4	164,8	159,4	170,9	165,7	169,1	164,4	158,5	174,3	174,4
Wiberg Bond Indices										
Ir - Mo	2,413	1,625	1,143	0,618	0,984	0,688	0,713	1,070	0,618	0,592
Ir - H	-	0,509	0,520 / 0,588	0,445 / 0,542 / 0,601	0,529 / 0,490	0,471 / 0,480 / 0,609	0,541 / 0,385 / 0,610	0,482 / 0,577	0,510 / 0,594 / 0,506	0,534 / 0,478 / 0,588
Mo - H	-	0,375	0,289 / 0,214	0,344 / 0,542 / 0,179	0,283 / 0,302	0,323 / 0,324 / 0,172	0,249 / 0,442 / 0,156	0,332 / 0,236	0,274 / 0,184 / 0,269	0,246 / 0,301 / 0,190
Natural Charges										
Ir	-0,02	-0,07	-0,30	-0,48	-0,20	-0,46	-0,44	-0,25	-0,47	-0,46
Mo	0,49	0,36	0,34	0,13	0,12	0,25	0,36	0,35	0,30	0,32
H	-	0,02	0,10 / 0,07	0,11 / 0,15 / 0,17	0,08 / 0,08	0,14 / 0,12 / 0,13	0,10 / 0,16 / 0,11	0,08 / 0,07	0,13 / 0,12 / 0,14	0,10 / 0,14 / 0,14
QTAIM – Ir – Mo Bond										
Density of all electrons	0.16198112	0.13186215	0.10647817	0.07112934	0.09156807	0.07588834	0.07913922	0.10365319	0.06708042	0.06552125
Laplacian of electron density	0.32173476	0.23577310	0.13870597	0.13642017	0.13599296	0.14145584	0.13029446	0.12323384	0.14540137	0.14231628
Lagrangian kinetic energy G(r)	0.16685695	0.12065617	0.07776420	0.05362804	0.06738457	0.05802710	0.05692818	0.05692818	0.05345017	0.05174926
Potential energy density V(r)	-0.2552242	-0.1833743	-0.1213637	-0.0732660	-0.1010581	-0.0808309	-0.0814581	-0.1136806	-0.0706443	-0.0680068
Energy density E(r) or H(r)	-0.0883672	-0.0627181	-0.0435995	-0.0196380	-0.0336735	-0.0228038	-0.0245299	-0.0416519	-0.0171941	-0.0162576
Ellipticity of electron density	0.051861	0.199350	0.025125	0.987941	0.046144	0.416995	0.906974	0.022598	0.537625	1.000349
Type	(3,-1)	(3,-1)	(3,-1)	(3,-1)	(3,-1)	(3,-1)	(3,-1)	(3,-1)	(3,-1)	(3,-1)

Table S4 – Bond distances, angles, Wiberg Bond Indices, Natural Charges and QTAIM results for the Ir – Mo bond of intermediates and complexes of Scheme 6.

	Int 5	Int 10	Int 11	Int 12	Int 13	Int 13’	Complex 6
Distances in Å and Angles in °							
d(Ir - Mo)	2,210	2,210	2,235	2,219	2,312	2,374	2,670
d(Ir - Cp)	1,951	1,955	1,934	1,938	1,933	1,995	1,910
Cp-Ir-Mo	169,4	168,2	178,4	175,6	164,7	154,0	174,0
Wiberg Bond Indices							
Ir - Mo	2,413	2,423	2,182	2,274	1,579	1,444	0,519
Natural Charges							
Ir	-0,02	-0,02	-0,1	0,04	0,12	-0,02	0,12
Mo	0,49	0,50	0,27	0,45	0,39	0,77	0,70
QTAIM – Ir – Mo Bond							
Density of all electrons	0.16198112	0.16230043	0.15376372	0.15869890	0.13267179	0.12172361	0.06086824
Laplacian of electron density	0.32173476	0.32074537	0.35299537	0.35155026	0.21439951	0.16626852	0.16445957
Lagrangian kinetic energy G(r)	0.16685695	0.16688538	0.16702128	0.17118673	0.11612116	0.09600807	0.051748673
Potential energy density V(r)	-0.25522421	-0.25553658	-0.24771216	-0.25649923	-0.17953340	-0.15105300	-0.06246698
Energy density E(r) or H(r)	-0.08836726	-0.08865120	-0.08069088	-0.08531249	-0.06341224	-0.0550449	-0.01071831
Ellipticity of electron density	0.051861	0.051158	0.014444	0.027835	0.233211	0.276613	-2.208075
Type	(3,-1)	(3,-1)	(3,-1)	(3,-1)	(3,-1)	(3,-1)	(3,+1)

Table S5 – Bond distances, angles, Wiberg Bond Indices, Natural Charges and QTAIM results for the Ir – Mo bond of intermediates and complexes of Scheme 7.

NBO Analysis of the Ir – Mo Bond for all the intermediates and final complexes.

- **Int 1 :**

Bond Orbitals
(1.75459) BD (1)Ir 6 -Mo 7
(49.35%) 0.7025*Ir 6 s(38.02%)p 0.16(6.00%)d 1.47(55.96%)f 0.00(0.03%)
(50.65%) 0.7117*Mo 7 s(32.39%)p 0.54(17.39%)d 1.55(50.15%)f 0.00(0.07%)
(1.87474) BD (2)Ir 6 -Mo 7
(55.57%) 0.7455*Ir 6 s(0.00%)p 1.00(1.55%)d63.30(98.43%)f 0.01(0.01%)
(44.43%) 0.6666*Mo 7 s(0.00%)p 1.00(18.40%)d 4.43(81.53%)f 0.00(0.07%)

Second Order Perturbation Analysis :
LP Ir → LP*Mo 185.12 kcal/mol

- **Complex 4 :**

Bond Orbitals
(1.79588) BD (1)Ir 6 -Mo 7
(47.71%) 0.6908*Ir 6 s(27.64%)p 0.17(4.76%)d 2.44(67.57%)f 0.00(0.03%)
(52.29%) 0.7231*Mo 7 s(34.82%)p 0.22(7.56%)d 1.65(57.49%)f 0.00(0.12%)
(1.89319) BD (2)Ir 6 -Mo 7
57.56%) 0.7587*Ir 6 s(0.04%)p25.83(0.91%)d99.99(99.04%)f 0.41(0.01%)
(42.44%) 0.6514*Mo 7 s(0.12%)p99.99(13.54%)d99.99(86.13%)f 1.66(0.20%)
(1.69979) BD (3)Ir 6 -Mo 7
(79.78%) 0.8932*Ir 6 s(9.30%)p 0.33(3.05%)d 9.42(87.63%)f 0.00(0.02%)
(20.22%) 0.4496*Mo 7 s(2.61%)p10.92(28.50%)d26.30(68.66%)f 0.09(0.23%)

Second Order Perturbation Analysis :
LP (1)Ir → LP*(1)Mo 97.88 kcal/mol
LP (2)Ir → LP*(1)Mo 78.55 kcal/mol

- **Int 2 :**

Bond Orbitals
(1.80415) BD (1)Ir 6 -Mo 7
(48.48%) 0.6963*Ir 6 s(25.87%)p 0.18(4.63%)d 2.69(69.47%)
(51.52%) 0.7178*Mo 7 s(32.62%)p 0.23(7.59%)d 1.83(59.66%)f 0.00(0.13%)
(1.89089) BD (2)Ir 6 -Mo 7
(55.76%) 0.7467*Ir 6 s(1.24%)p 0.84(1.04%)d78.54(97.70%)f 0.01(0.02%)
(44.24%) 0.6652*Mo 7 s(1.02%)p13.27(13.52%)d83.68(85.23%)f 0.23(0.24%)
(1.67489) BD (3)Ir 6 -Mo 7
(81.59%) 0.9033*Ir 6 s(10.07%)p 0.32(3.24%)d 8.61(86.68%)f 0.00(0.02%)
(18.41%) 0.4290*Mo 7 s(1.61%)p21.07(33.95%)d39.81(64.16%)f 0.17(0.28%)

Second Order Perturbation Analysis :
LP (1)Ir → LP*(1)Mo 121.52 kcal/mol
LP (2)Ir → LP*(1)Mo 56.57 kcal/mol

- **Int 3 :**

Bond Orbitals
(1.80549) BD (1)Ir 6 -Mo 7
(51.93%) 0.7206*Ir 6 s(18.99%)p 0.22(4.26%)d 4.04(76.72%)f 0.00(0.03%)
(48.07%) 0.6933*Mo 7 s(31.66%)p 0.33(10.42%)d 1.83(57.86%)f 0.00(0.07%)
(1.64428) BD (2)Ir 6 -Mo 7
(76.65%) 0.8755*Ir 6 s(17.11%)p 0.26(4.44%)d 4.58(78.43%)f 0.00(0.02%)
(23.35%) 0.4832*Mo 7 s(7.19%)p 3.49(25.09%)d 9.37(67.44%)f 0.04(0.27%)
(1.85080) BD (3)Ir 6 -Mo 7
(55.04%) 0.7419*Ir 6 s(1.74%)p 0.37(0.64%)d56.16(97.61%)f 0.01(0.02%)
(44.96%) 0.6705*Mo 7 s(3.45%)p 4.85(16.75%)d23.08(79.74%)f 0.02(0.06%)

Second Order Perturbation Analysis :
LP Ir → LP*Mo 133.30 kcal/mol

- **Int 3' :**

Bond Orbitals
(1.84220) BD (1)Ir 6 -Mo 7
(51.22%) 0.7157*Ir 6 s(10.74%)p 0.13(1.44%)d 8.18(87.80%)f 0.00(0.02%)
(48.78%) 0.6985*Mo 7 s(8.93%)p 1.93(17.27%)d 8.27(73.77%)f 0.00(0.03%)
(1.82259) BD (2)Ir 6 -Mo 7
(55.03%) 0.7418*Ir 6 s(9.18%)p 0.17(1.56%)d 9.73(89.25%)f 0.00(0.02%)
(44.97%) 0.6706*Mo 7 s(7.31%)p 2.69(19.62%)d 9.99(73.01%)f 0.01(0.06%)
(1.74481) BD (3)Ir 6 -Mo 7
(39.68%) 0.6299*Ir 6 s(61.61%)p 0.11(6.61%)d 0.52(31.74%)f 0.00(0.04%)
(60.32%) 0.7767*Mo 7 s(43.47%)p 0.33(14.47%)d 0.97(42.02%)f 0.00(0.04%)
Second Order Perturbation Analysis :
LP (1)Ir → LP* Mo 15.75 kcal/mol
LP (2)Ir → LP* Mo 326.78 kcal/mol

- **Int 3" :**

Bond Orbitals
(1.88795) BD (1)Ir 6 -Mo 7
(30.97%) 0.5566*Ir 6 s(57.70%)p 0.07(4.10%)d 0.66(38.14%)f 0.00(0.06%)
(69.03%) 0.8308*Mo 7 s(17.93%)p 0.41(7.43%)d 4.16(74.62%)f 0.00(0.02%)
(1.93331) BD (2)Ir 6 -Mo 7
(48.39%) 0.6956*Ir 6 s(2.34%)p 0.25(0.59%)d41.52(97.05%)f 0.01(0.02%)
(51.61%) 0.7184*Mo 7 s(0.13%)p24.22(3.26%)d99.99(96.58%)f 0.19(0.03%)

Second Order Perturbation Analysis :
LP (1)Ir → LP*Mo ~285 kcal/mol
LP (2)Ir → LP*Mo ~300 kcal/mol
LP (3)Ir → LP*Mo ~94 kcal/mol

- **Int 4 :**

Bond Orbitals
(1.82401) BD (1)Ir 6 -Mo 7
(50.81%) 0.7128*Ir 6 s(15.28%)p 0.13(1.92%)d 5.42(82.78%)f 0.00(0.02%)
(49.19%) 0.7013*Mo 7 s(14.28%)p 1.11(15.82%)d 4.89(69.84%)f 0.00(0.06%)
(1.85703) BD (2)Ir 6 -Mo 7
(58.34%) 0.7638*Ir 6 s(3.33%)p 0.12(0.39%)d28.93(96.27%)f 0.00(0.01%)
(41.66%) 0.6454*Mo 7 s(1.84%)p12.14(22.33%)d41.17(75.76%)f 0.03(0.06%)
(1.71393) BD (3)Ir 6 -Mo 7
(39.02%) 0.6247*Ir 6 s(63.51%)p 0.10(6.16%)d 0.48(30.30%)f 0.00(0.03%)
(60.98%) 0.7809*Mo 7 s(45.56%)p 0.29(13.11%)d 0.91(41.31%)f 0.00(0.02%)

Second Order Perturbation Analysis :
LP (1)Ir → LP* Mo 2.85 kcal/mol
LP (2)Ir → LP* Mo 359.24 kcal/mol
LP (3)Ir → LP* Mo 203.78 kcal/mol

- **Int 5 :**

Bond Orbitals
(1.74612) BD (1)Ir 6 -Mo 7
(47.73%) 0.6908*Ir 6 s(44.01%)p 0.14(6.23%)d 1.13(49.60%)f 0.00(0.17%)
(52.27%) 0.7230*Mo 7 s(39.78%)p 0.21(8.20%)d 1.30(51.89%)f 0.00(0.13%)
(1.66405) BD (2)Ir 6 -Mo 7
(36.02%) 0.6001*Ir 6 s(3.22%)p11.37(36.65%)d18.45(59.47%)f 0.20(0.66%)
(63.98%) 0.7999*Mo 7 s(7.35%)p 0.48(3.52%)d12.12(89.09%)f 0.01(0.04%)
(1.86680) BD (3)Ir 6 -Mo 7
(41.78%) 0.6464*Ir 6 s(30.63%)p 0.38(11.64%)d 1.88(57.63%)f 0.00(0.09%)
(58.22%) 0.7630*Mo 7 s(15.90%)p 0.25(3.99%)d 5.04(80.08%)f 0.00(0.03%)

Second Order Perturbation Analysis :
LP (2)Ir → LP* Mo ~335 kcal/mol
LP (3)Ir → LP* Mo ~840 kcal/mol

- **Int 6 :**

Bond Orbitals
(1.86461) BD (1)Ir 6 -Mo 7
(54.46%) 0.7379*Ir 6 s(10.48%)p 0.20(2.07%)d 8.34(87.42%)f 0.00(0.02%)
(45.54%) 0.6749*Mo 7 s(2.02%)p 2.84(5.74%)d45.63(92.21%)f 0.02(0.03%)
(1.93175) BD (2)Ir 6 -Mo 7
(50.69%) 0.7120*Ir 6 s(1.43%)p 1.40(2.01%)d67.48(96.55%)f 0.01(0.01%)
(49.31%) 0.7022*Mo 7 s(0.04%)p45.81(1.77%)d99.99(98.17%)f 0.62(0.02%)

Second Order Perturbation Analysis :
LP (1)Ir → LP* Mo ~356 kcal/mol
LP (2)Ir → LP* Mo ~27 kcal/mol
LP (3)Ir → LP* Mo ~36 kcal/mol

- **Int 7 :**

Bond Orbitals
(1.83668) BD (1)Ir 6 -Mo 7
(51.90%) 0.7204*Ir 6 s(17.39%)p 0.37(6.39%)d 4.38(76.21%)f 0.00(0.01%)
(48.10%) 0.6935*Mo 7 s(22.86%)p 0.26(6.01%)d 3.11(71.05%)f 0.00(0.08%)

Second Order Perturbation Analysis :
LP (1)Ir → LP* Mo ~26 kcal/mol
LP (2)Ir → LP* Mo ~34 kcal/mol

- **Int 8 :**

Bond Orbitals
-
Second Order Perturbation Analysis :
LP Ir → LP* Mo <10 kcal/mol

- **Int 8' :**

Bond Orbitals
(1.83600) BD (1)Ir 6 -Mo 7
(50.53%) 0.7108*Ir 6 s(11.54%)p 0.24(2.73%)d 7.43(85.72%)f 0.00(0.01%)
(49.47%) 0.7034*Mo 7 s(7.74%)p 0.97(7.53%)d10.95(84.71%)f 0.00(0.02%)

Second Order Perturbation Analysis :
LP (1)Ir → LP* Mo 7 ~20 kcal/mol
LP (2)Ir → LP* Mo 7 ~8 kcal/mol
LP (3)Ir → LP* Mo 7 ~15 kcal/mol

- **Int 9 :**

Bond Orbitals
-
Second Order Perturbation Analysis :
LP Ir → LP* Mo <5 kcal/mol
LP Mo → LP*Ir 8.99 kcal/mol

- **Int 9' :**

Bond Orbitals
-
Second Order Perturbation Analysis :
LP (1)Ir → LP* Mo ~15 kcal/mol
LP (2)Ir → LP* Mo <5 kcal/mol
LP Mo → LP* Ir ~50 kcal/mol

- **Int 9" :**

Bond Orbitals
(1.84514) BD (1)Ir 6 -Mo 7
(45.08%) 0.6714*Ir 6 s(17.58%)p 0.56(9.76%)d 4.13(72.61%)f 0.00(0.05%)
(54.92%) 0.7411*Mo 7 s(8.03%)p 0.71(5.70%)d10.73(86.23%)f 0.00(0.03%)

Second Order Perturbation Analysis :
LP (1)Ir → LP*(1)Mo 7 ~45 kcal/mol

- **Complex 5 :**

Bond Orbitals
-
Second Order Perturbation Analysis :
LP Ir → LP* Mo <5 kcal/mol

- **Complex 5':**

Bond Orbitals
-
Second Order Perturbation Analysis :
LP Ir → LP* Mo <5 kcal/mol

- **Int 10 :**

Bond Orbitals
(1.91393) BD (1)Ir 6 -Mo 7
(49.08%) 0.7005*Ir 6 s(34.27%)p 0.13(4.51%)d 1.79(61.19%)f 0.00(0.03%)
(50.92%) 0.7136*Mo 7 s(26.39%)p 0.22(5.93%)d 2.56(67.63%)f 0.00(0.05%)
(1.89287) BD (2)Ir 6 -Mo 7
(51.27%) 0.7160*Ir 6 s(11.50%)p 0.16(1.86%)d 7.53(86.61%)f 0.00(0.03%)
(48.73%) 0.6981*Mo 7 s(13.03%)p 0.35(4.54%)d 6.32(82.38%)f 0.00(0.04%)
(1.89518) BD (3)Ir 6 -Mo 7
(42.83%) 0.6545*Ir 6 s(33.61%)p 0.16(5.40%)d 1.81(60.97%)f 0.00(0.03%)
(57.17%) 0.7561*Mo 7 s(16.22%)p 0.14(2.19%)d 5.03(81.55%)f 0.00(0.03%)

Second Order Perturbation Analysis :
LP (1)Ir → LP* Mo 7 ~285 kcal/mol
LP (2)Ir → LP* Mo 7 ~18 kcal/mol
LP (3)Ir → LP* Mo 7 ~1071 kcal/mol

- **Int 11 :**

Bond Orbitals
(1.90388) BD (1)Ir 6 -Mo 7
(54.05%) 0.7352*Ir 6 s(0.06%)p11.90(0.67%)d99.99(99.26%)f 0.30(0.02%)
(45.95%) 0.6778*Mo 7 s(0.39%)p44.56(17.45%)d99.99(82.12%)f 0.10(0.04%)
(1.65437) BD (2)Ir 6 -Mo 7
(36.94%) 0.6078*Ir 6 s(78.27%)p 0.10(7.62%)d 0.18(14.05%)f 0.00(0.05%)
(63.06%) 0.7941*Mo 7 s(64.16%)p 0.13(8.23%)d 0.43(27.59%)f 0.00(0.02%)
(1.84243) BD (3)Ir 6 -Mo 7
(57.65%) 0.7593*Ir 6 s(3.40%)p 0.30(1.01%)d28.07(95.56%)f 0.01(0.02%)
(42.35%) 0.6508*Mo 7 s(1.48%)p15.64(23.22%)d50.67(75.21%)f 0.06(0.08%)

Second Order Perturbation Analysis :
LP (1)Ir → LP* Mo 7 84.25 kcal/mol
LP (2)Ir → LP* Mo 7 304.92 kcal/mol
LP (3)Ir → LP* Mo 7 213.79 kcal/mol

- **Int 12 :**

Bond Orbitals
(1.93529) BD (1)Ir 6 -Mo 7
(49.80%) 0.7057*Ir 6 s(10.51%)p 0.15(1.56%)d 8.37(87.91%)f 0.00(0.02%)
(50.20%) 0.7085*Mo 7 s(11.00%)p 0.33(3.61%)d 7.76(85.34%)f 0.00(0.05%)
(1.83196) BD (2)Ir 6 -Mo 7
(55.77%) 0.7468*Ir 6 s(5.78%)p 0.20(1.16%)d16.11(93.04%)f 0.00(0.02%)
(44.23%) 0.6651*Mo 7 s(4.88%)p 3.88(18.96%)d15.59(76.14%)f 0.00(0.02%)
1.74617) BD (3)Ir 6 -Mo 7
(38.08%) 0.6171*Ir 6 s(63.94%)p 0.12(7.44%)d 0.45(28.58%)f 0.00(0.04%)
(61.92%) 0.7869*Mo 7 s(46.73%)p 0.23(10.84%)d 0.91(42.41%)f 0.00(0.01%)

Second Order Perturbation Analysis :
LP (1)Ir → LP* Mo ~290 kcal/mol
LP (3)Ir → LP* Mo ~400 kcal/mol

- **Int 13 :**

Bond Orbitals
(1.53554) BD (1)Ir 6 -Mo 7
(33.48%) 0.5786*Ir 6 s(32.88%)p 0.70(23.17%)d 1.33(43.63%)f 0.01(0.32%)
(66.52%) 0.8156*Mo 7 s(15.50%)p 0.59(9.12%)d 4.86(75.35%)f 0.00(0.03%)
(1.89715) BD (2)Ir 6 -Mo 7
(53.69%) 0.7327*Ir 6 s(1.04%)p 1.39(1.45%)d93.81(97.50%)f 0.01(0.01%)
(46.31%) 0.6805*Mo 7 s(2.47%)p 1.82(4.50%)d37.67(93.00%)f 0.01(0.03%)

Second Order Perturbation Analysis :
LP (1)Ir → LP* Mo ~450 kcal/mol
LP (2)Ir → LP* Mo <5 kcal/mol
LP (3)Ir → LP* Mo 7 ~80 kcal/mol

- **Int 13' :**

Bond Orbitals
(1.80257) BD (1)Ir 6 -Mo 7
(54.34%) 0.7371*Ir 6 s(32.98%)p 0.16(5.28%)d 1.87(61.72%)f 0.00(0.02%)
(45.66%) 0.6757*Mo 7 s(5.00%)p 3.00(15.00%)d16.00(79.96%)f 0.01(0.04%)
(1.92385) BD (2)Ir 6 -Mo 7
(38.54%) 0.6208*Ir 6 s(14.99%)p 0.19(2.80%)d 5.48(82.18%)f 0.00(0.02%)
(61.46%) 0.7840*Mo 7 s(3.09%)p 0.71(2.21%)d30.68(94.69%)f 0.00(0.01%)

Second Order Perturbation Analysis :
LP (1)Ir → LP* Mo ~301 kcal/mol
LP (2)Ir → LP* Mo ~10 kcal/mol
LP (3)Ir → LP* Mo ~73 kcal/mol

- **Complex 6 :**

Bond Orbitals
-
Second Order Perturbation Analysis :
LP (1)Ir → LP* Mo 129.02 kcal/mol
LP (3)Ir → LP* Mo 103.57 kcal/mol
LP (4)Ir → LP* Mo 537.45 kcal/mol

Cartesian coordinates of all optimized structures:

3
CO2

C	0.09117	0.00000	1.21200
O	0.16203	0.00000	2.37702
O	0.02083	0.00000	0.04695

2
H2

H	0.00000	0.00000	0.02728
H	0.00000	0.00000	0.77272

10
HNMe2

N	0.00115	0.00195	-0.07128
H	-0.05152	-0.08872	0.93667
C	1.39253	-0.00723	-0.48369
C	-0.70259	1.20205	-0.48419
H	1.44877	-0.04174	-1.57687
H	1.97257	0.87343	-0.15118
H	1.88578	-0.90547	-0.10291
H	-0.76292	1.23202	-1.57731
H	-1.72638	1.18112	-0.10138
H	-0.22872	2.14478	-0.15384

16
tBuNCO

N	-0.04039	-0.13462	0.18583
C	0.20987	0.28473	1.28213
O	0.51353	0.79362	2.29300
C	-0.72073	-1.27259	-0.42929
C	-0.62859	-1.08466	-1.94450
C	-0.01485	-2.56516	-0.00826
C	-2.18550	-1.28333	0.01895
H	-1.12294	-1.91158	-2.46111
H	-1.10863	-0.14999	-2.24412
H	0.41594	-1.04972	-2.26303
H	-2.72291	-2.10566	-0.46200
H	-2.26057	-1.41319	1.10228
H	-2.67690	-0.34554	-0.25129
H	-0.48386	-3.42737	-0.49081
H	1.03861	-2.53871	-0.29735
H	-0.07241	-2.70589	1.07471

26
tBuNHCONMe2

C	1.35409	-0.05752	-0.57653
N	0.04353	0.13849	-0.19965
C	-0.73812	1.33397	-0.56804
H	-0.25981	-0.31330	0.64747
O	1.95508	0.70261	-1.32513
N	1.93952	-1.20403	-0.07323

C	-0.89323	1.40480	-2.09069
C	-2.11412	1.14770	0.07653
C	-0.08103	2.61343	-0.03262
H	-2.75979	1.99650	-0.16030
H	-2.59738	0.23706	-0.29001
H	-2.03567	1.08611	1.16830
H	-1.52221	2.25777	-2.36252
H	0.08016	1.51639	-2.56772
H	-1.36572	0.49289	-2.46685
H	-0.68238	3.48983	-0.29337
H	0.00646	2.57365	1.05804
H	0.91531	2.73042	-0.46000
C	3.36179	-1.39275	-0.26279
C	1.24084	-2.16905	0.74336
H	1.75754	-3.12933	0.67693
H	1.19849	-1.88979	1.80771
H	0.22274	-2.32422	0.37681
H	3.90231	-1.35263	0.69191
H	3.56408	-2.36044	-0.73611
H	3.72436	-0.59789	-0.91138

55

Complex 1

C	7.76677	5.26046	5.98147
C	7.14648	5.66028	7.20689
C	6.46219	4.51501	7.75631
C	6.63842	3.42206	6.84257
C	7.44563	3.88978	5.74196
Ir	5.45073	5.01005	5.84251
Mo	3.19942	5.23090	5.40732
N	2.80705	7.13249	5.30496
C	3.69260	8.27723	5.19186
C	7.29357	6.98731	7.87730
C	5.82979	4.44729	9.10994
C	6.21236	2.00671	7.06579
C	7.95072	3.04865	4.61468
C	8.61199	6.12961	5.10664
N	2.07804	4.46367	6.81470
C	1.78601	3.04651	6.91190
N	2.84056	4.24402	3.77119
C	1.45797	4.15684	3.32191
C	1.71959	5.18244	8.02243
C	3.75014	3.71312	2.77202
C	1.41932	7.52255	5.09624
H	2.24684	4.78640	8.90338
H	0.63911	5.09927	8.21875
H	1.97018	6.24079	7.92785
H	2.05264	2.53414	5.98547
H	0.71327	2.88287	7.09981
H	2.34192	2.57140	7.73490

H	3.61268	4.21673	1.80226
H	3.56277	2.64037	2.61559
H	4.77771	3.84512	3.10554
H	1.18489	3.11213	3.11212
H	1.29460	4.73396	2.39987
H	0.77308	4.52898	4.08824
H	1.26607	7.95426	4.09614
H	1.11583	8.27931	5.83455
H	0.74918	6.66621	5.20876
H	4.72550	7.95560	5.30732
H	3.46222	9.01666	5.97309
H	3.57246	8.77755	4.21816
H	7.43359	7.78969	7.15022
H	8.16208	6.99187	8.54874
H	6.41189	7.23144	8.47309
H	5.36439	5.39716	9.38137
H	6.57736	4.21054	9.87808
H	5.05660	3.67808	9.15072
H	5.32727	1.95484	7.70202
H	7.01022	1.43279	7.55438
H	5.97124	1.50865	6.12432
H	7.24374	2.25715	4.35687
H	8.90239	2.56949	4.87864
H	8.12087	3.64464	3.71552
H	8.55080	5.82341	4.06024
H	9.66627	6.08144	5.40591
H	8.30035	7.17489	5.16094
H	5.08256	5.79094	4.44057

55

Complex 1 - triplet

C	7.88467	4.96546	5.83812
C	7.14730	5.95333	6.60442
C	6.38523	5.26426	7.59490
C	6.58959	3.84376	7.39466
C	7.55557	3.68183	6.33033
Ir	5.57148	4.67611	5.61039
Mo	3.21713	5.07762	5.24629
N	2.61927	6.78417	5.97718
C	3.41051	7.99584	5.86648
C	7.34120	7.43218	6.49469
C	5.63578	5.88277	8.73097
C	6.11206	2.75653	8.30092
C	8.11542	2.37508	5.86851
C	8.85630	5.28592	4.74966
N	2.24318	3.73800	6.28007
C	2.06981	2.38660	5.78036
N	2.71322	4.97330	3.36828
C	1.32661	4.88793	2.95541
C	1.92620	3.83905	7.68940

C	3.62258	5.09839	2.24462
C	1.26518	7.06118	6.41662
H	2.54981	3.16229	8.29421
H	0.87534	3.56613	7.87626
H	2.08286	4.85765	8.04809
H	2.29864	2.33939	4.71378
H	1.03317	2.04577	5.92604
H	2.73396	1.67842	6.29908
H	3.42600	6.01622	1.66935
H	3.51278	4.24427	1.55853
H	4.65703	5.11890	2.59268
H	1.16196	4.01920	2.29934
H	1.01328	5.78614	2.40148
H	0.67242	4.78044	3.82450
H	0.74986	7.74687	5.72595
H	1.26007	7.53519	7.41076
H	0.68219	6.14012	6.47630
H	4.41448	7.75604	5.51122
H	3.50344	8.49491	6.84371
H	2.95162	8.71719	5.17156
H	7.44433	7.74668	5.45328
H	8.24858	7.74789	7.02575
H	6.50082	7.98033	6.92409
H	5.29390	6.88897	8.48310
H	6.27402	5.95594	9.62101
H	4.75647	5.29391	8.99853
H	5.16754	3.02289	8.77795
H	6.84474	2.56506	9.09636
H	5.95948	1.82127	7.75890
H	7.37614	1.57570	5.94922
H	8.98512	2.08607	6.47180
H	8.43569	2.42101	4.82587
H	9.03204	4.42588	4.10083
H	9.82511	5.58998	5.16678
H	8.49819	6.10627	4.12291
H	4.97258	3.53346	4.61251

71

Int 1

C	-0.53215	1.26416	-3.48327
C	-0.37005	2.51747	-2.81656
C	-1.07556	2.44873	-1.55910
C	-1.64894	1.13795	-1.44932
C	-1.29778	0.40217	-2.64069
Ir	0.56766	0.95694	-1.43771
Mo	2.03162	0.50495	0.27689
N	-2.79872	-2.67881	3.62771
C	-3.34086	-1.61340	3.52926
O	-3.75507	-0.51834	3.46715
C	0.29657	3.73345	-3.37243

C	-1.30190	3.59853	-0.62947
C	-2.59189	0.66603	-0.38929
C	-1.77197	-0.97253	-2.98331
C	0.00629	0.91658	-4.83407
N	1.32406	1.04545	2.01481
C	0.38047	0.24002	2.76816
N	3.63936	1.54753	-0.05719
C	4.70543	1.47803	0.93189
N	2.29871	-1.42167	0.31985
C	1.96226	-2.44513	-0.65278
C	1.38074	2.41531	2.48838
C	4.06865	2.26557	-1.24310
C	3.14022	-1.95999	1.37944
C	-3.10143	-4.09883	3.46234
C	-4.02782	-4.54074	4.59941
C	-3.76858	-4.32107	2.10179
C	-1.77182	-4.85231	3.53382
H	1.36101	-2.01181	-1.44978
H	1.38858	-3.25162	-0.17180
H	2.86908	-2.89793	-1.08391
H	4.36696	0.96538	1.83619
H	5.02794	2.48810	1.22484
H	5.58724	0.94819	0.54214
H	4.08954	-2.35177	0.98502
H	2.63001	-2.78585	1.89702
H	3.36589	-1.19427	2.12623
H	4.95574	1.79492	-1.69557
H	4.33970	3.29998	-0.98449
H	3.25972	2.28619	-1.97087
H	-1.09827	-4.51117	2.74407
H	-1.93639	-5.92627	3.41311
H	-1.28478	-4.68072	4.49681
H	-3.56564	-4.34358	5.56964
H	-4.23313	-5.61246	4.52553
H	-4.98024	-4.00537	4.55539
H	0.35409	-0.78062	2.38198
H	-0.63845	0.64967	2.71185
H	0.66656	0.20143	3.83042
H	-4.71589	-3.77815	2.03984
H	-3.97368	-5.38453	1.95016
H	-3.11855	-3.97723	1.29356
H	0.40344	2.91366	2.40492
H	2.10346	2.99220	1.90776
H	1.68261	2.44746	3.54668
H	-2.48243	-0.40486	-0.20655
H	-3.63238	0.84992	-0.68595
H	-2.42374	1.17715	0.55962
H	-1.07027	-1.48572	-3.64432
H	-2.74326	-0.93686	-3.49379

H	-1.89095	-1.58646	-2.08810
H	-1.54362	3.25539	0.37788
H	-2.13606	4.22135	-0.97745
H	-0.41795	4.23604	-0.55802
H	0.72038	4.35225	-2.57887
H	-0.41824	4.35190	-3.93082
H	1.10712	3.46916	-4.05486
H	0.93535	1.45054	-5.04456
H	-0.71159	1.17671	-5.62170
H	0.21663	-0.15167	-4.91836
H	1.98200	0.27522	-1.92447

71

TS Int - complex 4

C	0.43293	1.80409	6.35347
C	0.68448	2.99473	7.10904
C	-0.24495	3.03007	8.20796
C	-1.04361	1.84045	8.13940
C	-0.62371	1.08440	6.98814
Ir	1.09469	1.25316	8.51077
Mo	2.36910	0.66392	10.34399
N	0.39197	-0.36759	11.56130
C	-0.10289	0.70304	11.94842
O	-0.83424	1.54770	12.31071
C	1.63174	4.08823	6.73707
C	-0.46539	4.18246	9.13507
C	-2.24835	1.55409	8.97607
C	-1.25842	-0.17399	6.49236
C	1.14042	1.39891	5.10019
N	1.92523	1.65543	12.07649
C	2.24160	1.15538	13.40122
N	3.96768	1.72372	9.96453
C	4.98298	1.88547	10.99310
N	3.08412	-1.15650	10.21521
C	3.04458	-2.14542	9.15704
C	1.85597	3.10497	12.05980
C	4.43777	2.29895	8.72042
C	4.07468	-1.50594	11.21362
C	-0.35365	-1.66281	11.61425
C	-1.60231	-1.55673	12.49253
C	-0.73998	-2.05560	10.18932
C	0.60792	-2.69388	12.20588
H	2.26840	-1.90596	8.43324
H	2.84215	-3.14529	9.57259
H	4.00822	-2.20371	8.62570
H	4.65141	1.48870	11.95430
H	5.22340	2.94945	11.13893
H	5.91975	1.37690	10.71816
H	5.08207	-1.58715	10.77644
H	3.83880	-2.47204	11.68607

H	4.11526	-0.76115	12.01403
H	5.38884	1.83626	8.40938
H	4.62485	3.37850	8.83368
H	3.69410	2.14821	7.94065
H	1.51466	-2.74803	11.60285
H	0.13707	-3.68117	12.22907
H	0.88577	-2.42239	13.22809
H	-1.34377	-1.26961	13.51599
H	-2.10514	-2.52717	12.53236
H	-2.31263	-0.82517	12.09943
H	2.19716	0.06448	13.42075
H	1.52824	1.53931	14.14672
H	3.24645	1.46757	13.72910
H	-1.46953	-1.35315	9.78267
H	-1.18167	-3.05727	10.18262
H	0.13331	-2.04356	9.53653
H	1.02747	3.45010	12.69613
H	1.68157	3.46476	11.04582
H	2.78546	3.56124	12.43269
H	-2.44928	0.48303	9.03855
H	-3.13516	2.02620	8.53369
H	-2.13962	1.93562	9.99208
H	-0.56010	-0.76628	5.89731
H	-2.12762	0.04660	5.85940
H	-1.59982	-0.80208	7.31753
H	-0.80814	3.84947	10.11643
H	-1.22317	4.86523	8.72961
H	0.44953	4.76101	9.27978
H	2.03894	4.58247	7.62120
H	1.12456	4.85036	6.13171
H	2.47243	3.71028	6.15214
H	2.16883	1.76527	5.08301
H	0.63022	1.79987	4.21579
H	1.17872	0.31264	4.99660
H	2.21522	0.12366	8.17479

71
Complex4

C	-2.45256	0.72685	-1.62442
C	-1.75479	0.55325	-2.85580
C	-0.64957	1.45144	-2.87803
C	-0.68790	2.23144	-1.66369
C	-1.80814	1.78572	-0.88788
Ir	-0.29992	0.10989	-0.99992
Mo	0.98694	-0.85693	0.59727
N	2.61836	0.15350	0.34957
C	2.99056	1.17268	-0.61296
C	-2.11662	-0.42045	-3.93108
C	0.30155	1.65295	-4.01221
C	0.13892	3.44532	-1.38253

C	-2.34841	2.43489	0.34812
C	-3.73037	0.06002	-1.23138
N	1.71116	-2.58320	-0.06716
C	2.77925	-2.68792	-1.04173
N	0.42389	0.15668	2.47363
C	0.82144	1.30387	3.30931
C	1.96012	0.84225	4.21863
C	-0.75068	-0.34932	2.65782
O	-1.84173	-0.24170	3.11313
N	-0.54078	-1.75501	1.93325
C	-0.06150	-2.58795	3.03963
C	0.81712	-3.72178	-0.20805
C	-1.84479	-2.19740	1.47438
C	3.73655	-0.18715	1.21310
C	1.27884	2.43027	2.39014
C	-0.33314	1.80904	4.17768
H	0.02562	-3.70100	0.54063
H	1.37549	-4.66190	-0.08729
H	0.33557	-3.74524	-1.20035
H	3.45745	-0.96189	1.93152
H	4.10357	0.69029	1.76388
H	4.57448	-0.57525	0.61691
H	2.39580	-2.63143	-2.07387
H	3.29157	-3.65566	-0.93116
H	3.51307	-1.89355	-0.91585
H	3.84666	0.82904	-1.21283
H	3.29778	2.10414	-0.11387
H	2.14886	1.36719	-1.27365
H	1.61944	0.03952	4.87823
H	2.30942	1.67125	4.84189
H	2.80573	0.47207	3.63745
H	2.07907	2.09722	1.73173
H	1.63796	3.28108	2.97748
H	0.44800	2.76728	1.76555
H	-2.14721	-1.59406	0.61931
H	-2.59006	-2.10586	2.27410
H	-1.79598	-3.24704	1.16211
H	-1.18181	2.13471	3.57561
H	0.01942	2.66410	4.76232
H	-0.68680	1.04768	4.87441
H	-0.78886	-2.61897	3.86402
H	0.88545	-2.20410	3.42477
H	0.10282	-3.61806	2.70351
H	-3.78965	-0.96042	-1.61586
H	-4.59209	0.61272	-1.62691
H	-3.84038	0.01565	-0.14628
H	-1.23597	-0.76018	-4.47984
H	-2.80272	0.03625	-4.65484
H	-2.61022	-1.30394	-3.52099

H	-2.77974	1.70582	1.03626
H	-3.13037	3.16119	0.09114
H	-1.56881	2.97383	0.88951
H	0.27236	3.60660	-0.31166
H	-0.34617	4.33874	-1.79563
H	1.13015	3.37644	-1.83445
H	1.28525	1.97145	-3.66133
H	-0.06826	2.42166	-4.70284
H	0.44107	0.73402	-4.58496
H	0.07659	-1.31314	-1.63642

71

Complex4- triplet

Ir	1.24870	1.36111	8.37031
Mo	2.07312	0.56396	10.47161
N	4.00971	0.67788	10.81555
N	2.11202	-1.29602	9.68527
O	-0.38694	2.52054	12.65070
N	0.79765	0.56703	12.11430
N	1.77343	2.56004	11.70173
C	0.93411	-2.08630	9.39704
H	0.03796	-1.61792	9.80327
H	1.02296	-3.10247	9.81479
H	0.79271	-2.19241	8.31129
C	0.56446	1.89696	12.22769
C	-0.75532	1.07383	7.38929
C	4.69070	-0.33107	11.59929
H	3.99418	-1.11253	11.91403
H	5.14100	0.10580	12.50515
H	5.50605	-0.81474	11.03601
C	3.27320	-1.89307	9.05365
H	3.11696	-1.99289	7.96888
H	3.46060	-2.90269	9.45182
H	4.15743	-1.27929	9.20979
C	4.90860	1.71035	10.35039
H	5.65117	1.31097	9.64029
H	5.47147	2.15710	11.18647
H	4.35579	2.49973	9.83975
C	0.69168	-1.73798	12.90042
H	0.86873	-2.11246	11.89353
H	0.09519	-2.47601	13.44456
H	1.65978	-1.64765	13.40187
C	0.23737	0.98937	6.33734
C	-0.02874	-0.38929	12.89279
C	-0.15023	0.08232	14.35122
H	0.84193	0.21824	14.79256
H	-0.67655	-0.67803	14.93577
H	-0.69936	1.02047	14.42591
C	2.73930	2.72531	12.80763
H	2.93182	1.76075	13.27458

H	2.34555	3.42433	13.55616
H	3.67504	3.11822	12.40950
C	-0.69172	2.38698	7.94470
C	-1.42718	-0.51590	12.27639
H	-1.92976	0.45312	12.28831
H	-2.03286	-1.22986	12.84363
H	-1.36903	-0.86515	11.24261
C	1.49238	3.86361	11.09504
H	2.42867	4.28370	10.72335
H	1.04200	4.54619	11.82406
H	0.82396	3.71630	10.25091
C	-1.79581	0.04172	7.68383
H	-1.40229	-0.97227	7.58741
H	-2.63940	0.13474	6.98770
H	-2.18982	0.15100	8.69554
C	0.89500	2.24527	6.24001
C	0.35745	3.10927	7.24959
C	0.45946	-0.19200	5.45148
H	1.49414	-0.24609	5.10715
H	-0.18480	-0.14048	4.56410
H	0.23163	-1.12684	5.96742
C	-1.66400	2.97261	8.91724
H	-1.99834	2.23248	9.64468
H	-2.54774	3.35312	8.38932
H	-1.23556	3.80489	9.47729
C	0.67478	4.56135	7.39654
H	0.43171	4.93318	8.39291
H	0.09822	5.15632	6.67650
H	1.73381	4.75864	7.21672
C	1.95550	2.61473	5.25340
H	2.67124	3.32030	5.68033
H	1.51617	3.08389	4.36441
H	2.51695	1.73933	4.92108
H	2.87090	1.43135	8.44203

71

TS Complex 4 - Int 3''

C	-1.77430	1.56781	-1.69309
C	-1.88165	0.21097	-2.21159
C	-0.82946	-0.01646	-3.11369
C	-0.10585	1.29134	-3.35214
C	-0.65167	2.17909	-2.24933
Ir	0.09692	0.29492	-1.04794
Mo	1.15809	-0.65633	0.69565
N	-0.31708	-0.91444	2.38530
C	0.02039	-1.92973	3.39644
C	-3.00255	-0.72954	-1.91062
C	-0.64005	-1.19727	-4.00580
C	-0.22959	1.89986	-4.75956
C	-0.21165	3.59413	-2.08160

C	-2.74598	2.19741	-0.75030
N	3.07895	-0.42035	0.46881
C	3.80575	-0.15505	-0.75502
N	0.95571	-2.58179	0.26842
C	1.73372	-3.54769	1.01839
N	0.94847	0.92664	2.20258
C	1.38933	2.32786	2.39558
C	0.20568	3.26419	2.11208
C	3.96161	-0.63817	1.59527
C	0.14667	-3.24910	-0.72522
C	-0.04444	0.45760	2.94149
O	-0.70703	0.88368	3.87069
C	-1.73532	-1.04975	2.00727
C	1.90341	2.52955	3.82815
C	2.50800	2.64404	1.40453
H	-0.41485	-2.50870	-1.29205
H	-0.55356	-3.96816	-0.26445
H	0.77658	-3.82044	-1.42722
H	3.39552	-0.75661	2.52384
H	4.64467	0.21430	1.73507
H	4.58201	-1.53647	1.45220
H	2.42778	-4.08788	0.35441
H	1.10080	-4.30780	1.50681
H	2.33480	-3.06662	1.79518
H	4.48167	-0.99031	-0.99726
H	4.41890	0.75417	-0.65353
H	3.10890	-0.01376	-1.58042
H	3.40247	2.05270	1.61091
H	2.77537	3.70260	1.48211
H	2.18382	2.42834	0.38351
H	-0.15993	3.07217	1.09937
H	0.52007	4.31042	2.18289
H	-0.60524	3.09317	2.82204
H	-1.98190	-0.28353	1.27305
H	-2.36813	-0.93475	2.89330
H	-1.88741	-2.03212	1.55748
H	1.11514	2.33106	4.55556
H	2.25110	3.55925	3.95992
H	2.74515	1.85985	4.02969
H	-0.16667	-2.92371	2.98995
H	-0.58776	-1.77301	4.29325
H	1.07441	-1.84412	3.66821
H	-3.34421	-0.63811	-0.87844
H	-2.70606	-1.76714	-2.07309
H	-3.85973	-0.52345	-2.56384
H	0.42195	-1.38301	-4.18504
H	-1.12218	-1.03792	-4.97997
H	-1.06679	-2.10420	-3.57417
H	-3.57545	2.64999	-1.30778

H	-2.27502	2.98399	-0.15954
H	-3.17470	1.47174	-0.05671
H	0.87568	3.67847	-2.15535
H	-0.50955	3.99689	-1.11255
H	-0.64835	4.23978	-2.85589
H	0.32927	2.83674	-4.81781
H	-1.27648	2.10227	-5.00207
H	0.17652	1.21838	-5.51147
H	0.98159	1.12556	-3.17048

71

Int 3''

C	-2.10092	1.52400	-1.73415
C	-2.19624	0.16375	-2.21182
C	-0.94982	-0.16453	-2.84056
C	-0.25142	1.15412	-3.16951
C	-0.82217	2.03425	-2.05891
Ir	-0.52070	0.19996	-0.75391
Mo	0.76762	-0.84045	0.86061
N	-0.47746	-1.21775	2.66254
C	-0.01171	-2.23059	3.62335
C	-3.40481	-0.70874	-2.10552
C	-0.68180	-1.39596	-3.64442
C	-0.59678	1.68488	-4.57649
C	-0.34199	3.43156	-1.84554
C	-3.15984	2.22210	-0.94508
N	2.67428	-0.64503	0.57041
C	3.26826	-0.84547	-0.73897
N	0.55511	-2.72826	0.31602
C	1.40394	-3.74193	0.92025
N	0.57345	0.73936	2.38365
C	0.90444	2.17505	2.54884
C	-0.38140	3.00204	2.41061
C	3.65979	-0.50353	1.61838
C	-0.44970	-3.35315	-0.51625
C	-0.26260	0.16644	3.23171
O	-0.84185	0.50353	4.24769
C	-1.90969	-1.41613	2.36133
C	1.55043	2.41560	3.92115
C	1.88168	2.59000	1.45026
H	-1.10910	-2.58640	-0.92216
H	-1.04906	-4.08605	0.05118
H	0.01514	-3.90177	-1.35097
H	3.17594	-0.31482	2.57832
H	4.34077	0.33533	1.40890
H	4.27854	-1.41032	1.70956
H	1.91977	-4.32389	0.14099
H	0.83206	-4.46282	1.52760
H	2.16792	-3.28923	1.55709
H	3.91981	-1.73331	-0.74583

H	3.87693	0.02580	-1.02137
H	2.49066	-0.97218	-1.49316
H	2.83896	2.07556	1.55440
H	2.06690	3.66681	1.51355
H	1.47232	2.35225	0.46578
H	-0.83479	2.80547	1.43461
H	-0.15690	4.07090	2.48407
H	-1.09685	2.74179	3.19286
H	-2.20821	-0.70245	1.58843
H	-2.50144	-1.25882	3.26817
H	-2.05760	-2.42809	1.98247
H	0.86746	2.14707	4.72794
H	1.81989	3.47131	4.02585
H	2.46496	1.82307	4.02272
H	-0.18815	-3.22754	3.21954
H	-0.54982	-2.11143	4.56880
H	1.05676	-2.09968	3.80642
H	-3.93800	-0.54939	-1.16521
H	-3.13923	-1.76644	-2.16120
H	-4.10804	-0.50361	-2.92335
H	0.38947	-1.60848	-3.68688
H	-1.03844	-1.27217	-4.67587
H	-1.18015	-2.27278	-3.22852
H	-3.92145	2.64670	-1.61103
H	-2.74347	3.04058	-0.35501
H	-3.66474	1.53830	-0.25873
H	0.74613	3.45798	-1.74612
H	-0.76737	3.87629	-0.94436
H	-0.61364	4.07706	-2.69246
H	-0.13874	2.66453	-4.74019
H	-1.67849	1.78325	-4.70184
H	-0.21776	1.00837	-5.34810
H	0.83618	1.05639	-3.08536

71

TS Complex 4 - Int 3'

C	-1.16617	2.37598	7.76840
C	-0.85023	1.21932	6.98402
C	0.42014	1.44072	6.36228
C	0.88313	2.74407	6.74706
C	-0.09882	3.32441	7.61362
Ir	0.73566	1.46195	8.62289
Mo	1.82331	0.68543	10.44193
N	0.83199	1.61444	12.33639
C	0.88833	0.76651	13.54119
C	1.07526	0.53730	5.36797
C	2.10974	3.41976	6.22573
C	-1.72887	0.02978	6.76968
C	-0.08629	4.71772	8.15440
C	-2.44852	2.61994	8.49577

N	3.30376	-0.29829	9.68249
C	3.68878	-0.62433	8.32375
N	0.47214	-0.97874	10.56783
C	0.99498	-2.33228	10.67238
N	2.78283	2.29612	11.40638
C	3.91565	3.20522	11.09368
C	4.87332	3.27656	12.29087
C	4.24695	-0.86121	10.64235
C	-0.93338	-0.90017	10.91650
C	1.94909	2.61156	12.39851
O	1.95382	3.48662	13.24184
C	-0.45663	2.33048	12.28499
C	4.66322	2.66651	9.87567
C	3.36700	4.59815	10.75469
H	-1.32983	0.06021	10.58025
H	-1.10361	-0.99957	11.99729
H	-1.50147	-1.70190	10.42146
H	3.99093	-0.56846	11.66504
H	5.26909	-0.51267	10.43849
H	4.25627	-1.95956	10.59253
H	0.28251	-3.05292	10.24585
H	1.17982	-2.63076	11.71477
H	1.93368	-2.41460	10.12381
H	3.76861	-1.71442	8.19069
H	4.67340	-0.19271	8.09050
H	2.94605	-0.22435	7.63707
H	5.13431	1.70509	10.09091
H	5.44911	3.37161	9.58909
H	3.98068	2.53341	9.03222
H	2.66896	4.52139	9.91680
H	4.18527	5.26559	10.46739
H	2.84702	5.03330	11.60902
H	-0.50408	2.90160	11.35830
H	-0.55197	2.99713	13.14852
H	-1.26977	1.60433	12.29120
H	4.36699	3.67574	13.17037
H	5.72250	3.92458	12.05196
H	5.26274	2.28198	12.52902
H	0.10214	0.01229	13.49415
H	0.75781	1.37542	14.44242
H	1.85643	0.26362	13.59253
H	2.16171	0.65011	5.37503
H	0.73014	0.76201	4.35039
H	0.84798	-0.51241	5.56723
H	2.49057	4.16241	6.92934
H	1.89810	3.93713	5.28107
H	2.91120	2.70261	6.03640
H	-1.14288	-0.88511	6.65536
H	-2.33757	0.14927	5.86419

H	-2.41316	-0.11963	7.60723
H	-2.88749	1.68827	8.85897
H	-3.18608	3.09762	7.83799
H	-2.30364	3.27644	9.35648
H	-0.57438	4.77612	9.12957
H	-0.61374	5.40303	7.47840
H	0.93113	5.09432	8.27512
H	0.49298	-0.32544	9.40516

71

Int 3'

C	-2.38648	0.95185	-1.73688
C	-1.99855	-0.19369	-2.51103
C	-0.73675	0.09918	-3.12711
C	-0.34645	1.41845	-2.72814
C	-1.36805	1.94804	-1.87647
Ir	-0.47177	0.07930	-0.86615
Mo	0.55981	-0.79416	0.92466
N	-0.39702	0.12921	2.83936
C	-0.31519	-0.67660	4.07045
C	-0.03203	-0.76320	-4.12408
C	0.87003	2.14791	-3.19739
C	-2.83308	-1.40715	-2.76729
C	-1.41550	3.33366	-1.31903
C	-3.68701	1.12607	-1.02117
N	2.00565	-1.81184	0.13421
C	2.39959	-2.07602	-1.23646
N	-0.86892	-2.56472	1.01557
C	-0.34086	-3.83057	1.54202
N	1.52614	0.84013	1.85263
C	2.64928	1.75214	1.52017
C	3.62194	1.83713	2.70484
C	2.94878	-2.41758	1.06767
C	-2.26778	-2.34855	1.40594
C	0.72135	1.14096	2.86582
O	0.73178	2.01331	3.71430
C	-1.68496	0.85060	2.79837
C	3.38490	1.20039	0.30112
C	2.08979	3.13919	1.17824
H	-2.61608	-1.43739	0.91933
H	-2.34031	-2.23539	2.48906
H	-2.89383	-3.19894	1.10678
H	2.67524	-2.19727	2.10406
H	3.96507	-2.03327	0.90033
H	2.98963	-3.51043	0.94505
H	-1.02020	-4.66467	1.32548
H	-0.21605	-3.76002	2.62525
H	0.63023	-4.03467	1.09265
H	2.43465	-3.15952	-1.43189
H	3.40616	-1.67604	-1.43010

H	1.68570	-1.60306	-1.90737
H	3.85182	0.23769	0.52386
H	4.17240	1.89814	0.00068
H	2.69218	1.06137	-0.53256
H	1.37531	3.04775	0.35647
H	2.89860	3.80894	0.86956
H	1.58340	3.57818	2.03918
H	-1.74690	1.39972	1.85910
H	-1.75885	1.53606	3.64916
H	-2.50349	0.13062	2.83722
H	3.12623	2.24195	3.58777
H	4.46577	2.48556	2.44834
H	4.01783	0.84564	2.94593
H	-1.11675	-1.41813	4.08427
H	-0.40768	-0.03440	4.95301
H	0.64707	-1.19220	4.11032
H	1.04229	-0.56565	-4.13854
H	-0.41114	-0.58037	-5.13817
H	-0.17063	-1.82545	-3.90773
H	1.22326	2.85800	-2.44714
H	0.66367	2.71005	-4.11741
H	1.69091	1.45850	-3.40733
H	-2.21176	-2.28656	-2.95473
H	-3.47656	-1.26561	-3.64586
H	-3.48484	-1.63488	-1.92016
H	-4.08074	0.17060	-0.66563
H	-4.44467	1.56775	-1.68158
H	-3.58265	1.78407	-0.15542
H	-1.93982	3.36611	-0.36143
H	-1.93628	4.01500	-2.00455
H	-0.41376	3.73570	-1.15536
H	-0.85014	-2.55878	-0.01208

61

Int 2

C	-0.20280	3.97525	-1.64327
C	-1.44427	3.30161	-1.38903
C	-1.62244	2.30423	-2.41478
C	-0.50242	2.37217	-3.29476
C	0.38407	3.39091	-2.81866
Ir	0.25479	1.82448	-1.16417
Mo	1.17759	0.68605	0.59788
N	3.01487	1.37039	0.56930
C	3.66283	2.26306	-0.37142
C	-2.46476	3.70598	-0.37323
C	-2.83861	1.46152	-2.62144
C	-0.30430	1.54661	-4.52518
C	1.62283	3.86819	-3.50541
C	0.30870	5.18976	-0.93808
N	1.44959	-1.18820	0.06285

C	0.98901	-1.89849	-1.11503
N	0.49905	1.48640	2.46189
C	0.84839	2.59011	3.40060
C	0.06496	3.85779	3.03479
N	-0.83376	-0.09104	1.55341
C	-0.65992	-1.33895	2.32250
C	-0.99887	-6.11836	0.14306
N	-1.04866	-7.14596	-0.46911
C	-1.85688	-8.35748	-0.58373
C	-1.80380	-9.11668	0.74544
O	-0.84353	-5.08299	0.67430
C	-3.29686	-7.96752	-0.93018
C	-1.24698	-9.20050	-1.70509
C	2.23388	-2.06555	0.90981
C	-0.77807	1.05271	2.47714
O	-1.76552	1.43872	3.07225
C	-2.10111	-0.15455	0.81705
C	4.00614	0.81792	1.47620
C	0.53289	2.16073	4.84285
C	2.33949	2.89748	3.31668
H	0.35316	-1.25691	-1.72138
H	0.41977	-2.79554	-0.82718
H	1.83812	-2.23548	-1.73240
H	3.54352	0.22629	2.26921
H	4.58897	1.61430	1.96077
H	4.71515	0.17403	0.93457
H	2.52087	-1.57526	1.84387
H	3.15806	-2.38572	0.40348
H	1.67317	-2.97453	1.17397
H	4.47333	1.74438	-0.90871
H	4.11699	3.11953	0.15234
H	2.93324	2.62870	-1.09076
H	2.93525	2.03537	3.62294
H	2.57395	3.72385	3.99398
H	2.63048	3.18248	2.30476
H	0.29748	4.14491	2.00637
H	0.35005	4.68130	3.69721
H	-1.00844	3.69087	3.12557
H	-2.24148	0.77925	0.27832
H	-2.94011	-0.32226	1.50174
H	-2.04163	-0.96984	0.09418
H	-0.53234	1.97698	4.97969
H	0.84174	2.94865	5.53673
H	1.08505	1.25085	5.09830
H	-0.60322	-2.18184	1.63432
H	-1.50738	-1.48305	3.00368
H	0.25872	-1.29022	2.90762
H	-2.00083	4.10516	0.52936
H	-3.09609	2.86940	-0.06889

H	-3.12455	4.48163	-0.78243
H	-2.58718	0.49274	-3.05803
H	-3.54017	1.96032	-3.30233
H	-3.37012	1.27717	-1.68654
H	-0.07798	5.25681	0.07943
H	0.00046	6.09925	-1.46939
H	1.39918	5.19281	-0.87999
H	2.32675	4.31411	-2.79982
H	1.38178	4.63206	-4.25557
H	2.13868	3.05364	-4.01807
H	-0.74898	2.03688	-5.40007
H	-0.76765	0.56272	-4.42659
H	0.75558	1.39160	-4.73707
H	1.30901	0.66793	-1.59792
H	-3.91006	-8.86388	-1.05913
H	-3.73918	-7.36235	-0.13399
H	-3.32702	-7.39314	-1.85923
H	-2.37071	-10.04899	0.67102
H	-0.77156	-9.36078	1.00761
H	-2.23393	-8.51817	1.55315
H	-1.81302	-10.12678	-1.83346
H	-1.26029	-8.65074	-2.64918
H	-0.21037	-9.45547	-1.47253

87

TS Int 2 - Int 3

C	-0.34356	3.00343	-2.03009
C	-1.60072	2.60714	-1.47018
C	-2.00578	1.38726	-2.12243
C	-1.01614	1.05525	-3.09541
C	0.02550	2.02920	-3.02870
Ir	-0.04644	1.02681	-0.98333
Mo	0.98488	0.29194	0.92936
N	2.79373	0.88274	0.60214
C	3.40053	1.52408	-0.55217
C	-2.44912	3.39647	-0.52519
C	-3.31465	0.69222	-1.93527
C	-1.07330	-0.10346	-4.03618
C	1.20058	2.12040	-3.94747
C	0.37116	4.29564	-1.79518
N	1.45776	-1.78423	0.72014
C	2.44097	-2.04732	-0.33978
N	0.43862	1.60163	2.48690
C	0.82977	2.94272	3.01056
C	0.14288	4.05658	2.21415
N	-0.95342	-0.16074	2.20168
C	-0.80870	-0.95624	3.44681
C	-0.08045	-3.07404	0.28444
N	0.07784	-3.29906	-0.92633
C	-0.73091	-4.34991	-1.58846

C	-0.46871	-5.70747	-0.92572
O	-0.62902	-3.25747	1.33519
C	-2.22236	-4.00663	-1.51231
C	-0.27634	-4.39016	-3.04803
C	1.96087	-2.36398	1.97041
C	-0.86624	1.25677	2.59168
O	-1.84376	1.88850	2.93502
C	-2.25267	-0.48655	1.58716
C	3.81728	0.59708	1.60397
C	0.44897	3.02598	4.49773
C	2.33946	3.12325	2.90295
H	2.04536	-1.74929	-1.30771
H	2.65119	-3.12132	-0.38702
H	3.37928	-1.51153	-0.15511
H	3.37448	0.23526	2.53436
H	4.39965	1.49731	1.83425
H	4.51689	-0.16352	1.23267
H	1.21112	-2.30529	2.75512
H	2.88281	-1.88003	2.31537
H	2.18851	-3.42739	1.82255
H	4.24623	0.92498	-0.91885
H	3.79369	2.51489	-0.28045
H	2.65687	1.62582	-1.33998
H	2.86886	2.35776	3.47418
H	2.61373	4.09798	3.31587
H	2.67051	3.08761	1.86400
H	0.38572	3.94442	1.15482
H	0.49670	5.03532	2.55332
H	-0.93875	4.01103	2.33671
H	-2.35345	0.08168	0.66558
H	-3.06677	-0.23840	2.27542
H	-2.26098	-1.55118	1.36163
H	-0.62831	2.94324	4.63774
H	0.77821	3.98591	4.90652
H	0.94344	2.22992	5.06272
H	-0.84117	-2.01274	3.18949
H	-1.63368	-0.71665	4.12804
H	0.13414	-0.71019	3.93624
H	-1.87265	4.17023	-0.01841
H	-2.89885	2.77101	0.24798
H	-3.26058	3.89300	-1.07155
H	-3.21207	-0.39188	-2.01388
H	-4.03234	1.01539	-2.69973
H	-3.75365	0.91805	-0.96209
H	0.10834	4.73500	-0.83261
H	0.10386	5.02186	-2.57293
H	1.45584	4.17153	-1.82001
H	2.02950	2.66355	-3.48912
H	0.93148	2.65200	-4.86901

H	1.56647	1.13138	-4.23082
H	-1.57773	0.18207	-4.96728
H	-1.62225	-0.94134	-3.60374
H	-0.07507	-0.46098	-4.29500
H	0.47372	-0.49038	-1.13945
H	-2.81941	-4.75370	-2.04528
H	-2.56090	-3.97631	-0.47359
H	-2.41071	-3.02933	-1.96488
H	-1.02715	-6.50034	-1.43359
H	0.59580	-5.95381	-0.97310
H	-0.77298	-5.69199	0.12409
H	-0.82761	-5.15213	-3.60816
H	-0.43864	-3.42032	-3.52491
H	0.79109	-4.61905	-3.10651

87

Int 3

C	-0.47304	2.61812	-1.94225
C	-1.74044	2.19210	-1.42624
C	-2.09416	0.96336	-2.08935
C	-1.05998	0.65178	-3.02447
C	-0.04696	1.65365	-2.92557
Ir	-0.17067	0.64406	-0.88855
Mo	0.82964	-0.11688	1.01992
N	2.65773	0.41188	0.71586
C	3.31707	0.97759	-0.45028
C	-2.64020	2.95965	-0.51217
C	-3.38736	0.23162	-1.94414
C	-1.05471	-0.50908	-3.96363
C	1.15782	1.77230	-3.80112
C	0.20693	3.92358	-1.67966
N	1.34140	-2.32561	0.67720
C	2.41170	-2.43628	-0.34660
N	0.30441	1.20610	2.55295
C	0.67710	2.56859	3.03522
C	-0.14936	3.63635	2.31095
N	-1.05070	-0.58903	2.30303
C	-0.85041	-1.44903	3.49821
C	0.10360	-3.23861	0.28461
N	0.18063	-3.62450	-0.93734
C	-0.81968	-4.57513	-1.43766
C	-0.73487	-5.90335	-0.66816
O	-0.66749	-3.43214	1.23920
C	-2.24757	-4.01691	-1.34562
C	-0.47353	-4.82723	-2.90934
C	1.88079	-2.89201	1.93566
C	-0.97588	0.80827	2.75734
O	-1.93594	1.39169	3.21040
C	-2.36202	-0.91285	1.70629
C	3.64280	0.15887	1.76444

C	0.44859	2.64035	4.55333
C	2.15317	2.83630	2.76556
H	2.08803	-1.98727	-1.28030
H	2.61597	-3.49182	-0.53507
H	3.31141	-1.93784	0.01792
H	3.16321	-0.14001	2.69927
H	4.23814	1.05812	1.95963
H	4.33685	-0.63460	1.45524
H	1.08993	-2.96292	2.67429
H	2.70473	-2.28252	2.31053
H	2.25830	-3.90345	1.74492
H	4.16022	0.34141	-0.75473
H	3.72438	1.97063	-0.21297
H	2.60460	1.05739	-1.26820
H	2.78679	2.14092	3.31842
H	2.39951	3.84754	3.10032
H	2.38144	2.76354	1.70054
H	0.00889	3.54168	1.23421
H	0.17127	4.63458	2.62478
H	-1.21114	3.52334	2.52629
H	-2.52427	-0.26508	0.84761
H	-3.15547	-0.76463	2.44530
H	-2.31927	-1.95091	1.38071
H	-0.60308	2.50655	4.80504
H	0.77163	3.61701	4.92592
H	1.03601	1.87193	5.06495
H	-0.88637	-2.48555	3.16761
H	-1.64687	-1.24912	4.22368
H	0.11247	-1.22265	3.95949
H	-2.08742	3.66805	0.10462
H	-3.19280	2.30249	0.16163
H	-3.37289	3.52891	-1.09743
H	-3.25346	-0.84723	-2.03671
H	-4.09430	0.54936	-2.72063
H	-3.85520	0.42757	-0.97816
H	-0.11826	4.36897	-0.73913
H	-0.02692	4.63726	-2.47922
H	1.29302	3.81594	-1.64132
H	1.95718	2.33368	-3.31323
H	0.90833	2.29947	-4.73044
H	1.55433	0.79239	-4.07416
H	-1.55061	-0.24044	-4.90409
H	-1.57880	-1.36752	-3.54210
H	-0.03923	-0.82977	-4.20169
H	0.32797	-0.88207	-1.04813
H	-2.96462	-4.70112	-1.81248
H	-2.53207	-3.86785	-0.30310
H	-2.30530	-3.05464	-1.86334
H	-1.44047	-6.63667	-1.07388

H	0.27418	-6.31997	-0.74932
H	-0.96247	-5.74478	0.38784
H	-1.15982	-5.55159	-3.36126
H	-0.52780	-3.89388	-3.47731
H	0.54675	-5.21159	-2.99595

87

TS Int 3 - Int 4

C	-1.30050	2.86820	-1.78477
C	-2.12055	1.77535	-2.21029
C	-1.36057	0.99148	-3.13275
C	-0.06603	1.58013	-3.26274
C	-0.03497	2.75931	-2.44762
Ir	-0.23164	0.96962	-1.04494
Mo	0.85405	0.17048	0.74231
N	-0.66843	-0.43411	2.39462
C	-0.51123	-1.66241	3.19244
C	-3.56703	1.61910	-1.86982
C	-1.84317	-0.14903	-3.97140
C	0.97686	1.14401	-4.24078
C	1.04189	3.79697	-2.44594
C	-1.75784	4.01904	-0.94855
N	2.67054	0.70684	0.35979
C	3.28115	1.31335	-0.80771
N	1.21123	-1.96365	0.10901
C	-0.03168	-2.86956	0.01120
N	-1.07901	-2.22053	-0.43670
C	-2.28820	-3.01699	-0.75965
C	-2.79755	-3.82728	0.44513
N	0.48624	1.50304	2.34758
C	0.83172	2.89473	2.73771
C	-0.45863	3.67692	3.02672
C	3.65453	0.53836	1.42475
C	2.17806	-2.58264	1.04438
C	1.82331	-2.01359	-1.25088
C	-0.25036	0.76146	3.18057
O	-0.60522	0.91209	4.33275
C	-2.09645	-0.25194	2.04274
O	0.14547	-4.04337	0.32175
C	1.73281	2.89273	3.98101
C	1.55905	3.56584	1.57568
C	-3.38586	-2.04115	-1.17944
C	-1.98831	-3.97141	-1.92547
H	1.14732	-1.54071	-1.95912
H	2.00251	-3.06174	-1.51835
H	2.76854	-1.47033	-1.24182
H	3.20348	0.11822	2.32691
H	4.10171	1.50514	1.69286
H	4.47119	-0.12238	1.10164
H	3.11168	-2.02322	1.00006

H	2.34854	-3.62415	0.76641
H	1.78921	-2.56871	2.06134
H	4.18747	0.75965	-1.09494
H	3.58258	2.34887	-0.59661
H	2.56527	1.30331	-1.62681
H	2.55161	3.13856	1.42228
H	1.67990	4.63310	1.78265
H	0.98960	3.43885	0.65194
H	-1.09725	3.69567	2.14021
H	-0.21522	4.70933	3.29561
H	-1.01106	3.22758	3.85265
H	-2.20696	0.68348	1.49556
H	-2.70212	-0.23923	2.95600
H	-2.39508	-1.07118	1.39400
H	1.21646	2.44766	4.83213
H	2.01416	3.91872	4.23870
H	2.65000	2.32785	3.79220
H	-0.73945	-2.53212	2.57588
H	-1.17960	-1.63554	4.05792
H	0.51355	-1.73362	3.56153
H	-3.76481	1.89527	-0.83167
H	-3.91378	0.59548	-2.00816
H	-4.18410	2.26811	-2.50428
H	-1.27912	-1.06819	-3.79309
H	-1.74199	0.09852	-5.03436
H	-2.89740	-0.36257	-3.79314
H	-2.47685	3.70308	-0.19019
H	-2.24879	4.77715	-1.57237
H	-0.92603	4.50593	-0.43632
H	1.14395	4.28262	-1.47363
H	0.81394	4.57921	-3.18096
H	2.01361	3.37740	-2.71188
H	1.97930	1.45169	-3.93591
H	0.78942	1.58235	-5.22981
H	0.98418	0.05827	-4.35924
H	-0.76996	-0.78765	-0.74876
H	-4.29033	-2.58908	-1.45997
H	-3.64169	-1.35473	-0.36866
H	-3.06145	-1.44914	-2.03330
H	-3.71862	-4.35151	0.17007
H	-2.05892	-4.56058	0.76669
H	-3.03260	-3.17154	1.28897
H	-2.88589	-4.53384	-2.20269
H	-1.65440	-3.40989	-2.80293
H	-1.20890	-4.68179	-1.64437

87

Int 4

C	-1.40041	2.49634	-1.63862
C	-1.99106	1.29432	-2.13042

C	-1.04997	0.66253	-3.00865
C	0.12132	1.48333	-3.06461
C	-0.09435	2.61673	-2.21455
Ir	-0.08233	0.73381	-0.91976
Mo	0.97756	-0.19624	0.82378
N	-0.59857	-0.70862	2.46406
C	-0.43047	-1.89637	3.31953
C	-3.40625	0.87920	-1.89261
C	-1.29834	-0.52745	-3.87718
C	1.28036	1.27667	-3.98605
C	0.81322	3.79769	-2.08797
C	-2.08066	3.50713	-0.77384
N	2.82624	0.21276	0.40028
C	3.45592	0.81504	-0.75924
N	1.16497	-2.45033	0.13108
C	-0.00920	-3.35202	0.26075
N	-1.13412	-2.87453	-0.28730
C	-2.33866	-3.71850	-0.49377
C	-2.87052	-4.26995	0.83562
N	0.66619	1.17129	2.40490
C	1.09205	2.54628	2.77247
C	-0.15040	3.43249	2.94503
C	3.81494	-0.02972	1.44550
C	2.27542	-3.02521	0.93016
C	1.58418	-2.46203	-1.30391
C	-0.16219	0.51572	3.21336
O	-0.59491	0.74099	4.32844
C	-2.02908	-0.52969	2.13073
O	0.12325	-4.43337	0.79967
C	1.90826	2.51614	4.07277
C	1.94632	3.12007	1.64564
C	-3.40531	-2.83255	-1.13104
C	-1.99407	-4.87436	-1.44188
H	0.82476	-1.96969	-1.90628
H	1.75570	-3.49686	-1.62771
H	2.50570	-1.88959	-1.39467
H	3.35232	-0.45142	2.34171
H	4.31321	0.90659	1.73323
H	4.59931	-0.72001	1.10056
H	3.15935	-2.41623	0.75941
H	2.46884	-4.05878	0.63531
H	2.02143	-3.01624	1.98914
H	4.26516	0.17114	-1.13839
H	3.90812	1.78281	-0.49683
H	2.70609	0.96333	-1.53358
H	2.90224	2.59887	1.56550
H	2.15325	4.17611	1.84340
H	1.42623	3.02388	0.68977
H	-0.73115	3.44208	2.01952

H	0.15157	4.45902	3.17508
H	-0.78309	3.06845	3.75548
H	-2.13187	0.36177	1.51256
H	-2.61828	-0.43718	3.05043
H	-2.36643	-1.39094	1.55644
H	1.30951	2.13111	4.89898
H	2.24536	3.52683	4.32441
H	2.79330	1.88373	3.95494
H	-0.73645	-2.79240	2.77905
H	-1.03033	-1.79260	4.22889
H	0.61765	-1.99351	3.61136
H	-3.56361	-0.17305	-2.12908
H	-4.09112	1.46122	-2.52296
H	-3.70660	1.03627	-0.85348
H	-0.37854	-1.08655	-4.06527
H	-1.70057	-0.22342	-4.85256
H	-2.01821	-1.21533	-3.43059
H	-2.76029	3.03312	-0.06171
H	-2.67092	4.20770	-1.37904
H	-1.36129	4.09474	-0.20014
H	0.67756	4.31158	-1.13447
H	0.61248	4.52565	-2.88457
H	1.86477	3.51189	-2.16085
H	2.17222	1.80272	-3.63899
H	1.05202	1.65510	-4.99098
H	1.53596	0.21851	-4.08318
H	-1.10662	-1.90046	-0.62020
H	-4.31838	-3.41221	-1.28539
H	-3.64831	-1.97830	-0.49442
H	-3.07655	-2.45855	-2.10320
H	-3.76411	-4.87123	0.64458
H	-2.12728	-4.89781	1.32542
H	-3.15365	-3.46104	1.51413
H	-2.88350	-5.48304	-1.62747
H	-1.63586	-4.49095	-2.40136
H	-1.22552	-5.51610	-1.00809

77

Int 5

C	-1.48703	2.85291	-1.88668
C	-2.03377	1.54380	-1.97158
C	-1.08352	0.70890	-2.66147
C	0.04287	1.53640	-3.03310
C	-0.20499	2.85362	-2.52730
Ir	-0.02961	1.29184	-0.80867
Mo	1.06551	0.25486	0.80705
N	-0.40668	-0.71479	2.21273
C	0.13755	-1.88123	2.93508
C	-3.39064	1.12086	-1.50993
C	-1.31374	-0.69565	-3.11860

C	1.14972	1.13812	-3.95346
C	0.67397	4.04656	-2.72719
C	-2.13311	4.03567	-1.24094
N	2.92068	0.17335	0.27081
C	3.56859	0.48373	-0.99103
N	0.67145	1.25785	2.57016
C	0.95354	2.61380	3.10123
C	-0.26032	3.51710	2.85549
C	3.87620	-0.36318	1.23216
C	-0.31543	0.51552	3.08525
O	-1.08548	0.67670	4.00816
C	-1.80874	-0.97460	1.83229
C	1.27879	2.52864	4.59848
C	2.16584	3.16607	2.35245
H	3.39896	-0.55508	2.19755
H	4.69436	0.35205	1.40011
H	4.31946	-1.30353	0.87472
H	4.02223	-0.41667	-1.43234
H	4.37311	1.21696	-0.83222
H	2.83677	0.89735	-1.67961
H	3.04605	2.53742	2.51786
H	2.39424	4.17437	2.71012
H	1.97149	3.20687	1.27755
H	-0.49177	3.53813	1.78651
H	-0.04784	4.53730	3.18996
H	-1.13319	3.14894	3.39815
H	-2.20758	-0.10079	1.32005
H	-2.40795	-1.19403	2.72198
H	-1.83141	-1.82324	1.14595
H	0.42881	2.14667	5.16449
H	1.53385	3.52287	4.97756
H	2.13741	1.87110	4.76646
H	0.14204	-2.74780	2.26990
H	-0.47427	-2.10183	3.81718
H	1.16002	-1.67642	3.25703
H	-3.40907	0.06414	-1.23490
H	-4.13638	1.26675	-2.30186
H	-3.71870	1.69564	-0.64088
H	-0.37318	-1.24535	-3.19369
H	-1.79424	-0.71331	-4.10541
H	-1.95850	-1.24073	-2.42601
H	-2.82185	3.73405	-0.44898
H	-2.70491	4.62143	-1.97178
H	-1.39204	4.70325	-0.79495
H	0.57334	4.76064	-1.90686
H	0.41867	4.57379	-3.65537
H	1.72700	3.76310	-2.78558
H	2.04271	1.74695	-3.79686
H	0.84386	1.26927	-4.99961

H 1.42978 0.09113 -3.82001
87

Int 5 - Int 6

C	-3.02982	1.63412	-0.30290
C	-3.17474	0.33126	0.28371
C	-2.73022	-0.63145	-0.70635
C	-2.35679	0.09368	-1.89598
C	-2.54327	1.47849	-1.64294
Ir	-1.02555	0.57129	-0.06156
Mo	0.92082	0.21145	1.07416
C	-0.01949	-2.32794	2.44562
N	0.92528	-1.22651	2.39187
C	1.89912	-1.29162	3.46602
C	-3.84104	0.01635	1.58351
C	-2.86585	-2.11594	-0.60441
C	-1.91734	-0.51740	-3.18680
C	-2.27411	2.58769	-2.60697
C	-3.41374	2.92681	0.34036
C	1.37909	2.30899	1.07217
N	2.70939	2.49430	0.75781
C	3.36194	3.77710	0.94549
N	2.62623	0.34535	-0.09946
C	3.38422	1.46645	0.02834
O	4.50330	1.68536	-0.41306
C	3.10067	-0.71416	-1.02928
C	2.05782	-1.83430	-1.00406
C	3.20328	-0.16127	-2.45691
C	4.44780	-1.28753	-0.56718
H	1.08302	1.77205	2.12590
H	1.40914	-1.24434	4.45006
H	2.60708	-0.46079	3.39812
H	2.47569	-2.22744	3.42377
H	5.22499	-0.52465	-0.59258
H	4.74253	-2.11671	-1.21897
H	4.36437	-1.67140	0.45439
H	-2.00084	3.51053	-2.09091
H	-3.16114	2.80255	-3.21593
H	-1.45853	2.33681	-3.28854
H	-3.33045	2.87142	1.42740
H	-4.45087	3.19085	0.09776
H	-2.77796	3.74744	0.00035
H	1.99980	-2.29487	-0.01115
H	2.32828	-2.62032	-1.71549
H	1.06698	-1.45061	-1.27441
H	2.77140	4.58233	0.49553
H	4.33120	3.72401	0.45179
H	3.50949	3.99325	2.00846
H	-3.45463	-0.90787	2.01674

H	-4.92418	-0.10151	1.44849
H	-3.67960	0.81156	2.31357
H	-2.77365	-0.68125	-3.85334
H	-1.43445	-1.48376	-3.02910
H	-1.20670	0.12336	-3.71314
H	2.23358	0.23067	-2.77707
H	3.50021	-0.95528	-3.14966
H	3.94348	0.63779	-2.50913
H	-2.84277	-2.44999	0.43429
H	-2.06581	-2.63042	-1.14072
H	-3.82086	-2.44342	-1.03450
H	0.85625	3.25712	1.22410
H	0.48072	1.83536	0.19984
H	0.49698	-3.29512	2.34713
H	-0.55786	-2.33566	3.40498
H	-0.74756	-2.23687	1.64008

61

Int 6

C	-2.37563	0.93389	-1.71474
C	-2.61692	1.21890	-0.32297
C	-3.01234	-0.00860	0.31788
C	-2.99522	-1.04733	-0.68108
C	-2.61137	-0.45583	-1.93060
Ir	-0.93065	-0.20718	-0.33982
Mo	1.09939	-0.25737	0.77242
C	0.56144	-3.24408	1.28974
N	1.33410	-2.03369	1.52343
C	2.56874	-2.34196	2.23335
C	-2.61181	2.57629	0.30343
C	-3.50539	-0.15505	1.72156
C	-3.43353	-2.46084	-0.47693
C	-2.50889	-1.16952	-3.23994
C	-2.03433	1.94134	-2.76307
C	0.82827	1.18278	2.24373
N	2.27807	1.10732	2.19243
C	3.02702	1.07665	3.44378
N	2.40659	1.06004	-0.08661
C	2.99303	1.59291	1.02949
O	3.97497	2.29523	1.15837
C	2.87586	1.50326	-1.42529
C	2.07966	0.76084	-2.49344
C	2.64519	3.01359	-1.57392
C	4.36520	1.16511	-1.59792
H	0.44703	0.93287	3.23115
H	2.35137	-2.76217	3.22570
H	3.17726	-1.44552	2.36869
H	3.16893	-3.08112	1.68392
H	4.97919	1.69926	-0.87350
H	4.69039	1.43753	-2.60674

H	4.52365	0.08974	-1.47070
H	-1.75080	-0.71885	-3.88377
H	-3.46380	-1.13839	-3.77889
H	-2.24157	-2.21927	-3.10322
H	-1.45122	2.76732	-2.35156
H	-2.94582	2.36615	-3.20274
H	-1.45368	1.49745	-3.57412
H	2.24988	-0.31818	-2.43211
H	2.39643	1.10213	-3.48377
H	1.00728	0.92770	-2.37466
H	3.03105	2.06427	3.91558
H	4.05974	0.78561	3.25174
H	2.56082	0.35741	4.12009
H	-2.40133	2.51930	1.37314
H	-3.58556	3.06725	0.18185
H	-1.85535	3.22078	-0.14890
H	-2.91057	-3.14416	-1.14931
H	-4.50896	-2.56594	-0.66914
H	-3.24742	-2.79335	0.54628
H	1.58317	3.24664	-1.45658
H	2.95955	3.34454	-2.56832
H	3.21536	3.57320	-0.83053
H	-3.25968	-1.13779	2.12815
H	-4.59524	-0.03369	1.76513
H	-3.06027	0.59321	2.37947
H	0.40991	2.11239	1.85535
H	0.28741	-1.23558	-0.83678
H	1.15632	-3.99632	0.74948
H	0.26090	-3.68695	2.24997
H	-0.33189	-3.00418	0.71770

61

TS Int 6 - Int 7

C	-2.30253	0.86727	-1.70909
C	-2.25381	1.39811	-0.36916
C	-2.80760	0.41385	0.51613
C	-3.18609	-0.73327	-0.27425
C	-2.88605	-0.43863	-1.64414
Ir	-0.97073	-0.42951	-0.40396
Mo	1.08757	-0.92925	0.60993
C	1.08109	-3.85582	-0.37219
N	1.63176	-2.76259	0.41368
C	2.89735	-3.12910	1.04018
C	-1.83237	2.78353	0.00064
C	-3.07478	0.58442	1.97711
C	-3.89262	-1.94896	0.23089
C	-3.17734	-1.32480	-2.81183
C	-1.93944	1.60584	-2.95552
C	1.38870	-0.19155	2.45895
N	1.54290	1.14820	2.42397

C	1.34550	2.02331	3.56746
N	2.26954	0.74780	0.24632
C	2.10324	1.69969	1.20017
O	2.36302	2.88985	1.16111
C	2.88742	1.15103	-1.04499
C	3.04624	-0.12366	-1.87832
C	1.98596	2.13433	-1.79833
C	4.28081	1.74952	-0.80759
H	1.41809	-0.65189	3.44898
H	2.75007	-3.95550	1.74885
H	3.31361	-2.28051	1.59038
H	3.63206	-3.45053	0.28935
H	4.21995	2.67487	-0.23608
H	4.75880	1.96157	-1.76926
H	4.91151	1.03975	-0.26320
H	-2.44761	-1.18562	-3.61191
H	-4.17026	-1.10842	-3.22491
H	-3.15997	-2.37890	-2.52778
H	-1.14463	2.33143	-2.77411
H	-2.80553	2.15464	-3.34724
H	-1.59627	0.92664	-3.73853
H	3.67277	-0.85502	-1.35620
H	3.52626	0.10832	-2.83355
H	2.07227	-0.57439	-2.09477
H	0.29048	2.05172	3.85303
H	1.67177	3.02221	3.28175
H	1.93777	1.67494	4.41954
H	-1.50846	2.83713	1.04119
H	-2.66277	3.48914	-0.12827
H	-1.00065	3.12745	-0.61633
H	-3.63991	-2.83044	-0.36147
H	-4.98140	-1.81585	0.18659
H	-3.62563	-2.16231	1.26768
H	0.99682	1.68597	-1.92785
H	2.40372	2.35624	-2.78587
H	1.88930	3.06805	-1.24245
H	-2.95485	-0.35732	2.51699
H	-4.09950	0.93822	2.14634
H	-2.39627	1.31280	2.42519
H	0.04377	-0.68192	2.00958
H	-0.00138	-1.45524	-1.24103
H	1.78179	-4.16428	-1.16132
H	0.90311	-4.72538	0.27508
H	0.13826	-3.55520	-0.82261

61

Int 7

C	-2.18797	1.07173	-1.47057
C	-2.20872	1.50577	-0.09667
C	-2.88486	0.50467	0.67930

C	-3.29486	-0.54374	-0.22556
C	-2.87824	-0.19091	-1.54245
Ir	-1.02936	-0.37716	-0.22009
Mo	1.22032	-0.73335	0.63948
C	1.18241	-3.72624	0.00372
N	1.83057	-2.54159	0.54061
C	3.20389	-2.79766	0.95709
C	-1.72920	2.82964	0.40277
C	-3.25457	0.60283	2.12484
C	-4.09721	-1.74818	0.14840
C	-3.16817	-0.95437	-2.79421
C	-1.72872	1.88067	-2.64057
C	1.56233	-0.06552	2.42284
N	1.54125	1.29535	2.24128
C	1.10619	2.22180	3.27481
N	2.39604	0.83330	0.13557
C	2.08254	1.83579	1.02153
O	2.18173	3.04334	0.87767
C	3.10661	1.18272	-1.11978
C	3.40259	-0.12865	-1.84990
C	2.23240	2.06916	-2.01401
C	4.43446	1.87846	-0.79106
H	1.65400	-0.41118	3.45424
H	3.22748	-3.55025	1.75655
H	3.66280	-1.88133	1.33763
H	3.80608	-3.16939	0.11726
H	4.26132	2.82625	-0.28140
H	4.99058	2.07358	-1.71327
H	5.05029	1.24088	-0.14945
H	-2.38066	-0.81660	-3.53726
H	-4.11119	-0.61744	-3.24231
H	-3.25657	-2.02498	-2.60049
H	-0.93730	2.57670	-2.35988
H	-2.55864	2.46909	-3.05176
H	-1.34423	1.24608	-3.44164
H	4.02602	-0.78592	-1.23653
H	3.93921	0.07604	-2.78084
H	2.47763	-0.65648	-2.10093
H	0.05615	2.04936	3.52249
H	1.23946	3.23573	2.90048
H	1.71110	2.08215	4.17677
H	-1.48108	2.78678	1.46397
H	-2.50882	3.59056	0.27215
H	-0.83741	3.16625	-0.12737
H	-3.89037	-2.58946	-0.51569
H	-5.17052	-1.53001	0.08706
H	-3.88224	-2.07030	1.16880
H	1.28058	1.56787	-2.20905
H	2.73309	2.25217	-2.97029

H	2.03777	3.02741	-1.53122
H	-3.28513	-0.38012	2.59902
H	-4.24381	1.06208	2.24473
H	-2.53834	1.21148	2.67941
H	-0.64744	-1.58159	0.84185
H	0.06126	-1.02569	-1.25251
H	1.74956	-4.12231	-0.85014
H	1.13345	-4.51237	0.76924
H	0.17307	-3.48282	-0.32211

61

TS Int 7 - Int 8'

C	0.07083	7.67221	5.37537
C	0.28489	6.54193	4.52607
C	0.29235	6.99785	3.15352
C	0.10919	8.41625	3.16924
C	-0.01272	8.84679	4.53652
Ir	2.00585	7.95081	4.28614
Mo	4.29077	8.41585	5.11881
C	3.28921	9.40504	7.19470
N	4.54994	7.92328	6.79658
C	4.67189	7.24182	8.03862
C	0.35179	5.11238	4.96073
C	0.37134	6.12799	1.94167
C	-0.00865	9.29390	1.96459
C	-0.35767	10.22508	5.00196
C	-0.16426	7.64341	6.84994
C	5.03979	10.27254	4.82449
N	5.45271	10.45482	3.57596
C	5.63096	11.75270	2.94171
N	5.67149	8.13984	3.55117
C	5.84516	9.25574	2.81630
O	6.28890	9.41710	1.69204
C	6.23025	6.86954	3.01169
C	6.09390	5.79193	4.08990
C	5.45953	6.43758	1.75730
C	7.72715	7.03204	2.70160
H	5.04006	11.17232	5.44714
H	5.38844	6.42543	7.90797
H	3.71572	6.82124	8.37484
H	5.05868	7.90908	8.81906
H	7.89335	7.76835	1.91627
H	8.14196	6.07369	2.37397
H	8.26723	7.34645	3.60021
H	0.04944	10.42432	5.99585
H	-1.44563	10.35892	5.05822
H	0.03496	10.98664	4.32544
H	0.30925	6.77709	7.31546
H	-1.23853	7.59232	7.06906
H	0.22578	8.54111	7.33461

H	6.59139	6.10312	5.01351
H	6.56278	4.86629	3.74251
H	5.04680	5.57807	4.31612
H	6.38276	11.65791	2.16066
H	5.94601	12.47756	3.69543
H	4.69395	12.08824	2.48824
H	0.99719	4.52646	4.30366
H	-0.64496	4.65490	4.94444
H	0.74516	5.02103	5.97468
H	0.32050	10.31206	2.17881
H	-1.05048	9.34329	1.62565
H	0.59575	8.91891	1.13677
H	4.39498	6.33977	1.98695
H	5.82935	5.47166	1.39719
H	5.58054	7.17481	0.96224
H	0.80979	6.66104	1.09601
H	-0.62597	5.78352	1.63921
H	0.98522	5.24421	2.12533
H	3.25847	6.85286	4.34148
H	2.99921	8.90607	3.41635
H	2.61944	8.78388	7.77832
H	2.73447	10.05307	6.52483
H	4.01390	9.95064	7.79296

61

Int 8'

C	-3.02001	-0.48833	1.13603
C	-2.73783	-1.70447	0.43897
C	-2.66635	-1.41168	-0.97436
C	-2.89825	-0.00709	-1.13962
C	-3.10208	0.57515	0.15804
Ir	-1.05645	-0.26744	0.11461
Mo	1.21144	0.41179	0.92302
C	0.03118	1.92946	2.06171
N	1.75206	-0.40012	2.34042
C	1.83590	-1.25479	3.47191
C	-2.64843	-3.07035	1.04161
C	-2.50205	-2.41451	-2.06872
C	-2.98629	0.71264	-2.44709
C	-3.47128	1.99183	0.45503
C	-3.28739	-0.33301	2.59647
C	2.27863	2.27226	0.75949
N	2.86410	2.49624	-0.37033
C	3.48043	3.75111	-0.79052
N	2.60272	0.21210	-0.74805
C	3.01320	1.34545	-1.31398
O	3.50267	1.61174	-2.39783
C	2.92786	-1.04468	-1.49766
C	2.63891	-2.25181	-0.60209
C	2.07991	-1.13639	-2.77106

C	4.42918	-1.08700	-1.83292
H	2.30406	3.11235	1.45895
H	2.87532	-1.54877	3.66176
H	1.24146	-2.16308	3.31853
H	1.45297	-0.74219	4.36268
H	4.71777	-0.28458	-2.50949
H	4.66621	-2.04385	-2.30755
H	5.02218	-1.00943	-0.91609
H	-3.00706	2.33556	1.38242
H	-4.55783	2.10015	0.56270
H	-3.14758	2.66274	-0.34275
H	-2.84709	-1.14737	3.17409
H	-4.36689	-0.32735	2.79239
H	-2.87880	0.60548	2.97900
H	3.16517	-2.16318	0.35234
H	2.98737	-3.16004	-1.10282
H	1.57252	-2.36297	-0.40425
H	4.55102	3.60336	-0.93701
H	3.29728	4.50385	-0.02468
H	3.05040	4.05932	-1.74346
H	-1.93251	-3.69609	0.50519
H	-3.62270	-3.57251	1.00663
H	-2.33217	-3.02759	2.08512
H	-2.68990	1.75814	-2.34869
H	-4.01399	0.68995	-2.82905
H	-2.34028	0.25596	-3.19902
H	1.01820	-1.10048	-2.51233
H	2.27966	-2.07853	-3.29230
H	2.31052	-0.31109	-3.44683
H	-2.03385	-1.97073	-2.94921
H	-3.47408	-2.82087	-2.37657
H	-1.87872	-3.25296	-1.75179
H	0.17969	-1.27546	0.54948
H	-0.06693	0.69814	-0.76750
H	-0.78822	1.46837	2.61295
H	-0.38802	2.69822	1.40437
H	0.69762	2.41540	2.78473

61

TS Int 8' - Int 9''

C	-2.89884	0.52521	0.40622
C	-2.87508	-0.74926	1.08490
C	-2.77094	-1.77914	0.09498
C	-2.74550	-1.14437	-1.20430
C	-2.83028	0.26763	-1.00834
Ir	-0.94977	-0.48025	0.01502
Mo	1.39199	-0.33452	0.87213
C	0.55251	1.02039	2.57211
N	1.89471	-1.81495	1.59167
C	1.91133	-3.19171	1.95049

C	-3.04582	-0.94679	2.55600
C	-2.83282	-3.25334	0.34048
C	-2.73741	-1.85138	-2.52039
C	-2.90747	1.29778	-2.08872
C	-3.13097	1.85275	1.05339
C	2.44304	1.11268	1.87643
N	2.77466	2.14788	1.09212
C	3.25356	3.42688	1.58866
N	2.62634	0.54729	-0.59508
C	2.91158	1.84641	-0.32746
O	3.29127	2.72941	-1.07465
C	2.94401	0.04240	-1.96337
C	2.72962	-1.47331	-1.97122
C	2.03182	0.69117	-3.01196
C	4.42323	0.30207	-2.29021
H	2.91754	1.07913	2.85545
H	1.76825	-3.30325	3.03185
H	2.86832	-3.65424	1.68122
H	1.10403	-3.73147	1.44160
H	4.64239	1.36787	-2.33190
H	4.66621	-0.14271	-3.26024
H	5.06365	-0.16220	-1.53380
H	-2.48561	2.25038	-1.76384
H	-3.95011	1.47517	-2.37930
H	-2.36146	0.98320	-2.97986
H	-2.68607	1.89547	2.05051
H	-4.20412	2.05299	1.16456
H	-2.70246	2.66645	0.46466
H	3.33051	-1.95406	-1.19322
H	3.03687	-1.88046	-2.93904
H	1.67995	-1.73041	-1.81478
H	3.58827	4.01029	0.73205
H	4.08858	3.28032	2.28171
H	2.45084	3.96424	2.10099
H	-2.63194	-1.90266	2.88160
H	-4.10896	-0.93142	2.82718
H	-2.54854	-0.15732	3.12428
H	-2.25670	-1.25005	-3.29428
H	-3.76063	-2.06790	-2.85267
H	-2.20195	-2.80093	-2.46128
H	0.98388	0.49379	-2.77054
H	2.24278	0.27631	-4.00322
H	2.19226	1.76962	-3.04520
H	-2.25728	-3.80584	-0.40476
H	-3.86831	-3.61270	0.29481
H	-2.43350	-3.51212	1.32282
H	0.20132	-1.62905	-0.24246
H	0.06172	0.70982	-0.45807
H	0.14617	0.17272	3.13041

H	-0.22340	1.58323	2.06371
H	1.03427	1.67160	3.30667

61

Int 9''

C	-3.24484	0.59590	-0.85475
C	-3.65690	0.18968	0.44604
C	-3.19078	-1.16415	0.67060
C	-2.48194	-1.58043	-0.49932
C	-2.48319	-0.47649	-1.43804
Ir	-1.38227	0.12016	0.37956
Mo	0.94779	0.03542	1.15175
N	2.58906	0.79105	0.16183
C	3.57479	0.32636	-0.83724
C	3.40895	1.12356	-2.13889
C	-4.50185	0.98001	1.39141
C	-3.52563	-2.01231	1.85600
C	-1.93596	-2.94420	-0.77070
C	-1.96783	-0.52301	-2.84110
C	-3.57257	1.89354	-1.52016
N	1.36745	-1.55966	1.66185
C	1.66805	-2.83150	2.21656
C	0.74359	1.68233	3.98853
C	1.51600	1.24925	2.77050
N	1.57826	2.17284	1.64861
C	0.89315	3.46281	1.70515
C	2.62037	2.06141	0.63041
O	3.26333	3.03143	0.28481
C	4.99565	0.49244	-0.28092
C	3.30710	-1.15273	-1.11082
H	2.51659	0.88613	3.02431
H	2.26937	-3.42153	1.51316
H	0.74980	-3.39448	2.42650
H	2.23641	-2.73743	3.15036
H	5.21576	1.54288	-0.08507
H	5.72746	0.11309	-1.00040
H	5.10743	-0.07177	0.64967
H	-2.77847	-2.79242	2.01072
H	-4.49909	-2.50092	1.72463
H	-3.57365	-1.41848	2.77098
H	-1.02022	-2.89573	-1.36249
H	-2.66599	-3.54491	-1.32787
H	-1.70381	-3.47344	0.15452
H	3.43262	-1.74712	-0.20321
H	4.00490	-1.52142	-1.86811
H	2.28899	-1.30622	-1.48170
H	1.38028	4.13398	2.41897
H	-0.14964	3.31559	1.98253
H	0.93654	3.91943	0.71816
H	-1.66276	0.46661	-3.18621

H	-2.73937	-0.89387	-3.52769
H	-1.10207	-1.18218	-2.92720
H	-4.26540	0.74320	2.43054
H	-5.56617	0.76377	1.23557
H	-4.35699	2.05338	1.25545
H	2.39658	0.99767	-2.53355
H	4.11829	0.76514	-2.89154
H	3.58747	2.18545	-1.96642
H	-3.73024	2.68990	-0.79046
H	-4.48866	1.80133	-2.11635
H	-2.77180	2.21276	-2.18954
H	-0.38916	1.33960	-0.06597
H	-0.84684	0.32585	1.95727
H	0.74184	0.85557	4.70271
H	1.20123	2.54846	4.48295
H	-0.29683	1.91875	3.75942

61

TS Int 7 - Int 8

C	0.18679	8.92879	4.29642
C	0.07337	8.07131	5.43168
C	0.06152	6.70059	4.96415
C	0.14953	6.72720	3.53099
C	0.24337	8.09626	3.11201
Ir	2.00434	7.62786	4.38748
Mo	4.43596	7.78640	5.11113
C	3.86994	6.78137	7.06916
N	4.77514	6.19984	6.18285
C	4.72013	4.78890	5.87790
C	-0.12432	8.50582	6.84799
C	-0.18640	5.49241	5.80956
C	0.06950	5.54069	2.62548
C	0.25239	8.58922	1.70166
C	0.13659	10.42322	4.30539
C	4.96730	9.28994	6.23277
N	5.58692	10.34178	5.63420
C	5.80930	11.63031	6.25929
N	5.74080	8.83901	3.89349
C	6.04581	10.10943	4.30848
O	6.62152	10.99140	3.69042
C	6.21500	8.42092	2.54565
C	5.86583	6.94031	2.37364
C	5.51599	9.23233	1.44709
C	7.74155	8.56263	2.44085
H	4.77825	9.43749	7.29956
H	5.22652	4.20890	6.65919
H	5.22709	4.59477	4.93030
H	3.68056	4.43953	5.79946
H	8.04658	9.60533	2.51376
H	8.08402	8.16166	1.48130

H	8.22926	7.99645	3.24038
H	0.80842	9.52393	1.61051
H	-0.77008	8.77247	1.34853
H	0.71330	7.86493	1.02793
H	0.52354	10.83129	5.24060
H	-0.89518	10.77590	4.18660
H	0.72858	10.84738	3.49246
H	6.34119	6.33583	3.15502
H	6.23194	6.58017	1.40744
H	4.78461	6.77656	2.40248
H	6.34353	11.51012	7.20683
H	4.86116	12.14514	6.44547
H	6.41175	12.22934	5.57741
H	0.31363	7.79622	7.55307
H	-1.19414	8.58165	7.07885
H	0.32581	9.48220	7.03466
H	0.61459	5.71170	1.69574
H	-0.97451	5.32552	2.36793
H	0.48799	4.64945	3.09578
H	4.43189	9.10300	1.51629
H	5.84032	8.89055	0.45850
H	5.75427	10.29192	1.54460
H	0.25653	4.59766	5.36855
H	-1.26260	5.30984	5.92249
H	0.23257	5.61027	6.81103
H	3.08199	6.49025	3.91100
H	3.11848	8.74401	3.91007
H	3.00923	6.16058	7.34334
H	4.28691	7.31957	7.92286
H	3.02935	7.60816	6.28113

61

Int 8

C	-2.85342	0.98048	-0.57943
C	-3.07765	0.47139	0.73367
C	-3.17062	-0.97201	0.64922
C	-3.01507	-1.33646	-0.72877
C	-2.80370	-0.14030	-1.49724
Ir	-1.13092	-0.35260	-0.04800
Mo	1.40091	-0.39097	0.65506
C	1.77904	-1.69399	2.35916
N	2.10454	-2.12624	1.08834
C	1.82414	-3.44724	0.57241
C	-3.29396	1.28111	1.97146
C	-3.53495	-1.89150	1.77058
C	-3.14773	-2.71759	-1.28646
C	-2.73098	-0.04671	-2.98776
C	-2.79315	2.42231	-0.97027
C	1.93905	0.93524	1.99513
N	2.33710	2.14180	1.51076

C	2.49644	3.33957	2.31439
N	2.56992	0.90966	-0.40502
C	2.74710	2.14968	0.15330
O	3.18147	3.16420	-0.37035
C	3.05683	0.69135	-1.79549
C	2.80841	-0.77666	-2.15363
C	2.30062	1.58168	-2.79078
C	4.56765	0.95657	-1.86667
H	1.91786	0.89095	3.08466
H	2.62322	-4.13589	0.87148
H	1.77561	-3.43059	-0.51873
H	0.87277	-3.83168	0.96487
H	4.79089	1.99751	-1.63471
H	4.93693	0.73383	-2.87285
H	5.09815	0.31459	-1.15724
H	-2.10760	0.79111	-3.30504
H	-3.72970	0.09789	-3.41818
H	-2.30856	-0.95332	-3.42441
H	-2.42512	3.04386	-0.15277
H	-3.79076	2.78487	-1.24578
H	-2.13416	2.57810	-1.82585
H	3.31770	-1.44259	-1.44984
H	3.19959	-0.98201	-3.15462
H	1.73910	-1.01353	-2.16353
H	3.16350	3.14843	3.16063
H	1.52912	3.68310	2.69152
H	2.92961	4.11087	1.67846
H	-2.94049	0.75484	2.85992
H	-4.36156	1.49014	2.11042
H	-2.77042	2.23713	1.92229
H	-2.55026	-2.84229	-2.19071
H	-4.19355	-2.92339	-1.54331
H	-2.82561	-3.47241	-0.56754
H	1.22653	1.38067	-2.73665
H	2.63662	1.37116	-3.81142
H	2.47503	2.63549	-2.57464
H	-3.14502	-2.89716	1.60447
H	-4.62491	-1.96832	1.87100
H	-3.13864	-1.53810	2.72421
H	-0.13760	-1.57814	-0.44346
H	0.05542	0.56023	-0.70189
H	0.91032	-2.15581	2.84381
H	2.58757	-1.41532	3.03091
H	-0.31506	-0.09828	1.40887

61

TS Int 8 - Int 9'

C	0.17445	9.05600	3.49905
C	-0.33401	8.62609	4.78665
C	-0.53918	7.21112	4.72151

C	-0.14260	6.74842	3.41693
C	0.28345	7.90648	2.66028
Ir	1.63714	7.61711	4.41640
Mo	4.07080	7.67750	5.35118
N	4.32755	6.49029	6.87812
C	4.11955	5.17172	7.40997
C	-0.71689	9.52781	5.91583
C	-1.14295	6.36225	5.79406
C	-0.32600	5.36665	2.87587
C	0.66211	7.90205	1.21435
C	0.42007	10.47443	3.09446
C	5.10702	8.85024	6.59701
N	5.13822	10.15531	6.17041
C	5.72021	11.23740	6.94718
C	3.94536	7.68367	7.54609
N	5.23281	8.91923	4.18430
C	5.30470	10.18796	4.74153
O	5.52549	11.23623	4.17051
C	5.65541	8.75542	2.76586
C	5.69562	7.25294	2.47165
C	4.65847	9.43766	1.82079
C	7.07176	9.31124	2.55419
H	2.87552	7.88240	7.69493
H	4.49021	7.94973	8.45543
H	5.96410	8.56031	7.21697
H	4.41559	4.43661	6.65800
H	3.06489	4.98844	7.66857
H	4.72520	4.99973	8.31131
H	7.10453	10.38854	2.70999
H	7.40055	9.09573	1.53259
H	7.77331	8.83207	3.24400
H	1.32431	8.73357	0.96934
H	-0.23332	7.99199	0.58766
H	1.17145	6.97774	0.93665
H	0.77161	11.07518	3.93483
H	-0.50478	10.93080	2.72070
H	1.17004	10.54481	2.30522
H	6.38814	6.74276	3.14840
H	6.03568	7.08365	1.44538
H	4.70801	6.79466	2.57543
H	6.71654	10.97176	7.32538
H	5.07297	11.48295	7.79216
H	5.80680	12.10831	6.29792
H	-0.68353	9.00575	6.87379
H	-1.73640	9.90926	5.77932
H	-0.04787	10.38755	5.98492
H	0.41190	5.14082	2.10409
H	-1.32209	5.25021	2.43076
H	-0.22033	4.61400	3.65912

H	3.65664	9.03285	1.98830
H	4.93923	9.25895	0.77751
H	4.64050	10.51349	1.99963
H	-0.75482	5.34302	5.76166
H	-2.23111	6.31134	5.66969
H	-0.93891	6.76487	6.78737
H	2.18425	6.58975	5.55363
H	2.61164	8.79735	5.19828
H	2.94333	6.97122	3.65918

61

Int 9'

C	-2.70727	0.84278	-0.90184
C	-2.96780	0.17230	0.36037
C	-3.03196	-1.22627	0.10909
C	-2.80995	-1.44152	-1.31129
C	-2.65698	-0.15424	-1.92626
Ir	-0.98520	-0.51442	-0.46654
Mo	1.41003	-0.41732	0.51520
N	1.00463	-0.80332	2.38378
C	-0.02035	-1.55392	3.06663
C	-3.23439	0.86115	1.65961
C	-3.39527	-2.29411	1.08934
C	-2.93186	-2.75151	-2.02150
C	-2.51165	0.10278	-3.39169
C	-2.67418	2.32342	-1.10668
C	2.60125	0.60699	1.96432
N	2.85783	1.94778	1.56424
C	3.48036	2.91925	2.43601
C	1.69409	0.24266	3.11133
N	2.63825	0.86569	-0.44062
C	3.01208	2.06267	0.20040
O	3.42570	3.06833	-0.35514
C	3.08138	0.66952	-1.85417
C	2.91265	-0.81585	-2.20662
C	2.25243	1.54006	-2.80591
C	4.57855	0.98906	-2.00062
H	1.00471	1.05777	3.37424
H	2.17484	-0.11234	4.03988
H	3.53050	-0.00078	2.00287
H	-0.45801	-2.29903	2.40435
H	-0.81668	-0.89024	3.43442
H	0.41524	-2.06775	3.93516
H	4.77989	2.04481	-1.83215
H	4.90287	0.72040	-3.01118
H	5.16437	0.39867	-1.28923
H	-1.94046	1.01212	-3.58462
H	-3.49852	0.22317	-3.85388
H	-2.00537	-0.72208	-3.89557
H	-2.24154	2.83673	-0.24623

H	-3.68759	2.71592	-1.25611
H	-2.07973	2.59327	-1.98093
H	3.53918	-1.44353	-1.56401
H	3.22434	-0.98276	-3.24231
H	1.87634	-1.16000	-2.13078
H	4.45654	2.56879	2.80019
H	2.84069	3.12681	3.29859
H	3.62719	3.83840	1.86836
H	-3.03244	0.20675	2.50881
H	-4.28408	1.17336	1.71958
H	-2.61631	1.75340	1.77344
H	-2.34677	-2.76090	-2.94267
H	-3.97702	-2.95512	-2.28567
H	-2.57852	-3.57626	-1.40011
H	1.19057	1.29297	-2.72216
H	2.56616	1.37607	-3.84218
H	2.39177	2.59288	-2.55811
H	-2.85352	-3.22106	0.89219
H	-4.46729	-2.51705	1.02781
H	-3.17866	-1.99081	2.11457
H	-0.03679	-1.79885	0.02420
H	-0.02797	0.64613	0.43605
H	0.19816	-0.44160	-1.54766

61

TS Int 9' - Complex 5'

C	-0.64720	8.46237	4.98743
C	-0.47860	7.10764	4.51966
C	0.18922	7.17243	3.24010
C	0.44389	8.54074	2.92061
C	-0.05359	9.33719	4.02105
Ir	1.51983	7.98880	4.85416
Mo	3.98846	8.21623	5.65686
N	4.08761	8.56263	7.46014
C	3.89335	8.55372	8.87401
C	-1.06339	5.87989	5.14151
C	0.45683	5.99788	2.35610
C	1.00823	9.07171	1.64233
C	-0.06699	10.83156	4.06578
C	-1.40233	8.87867	6.20866
C	5.60075	9.56238	5.74307
N	5.69880	10.14478	4.45957
C	6.56253	11.28875	4.22552
C	4.81727	10.01175	6.90985
N	5.17610	7.92414	3.97492
C	5.67308	9.13741	3.47763
O	6.03690	9.35264	2.33359
C	5.37772	6.72136	3.11405
C	5.02195	5.47074	3.92482
C	4.48497	6.79002	1.86883

C	6.85728	6.58738	2.71270
H	4.09236	10.80858	6.75677
H	5.42097	10.18551	7.80400
H	6.51804	9.04073	6.06012
H	3.50861	7.57033	9.15489
H	3.15826	9.30755	9.18639
H	4.82847	8.73369	9.42496
H	7.18294	7.42214	2.09496
H	6.99896	5.65895	2.15035
H	7.48696	6.53915	3.60686
H	-0.09294	11.20114	5.09206
H	-0.95217	11.21927	3.54739
H	0.81436	11.25386	3.58022
H	-1.32896	8.12771	6.99696
H	-2.46463	9.01646	5.97362
H	-1.02495	9.81972	6.61227
H	5.60992	5.42400	4.84680
H	5.24741	4.57861	3.33222
H	3.96425	5.43964	4.19168
H	7.58845	11.10576	4.57742
H	6.16538	12.16706	4.73988
H	6.59153	11.47972	3.15315
H	-0.46911	4.99440	4.90958
H	-2.08252	5.70455	4.77496
H	-1.10996	5.96701	6.22842
H	0.20581	9.30277	0.93048
H	1.67712	8.35215	1.16803
H	1.58146	9.98603	1.80559
H	3.44186	6.89773	2.17752
H	4.58308	5.87294	1.27817
H	4.76225	7.64323	1.24954
H	1.27808	6.19086	1.66541
H	-0.43448	5.76844	1.76020
H	0.70811	5.10834	2.93603
H	1.74778	7.70389	6.41490
H	2.66813	9.28311	4.99139
H	2.73756	6.85731	4.81008

61

Complex 5'

Ir	1.68954	7.81049	4.44465
Mo	4.20504	7.65225	5.28416
O	6.24239	10.96027	3.95720
N	5.59357	10.08505	5.96747
N	5.42970	8.77434	4.08214
N	4.83811	6.10681	5.65538
C	4.14117	8.53640	7.25830
H	3.36356	9.29588	7.27693
H	4.11036	7.83237	8.08325
C	-0.31068	6.80901	4.68308

C	0.06538	6.74917	3.31208
C	-0.11430	8.99405	3.90332
C	0.20395	8.10663	2.81858
C	5.81057	10.01079	4.59578
C	5.38695	8.84659	6.63604
H	6.28994	8.34352	6.98981
C	5.81758	8.48349	2.67176
C	-0.41117	8.20646	5.06493
C	5.28968	4.77455	5.84607
H	6.07178	4.51709	5.12190
H	4.46400	4.06126	5.74089
H	5.70833	4.66671	6.85372
C	7.32593	8.71097	2.46806
H	7.59481	9.75436	2.62140
H	7.60649	8.42052	1.45051
H	7.89774	8.09209	3.16694
C	0.44146	8.49914	1.39596
H	0.89872	9.48716	1.32534
H	-0.50342	8.52687	0.83916
H	1.10370	7.79410	0.89044
C	-0.19817	10.48400	3.81205
H	0.00390	10.95605	4.77449
H	-1.20265	10.78566	3.49304
H	0.51734	10.88155	3.09108
C	5.54875	7.00615	2.36641
H	6.07791	6.35873	3.07101
H	5.90862	6.77777	1.35879
H	4.48453	6.76161	2.39886
C	6.09807	11.22133	6.70565
H	7.06588	11.00000	7.17808
H	5.38906	11.50980	7.48717
H	6.23138	12.04712	6.00690
C	-0.90634	8.71800	6.37957
H	-0.60988	8.06207	7.20022
H	-2.00135	8.78399	6.38420
H	-0.51125	9.71230	6.59406
C	0.19748	5.51352	2.48116
H	0.96026	5.63085	1.70963
H	-0.75156	5.28858	1.98000
H	0.46835	4.64823	3.08807
C	5.00250	9.35182	1.70261
H	3.93344	9.17111	1.84844
H	5.25657	9.10701	0.66548
H	5.21042	10.40741	1.87719
C	-0.64924	5.64842	5.56240
H	-0.12907	4.74208	5.24891
H	-1.72642	5.44555	5.52863
H	-0.38046	5.84276	6.60209
H	2.35360	7.27823	5.87546

H 2.64882 9.11157 4.58950
H 2.98032 7.10561 3.69725
61
TS Int 8 - Int 9
C -3.01208 0.63798 -1.61504
C -3.52335 1.05393 -0.32841
C -3.58846 -0.11492 0.50932
C -3.08874 -1.22882 -0.23538
C -2.74278 -0.76507 -1.56582
Ir -1.44963 0.27206 -0.06280
Mo 1.06509 0.85802 0.31810
N 2.55475 -0.51345 -0.02348
C 3.33002 -0.35781 -1.13732
O 4.19885 -1.08727 -1.59366
C -4.05719 2.40964 0.00872
C -4.15365 -0.16590 1.89234
C -3.06573 -2.65412 0.21595
C -2.31778 -1.63169 -2.70766
C -2.88783 1.51423 -2.81990
N 1.73259 2.03926 1.66462
C 2.43021 2.59189 0.58131
C 2.03335 1.59773 -1.26475
N 3.02070 0.86067 -1.80747
C 3.84980 1.25554 -2.93278
C 1.22897 2.81088 2.77677
C 2.83499 -1.71834 0.81845
C 1.95421 -1.66986 2.06951
C 2.51869 -3.00156 0.03780
C 4.30069 -1.70890 1.28367
H 1.83140 2.51227 -1.83026
H 2.04861 3.08884 3.45025
H 0.50614 2.21621 3.33873
H 0.73650 3.73269 2.43710
H 4.98451 -1.78121 0.43938
H 4.48084 -2.55454 1.95504
H 4.51280 -0.78764 1.83528
H -3.69110 -0.95627 2.48557
H -5.23223 -0.36059 1.85573
H -4.00362 0.77681 2.42117
H -2.23966 -3.20310 -0.23916
H -3.99767 -3.16016 -0.06388
H -2.95664 -2.73026 1.29900
H 2.13596 -0.75681 2.64455
H 2.19554 -2.52576 2.70792
H 0.89077 -1.72108 1.82442
H 3.35026 2.06311 -3.46995
H 3.99275 0.40068 -3.59377
H 4.83580 1.59497 -2.60042
H -1.74422 -1.06744 -3.44475

H	-3.19072	-2.05850	-3.21706
H	-1.69208	-2.45908	-2.36901
H	-3.95981	2.62185	1.07495
H	-5.11998	2.48842	-0.25219
H	-3.52349	3.19408	-0.53087
H	1.47259	-2.99636	-0.28342
H	2.67656	-3.87947	0.67286
H	3.15771	-3.08671	-0.84124
H	-2.64306	2.54115	-2.54337
H	-3.83349	1.53391	-3.37474
H	-2.11005	1.15666	-3.49641
H	-0.88080	0.78136	1.38032
H	-0.16918	-0.76425	0.05724
H	2.13255	3.59649	0.26524
H	3.50888	2.41553	0.55604
H	-0.41043	1.47141	-0.68918

61

Int 9

C	-3.26363	0.89299	-0.64514
C	-3.28408	0.16516	0.57884
C	-2.98443	-1.22549	0.28125
C	-2.80621	-1.33888	-1.13496
C	-2.94387	-0.03133	-1.71694
Ir	-1.23409	0.01846	-0.30752
Mo	1.19319	0.73200	0.20500
N	2.48541	-0.70276	0.82811
C	3.39460	-1.19831	-0.10320
O	4.14910	-2.15423	0.04352
C	-3.66004	0.69783	1.92406
C	-3.02667	-2.35290	1.26203
C	-2.57331	-2.60783	-1.88952
C	-2.95006	0.28658	-3.17780
C	-3.60500	2.33784	-0.82130
N	2.07503	2.34292	0.57738
C	3.00884	1.96371	-0.52972
C	2.51241	0.64605	-1.33499
N	3.37832	-0.46841	-1.28216
C	4.29271	-0.78583	-2.35226
C	2.18860	3.66026	1.13037
C	2.56061	-1.30686	2.19515
C	1.62801	-0.54523	3.14113
C	2.12357	-2.77855	2.15871
C	3.98369	-1.17488	2.76829
H	2.28193	0.92011	-2.36607
H	3.16879	3.80720	1.60380
H	1.41557	3.80194	1.88833
H	2.06433	4.43269	0.35796
H	4.70370	-1.72865	2.16902
H	4.00662	-1.55968	3.79298

H	4.28073	-0.12124	2.79807
H	-2.39266	-3.18228	0.94513
H	-4.04958	-2.73404	1.36899
H	-2.68498	-2.03681	2.24924
H	-1.99893	-2.43101	-2.79999
H	-3.53110	-3.05702	-2.17720
H	-2.02749	-3.33686	-1.28915
H	1.86926	0.52257	3.16327
H	1.75085	-0.93951	4.15505
H	0.57844	-0.65827	2.86171
H	5.10019	-0.04591	-2.43564
H	3.76252	-0.83106	-3.31002
H	4.73381	-1.75851	-2.13610
H	-2.62507	1.31168	-3.36446
H	-3.95791	0.17401	-3.59588
H	-2.28271	-0.37539	-3.73215
H	-3.11571	0.18955	2.72171
H	-4.73165	0.55252	2.10648
H	-3.44893	1.76514	2.00638
H	1.11047	-2.85578	1.75222
H	2.12038	-3.19924	3.17031
H	2.79967	-3.36327	1.53568
H	-3.38017	2.91442	0.07717
H	-4.67392	2.45347	-1.03705
H	-3.04855	2.78199	-1.64815
H	-0.32575	-0.09261	1.09949
H	0.02987	-0.84009	-0.87656
H	3.01183	2.77832	-1.25942
H	4.01307	1.76959	-0.13736
H	-0.32062	1.27777	-0.92906

61

TS Int 9 - Complex 5

C	-3.37945	1.06068	-1.03333
C	-3.44540	0.46973	0.26158
C	-3.18754	-0.95319	0.11430
C	-2.99420	-1.22258	-1.27806
C	-3.07931	0.02094	-1.99733
Ir	-1.38697	0.15603	-0.56779
Mo	1.04810	0.89900	-0.07853
N	2.35577	-0.57311	0.45911
C	3.36262	-0.92730	-0.43718
O	4.13836	-1.86981	-0.32742
C	-3.82739	1.15393	1.53487
C	-3.27379	-1.96858	1.20797
C	-2.79761	-2.57158	-1.89090
C	-3.06065	0.18126	-3.48389
C	-3.66337	2.49019	-1.36548
N	1.71054	2.44941	0.53155
C	2.62301	2.24565	-1.11791

C	2.42742	0.91217	-1.67367
N	3.44674	-0.06448	-1.51969
C	4.34449	-0.35021	-2.61227
C	2.09460	3.69526	1.10318
C	2.39529	-1.30100	1.77019
C	1.36537	-0.71208	2.73838
C	2.06542	-2.78869	1.57118
C	3.77209	-1.13012	2.43985
H	1.91145	0.88544	-2.63564
H	3.16597	3.72295	1.34166
H	1.53593	3.82386	2.03504
H	1.85755	4.54173	0.44536
H	4.56535	-1.56990	1.83881
H	3.76706	-1.61328	3.42229
H	3.98551	-0.06642	2.58906
H	-2.65061	-2.83859	0.99526
H	-4.30679	-2.31572	1.33151
H	-2.94580	-1.55454	2.16302
H	-2.17122	-2.51824	-2.78252
H	-3.76409	-2.99890	-2.18302
H	-2.32298	-3.26225	-1.19266
H	1.50522	0.36538	2.86677
H	1.49290	-1.19087	3.71472
H	0.34050	-0.89021	2.40909
H	4.98076	0.51389	-2.83669
H	3.79621	-0.62196	-3.52521
H	4.97090	-1.19057	-2.31346
H	-2.70069	1.16988	-3.77444
H	-4.06687	0.05612	-3.90268
H	-2.40938	-0.55689	-3.95524
H	-3.31686	0.71396	2.39322
H	-4.90702	1.06885	1.70881
H	-3.57559	2.21536	1.50990
H	1.08728	-2.89071	1.09108
H	2.02738	-3.30045	2.53919
H	2.81687	-3.26941	0.94667
H	-3.42195	3.15077	-0.53144
H	-4.72613	2.62063	-1.60191
H	-3.08669	2.82158	-2.23048
H	-0.49308	0.08052	0.84378
H	-0.14965	-0.74108	-1.12274
H	2.19899	3.08473	-1.66039
H	3.60258	2.42005	-0.67891
H	-0.43498	1.39136	-1.22248

61

Complex 5

C	-3.26324	1.05655	-0.70995
C	-3.29417	0.50414	0.60248
C	-3.07899	-0.92817	0.48873

C	-2.93527	-1.23976	-0.90133
C	-3.02332	-0.01507	-1.65414
Ir	-1.26920	0.10952	-0.29556
Mo	1.29095	0.66516	0.02641
N	2.66025	-0.76561	0.65745
C	3.87696	-0.83726	-0.01300
O	4.86538	-1.47272	0.33013
C	-3.60769	1.23552	1.86794
C	-3.16158	-1.91005	1.61276
C	-2.80205	-2.61077	-1.48212
C	-3.06357	0.10417	-3.14410
C	-3.52136	2.48354	-1.07207
N	1.81799	1.95345	1.02681
C	2.05841	1.56090	-1.82595
C	2.59589	0.26082	-1.63735
N	3.88971	-0.05304	-1.16682
C	4.95182	-0.26612	-2.12958
C	2.35471	2.94988	1.87985
C	2.61756	-1.48374	1.96899
C	1.24670	-1.30590	2.62335
C	2.82178	-2.99281	1.74808
C	3.67176	-0.92852	2.94233
H	2.10448	-0.54403	-2.20241
H	3.22502	2.56535	2.42503
H	1.60762	3.29480	2.60447
H	2.67798	3.81420	1.28622
H	4.67787	-1.06951	2.55098
H	3.59387	-1.43895	3.90814
H	3.50075	0.13907	3.11165
H	-2.60125	-2.81984	1.39195
H	-4.20429	-2.19577	1.79744
H	-2.76251	-1.49333	2.53911
H	-2.22935	-2.59880	-2.41053
H	-3.79307	-3.02364	-1.70394
H	-2.30198	-3.29136	-0.79182
H	1.03743	-0.25799	2.85813
H	1.23135	-1.86434	3.56430
H	0.44556	-1.68666	1.98620
H	5.14455	0.65898	-2.67842
H	4.70161	-1.05808	-2.85121
H	5.84701	-0.56557	-1.58536
H	-2.68252	1.07071	-3.47866
H	-4.09144	0.00500	-3.51444
H	-2.46140	-0.66985	-3.62301
H	-3.09301	0.79293	2.72236
H	-4.68436	1.20337	2.07390
H	-3.30978	2.28352	1.80904
H	2.05673	-3.37576	1.06551
H	2.72627	-3.52464	2.70083

H 3.80467 -3.19747 1.32794
H -3.24243 3.15980 -0.26288
H -4.58681 2.63276 -1.28326
H -2.96133 2.77869 -1.96076
H -0.38111 0.22957 1.09028
H -0.09109 -0.84862 -0.87493
H 1.31982 1.71657 -2.60556
H 2.66216 2.42867 -1.58002
H -0.36968 1.37828 -0.86710
75

Int 10
C -3.23742 -0.40109 -1.91489
C -1.94509 -0.19286 -2.48811
C -1.60658 1.20307 -2.33329
C -2.71176 1.84548 -1.65853
C -3.70046 0.84657 -1.40285
Ir -1.63289 0.34559 -0.31444
Mo -0.13253 0.07965 1.28607
N 0.42974 1.85119 1.82171
C 0.15152 3.15584 1.24819
C -1.14711 -1.20039 -3.25106
C -0.44356 1.89582 -2.96674
C -2.87359 3.31617 -1.44730
C -5.01230 1.07728 -0.72433
C -3.99268 -1.69097 -1.89980
N -0.95801 -1.12995 2.74540
C -2.08789 -1.01938 3.69884
C -2.33541 0.46573 3.96242
N 0.55015 -2.06108 1.32103
C 1.89965 -2.24103 1.89540
C -0.49281 -2.33007 2.37538
O -0.77645 -3.46547 2.69437
C 0.37251 -2.95938 0.16455
C 1.36624 1.95816 2.93316
C -3.34113 -1.64993 3.08150
C -1.72194 -1.71031 5.01966
N 6.18034 -1.12510 -1.89616
C 7.20032 -0.21804 -2.41734
C 6.60461 0.58620 -3.57661
C 5.09123 -1.12035 -1.40218
O 4.03016 -1.24471 -0.91309
C 7.66076 0.71092 -1.29011
C 8.36357 -1.08210 -2.90722
H 1.55449 0.98057 3.38619
H 0.96331 2.61837 3.71502
H 2.33008 2.37440 2.60587
H 1.07210 3.62074 0.86286
H -0.26261 3.82621 2.01589

H	-0.57008	3.04926	0.44185
H	-1.44529	0.94003	4.38704
H	-3.15780	0.58340	4.67435
H	-2.59398	0.98721	3.03769
H	-3.55809	-1.16858	2.12337
H	-4.20035	-1.51641	3.74629
H	-3.19331	-2.71840	2.91357
H	-0.62178	-2.80227	-0.25019
H	0.49336	-4.00246	0.47439
H	1.11802	-2.70283	-0.58969
H	-1.54400	-2.77563	4.87030
H	-2.53889	-1.58894	5.73754
H	-0.82242	-1.26017	5.45124
H	2.64763	-1.95495	1.15430
H	2.04814	-3.28753	2.18538
H	2.00961	-1.61193	2.78069
H	-0.07549	-1.01074	-3.16273
H	-1.40502	-1.17552	-4.31770
H	-1.33365	-2.21487	-2.89212
H	-0.14585	2.77826	-2.39658
H	-0.69018	2.22175	-3.98580
H	0.42411	1.23566	-3.02766
H	-3.31942	-2.55074	-1.88873
H	-4.63136	-1.78466	-2.78764
H	-4.63805	-1.77110	-1.02227
H	-5.33601	0.19644	-0.16514
H	-5.79736	1.30995	-1.45506
H	-4.95913	1.91229	-0.02226
H	-3.44878	3.53439	-0.54478
H	-3.40054	3.77501	-2.29417
H	-1.90840	3.81840	-1.35450
H	7.36327	1.24603	-4.00679
H	6.24271	-0.08097	-4.36253
H	5.76912	1.20319	-3.23447
H	9.16028	-0.45153	-3.31016
H	8.77194	-1.67684	-2.08674
H	8.03053	-1.76543	-3.69197
H	8.45188	1.37558	-1.64819
H	6.83237	1.32760	-0.93080
H	8.05163	0.13185	-0.45001

77

TS Int 10 - Int 11

O	0.94071	-3.72969	0.84913
Mo	0.82448	0.03593	1.46013
N	2.75319	-0.00540	1.33245
C	3.50169	-0.39190	2.52185
Ir	0.10341	0.89952	-0.44435
C	0.40172	0.59334	-2.61557
C	0.61954	1.99807	-2.37800

C	-0.62823	2.56962	-1.96967
C	-1.61308	1.54269	-1.93951
C	-0.98333	0.31455	-2.32630
C	1.87514	2.75715	-2.66459
C	-0.85463	4.01089	-1.64592
C	-3.06219	1.72113	-1.62000
C	-1.67035	-0.99372	-2.55316
C	1.36648	-0.35437	-3.25032
N	-0.00068	1.17721	2.97408
C	-1.07768	0.44634	3.28599
O	-2.07026	0.66987	3.94681
N	-0.90630	-0.87265	2.57417
C	-2.15988	-1.27537	1.90979
C	1.00659	-4.26293	-0.19622
N	1.01119	-4.70754	-1.30673
C	1.45018	-5.88526	-2.05400
C	0.95687	-5.71875	-3.49213
C	0.10274	2.59173	3.40609
C	-1.02629	3.40481	2.76292
C	0.03554	2.67338	4.93678
C	1.45354	3.12551	2.93049
C	2.97971	-5.95191	-2.01618
C	0.83524	-7.13615	-1.41920
C	-0.49782	-1.90977	3.54462
C	3.63984	0.18617	0.19906
H	2.84005	-0.50253	3.38583
H	4.25059	0.37441	2.76990
H	4.03047	-1.34394	2.37136
H	4.22101	-0.72680	-0.00283
H	4.35464	0.99581	0.40812
H	3.05182	0.44520	-0.67827
H	2.27643	2.55827	3.37626
H	1.56289	4.17369	3.22416
H	1.53435	3.05183	1.84280
H	-0.98201	3.28981	1.67580
H	-0.92014	4.46532	3.01190
H	-2.00056	3.06021	3.11473
H	-2.43830	-0.50975	1.18749
H	-2.95569	-1.40385	2.65066
H	-1.98452	-2.21380	1.38119
H	-0.91977	2.30157	5.30834
H	0.15503	3.71320	5.25625
H	0.84149	2.08566	5.38725
H	-0.28071	-2.83529	3.00959
H	-1.29754	-2.07935	4.27463
H	0.40003	-1.58582	4.07404
H	-0.99484	-1.83377	-2.37929
H	-2.03967	-1.06731	-3.58443
H	-2.52686	-1.11615	-1.88676

H	2.40086	-0.07079	-3.04616
H	1.23715	-0.36725	-4.34065
H	1.22284	-1.37223	-2.88325
H	-3.63318	1.99293	-2.51729
H	-3.21904	2.50829	-0.87937
H	-3.50039	0.80445	-1.21945
H	-1.67114	4.13966	-0.93224
H	-1.11353	4.57933	-2.54835
H	0.03726	4.47016	-1.21345
H	2.75844	2.12105	-2.57865
H	2.00418	3.59009	-1.97003
H	1.86295	3.17023	-3.68169
H	3.33359	-6.80600	-2.60032
H	3.41573	-5.04243	-2.43600
H	3.33933	-6.06655	-0.98992
H	1.25988	-6.57762	-4.09651
H	-0.13267	-5.64240	-3.51848
H	1.37431	-4.81399	-3.93994
H	1.12242	-8.02606	-1.98613
H	1.18059	-7.26110	-0.38916
H	-0.25536	-7.06892	-1.41389

77

Int 11

C	-2.56384	1.78963	-0.43165
C	-1.81912	1.74995	-1.65426
C	-1.98147	0.44468	-2.22832
C	-2.83938	-0.31451	-1.36568
C	-3.19339	0.51502	-0.25714
Ir	-0.90195	0.22974	-0.24714
Mo	0.91797	-0.52587	0.80687
O	2.50154	-0.12376	-0.73106
C	2.60134	0.76109	-1.52825
N	2.75476	1.54581	-2.39002
C	3.33328	2.76289	-2.94684
C	2.95495	3.93784	-2.04200
C	-1.45352	0.00604	-3.55632
C	-1.12659	2.91006	-2.29115
C	-3.35698	-1.69253	-1.62275
C	-4.15465	0.16257	0.83116
C	-2.77443	2.99143	0.43145
N	1.24922	-2.68038	0.08502
C	2.64282	-3.15562	0.13166
N	0.21214	-2.09096	2.01460
C	-0.64417	-2.21566	3.21848
C	-0.85460	-0.82298	3.81019
N	1.37129	0.93950	1.98602
C	0.80429	2.25807	2.18756
C	0.59361	-3.16597	-1.14279
C	0.51248	-3.17313	1.29739

O	0.29795	-4.36068	1.44977
C	2.52245	0.72851	2.85528
C	-2.00397	-2.80250	2.81837
C	0.04729	-3.11100	4.25611
C	4.85303	2.57725	-3.00036
C	2.76297	2.94997	-4.35343
H	2.93486	-0.27623	2.72336
H	2.24096	0.84053	3.91238
H	3.31857	1.45799	2.64468
H	1.56252	3.03936	2.01912
H	0.44576	2.36708	3.22227
H	-0.02674	2.39401	1.49796
H	0.09124	-0.39207	4.14998
H	-1.52855	-0.88695	4.66984
H	-1.28832	-0.15291	3.06300
H	-2.46148	-2.16857	2.05513
H	-2.66883	-2.84304	3.68705
H	-1.88865	-3.81165	2.41979
H	-0.43709	-2.81468	-1.15026
H	0.62864	-4.26035	-1.18684
H	1.10968	-2.74029	-2.00564
H	0.19954	-4.11657	3.86223
H	-0.56760	-3.17756	5.15929
H	1.01841	-2.69135	4.53649
H	3.19261	-2.74655	-0.71788
H	2.67341	-4.25005	0.09811
H	3.11689	-2.81725	1.05580
H	-4.19544	-0.91528	0.99971
H	-5.16994	0.49348	0.57529
H	-3.88408	0.63425	1.77850
H	-2.69271	-2.25315	-2.28378
H	-4.34386	-1.66025	-2.10262
H	-3.45709	-2.26369	-0.69693
H	-2.92813	2.71233	1.47623
H	-3.65740	3.55879	0.10830
H	-1.91893	3.67040	0.39375
H	-0.70636	3.58193	-1.53867
H	-1.82035	3.49509	-2.91010
H	-0.31053	2.57592	-2.93563
H	-1.32008	-1.07706	-3.59564
H	-0.48332	0.46231	-3.76648
H	-2.13888	0.28487	-4.36776
H	3.36474	4.86357	-2.45458
H	1.87054	4.03754	-1.97120
H	3.35786	3.80021	-1.03568
H	3.19015	3.84719	-4.80789
H	3.00366	2.09261	-4.98614
H	1.67765	3.06270	-4.32027
H	5.31676	3.46802	-3.43192

H	5.26384	2.42740	-1.99870
H	5.11832	1.71691	-3.61927

77

TS Int 11 - Int 12

C	1.75672	1.20541	6.56547
C	2.66172	2.28173	6.84821
C	1.90576	3.35970	7.41973
C	0.53838	2.94970	7.49895
C	0.44781	1.61763	6.97789
Ir	1.80976	1.54422	8.80667
Mo	2.25111	0.71858	10.83309
N	4.06719	-0.13817	10.59050
C	4.72589	-0.64495	11.79576
C	4.10565	2.34360	6.46857
C	2.43977	4.71560	7.74584
C	-0.60568	3.80173	7.94392
C	-0.80658	0.82316	6.81175
C	2.09348	-0.06477	5.85454
N	1.87699	2.27123	12.17026
C	0.60720	2.06079	12.51631
O	-0.19679	2.69602	13.16595
O	1.75595	-1.37804	10.61557
C	2.80304	-1.75567	10.08330
N	3.51263	-2.55894	9.51666
C	3.17037	-3.91988	9.04205
C	1.88511	-3.87334	8.21303
N	0.24666	0.73863	11.90030
C	-1.02838	0.82679	11.16646
C	2.57401	3.53213	12.49303
C	2.63085	3.71991	14.01400
C	3.99222	3.41010	11.93434
C	1.85044	4.70567	11.82442
C	4.34124	-4.40074	8.18697
C	2.99537	-4.82477	10.26525
C	0.14354	-0.28137	12.96304
C	5.00735	-0.05015	9.48495
H	4.01350	-0.73909	12.62037
H	5.52041	0.04291	12.11088
H	5.17801	-1.62765	11.60744
H	5.47283	-1.02710	9.30008
H	5.79273	0.67625	9.72958
H	4.47573	0.28650	8.59693
H	4.53112	2.58846	12.41780
H	4.55344	4.33200	12.11117
H	3.96136	3.22498	10.85477
H	1.77388	4.51700	10.75083
H	2.40315	5.63670	11.98484
H	0.84571	4.82064	12.23511
H	-0.94513	1.60648	10.41141

H	-1.84754	1.05151	11.85791
H	-1.20806	-0.12627	10.66650
H	1.62649	3.79696	14.43269
H	3.18183	4.63404	14.25651
H	3.14433	2.87652	14.48571
H	-0.04164	-1.25494	12.50831
H	-0.67159	-0.02736	13.64866
H	1.07933	-0.32406	13.52420
H	3.10851	-0.39583	6.08413
H	2.02134	0.06230	4.76637
H	1.41243	-0.86960	6.13884
H	4.68642	2.91615	7.19542
H	4.22969	2.82498	5.48995
H	4.54764	1.34725	6.40011
H	-0.61805	-0.24678	6.92325
H	-1.24479	0.98448	5.81846
H	-1.55748	1.10313	7.55323
H	-1.44187	3.19696	8.30089
H	-0.97902	4.41804	7.11587
H	-0.31819	4.47498	8.75441
H	1.82821	5.21861	8.49691
H	2.45404	5.35267	6.85190
H	3.46060	4.66322	8.13040
H	1.64068	-4.87377	7.84376
H	1.04351	-3.51223	8.80941
H	2.00766	-3.20957	7.35412
H	2.79813	-5.85319	9.94846
H	3.90009	-4.82299	10.87866
H	2.15711	-4.49146	10.88314
H	4.15174	-5.41062	7.81249
H	4.48865	-3.73593	7.33248
H	5.26388	-4.41457	8.77249
77			
Int 12			
C	-0.24978	0.34589	-3.15936
C	0.64764	1.40955	-2.80104
C	-0.12798	2.46865	-2.20554
C	-1.49084	2.04663	-2.17023
C	-1.56134	0.73742	-2.75177
Ir	-0.18928	0.60951	-0.88688
Mo	0.37945	-0.21609	1.09266
N	2.20765	-1.39015	0.65608
C	2.99945	-1.61600	1.88728
C	2.09018	1.50509	-3.17934
C	0.38684	3.82336	-1.84505
C	-2.65133	2.86615	-1.70673
C	-2.80621	-0.06564	-2.94935
C	0.10299	-0.89157	-3.91893
N	-0.12517	1.24893	2.49377

C	-1.38458	0.92418	2.78066
O	-2.27766	1.46551	3.39434
O	0.14841	-2.28316	0.88940
C	1.32972	-2.57616	0.41600
N	1.82742	-3.59200	-0.13624
C	1.02833	-4.79535	-0.41478
C	-0.13677	-4.46749	-1.35824
N	-1.56905	-0.42275	2.13740
C	-2.82565	-0.50133	1.37437
C	0.45495	2.55272	2.86740
C	0.43795	2.71071	4.39211
C	1.89953	2.54730	2.36385
C	-0.32960	3.68123	2.19117
C	1.97394	-5.78877	-1.09710
C	0.50305	-5.40768	0.89119
C	-1.54164	-1.47090	3.18033
C	3.10645	-1.08379	-0.47039
H	2.33611	-1.88794	2.71016
H	3.53610	-0.70132	2.14802
H	3.71412	-2.42832	1.71899
H	3.82612	-1.89661	-0.60229
H	3.62454	-0.14621	-0.25843
H	2.50611	-0.95835	-1.36723
H	2.47635	1.75447	2.85269
H	2.38856	3.50154	2.57951
H	1.92362	2.38974	1.27886
H	-0.35734	3.50432	1.11309
H	0.14573	4.64814	2.38380
H	-1.35349	3.71367	2.56806
H	-2.82596	0.28868	0.62445
H	-3.68111	-0.38990	2.04815
H	-2.86150	-1.46825	0.87070
H	-0.58561	2.69980	4.76932
H	0.90459	3.65962	4.67428
H	0.99657	1.89971	4.86920
H	-1.55370	-2.44844	2.70009
H	-2.40712	-1.35696	3.84054
H	-0.62865	-1.37435	3.77107
H	1.12506	-1.21460	-3.71069
H	0.02059	-0.72559	-5.00098
H	-0.55920	-1.71977	-3.65941
H	2.66618	2.05981	-2.43532
H	2.20160	2.02399	-4.13994
H	2.54491	0.51850	-3.28744
H	-2.60795	-1.13651	-2.86741
H	-3.23587	0.11831	-3.94205
H	-3.56956	0.18745	-2.21059
H	-3.47700	2.23838	-1.36477
H	-3.03511	3.49565	-2.51973

H	-2.37621	3.52355	-0.87970
H	-0.22518	4.29541	-1.07457
H	0.38012	4.48436	-2.72151
H	1.41307	3.77937	-1.47424
H	-0.70147	-5.37500	-1.59440
H	-0.81313	-3.74009	-0.90549
H	0.24222	-4.04918	-2.29472
H	-0.01932	-6.34795	0.68863
H	1.33244	-5.62105	1.57238
H	-0.18988	-4.72645	1.38892
H	1.45372	-6.71965	-1.34327
H	2.37440	-5.35956	-2.01955
H	2.81748	-6.02369	-0.44204

77

TS Int 12 - Int 13'

C	-1.52911	-0.17846	-2.59251
C	-0.28978	0.23159	-3.13301
C	0.01081	1.54278	-2.61713
C	-1.09582	1.98058	-1.81287
C	-2.02407	0.89083	-1.74674
Ir	-0.14507	0.27220	-0.75144
Mo	0.66735	-0.16741	1.31619
N	2.69385	-0.15128	0.45631
C	3.79589	-0.67790	1.28620
C	0.60584	-0.53081	-4.05345
C	1.14746	2.40202	-3.07031
C	-1.31031	3.36247	-1.28850
C	-3.38927	0.94118	-1.13969
C	-2.28675	-1.43040	-2.89392
N	-0.18520	1.02891	2.80100
C	-1.22115	0.30247	3.23446
O	-2.11954	0.52021	4.01735
C	1.93383	-1.25205	-0.23412
N	2.54775	-2.01074	-1.04032
C	2.13557	-3.35504	-1.43634
C	0.61878	-3.50007	-1.58973
N	-1.08457	-0.99587	2.51013
C	-2.34528	-1.39231	1.86393
C	0.06361	2.40804	3.25890
C	0.32237	2.41362	4.77012
C	1.30884	2.88956	2.51171
C	-1.13076	3.30279	2.91137
C	2.82687	-3.66541	-2.76884
C	2.64355	-4.34271	-0.37099
C	-0.65033	-2.04965	3.45082
C	3.21188	0.83744	-0.49339
O	1.24779	-1.90145	1.15573
H	3.40990	-1.43919	1.96144
H	4.23463	0.14252	1.85886

H	4.55463	-1.12713	0.63783
H	4.00836	0.38523	-1.09420
H	3.60438	1.69591	0.05661
H	2.39382	1.15672	-1.13697
H	2.17901	2.27654	2.77267
H	1.54087	3.92668	2.76918
H	1.14855	2.83501	1.42799
H	-1.31378	3.27720	1.83446
H	-0.92876	4.33730	3.20544
H	-2.02868	2.96150	3.42891
H	-2.64823	-0.61257	1.16847
H	-3.12484	-1.54756	2.61738
H	-2.17487	-2.31469	1.30700
H	-0.55279	2.04908	5.31058
H	0.54388	3.42945	5.11166
H	1.17662	1.77598	5.01644
H	-0.39882	-2.94557	2.88296
H	-1.44704	-2.26341	4.17078
H	0.23910	-1.71803	3.98950
H	-1.66234	-2.17517	-3.38835
H	-3.13478	-1.21683	-3.55651
H	-2.68554	-1.88900	-1.98613
H	1.55936	-0.77015	-3.57088
H	0.82158	0.04905	-4.95766
H	0.14989	-1.47013	-4.37028
H	-3.74401	-0.05442	-0.86531
H	-4.11082	1.35813	-1.85380
H	-3.41202	1.56535	-0.24388
H	-1.98567	3.36247	-0.43156
H	-1.75625	4.00318	-2.06040
H	-0.37315	3.82739	-0.97467
H	1.44847	3.11693	-2.30101
H	0.85822	2.97913	-3.95787
H	2.02230	1.80696	-3.34129
H	0.36574	-4.51045	-1.92792
H	0.11682	-3.30581	-0.64019
H	0.24100	-2.78433	-2.32053
H	2.40539	-5.37191	-0.66082
H	3.72854	-4.25788	-0.26290
H	2.17871	-4.13157	0.59348
H	2.62499	-4.69506	-3.08052
H	2.47559	-2.99285	-3.55563
H	3.90817	-3.53625	-2.67164

77

Int 13'

C	-1.61566	-0.58240	-2.90672
C	-0.41922	0.02872	-3.29783
C	-0.26699	1.25561	-2.54172
C	-1.45903	1.44170	-1.74449

C	-2.24724	0.26585	-1.90850
Ir	-0.28845	-0.23397	-0.85709
Mo	0.32776	-0.19970	1.43505
N	2.32353	-0.09964	0.39085
C	3.46749	-0.69753	1.10071
C	0.53823	-0.42666	-4.34972
C	0.72249	2.32584	-2.87393
C	-1.86475	2.71052	-1.06484
C	-3.65391	0.05745	-1.44542
C	-2.23551	-1.82865	-3.44945
N	-0.28566	1.27689	2.79166
C	-1.41043	0.77068	3.32046
O	-2.15144	1.14950	4.20122
C	1.61476	-1.05199	-0.58571
N	2.34889	-2.02071	-0.91272
C	2.01213	-3.26065	-1.60164
C	0.52974	-3.48469	-1.89535
N	-1.64023	-0.50248	2.58373
C	-2.94769	-0.46649	1.91172
C	0.29165	2.52812	3.33776
C	0.68508	2.31860	4.80594
C	1.53655	2.86083	2.52026
C	-0.71852	3.67595	3.20927
C	2.82903	-3.29533	-2.90032
C	2.51043	-4.37944	-0.67062
C	-1.60719	-1.64049	3.52761
C	2.76832	1.12316	-0.29160
O	0.85547	-1.70591	1.99968
H	3.15730	-1.63780	1.55023
H	3.79054	-0.00265	1.88115
H	4.29157	-0.88721	0.40769
H	3.51951	0.86842	-1.04932
H	3.21480	1.80846	0.43111
H	1.91726	1.59950	-0.76983
H	2.27942	2.06098	2.59566
H	1.99465	3.78244	2.88960
H	1.28408	3.00901	1.46587
H	-1.00262	3.81948	2.16284
H	-0.27326	4.60721	3.57262
H	-1.61707	3.46932	3.79106
H	-2.99262	0.40330	1.26024
H	-3.75388	-0.40924	2.65139
H	-3.05588	-1.36617	1.30597
H	-0.19183	2.08521	5.41158
H	1.15060	3.22509	5.20482
H	1.40267	1.49757	4.89489
H	-1.69978	-2.56714	2.96077
H	-2.42689	-1.55162	4.24711
H	-0.65260	-1.65655	4.05185

H	-1.55181	-2.37378	-4.10196
H	-3.12882	-1.58911	-4.03991
H	-2.54566	-2.51134	-2.65349
H	1.57549	-0.31832	-4.02420
H	0.41754	0.16995	-5.26224
H	0.38180	-1.47216	-4.62017
H	-3.81876	-0.95581	-1.07185
H	-4.34486	0.21167	-2.28369
H	-3.93804	0.76005	-0.66163
H	-2.57572	2.52780	-0.25736
H	-2.34245	3.39540	-1.77741
H	-1.00730	3.22927	-0.63147
H	0.83152	3.04835	-2.06218
H	0.38908	2.88261	-3.75936
H	1.70978	1.91708	-3.09967
H	0.38811	-4.46305	-2.36673
H	-0.05627	-3.45316	-0.97353
H	0.13921	-2.71777	-2.56212
H	2.35376	-5.36058	-1.13061
H	3.57591	-4.25435	-0.46249
H	1.97033	-4.34842	0.27898
H	2.71617	-4.26351	-3.39792
H	2.50078	-2.51440	-3.59028
H	3.88866	-3.13622	-2.68436

77

TS Int 12 - Int 13

C	-1.51818	2.53118	-1.75494
C	-2.62204	1.61663	-1.63802
C	-2.45424	0.60770	-2.66293
C	-1.29805	0.95049	-3.43614
C	-0.70687	2.11191	-2.88027
Ir	-0.80199	0.58223	-1.08811
Mo	-0.32945	-0.23648	1.05098
C	1.62552	-0.55574	0.56711
O	1.48408	-1.36352	1.55939
C	-3.84889	1.80387	-0.80257
C	-3.45156	-0.45382	-2.99646
C	-0.82634	0.21511	-4.64932
C	0.47159	2.86009	-3.41432
C	-1.38036	3.84236	-1.04972
N	0.48869	-0.99617	-0.59605
C	0.79140	-2.24434	-1.34087
C	-0.54181	-2.81197	-1.84008
C	1.68517	-1.90098	-2.54008
C	1.48531	-3.31622	-0.49251
N	-1.53527	-0.39568	2.90446
C	-2.99820	-0.30297	2.76462
N	2.84170	-0.12016	0.12869
C	4.03225	-0.44070	0.88637

N	-0.07813	1.26594	2.55414
C	-0.96150	0.86916	3.47043
O	-1.34400	1.30299	4.53782
C	-1.15487	-1.55293	3.73712
C	2.96392	0.86430	-0.92126
C	0.71418	2.49111	2.81847
C	1.63509	2.74158	1.63186
C	-0.22533	3.69412	2.98953
C	1.56069	2.29447	4.08498
H	3.81093	-1.25134	1.57873
H	4.39153	0.42407	1.46185
H	4.83319	-0.75372	0.20686
H	3.66799	0.51732	-1.68691
H	3.33042	1.82030	-0.52765
H	1.98507	1.02493	-1.37574
H	2.37254	1.94285	1.54594
H	2.16362	3.69012	1.76812
H	1.06425	2.77638	0.70140
H	-0.82225	3.83871	2.08546
H	0.36163	4.60225	3.15851
H	-0.89561	3.55124	3.83765
H	-3.24721	0.53797	2.11582
H	-3.46345	-0.15519	3.74403
H	-3.37257	-1.22205	2.30930
H	0.92652	2.15042	4.96108
H	2.19089	3.17329	4.25400
H	2.21169	1.42262	3.97399
H	-1.50987	-2.46948	3.26047
H	-1.59606	-1.46157	4.73464
H	-0.06791	-1.59490	3.81663
H	-2.99422	-1.28879	-3.53147
H	-4.24503	-0.05075	-3.63959
H	-3.92669	-0.85576	-2.09910
H	0.23316	0.39204	-4.84576
H	-1.38067	0.54459	-5.53677
H	-0.97241	-0.86397	-4.55941
H	-4.28105	0.84516	-0.50823
H	-4.61603	2.35688	-1.36018
H	-3.63144	2.36737	0.10671
H	-1.90284	3.84247	-0.09227
H	-1.80681	4.64858	-1.66045
H	-0.33519	4.09209	-0.85688
H	1.02609	3.35806	-2.61573
H	0.15667	3.63613	-4.12419
H	1.16794	2.20270	-3.94024
H	1.61892	-4.20915	-1.11178
H	2.46878	-2.99353	-0.14859
H	0.89285	-3.58235	0.38363
H	1.82516	-2.77957	-3.17741

H	1.23017	-1.10607	-3.13455
H	2.66911	-1.56658	-2.20530
H	-0.36486	-3.65087	-2.52142
H	-1.14493	-3.16668	-0.99997
H	-1.11065	-2.03968	-2.36048

77

Int 13

C	-0.83219	2.09995	-1.91668
C	-2.07528	1.46744	-1.53936
C	-2.28312	0.35795	-2.44089
C	-1.21893	0.35379	-3.39675
C	-0.31524	1.39492	-3.07325
Ir	-0.42698	0.16282	-1.07702
Mo	0.49715	-0.51392	0.93183
C	2.58175	-0.36174	1.06910
O	2.61516	-1.21206	1.98460
C	-3.07804	2.00134	-0.56640
C	-3.49092	-0.52117	-2.46906
C	-1.09229	-0.58536	-4.55300
C	0.90233	1.78819	-3.84398
C	-0.32123	3.40876	-1.40284
N	0.51158	-1.67178	-0.48048
C	0.80433	-2.88451	-1.21899
C	-0.50653	-3.47253	-1.75327
C	1.74942	-2.54112	-2.37678
C	1.47990	-3.88212	-0.26804
N	-0.99060	-1.06632	2.50144
C	-2.42006	-1.01909	2.14228
N	3.67881	0.15883	0.49813
C	5.00772	-0.21181	0.95132
N	0.27404	0.79950	2.66733
C	-0.63771	0.12098	3.36080
O	-1.17304	0.26711	4.44181
C	-0.68359	-2.31636	3.22502
C	3.57713	1.10689	-0.58822
C	0.82772	2.04416	3.24842
C	1.79441	2.66183	2.24022
C	-0.30775	3.04488	3.51540
C	1.57860	1.72874	4.54996
H	4.90923	-0.94448	1.75125
H	5.54431	0.66632	1.32760
H	5.58701	-0.64570	0.12880
H	4.11771	0.73810	-1.46764
H	4.00142	2.07710	-0.30297
H	2.52075	1.22137	-0.84037
H	2.67809	2.03562	2.10738
H	2.12200	3.64398	2.59451
H	1.31179	2.78237	1.26704
H	-0.82946	3.28418	2.58376

H	0.10280	3.97383	3.92319
H	-1.02761	2.64095	4.22788
H	-2.62787	-0.07762	1.63535
H	-3.03473	-1.10085	3.04526
H	-2.64472	-1.84107	1.46045
H	0.89811	1.32508	5.30130
H	2.03948	2.63756	4.95011
H	2.36773	0.99585	4.36063
H	-0.94177	-3.16697	2.59142
H	-1.25801	-2.36045	4.15574
H	0.38315	-2.35011	3.45232
H	-3.26302	-1.50855	-2.87662
H	-4.28098	-0.08289	-3.09251
H	-3.90256	-0.66602	-1.46833
H	-0.04926	-0.75545	-4.82913
H	-1.59961	-0.17909	-5.43662
H	-1.54342	-1.55772	-4.34351
H	-3.71975	1.20714	-0.17927
H	-3.72731	2.74192	-1.05064
H	-2.59637	2.48373	0.28583
H	-0.64069	3.58438	-0.37441
H	-0.69778	4.23662	-2.01743
H	0.76978	3.45071	-1.42151
H	1.66442	2.22118	-3.19270
H	0.65616	2.54000	-4.60498
H	1.35114	0.93597	-4.35884
H	1.74350	-4.79958	-0.80310
H	2.38726	-3.45369	0.16221
H	0.80836	-4.14587	0.55311
H	1.96988	-3.43767	-2.96409
H	1.29251	-1.79175	-3.02595
H	2.68874	-2.13638	-1.99282
H	-0.30886	-4.38644	-2.32174
H	-1.18114	-3.71752	-0.92882
H	-1.00723	-2.75058	-2.40057

77

Int 13 - Complex 6

C	6.38857	13.78399	9.25212
C	6.19397	15.13434	8.79777
C	7.18228	15.39705	7.78299
C	7.97433	14.22266	7.60511
C	7.47538	13.21337	8.51255
Ir	5.80273	13.68097	7.11417
C	5.65291	11.84789	6.11985
N	6.35701	10.69598	6.28195
C	7.71420	10.69996	5.74572
C	5.28413	16.13095	9.44206
C	7.49329	16.72545	7.17359
C	9.20869	14.14899	6.76597

C	8.11276	11.89892	8.83800
C	5.70249	13.14482	10.41487
N	4.26064	14.37582	5.93583
Mo	3.89848	12.58136	5.79718
N	2.30900	12.12322	7.40315
C	1.54712	13.28763	7.89326
C	4.01996	15.56521	5.11820
C	5.24234	15.82438	4.23077
C	2.79154	15.32156	4.23131
C	3.74135	16.76309	6.03005
O	5.06561	11.92666	4.48258
N	2.08925	11.59297	5.23314
C	1.48944	11.36588	6.40517
O	0.52976	10.70558	6.74670
C	1.56170	10.97718	3.98985
C	0.08914	11.36574	3.79577
C	2.37820	11.50101	2.80831
C	1.70392	9.45173	4.07754
C	2.67418	11.25636	8.53467
C	5.65833	9.45336	5.96802
H	2.19611	13.89771	8.52265
H	0.67352	12.95665	8.46664
H	1.21310	13.88706	7.04593
H	3.27425	10.41912	8.17610
H	1.77322	10.86943	9.02195
H	3.26869	11.83333	9.24265
H	2.21978	12.57524	2.67411
H	2.05640	11.00166	1.88957
H	3.44718	11.32745	2.95378
H	2.75681	9.17498	4.17944
H	1.31614	8.98329	3.16765
H	1.14724	9.06028	4.93121
H	-0.53294	10.97889	4.60352
H	-0.27653	10.96194	2.84668
H	-0.01533	12.45448	3.76159
H	2.97616	14.50471	3.52921
H	2.55974	16.21917	3.65006
H	1.91362	15.07308	4.83430
H	2.86721	16.57394	6.65870
H	3.55254	17.66139	5.43406
H	4.59502	16.95554	6.67941
H	6.13010	15.98463	4.84462
H	5.08448	16.70602	3.60062
H	5.42861	14.95915	3.58987
H	8.31432	9.92452	6.23163
H	8.16371	11.67458	5.91736
H	7.70059	10.51748	4.66249
H	5.52848	9.32826	4.88623
H	4.67546	9.45433	6.44085

H	6.23430	8.61119	6.36279
H	9.54340	13.12107	6.62139
H	10.03232	14.69046	7.24843
H	9.05480	14.58905	5.77840
H	7.38471	11.08552	8.83609
H	8.57969	11.94378	9.82883
H	8.89398	11.64036	8.12337
H	4.67839	13.50629	10.52766
H	6.23678	13.37746	11.34543
H	5.67239	12.05847	10.31462
H	5.08260	16.98206	8.79092
H	5.73339	16.52346	10.36321
H	4.32228	15.68692	9.70807
H	7.91966	16.62456	6.17372
H	8.23022	17.25339	7.79221
H	6.61515	17.36800	7.09949

77

Complex6

Ir	5.89821	13.78601	6.92534
Mo	4.08917	12.71530	5.27963
O	4.43545	12.79267	3.60322
O	0.61696	11.13017	6.56469
N	2.67118	12.11708	7.14459
N	2.31585	11.51345	4.99649
N	4.21237	14.44623	5.91412
N	6.52742	10.93519	5.91748
C	2.04112	13.20027	7.90863
H	2.80630	13.68244	8.51901
H	1.23566	12.81319	8.54267
H	1.63172	13.94141	7.22261
C	3.15438	11.08043	8.06973
H	3.56250	10.24144	7.50471
H	2.33677	10.71557	8.70347
H	3.94824	11.50157	8.68670
C	1.71139	11.51353	6.19365
C	1.59762	10.88865	3.84863
C	2.48435	10.89144	2.60520
H	2.73450	11.90199	2.28468
H	1.95548	10.38164	1.79398
H	3.42420	10.36454	2.78745
C	1.25714	9.42793	4.18877
H	2.17282	8.86358	4.39229
H	0.75748	8.95929	3.33541
H	0.60070	9.36180	5.05601
C	0.31506	11.67919	3.55044
H	-0.35723	11.67083	4.40945
H	-0.20651	11.23776	2.69538
H	0.55923	12.71544	3.29911
C	3.86341	15.73624	5.29471

C	2.58233	15.53773	4.46917
H	2.74661	14.81354	3.66793
H	2.28293	16.48704	4.01426
H	1.75785	15.18821	5.09689
C	3.59516	16.78767	6.36998
H	2.80430	16.45791	7.04875
H	3.28213	17.72875	5.90759
H	4.49301	16.97783	6.95488
C	4.99496	16.17951	4.36122
H	5.92437	16.29635	4.92037
H	4.74834	17.13261	3.88185
H	5.15356	15.42634	3.58580
C	5.73878	12.02375	6.08011
C	7.93532	11.07614	5.58340
H	8.54028	10.31877	6.09282
H	8.26900	12.06928	5.86564
H	8.07823	10.96196	4.49987
C	5.93643	9.71995	5.37065
H	5.98045	9.71299	4.27312
H	4.89357	9.64865	5.67594
H	6.47683	8.84556	5.74779
C	7.98730	14.46348	7.45775
C	7.56542	13.40601	8.34393
C	6.43638	13.88513	9.08842
C	6.17973	15.23846	8.68734
C	7.13726	15.59806	7.68237
C	9.22587	14.50146	6.62123
H	9.65058	13.50930	6.46464
H	9.99415	15.10646	7.11830
H	9.03712	14.94333	5.64081
C	8.27628	12.12260	8.64385
H	7.62611	11.25407	8.51410
H	8.63096	12.12916	9.68027
H	9.14879	11.98398	8.00548
C	5.78607	13.17654	10.23054
H	4.73936	13.46457	10.34356
H	6.29801	13.42068	11.17064
H	5.82838	12.09328	10.10498
C	5.22260	16.14273	9.39780
H	5.16715	17.12725	8.93428
H	5.55405	16.28901	10.43264
H	4.21076	15.73334	9.43093
C	7.39930	16.96523	7.13776
H	7.78213	16.92889	6.11591
H	8.15242	17.47581	7.75166
H	6.50635	17.59128	7.13251

77

Complex 6 - triplet

Ir	5.89315	13.87484	6.80015

Mo	3.98424	12.77949	5.20026
O	4.27548	12.81590	3.50473
O	0.63955	11.08967	6.63414
N	2.67195	12.18425	7.09835
N	2.29997	11.41759	5.01018
N	4.28022	14.56731	5.82245
N	6.51594	11.10589	5.52177
C	2.00414	13.23404	7.87620
H	2.76121	13.78923	8.43139
H	1.26682	12.80214	8.56159
H	1.49435	13.92250	7.20157
C	3.29348	11.20564	8.00559
H	3.71849	10.38956	7.41948
H	2.55156	10.79876	8.70398
H	4.09786	11.69616	8.55394
C	1.71400	11.47849	6.21316
C	1.59662	10.68473	3.92129
C	2.47166	10.63853	2.66986
H	2.68841	11.63554	2.28835
H	1.95315	10.06422	1.89591
H	3.42858	10.15078	2.87233
C	1.31622	9.23991	4.36748
H	2.25382	8.72697	4.60578
H	0.83151	8.69040	3.55467
H	0.66746	9.21139	5.24279
C	0.28086	11.40217	3.58420
H	-0.37782	11.43543	4.45316
H	-0.23642	10.87818	2.77426
H	0.48301	12.42426	3.25093
C	3.87103	15.87066	5.29106
C	2.51316	15.70721	4.59095
H	2.58495	14.98798	3.77080
H	2.18639	16.66485	4.17392
H	1.74617	15.36875	5.29379
C	3.73056	16.90780	6.40413
H	3.00335	16.57953	7.15132
H	3.39674	17.86643	5.99463
H	4.68800	17.06436	6.89875
C	4.91337	16.32810	4.25962
H	5.89092	16.43811	4.73502
H	4.62768	17.28757	3.81489
H	5.00237	15.58299	3.46546
C	5.71778	12.11867	5.84364
C	7.96445	11.19273	5.48889
H	8.42386	10.40960	6.10079
H	8.25946	12.17421	5.84249
H	8.30995	11.07034	4.45550
C	5.97836	9.93238	4.84204
H	6.16310	9.99337	3.76375

H	4.90510	9.87398	5.01362
H	6.46010	9.03045	5.23367
C	7.97630	14.33617	7.55433
C	7.41751	13.32314	8.41395
C	6.35110	13.91010	9.16934
C	6.20667	15.25992	8.74601
C	7.19867	15.52976	7.74420
C	9.29030	14.30845	6.83606
H	9.70804	13.30230	6.77497
H	10.02447	14.92134	7.37360
H	9.21289	14.70224	5.81971
C	8.00718	11.98195	8.73135
H	7.27334	11.17609	8.65123
H	8.39017	11.97932	9.75807
H	8.84355	11.73801	8.07733
C	5.61907	13.27128	10.30262
H	4.57221	13.58188	10.34424
H	6.07606	13.55014	11.26179
H	5.64521	12.18164	10.24090
C	5.30356	16.24654	9.41499
H	5.34421	17.22849	8.94447
H	5.60954	16.37350	10.46012
H	4.26077	15.91988	9.41835
C	7.57295	16.86737	7.18925
H	7.95671	16.78611	6.17029
H	8.36084	17.32479	7.80168
H	6.73232	17.56248	7.17044

61

Complex 2

C	4.31800	9.66262	5.62896
C	5.32044	10.68846	5.64183
C	5.11916	11.47723	6.83108
C	3.99865	10.94223	7.54341
C	3.49977	9.80876	6.79588
Ir	5.58916	9.38815	7.48438
Mo	6.55660	7.99940	9.25586
N	6.63037	9.01405	10.92175
C	7.43003	10.22269	11.01132
C	5.88539	12.70924	7.19425
C	6.31765	10.96336	4.56372
C	3.35310	11.53300	8.75541
C	2.25921	9.03986	7.11343
C	4.11317	8.66908	4.53049
Ir	5.26494	5.94640	9.58438
C	4.26808	4.15105	10.55557
C	3.36957	4.69004	9.58206
C	3.99770	4.62244	8.29357
C	5.30029	4.02354	8.47241
C	5.46762	3.73785	9.86536

C	3.36516	4.94041	6.97789
C	1.98298	5.17772	9.85517
C	6.23300	3.63948	7.36948
C	6.62110	3.01639	10.48666
C	3.97924	3.95145	12.00795
N	8.40832	7.56215	8.82144
C	9.13161	7.81521	7.58917
C	9.23062	6.82653	9.76536
C	5.91267	8.77361	12.15971
H	7.54052	3.16957	9.91913
H	6.42706	1.93747	10.52598
H	6.80339	3.35855	11.50731
H	6.20019	4.36039	6.55083
H	5.96221	2.65628	6.96351
H	7.26463	3.58313	7.71993
H	1.90393	5.61780	10.85103
H	1.26461	4.35114	9.79610
H	1.67784	5.93685	9.13325
H	8.70667	6.68352	10.71052
H	10.16607	7.37000	9.97016
H	9.50116	5.83545	9.36990
H	2.56049	5.66895	7.09019
H	2.93393	4.03868	6.52433
H	4.09183	5.35343	6.27567
H	5.25336	9.62241	12.39902
H	6.61341	8.65587	13.00166
H	5.30682	7.87309	12.07939
H	9.44002	6.86973	7.11653
H	10.04622	8.39710	7.78548
H	8.50811	8.36791	6.88890
H	4.88995	4.01030	12.60728
H	3.52572	2.96831	12.18801
H	3.28879	4.70918	12.38396
H	8.00965	10.37553	10.10083
H	8.13119	10.16204	11.85815
H	6.79587	11.10910	11.16694
H	7.22315	11.42115	4.96695
H	5.90618	11.64804	3.81076
H	6.61467	10.04642	4.05053
H	5.88103	12.87846	8.27226
H	5.44711	13.59239	6.71384
H	6.92729	12.63917	6.87565
H	5.06129	8.37878	4.07365
H	3.47817	9.09263	3.74296
H	3.62925	7.76217	4.89593
H	2.28989	8.04191	6.67394
H	1.37181	9.55180	6.71950
H	2.12843	8.92293	8.19076
H	2.90077	10.76207	9.38148

H	2.56266	12.23759	8.46608
H	4.07386	12.07550	9.36930
H	4.57798	7.44223	9.68093
H	6.06263	7.82981	7.22640
H	6.45847	6.41886	10.59454
H	7.02164	9.71683	8.19244

75

Complex 2 - triplet

C	3.91989	9.37042	5.85929
C	5.08400	10.19727	5.93249
C	5.12119	10.79288	7.25073
C	3.98620	10.31401	7.98327
C	3.25002	9.42585	7.12018
Ir	5.40000	8.57371	7.47904
Mo	6.94109	8.10810	9.52563
N	7.02750	9.53509	10.85897
C	8.00461	10.60576	10.78655
C	6.06220	11.87088	7.68597
C	5.99417	10.53514	4.79353
C	3.52421	10.78713	9.32340
C	1.91990	8.82225	7.43357
C	3.43400	8.65805	4.63734
Ir	4.84908	6.55270	9.20798
C	4.22083	4.56821	10.10874
C	3.31129	4.83169	9.03755
C	4.05704	4.89056	7.81890
C	5.44253	4.61852	8.12820
C	5.54453	4.42475	9.54053
C	3.48609	5.00101	6.44289
C	1.82442	4.93781	9.16206
C	6.52108	4.39729	7.11784
C	6.76337	3.98169	10.28530
C	3.85130	4.30029	11.53362
N	8.72488	7.32747	9.33355
C	9.39712	7.01373	8.08718
C	9.50975	6.92269	10.48667
C	6.16044	9.67078	12.01403
H	7.67298	4.32647	9.79113
H	6.80560	2.88711	10.34951
H	6.77446	4.37426	11.30425
H	6.45750	5.11965	6.30123
H	6.44597	3.39209	6.68356
H	7.51146	4.49288	7.56558
H	1.53058	5.32709	10.13861
H	1.35680	3.95263	9.04228
H	1.40327	5.59845	8.40239
H	8.99250	7.17537	11.41542
H	10.48719	7.42895	10.49477
H	9.69911	5.83790	10.48772

H	2.54260	5.55007	6.44629
H	3.28708	4.00606	6.02387
H	4.16987	5.51943	5.76916
H	5.63486	10.63846	12.00881
H	6.73731	9.61739	12.95056
H	5.40976	8.87914	12.02850
H	9.63507	5.94052	8.01856
H	10.34689	7.56413	8.00320
H	8.77134	7.27819	7.23382
H	4.65920	4.57696	12.21373
H	3.63733	3.23500	11.68848
H	2.96458	4.86347	11.83121
H	8.63538	10.49233	9.90197
H	8.65849	10.60443	11.67245
H	7.52232	11.59471	10.73755
H	6.99163	10.80391	5.14641
H	5.60468	11.38555	4.21949
H	6.10518	9.69355	4.10681
H	6.17933	11.88030	8.77025
H	5.69226	12.85707	7.37828
H	7.05374	11.73773	7.24837
H	4.26238	8.34289	4.00001
H	2.78759	9.31429	4.04138
H	2.85546	7.76875	4.89235
H	1.73093	7.93542	6.82562
H	1.11439	9.53992	7.23074
H	1.85274	8.52648	8.48164
H	3.09407	9.97052	9.90773
H	2.75667	11.56496	9.21918
H	4.34792	11.20754	9.90220
H	3.80334	7.70646	9.48366
H	5.70315	7.19076	6.77878
H	5.34832	7.24160	10.57445
H	6.98743	8.51638	7.35289

79

Complex 3

C	10.75119	10.24724	10.91029
C	11.16299	8.89211	11.18751
C	10.28167	8.35553	12.19880
C	9.28792	9.36969	12.48260
C	9.61179	10.53458	11.70322
Ir	9.12245	8.58275	10.39811
Mo	8.29935	6.70750	9.30507
C	9.36097	5.08039	7.43150
N	10.23640	4.34971	6.68676
C	11.66387	4.38218	6.91043
C	12.40379	8.23661	10.67123
C	10.48133	7.08284	12.95970
C	8.24839	9.29466	13.55442

C	8.86624	11.82981	11.74267
C	11.44209	11.18060	9.96992
Ir	7.42404	8.54896	8.10336
C	6.19232	10.40904	7.25091
C	5.37737	9.25570	7.53768
C	5.83104	8.17090	6.69799
C	6.97522	8.65003	5.95718
C	7.16791	10.04455	6.29127
C	4.16098	9.23564	8.40696
C	5.16493	6.84359	6.52858
C	7.68742	7.90548	4.87331
C	8.20037	10.93676	5.68247
C	6.01622	11.76122	7.86331
O	8.01095	4.75659	10.25124
C	6.81166	5.05677	10.57465
N	6.00543	4.17684	11.20159
C	4.64732	4.53147	11.55219
O	6.41224	6.23198	10.27262
O	8.13312	5.05585	7.24248
O	9.88170	5.82339	8.38019
C	6.44953	2.82780	11.47820
C	9.74584	3.46565	5.65544
H	7.48162	2.72126	11.15146
H	5.82707	2.10031	10.94404
H	6.38624	2.61664	12.55173
H	4.48346	5.58115	11.31847
H	4.47666	4.36927	12.62244
H	3.92907	3.92199	10.99073
H	4.00723	8.24840	8.84548
H	3.26341	9.49535	7.83114
H	4.24709	9.94974	9.22855
H	5.89437	6.06559	6.29901
H	4.42635	6.88571	5.71781
H	4.64896	6.54675	7.44320
H	7.67889	6.83177	5.06545
H	8.72991	8.22120	4.79362
H	7.20810	8.08598	3.90242
H	8.43764	11.78179	6.33182
H	7.85007	11.34401	4.72564
H	9.12955	10.39651	5.48915
H	5.68513	11.69145	8.90149
H	5.26343	12.34149	7.31564
H	6.94621	12.33284	7.84939
H	8.66016	3.53032	5.62820
H	10.03952	2.42887	5.86209
H	10.14967	3.74778	4.67512
H	11.88639	5.09752	7.69797
H	12.18942	4.68053	5.99476
H	12.03362	3.39308	7.20973

H	13.27167	8.51173	11.28398
H	12.61230	8.53473	9.64188
H	12.31146	7.14967	10.68419
H	9.53106	6.66979	13.30139
H	11.11088	7.25867	13.84146
H	10.96669	6.32265	12.34537
H	7.90565	8.26955	13.70401
H	7.37419	9.89997	13.30646
H	8.64713	9.65917	14.51006
H	7.79943	11.67398	11.91704
H	8.97235	12.38426	10.80817
H	9.24134	12.47055	12.55023
H	11.88965	10.64277	9.13155
H	12.24637	11.72766	10.47814
H	10.75326	11.91984	9.55600
H	9.13233	8.44790	8.44591
H	7.53740	9.12269	9.79657

79

Complex 3 - triplet

C	10.67534	10.35770	11.02169
C	11.13695	8.98563	10.81887
C	10.41638	8.12200	11.71909
C	9.38094	8.92626	12.31133
C	9.60220	10.31760	11.91581
Ir	9.07071	8.79639	10.15406
Mo	8.14674	6.67137	9.17965
C	9.30369	4.94257	7.60528
N	10.09614	4.11788	6.88040
C	11.53897	4.19024	6.94883
C	12.34385	8.60210	10.02274
C	10.75283	6.70276	12.04490
C	8.43833	8.48501	13.38593
C	8.79155	11.47100	12.40926
C	11.26079	11.56166	10.35881
Ir	7.47756	8.82487	8.06612
C	6.11438	10.51957	7.04530
C	5.45628	9.25734	7.37290
C	6.03409	8.21511	6.56254
C	7.17324	8.79550	5.90440
C	7.16726	10.23455	6.17035
C	4.20991	9.13272	8.19011
C	5.48478	6.83785	6.37135
C	8.03534	8.11407	4.89000
C	8.13838	11.20262	5.57831
C	5.71459	11.85518	7.58181
O	8.07043	4.96697	10.86471
C	6.82641	5.14977	10.83026
N	5.96375	4.44243	11.59688
C	4.53451	4.65439	11.53245

O	6.35298	6.05440	10.02630
O	8.04865	4.87742	7.57366
O	9.86302	5.83807	8.36336
C	6.44874	3.39184	12.46391
C	9.51423	3.06808	6.07419
H	7.53309	3.34696	12.38855
H	6.02732	2.42299	12.16980
H	6.16589	3.58989	13.50447
H	4.32710	5.48347	10.86047
H	4.13792	4.88879	12.52724
H	4.02570	3.75628	11.16134
H	4.10783	8.12653	8.59863
H	3.32316	9.34557	7.57951
H	4.21286	9.83141	9.02939
H	6.27252	6.11121	6.16988
H	4.77285	6.82403	5.53595
H	4.96327	6.49771	7.26755
H	8.09250	7.04267	5.08724
H	9.05367	8.50848	4.89983
H	7.63168	8.25284	3.87860
H	8.19635	12.12465	6.15964
H	7.84417	11.46979	4.55578
H	9.14293	10.77672	5.52746
H	5.43019	11.79575	8.63503
H	4.85286	12.25549	7.03293
H	6.52453	12.58256	7.49861
H	8.43032	3.14388	6.12831
H	9.82433	2.08148	6.43978
H	9.83251	3.16471	5.02964
H	11.82463	5.04021	7.56352
H	11.96190	4.31328	5.94506
H	11.95408	3.27472	7.38823
H	13.25612	8.71456	10.62228
H	12.44879	9.22918	9.13494
H	12.27992	7.56577	9.68888
H	9.86007	6.11084	12.24907
H	11.41393	6.65828	12.91995
H	11.26370	6.22188	11.20927
H	8.22701	7.41774	13.30682
H	7.48642	9.01743	13.32835
H	8.86670	8.67280	14.37912
H	7.73535	11.20589	12.49466
H	8.86606	12.33308	11.74382
H	9.13089	11.78663	13.40364
H	11.51936	11.35936	9.31680
H	12.17914	11.88091	10.86726
H	10.56729	12.40479	10.36948
H	9.16813	9.18352	8.42664
H	7.45949	9.38963	9.74381

References

(1) *Ether Complexes of Molybdenum(III) and Molybdenum(IV) chlorides - Maria - 2014 - Inorganic Syntheses - Wiley Online Library*. https://onlinelibrary-wiley-com.docelec.univ-lyon1.fr/doi/10.1002/9781118744994.ch03 (accessed 2024-06-07).

(2) Gilbert, T. M.; Hollander, F. J.; Bergman, R. G. (Pentamethylcyclopentadienyl)Iridium Polyhydride Complexes: Synthesis of Intermediates in the Mechanism of Formation of (Pentamethylcyclopentadienyl)Iridium Tetrahydride and the Preparation of Several Iridium(V) Compounds. *J. Am. Chem. Soc.* **1985**, *107* (12), 3508–3516. https://doi.org/10.1021/ja00298a018.

(3) Cadot, S.; Renault, O.; Frégnaux, M.; Rouchon, D.; Nolot, E.; Szeto, K.; Thieuleux, C.; Veyre, L.; Okuno, H.; Martin, F.; Alessandra Quadrelli, E. A Novel 2-Step ALD Route to Ultra-Thin MoS 2 Films on SiO 2 through a Surface Organometallic Intermediate. *Nanoscale* **2017**, *9* (2), 538–546. https://doi.org/10.1039/C6NR06021H.

(4) *CrysAlisPro Software System*; Rigaku Oxford Diffraction.

(5) Clark, R. C.; Reid, J. S. The Analytical Calculation of Absorption in Multifaceted Crystals. *Acta Cryst A* **1995**, *51* (6), 887–897. https://doi.org/10.1107/S0108767395007367.

(6) Sheldrick, G. M. SHELXT – Integrated Space-Group and Crystal-Structure Determination. *Acta Cryst A* **2015**, *71* (1), 3–8. https://doi.org/10.1107/S2053273314026370.

(7) Dolomanov, O. V.; Bourhis, L. J.; Gildea, R. J.; Howard, J. a. K.; Puschmann, H. OLEX2: A Complete Structure Solution, Refinement and Analysis Program. *J Appl Cryst* **2009**, *42* (2), 339–341. https://doi.org/10.1107/S0021889808042726.

(8) Fulmer, G. R.; Miller, A. J. M.; Sherden, N. H.; Gottlieb, H. E.; Nudelman, A.; Stoltz, B. M.; Bercaw, J. E.; Goldberg, K. I. NMR Chemical Shifts of Trace Impurities: Common Laboratory Solvents, Organics, and Gases in Deuterated Solvents Relevant to the Organometallic Chemist. *Organometallics* **2010**, *29* (9), 2176–2179. https://doi.org/10.1021/om100106e.

(9) Chisholm, M. H.; Cotton, F. A.; Frenz, B. A.; Reichert, W. W.; Shive, L. W.; Stults, B. R. The Molybdenum-Molybdenum Triple Bond. 1. Hexakis(Dimethylamido)Dimolybdenum and Some Homologs: Preparation, Structure, and Properties. *J. Am. Chem. Soc.* **1976**, *98* (15), 4469–4476. https://doi.org/10.1021/ja00431a024.

(10) Chisholm, M. H.; Cotton, F. A.; Extine, M.; Stults, B. R. The Tungsten-Tungsten Triple Bond. 1. Preparation, Properties, and Structural Characterization of Hexakis(Dimethylamido)Ditungsten(III) and Some Homologs. *J. Am. Chem. Soc.* **1976**, *98* (15), 4477–4485. https://doi.org/10.1021/ja00431a025.

(11) Garner, C. D.; Senior, R. G.; King, T. J. Preparation and Characterization of Chromium(II) Molybdenum(II) Tetraacetate. A Compound Containing a Heteronuclear Quadruple Metal-Metal Bond. *J. Am. Chem. Soc.* **1976**, *98* (12), 3526–3529. https://doi.org/10.1021/ja00428a022.

(12) Brogden, D. W.; Turov, Y.; Nippe, M.; Li Manni, G.; Hillard, E. A.; Clérac, R.; Gagliardi, L.; Berry, J. F. Oxidative Stretching of Metal–Metal Bonds to Their Limits. *Inorg. Chem.* **2014**, *53* (9), 4777–4790. https://doi.org/10.1021/ic5007204.

(13) Cotton, F. A.; Dunbar, K. R.; Hong, B.; James, C. A.; Matonic, J. H.; Thomas, J. L. C. Complexes Containing Heteronuclear and Homonuclear Quadruple Bonds. Preparation and Characterization of Bis[Bis(Dimethylphosphino)Methane]Tetrachloromolybdenumtungsten and Mo2X4(Dmpm)2 (X = Bromide, Iodide). *Inorg. Chem.* **1993**, *32* (23), 5183–5187. https://doi.org/10.1021/ic00075a040.

(14) Carriedo, G. A.; Howard, J. A. K.; Jeffery, J. C.; Sneller, K.; Stone, F. G. A.; Weerasuria, A. M. M. Chemistry of Polynuclear Metal Complexes with Bridging Carbene or Carbyne Ligands.

Part 97. Synthesis and Crystal Structures of the Molybdenumditungsten Compounds [MoW2{μ-σ,Σ′ : H4-C(Ph)C(Ph)C(C6H4Me-4)C(C6H4Me-4)}(CO)6(η-C5H5)2]·2CH2Cl2 and [MoW2(μ-C2Ph2){μ-C2(C6H4Me-4)2}(CO)4(η-C5H5)2]·0.5CH2Cl2. *J. Chem. Soc., Dalton Trans.* **1990**, No. 3, 953–958. https://doi.org/10.1039/DT9900000953.

(15) Alvarez, M. A.; Garcia, M. E.; Riera, V.; Ruiz, M. A.; Bois, C.; Jeannin, Y. C-H Cleavages in the Photoreactions of [M2(.Eta.5-C5H5)2(CO)6] (M = Mo, W): Isolation and Characterization of the V-Shaped Trinuclear Clusters [M2M′(.Mu.-.Eta.1,.Eta.5-C5H4)(.Eta.5-C5H5)2(CO)6] (M, M′ = Mo or W). *J. Am. Chem. Soc.* **1995**, *117* (4), 1324–1335. https://doi.org/10.1021/ja00109a017.

(16) Vicic, D. A.; Jones, W. D. Activation of Sulfur- and Nitrogen-Containing Heterocycles by a Dinuclear Iridium Complex. *Organometallics* **1999**, *18* (2), 134–138. https://doi.org/10.1021/om9808007.

(17) Banta, G. A.; Louie, B. M.; Onyiriuka, E.; Rettig, S. J.; Storr, A. Synthesis and Characterization of LMo(CO)3M(PPh3)x (Where L = Tridentate Pyrazolyl-Gallate or -Borate Ligand; M = Rh, x = 2; M = Cu, x = 1). X-Ray Crystal Structures of [MeGapz3]Mo(CO)3Rh(PPh3)2 and [MeGapz3]Mo(CO)3Cu(PPh3) (Where Pz = Pyrazolyl, N2C3H3). *Can. J. Chem.* **1986**, *64* (2), 373–386. https://doi.org/10.1139/v86-061.

(18) Shima, T.; Ito, J.; Suzuki, H. Synthesis, Characterization, and Structure Determination of the Heterobimetallic Polyhydride Complexes (C5Me5)Ru(μ-H)3MH3(C5Me5) (M = Mo, W) Containing Group VI and Group VIII Metals. *Organometallics* **2001**, *20* (1), 10–12. https://doi.org/10.1021/om000769k.

(19) Collman, J. P.; Harford, S. T.; Franzen, S.; Shreve, A. P.; Woodruff, W. H. Resonance Raman and X-Ray Crystallographic Studies of Intertriad Metal−Metal Bonds. 2. WRu and MoOs Porphyrin Dimers. *Inorg. Chem.* **1999**, *38* (9), 2093–2097. https://doi.org/10.1021/ic9810337.

(20) Chisholm, M. H.; Johnston, V. J.; Eisenstein, O.; Streib, W. E. Synthesis, Structure, and Bonding of [CpCoW2(OCH2tBu)6], and Comments on the Combining Properties of CO and CpCo. *Angewandte Chemie International Edition in English* **1992**, *31* (7), 896–898. https://doi.org/10.1002/anie.199208961.

(21) Stevens, R. C.; Mclean, M. R.; Wen, T.; Carpenter, J. D.; Bau, R.; Koetzle, T. F. An X-Ray and Neutron Diffraction Structure Analysis of a Triply-Bridged Binuclear Iridium Complex, [[(C5(CH3)5Ir)2(μ-H)3]+ [ClO4]−• 2C6H6]. *Inorganica Chimica Acta* **1989**, *161* (2), 223–231. https://doi.org/10.1016/S0020-1693(00)83097-2.

(22) Crabtree, R. H.; Felkin, H.; Morris, G. E.; King, T. J.; Richards, J. A. Hr2(μ-H)3H2(PPh3)4]+PF6−, A Novel Iridium Complex Containing a Metal Metal Triple Bond. *Journal of Organometallic Chemistry* **1976**, *113* (1), C7–C9. https://doi.org/10.1016/S0022-328X(00)91767-4.

(23) Carmona, D.; Ferrer, J.; Mendoza, A.; Lahoz, F. J.; Reyes, J.; Oro, L. A. Reversible Isomerization of the Dinuclear Complex [(H6-p-Cymene)RuCl(μ-Pz)2 Ir(CO)2] with Formation of a Ruthenium-Iridium Bond. *Angewandte Chemie International Edition in English* **1991**, *30* (9), 1171–1173. https://doi.org/10.1002/anie.199111711.

(24) M. J. Frisch, G. W. Trucks, H. B. Schlegel, G. E. Scuseria, M. A. Robb, J. R. Cheeseman, G. Scalmani, V. Barone, G. A. Petersson, H. Nakatsuji, X. Li, M. Caricato, A. Marenich, J. Bloino, B. G. Janesko, R. Gomperts, B. Mennucci, H. P. Hratchian, J. V. Ortiz, A. F. Izmaylov, J. L. Sonnenberg, D. Williams-Young, F. Ding, F. Lipparini, F. Egidi, J. Goings, B. Peng, A. Petrone, T. Henderson, D. Ranasinghe, V. G. Zakrzewski, J. Gao, N. Rega, G. Zheng, W. Liang, M. Hada, M. Ehara, K. Toyota, R. Fukuda, J. Hasegawa, M. Ishida, T. Nakajima, Y. Honda, O. Kitao, H. Nakai, T. Vreven, K. Throssell, J. A. Montgomery, Jr., J. E. Peralta, F. Ogliaro, M. Bearpark, J. J. Heyd, E. Brothers, K. N. Kudin, V. N. Staroverov, T. Keith, R. Kobayashi, J. Normand, K. Raghavachari, A. Rendell, J. C. Burant, S. S. Iyengar, J. Tomasi, M. Cossi, J. M. Millam, M.

Klene, C. Adamo, R. Cammi, J. W. Ochterski, R. L. Martin, K. Morokuma, O. Farkas, J. B. Foresman, and D. J. Fox. *Gaussian 09*; Gaussian Inc: WAllingford, CT.
(25) Perdew, J. P.; Chevary, J. A.; Vosko, S. H.; Jackson, K. A.; Pederson, M. R.; Singh, D. J.; Fiolhais, C. Atoms, Molecules, Solids, and Surfaces: Applications of the Generalized Gradient Approximation for Exchange and Correlation. *Phys. Rev. B* **1992**, *46* (11), 6671–6687. https://doi.org/10.1103/PhysRevB.46.6671.
(26) Becke, A. D. Density-functional Thermochemistry. III. The Role of Exact Exchange. *The Journal of Chemical Physics* **1993**, *98* (7), 5648–5652. https://doi.org/10.1063/1.464913.
(27) Cao, X.; Dolg, M.; Stoll, H. Valence Basis Sets for Relativistic Energy-Consistent Small-Core Actinide Pseudopotentials. *The Journal of Chemical Physics* **2003**, *118* (2), 487–496. https://doi.org/10.1063/1.1521431.
(28) Cao, X.; Dolg, M. Segmented Contraction Scheme for Small-Core Actinide Pseudopotential Basis Sets. *Journal of Molecular Structure: THEOCHEM* **2004**, *673* (1), 203–209. https://doi.org/10.1016/j.theochem.2003.12.015.
(29) Andrae, D.; Häußermann, U.; Dolg, M.; Stoll, H.; Preuß, H. Energy-Adjustedab Initio Pseudopotentials for the Second and Third Row Transition Elements. *Theoret. Chim. Acta* **1990**, *77* (2), 123–141. https://doi.org/10.1007/BF01114537.
(30) Ehlers, A. W.; Böhme, M.; Dapprich, S.; Gobbi, A.; Höllwarth, A.; Jonas, V.; Köhler, K. F.; Stegmann, R.; Veldkamp, A.; Frenking, G. A Set of F-Polarization Functions for Pseudo-Potential Basis Sets of the Transition Metals Sc⍰Cu, Y⍰Ag and La⍰Au. *Chemical Physics Letters* **1993**, *208* (1), 111–114. https://doi.org/10.1016/0009-2614(93)80086-5.
(31) McLean, A. D.; Chandler, G. S. Contracted Gaussian Basis Sets for Molecular Calculations. I. Second Row Atoms, Z=11–18. *The Journal of Chemical Physics* **1980**, *72* (10), 5639–5648. https://doi.org/10.1063/1.438980.
(32) Krishnan, R.; Binkley, J. S.; Seeger, R.; Pople, J. A. Self-consistent Molecular Orbital Methods. XX. A Basis Set for Correlated Wave Functions. *The Journal of Chemical Physics* **1980**, *72* (1), 650–654. https://doi.org/10.1063/1.438955.
(33) Frisch, M. J.; Pople, J. A.; Binkley, J. S. Self-consistent Molecular Orbital Methods 25. Supplementary Functions for Gaussian Basis Sets. *The Journal of Chemical Physics* **1984**, *80* (7), 3265–3269. https://doi.org/10.1063/1.447079.
(34) Reed, A. E.; Curtiss, L. A.; Weinhold, F. Intermolecular Interactions from a Natural Bond Orbital, Donor-Acceptor Viewpoint. *Chem. Rev.* **1988**, *88* (6), 899–926. https://doi.org/10.1021/cr00088a005.
